# Cross-platform Hi-C meta-analysis identifies functional insulators that actively block enhancer-promoter interactions

**DOI:** 10.1038/s41467-026-75801-3

**Published:** 2026-07-16

**Authors:** Jian Cui, Wanying Xu, Xiuyuan Lang, Shanshan Zhang, Leina Lu, Xiaoxiao Liu, Yan Li, Fulai Jin

**Affiliations:** 1 Department of Genetics and Genome Sciences, School of Medicine, Case Western Reserve University, Cleveland, OH USA; 2 The Biomedical Sciences Training Program (BSTP), School of Medicine, Case Western Reserve University, Cleveland, Ohio, USA; 3 Department of Computer and Data Sciences, School of Engineering, Case Western Reserve University, Cleveland, OH USA; 4 Department of Population and Quantitative Health Sciences, School of Medicine, Case Western Reserve University, Cleveland, Ohio, USA; 5 Case Comprehensive Cancer Center, Case Western Reserve University, Cleveland, OH USA

**Keywords:** Transcriptional regulatory elements, Gene regulatory networks, Epigenomics, Gene regulation, Chromatin structure

## Abstract

The function of topologically associating domain (TAD) boundaries as transcriptional insulators remains a fundamental controversy in genome biology. Here, we demonstrate that bona fide Functional Insulators (FINs) should be defined dynamically by their capability to block architectural rewiring. Leveraging *DeepLoop* to enable robust cross-platform Hi-C analysis, we performed a meta-analysis of nine high-resolution 3D genomic datasets following acute CTCF or cohesin depletion, mapping FINs genome-wide. We show that CTCF loss triggers the reproducible formation of new enhancer-promoter loops at only a few hundred specific loci. These loops are cohesin-dependent, enriched at G-rich *cis*-regulatory elements, and directly drive recurrent, early-response gene activation. Multiplexed CTCF-displacement assays functionally confirmed the causal role of FINs in these localized rewiring events. Crucially, FINs reside within active euchromatin, infrequently coincide with traditional TAD boundaries, and are sensitive to WAPL depletion. Our results reveal that the genome’s functional insulation is mediated by these discrete, dynamically active sites rather than static TAD boundaries.

## Abstract (web summary)

The authors map de-repressed enhancer-promoter interactions after CTCF loss to show that Functional Insulators (FINs), defined by dynamic loop rewiring rather than static TAD boundaries, are true insulators blocking local gene activation.

## Introduction

It is generally accepted that topologically associating domains (TADs) confine the communication between genes and enhancers to facilitate transcriptional control^1–6^. Most TADs are maintained by CTCF and cohesin, which are widely recognized as insulator proteins. Cohesin is a multimeric complex consisting of SMC1A, SMC3, RAD21, and one SA subunit (STAG1 or STAG2). TADs form when a DNA loop extrudes through the cohesin ring before being blocked by CTCF binding sites in a convergent orientation, with cohesin serving as the enzymatic motor for this process. The depletion of CTCF or the cohesin core subunit RAD21 leads to the near-complete loss of TADs^7,8^. Cohesin function is additionally regulated by the loading complex MAU2/NIPBL^9^ and the release factor WAPL^10–12^. While deleting MAU2 or NIPBL results in the disappearance of TADs and a massive reduction of chromatin loops^13,14^, the loss of WAPL produces the opposite effect, generating larger loops by enabling cohesin to bypass CTCF barriers through prolonged loop extrusion^14,15^.

**Fig. 1:**
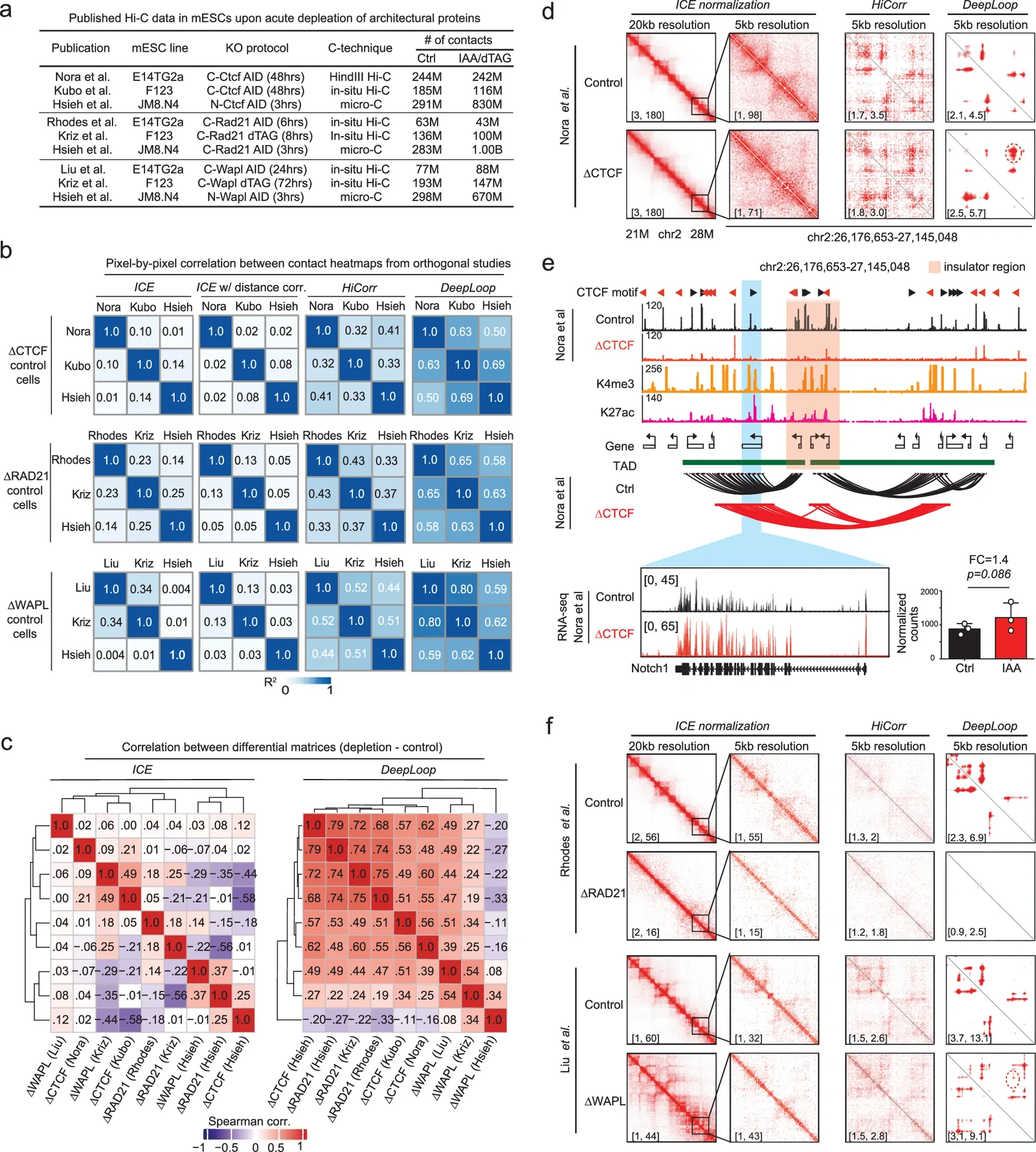
*DeepLoop* allows cross-platform comparison between independent CTCF- and cohesin-degron Hi-C datasets at loop-resolution. **a** A list of published Hi-C and micro-C studies with acute depletion of CTCF, RAD21, or WAPL in mESCs. **b** Matrices summarizing R-square values when comparing contact heatmaps from different pipelines and orthogonal Hi-C/micro-C datasets in control mESCs. **c** Correlation matrices comparing the differential matrices of all nine degron experiments. For each degron experiment, the differential matrix is the difference between two Hi-C contact matrices (depletion—control) at 5 kb resolution. Left, Hi-C contact matrices from *ICE* are used to compute differential matrices; right, Hi-C contact matrices from *DeepLoop* are used. **d** Contact heatmaps of the CTCF-degron Hi-C data at *Notch1* locus with *ICE*, *HiCorr,* and *DeepLoop*. Red dashed circle: gained loops. Numbers in brackets indicate the color scale of heatmaps: for *ICE* normalization and *HiCorr* heatmaps, the maximum intensity corresponds to the 98th percentile of the corresponding contact matrix; for *DeepLoop* heatmaps, the maximum intensity corresponds to the lower cutoff of top 500,000 pixels genome-wide. **e** Genome browser tracks showing ChIP-seq data and Hi-C loops from Nora et al. at *Notch1* locus. Blue highlighted region showing Notch1 gene which is at the anchor of a gained loop. Pink region highlights the potential insulator region that blocks the formation of *Notch1* E-P loop. Bar plot: quantitation of *Notch1* expression from RNA-seq data provided by Nora et al. Error bars: mean ± SEM. *P* value: one-side Welch *t*test (*n* = 3, biological replicates). **f** Contact heatmaps of the RAD21- and WAPL-degron Hi-C data at *Notch1* locus with *ICE*, *HiCorr*, and *DeepLoop* for the same genomic region as in (**d**).

Insulators are canonically defined as DNA elements that inhibit gene expression when positioned between enhancers and promoters^16–18^. The discovery of thousands of CTCF-demarcated TADs immediately suggests a model wherein CTCF sites at TAD boundaries function as enhancer-blocking insulators, a premise supported by genetic experiments at selected loci^19–23^. However, this model was challenged by a genome-wide Hi-C study in mouse embryonic stem cells (mESCs) (Nora et al., Fig. 1a), which observed only mild transcriptional changes despite the near complete loss of TADs following CTCF depletion^7^. This conclusion was corroborated by two subsequent genome-wide studies utilizing in situ Hi-C and micro-C^24,25^ (Fig. 1a). Notably, Kubo et al. reported a role for CTCF in mediating enhancer-promoter (E-P) interactions^24^, while Hsieh et al. observed many short-range, cohesin-independent cross-TAD E-P interactions^25^. Although these studies critically questioned the insulating function of TAD boundaries, they left unresolved the fundamental question regarding the genomic identity and location of bona fide enhancer-blocking insulators.

In this study, we demonstrated that “functional insulators” (FINs) can be identified by examining whether their flanking regions gain E-P interactions upon CTCF depletion. Mapping FINs using genome-wide chromosome conformation capture technologies presents several distinct challenges: (i) most public Hi-C datasets lack the billion-scale read depth typically required for robust loop-level analysis (Fig. 1a); (ii) E-P interactions are generally weaker than CTCF loops in Hi-C data and are therefore inherently harder to detect; (iii) the gain of E-P loop signal can be subtle, necessitating robust quantitation of loop strength; and (iv) it is difficult to definitively rule out the possibility that some loop changes are secondary effects arising from prolonged CTCF or cohesin depletion^7,25^. While these challenges can theoretically be mitigated by cross-referencing independent datasets, existing CTCF-depletion data are generated using diverse Hi-C platforms that exhibit significant performance variations in loop-level analyses when processed with conventional pipelines^26^. To overcome this, we leverage the *HiCorr* and *DeepLoop* computational pipelines we previously developed^27,28^ to enable the robust comparison of loop profiles from orthogonal Hi-C and micro-C platforms, even at lower sequencing depth. By applying *DeepLoop* to conduct a meta-analysis of diverse Hi-C datasets, we map recurrent DNA rewiring events upon the acute depletion of CTCF and cohesin components (Fig. 1a), ultimately generating a reliable, genome-wide map of enhancer-blocking insulators.

## Results

### *DeepLoop* enables cross-platform comparison of independent CTCF- and cohesin-degron Hi-C datasets at loop-resolution

We collected nine pairs of independent Hi-C or micro-C datasets from six publications in which CTCF^7,24,25^, RAD21^25,29,30^, or WAPL^25,29,31^ were acutely depleted in three different mESC lines. The number of valid contacts across these Hi-C/micro-C datasets is highly variable, ranging from 43 million to approximately 1 billion. For each target protein, the choice of degron system (AID or dTAG) and the duration of protein degradation (ranging from 3 h to 3 days) also varied considerably (Fig. 1a).

To assess how these technical and biological variations complicate cross-platform comparisons, we first performed low-resolution compartment- and TAD-level analyses on the three CTCF-degron datasets using conventional pipelines. At the compartment level (500 kb resolution), all datasets displayed highly consistent compartment correlation heatmaps, and the broad compartmental organization was largely preserved following CTCF depletion (Supplementary Fig. 1a–c). When plotting *ICE*-normalized contact matrices^32^ at 40 kb resolution followed by directionality index (DI)-based TAD analyses, all three studies consistently demonstrated similar TAD structures and a massive loss of TADs upon CTCF depletion, despite a trend where micro-C detected more bins with stronger directionality biases (Supplementary Fig. 1d–f). These observations corroborate the findings from the original reports and confirm that conventional pipelines are generally robust across different Hi-C platforms at low-resolution.

However, high-resolution Hi-C analysis is severely constrained by data sparsity and protocol biases^27,33^, making the reliability of cross-platform comparisons highly dependent on the choice of analytical pipelines. We generated 5 kb resolution contact heatmaps (where each pixel represents a pair of 5 kb bins) using conventional *ICE*^32^ (with or without distance correction), *HiCorr*^27^, and *DeepLoop*^28^. We then utilized scatter plots to compare control cell heatmaps from independent experiments, assessing the consistency of these pipelines when analyzing orthogonal Hi-C platforms (Supplementary Fig. 2). We restricted this comparison to control heatmaps because, prior to protein depletion, the control cells from all studies are expected to exist in similar biological states. In contrast, protein-depleted cells from independent studies, even when the same protein is targeted, can exhibit substantial diversity due to variations in the duration of degradation, incomplete depletion, or secondary effects like the loss of stemness. We found that while conventional *ICE* pipelines show poor cross-platform consistency, with R-square values often approaching zero, *HiCorr* and *DeepLoop* drastically improved consistency, yielding much higher cross-platform R-square values (Supplementary Fig. 2, summarized in Fig. 1b).

We additionally use scatter plots to compare the heatmaps pixel-by-pixel before and after protein depletion. Only the *HiCorr* and *DeepLoop* heatmaps showed recognizable populations of loop pixels that retained their strength upon CTCF or RAD21 depletion (Supplementary Fig. 2), highlighting their robustness in revealing the unchanged loop pixels that are otherwise masked by biases and noise in conventional pipelines. To further evaluate cross-platform consistency when analyzing dynamic loops, we generated differential heatmaps by subtracting each pair of 5kb-resolution contact matrices (depletion minus control), and subsequently computed the correlations between these differential matrices across all nine pairs of degron experiments (Fig. 1c, Supplementary Fig. 3). Only the matrices generated by *HiCorr* and *DeepLoop* successfully clustered the CTCF- and RAD21-degron datasets together while properly separating out the WAPL-degron datasets (Fig. 1c, Supplementary Fig. 3). This clustering accurately reflects the biological expectation that the depletion of CTCF or RAD21, but not WAPL, causes a massive loss of TADs and CTCF loops.

Taken together, these results support the robustness of *DeepLoop* for cross-platform loop analysis, regardless of initial sequencing depth. We attribute this improved robustness to the fact that the HiCorr/DeepLoop pipeline prioritizes loop signals through rigorous bias correction and normalization^27,28^. This process effectively eliminates signals arising from both platform-specific technical biases (*e.g*., restriction site density and distribution) and non-loop chromatin interactions, including non-specific local chromatin compaction and other broader structural features. While the non-loop architectural signals retained by ICE-based normalization may carry biological meaning, they are often highly sensitive to protocol variations in kilobase-resolution analyses. At this scale, such features can act as confounding noise, compromising the consistent and accurate quantification of loop strength across disparate datasets.

### Hi-C meta-analysis reveals recurrent E-P loop-gaining events upon CTCF depletion

We first focused on the CTCF-degron Hi-C datasets. In the example of Fig. 1d, with conventional *ICE*-normalization, we observed a dampened TAD structure at 20 kb resolution as the original study reported^7^, but at 5 kb resolution, loop signals from *ICE*-normalized heatmaps were weak and often invisible. From the *DeepLoop*-enhanced heatmaps, we consistently observed multiple CTCF loops that appeared in all control cell heatmaps but were substantially weakened upon CTCF depletion. A notable change in this region was the gain of long-range loops (Fig. 1d, loop pixels in dashed circle) connecting the *Notch1* gene to a distal enhancer region with multiple H3K27ac peaks (Fig. 1e, red arches). Consistent with the gain of an E-P loop, *Notch1* was upregulated upon CTCF-depletion (Fig. 1e). The gain of loop signals at this locus was also observable in one of the WAPL-degron studies (Fig. 1f, bottom two rows) and two other CTCF-degron studies (Supplementary Fig. 4a–f), but not in the RAD21-depleted cells (Fig. 1f, top two rows, and Supplementary Fig. 4g). These observations suggest that the CTCF loop anchors at the TAD boundary region (Fig. 1e, highlighted in pink) actively block the *Notch1*-enhancer interaction. This example also demonstrates that *DeepLoop* can detect recurrent insulating events from CTCF-degron Hi-C data without the need for ultra-high sequencing depth.

**Fig. 2:**
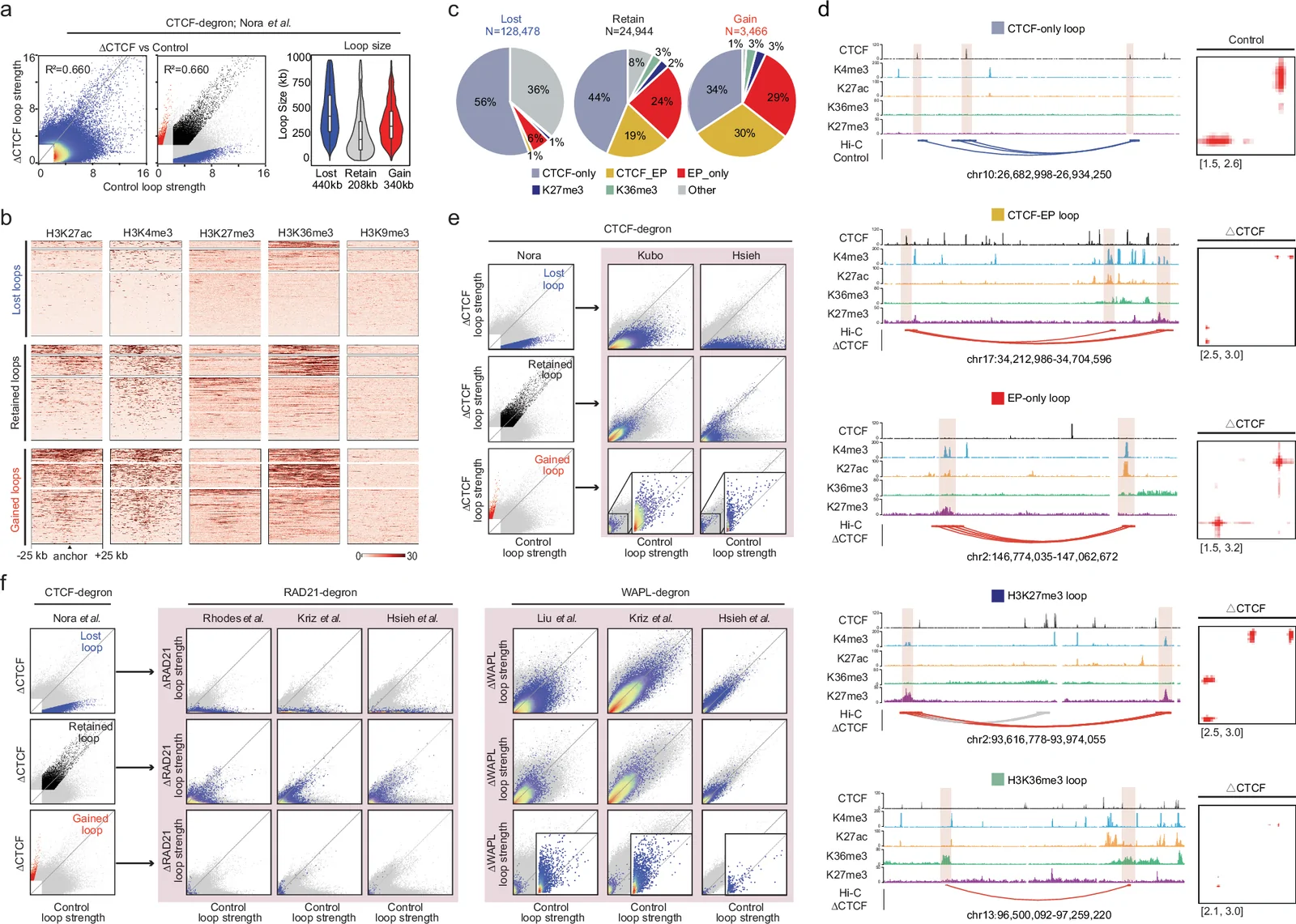
Identify and characterize recurrent gain of E-P loop events in CTCF-depleted cells through cross-platform Hi-C meta-analyses. **a** Left: scatterplot comparing the strength of loop pixels between control and CTCF-depleted mESCs (every dot represents one pixel in the contact heatmap). Middle: the same scatterplot as left but highlight the lost (blue), retained (black), and gained (red) loop pixels. Right: violin plot shows the size distribution of loop pixels (total pixel numbers show in **c**); median loop sizes of all categories are included in the x-labels; box in the violin show median ± upper and lower quartiles. **b** Heatmaps of ChIP-seq signal for histone marks at the anchors of lost, retained, and gained loop pixels in CTCF-depleted cells. The centers of loop anchors are aligned and the heatmaps plotted the regions ±25 kb of the anchor centers. **c** Pie charts showing the fractions of various types among lost, retained and gained loop pixels. **d** Representative example of different types of chromatins. Each panel shows genome browser tracks with loop arches together with DeepLoop heatmaps of the corresponding region. **e** Scatter plots showing the consistency of lost, retained and gained loop pixels between three CTCF degron studies. Plots in the left column highlight the lost, retained, and gained loop pixels from Nora et al., the right two columns show the quantitative changes of the corresponding pixels in Kubo et al. or Hsieh et al. **f** The lost, retained, and gained loop pixels from one CTCF-degron Hi-C data (Nora et al.) are plotted onto Hi-C scatter plots from RAD21-or WAPL-degron studies to examine the quantitative changes of loop strength upon cohesin protein depletion.

To perform a genome-wide analysis of insulating events, we identified 300,000 to 500,000 loop pixels from each CTCF-degron dataset and quantified loop strength using *DeepLoop* pixel intensities (Fig. 2a, Supplementary Fig. 5). Across all studies, the scatter plots were clearly down-skewed, reflecting a massive loss of chromatin loops following CTCF depletion (Fig. 2a, Supplementary Fig. 5a, d, left panels). Using a fold-change cutoff of > 4, we categorized loop pixels that lost strength (“lost”), retained strength (“retained”), or gained strength (“gained”) (Fig. 2a, Supplementary Fig. 5a, d, middle panels). We observed a clear trend in which the anchors of “retained” and “gained” loop pixels were more enriched for H3K27ac (enhancers), H3K4me3 (promoters), H3K27me3 (Polycomb-repressed sites), and H3K36me3 (transcribed gene bodies) compared to “lost” loop pixels (Fig. 2b). This suggests that, in the absence of CTCF, chromatin loops preferentially anchor at these epigenetically marked regions.

To quantify this observation, we classified the loop pixels based on their epigenetic signatures in wild type mESCs (“Methods”). “CTCF-only” loop pixels, defined as those with at least one anchor overlapping a CTCF peak, were predominantly found among the “lost” pixels. In contrast, “retained” and “gained” loop pixels were enriched with “CTCF-EP” or “EP-only” loops, characterized by promoter or enhancer marks at one or both anchors (Fig. 2c, Supplementary Fig. 5b, e, examples of loop classification in Fig. 2d). Notably, in all three CTCF-degron studies, the “gained” loops were large in size with median values between 185 kb and 369 kb (Fig. 2a, Supplementary Fig. 5a, d, right panels), which contrasts with RAD21-degron datasets (Supplementary Fig. 6) and support the hypothesis that newly “gained” E-P loops form through cohesin-mediated loop extrusion (discussed further below).

We also examined the robustness of the “lost”, “retained”, and “gained” pixels across independent Hi-C/micro-C studies. As expected, the “lost” pixels from any study generally also lost strength in the other two studies (Fig. 2e, Supplementary Fig. 5c,f, first row). However, the consistency of “retained” loop pixels was study-specific: (i) although the “retained” loop pixels from Nora et al. were mostly also “retained” in Kubo et al., some of them lost strength upon CTCF depletion in Hsieh et al. (Fig. 2e, second row); (ii) most of the “retained” loop pixels from Kubo et al. lost strength in the other two studies (Supplementary Fig. 5c, second row); and (iii) the “retained” loops from Hsieh et al. remained “retained” in both Nora et al. and Kubo et al. (Supplementary Fig. 5f, second row). Since incomplete CTCF depletion leaves residual CTCF loops^7^, these observations suggest that CTCF depletion in Kubo et al. may have been less complete than in the other two studies. On the other hand, Hsieh et al. appeared to have achieved better CTCF depletion, perhaps due to their use of an N-terminal degron system (Fig. 1a), although platform-specific biases, along with differences in assay sensitivity and sequencing depth, may also explain some of the discrepancies between studies.

Interestingly, the “gained” pixels from any one of the three studies generally also gained strength in the other two studies (Fig. 2e, Supplementary Fig. 5c, f, third row). The only exception was that some “gained” loop pixels from Kubo et al. lose strength in Hsieh et al. (Supplementary Fig. 5c). Again, this is likely because Kubo et al. retained more CTCF loops due to incomplete CTCF degradation. Nevertheless, most of the “gained” pixels from Nora et al. and Hsieh et al. did gain strength in Kubo et al. (Fig. 2e, Supplementary Fig. 5f), supporting the consistency across the three studies. Notably, E-P loop-gaining events were largely overlooked in the original CTCF-degron studies because the changes in loop strength were often too subtle to distinguish from background noise with conventional analytical tools (e.g., Fig. 1d). Our meta-analysis demonstrates that although there are only a small number of newly gained E-P loop pixels in each individual study, they are reliable consequences of CTCF-depletion; affected genes such as *Notch1* (Fig. 1e) are putative target genes of CTCF-mediated insulation.

### Meta-analyses of cohesin regulators reveal both conserved looping dynamics and dataset-specific artifacts

We also performed meta-analyses of the RAD21- and WAPL-depletion Hi-C datasets. Ablation of RAD21 also caused a massive loss of CTCF loops, while the “retained” and “gained” loops were similarly enriched with E-P loops (Supplementary Fig. 6a–i). However, there were important differences between RAD21- and CTCF-depleted cells. (i) In RAD21-depleted cells, the “retained” and “gained” loop pixels (except the “gained” loop pixels in Rhodes et al.) were much smaller (mostly <100 kb) than those in CTCF-depleted cells, indicating that cohesin-independent loops are short-range (Supplementary Fig. 6a, d, g, right panels). An alternative explanation is that the greatly reduced pool of cohesin only allows rare and short-lived extrusion events, resulting in smaller loops. (ii) Unlike in CTCF-depleted cells, the “gained” loop pixels were not recurrent across the three RAD21-degron datasets (Supplementary Fig. 6c, f, i, last row). (iii) The “retained” and “gained” loop pixels in RAD21-depleted cells (except the “gained” loop pixels in Rhodes et al.) were more enriched with the H3K36me3 mark, suggesting transcription-associated chromatin interactions (Supplementary Fig. 6b, e, h).

The “gained” loops in RAD21-depleted cells from Rhodes et al. were enriched with H3K27me3 and tended to be larger in size (Supplementary Fig. 6a, b, right panels). These observations align with the conclusion from the original study that cohesin removal enhances long-range interactions between a subset of polycomb-occupied regions^30^. However, these features (the larger loop size and the enrichment with H3K27me3) were specific to the “gained” loops in Rhodes et al. and were not observed in the other two RAD21-degron studies (Supplementary Fig. 6d, e, g, h, right panels). We manually examined the “gained” H3K27me3 loops in Rhodes et al. and found that in almost all cases, the long-range H3K27me3 loops were already present in the control cells from Kriz et al. and Hsieh et al. before RAD21 depletion (two examples are shown in Supplementary Fig. 7). Therefore, at least in the control mESCs from Kriz et al. and Hsieh et al., cohesin does not prevent the formation of long-range H3K27me3 loops.

In contrast, ablation of the cohesin unloading factor WAPL generated up-skewed scatter plots in all three studies (Supplementary Fig. 6j, m, p, left panels). The skewness in Hsieh et al. was less apparent, likely because Hsieh et al. performed WAPL depletion for a much shorter duration (3 h) than Liu et al. (24 h) and Kriz et al. (72 h) (Fig. 1a). In all WAPL-degron studies, the “lost”, “retained”, and “gained” loops were almost equally enriched with CTCF and E-P loops (Supplementary Fig. 6k, n, q). Consistent with the model that WAPL depletion stabilizes chromatin-bound cohesin and promotes loop extrusion^14,15^, the “gained” loops were significantly larger than the “lost” and “retained” loops in all three studies (Supplementary Fig. 6j, m, p, right panels). The “lost”, “retained”, and “gained” loop pixels were generally consistent across studies (Supplementary Fig. 6l, o, r) despite some discrepancies for the “lost” loop pixels from Hsieh et al. (Supplementary Fig. 6r), again likely because Hsieh et al. only depleted WAPL for a short period of time.

### Newly gained E-P loops in CTCF-depleted cells are cohesin-dependent

We next took the “lost”, “retained”, and “gained” loop pixels from the CTCF-degron studies and examined how they changed in the RAD21- and WAPL-degron data. First, nearly all “lost” loops in CTCF-depleted cells also disappeared in RAD21-depleted cells (Fig. 2d, first row, Supplementary Fig. 8a, b, first row), suggesting that most of the CTCF loops are cohesin-dependent. Second, a subset of the “retained” loops in CTCF-depleted cells were also “retained” upon RAD21 depletion (Fig. 2e, second row, Supplementary Fig. 8a, b, second row), suggesting cohesin-independence. The remaining “retained” loops in CTCF-depleted cells lost strength upon RAD21-depletion, likely because they represent either (i) residual CTCF loops resulting from incomplete CTCF-depletion, or (ii) non-CTCF loops formed via cohesin-dependent mechanisms. Interestingly, most of the “retained” loops in CTCF-depleted cells from Kubo et al. were lost upon RAD21 depletion (Supplementary Fig. 8a, second row), whereas most of the “retained” loops from Hsieh et al. remained “retained” upon RAD21-depletion (Supplementary Fig. 8b, second row). This again suggests that Hsieh et al. achieved more complete CTCF depletion than Kubo et al.

Finally, the “gained” loops in the CTCF-degron studies, which were weak in both CTCF-degron and RAD21-degron control mESCs, did not gain strength upon RAD21 depletion (Fig. 2e, Supplementary Fig. 8a, b, third row), suggesting that the formation of these “gained” loops requires cohesin. In contrast, we observed that these “gained” loops in CTCF-depleted cells showed an apparent upward bias in the scatter plots of WAPL-degron studies (Fig. 2e, Supplementary Fig. 8a, b, third row). This observation is consistent with the example in Fig. 1f and further supports a role for cohesin in the formation of these “gained” loops. WAPL depletion may perturb the function of insulators by releasing short-range CTCF loops, allowing previously insulated E-P interactions to bypass certain insulators.

### Systematic mapping of recurrent loop rewiring events identifies functional insulators (FINs) in the mESC genome

**Fig. 3:**
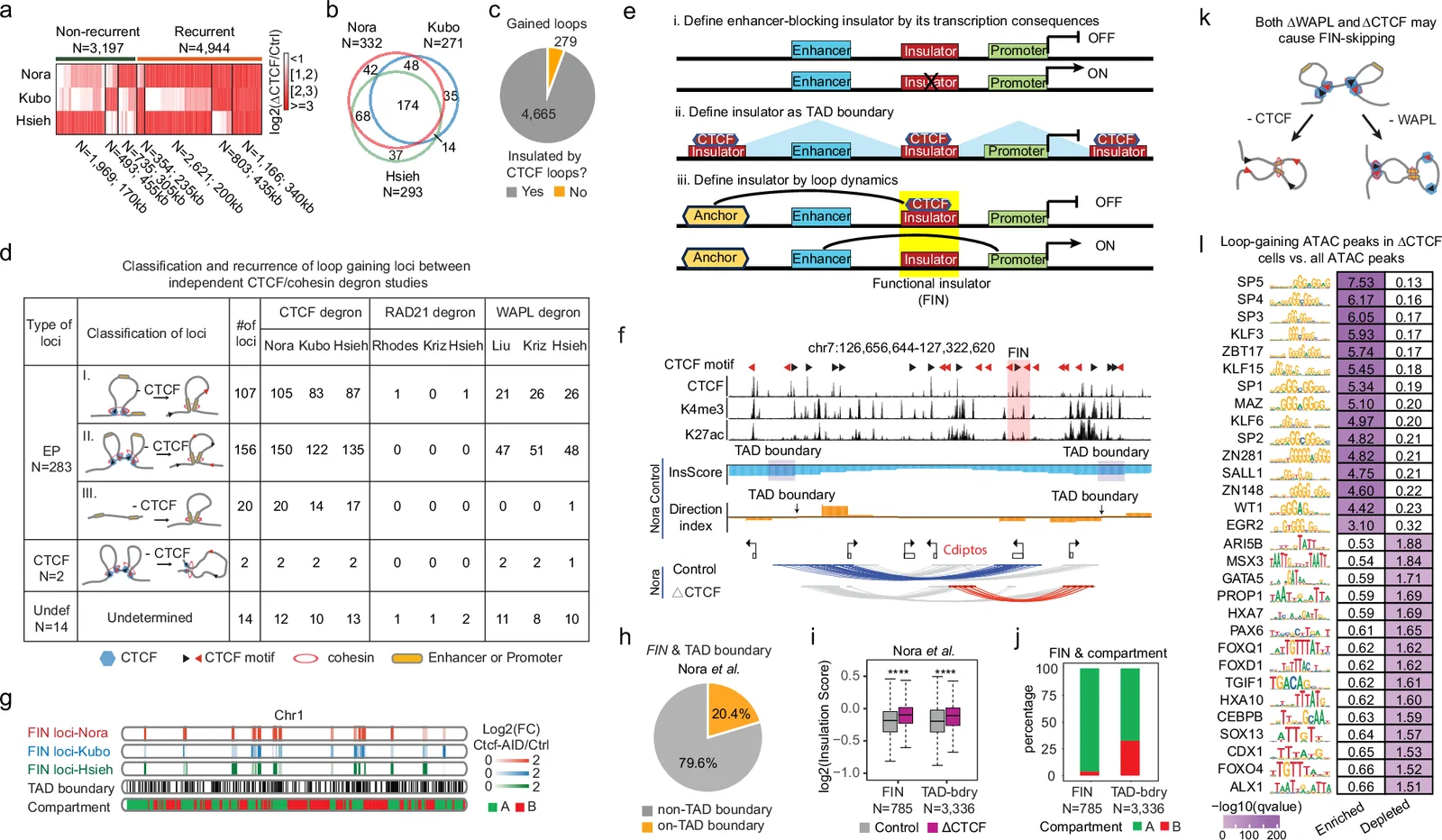
Characterization of functional insulators (FINs) in mESC genome. **a** Heatmap showing the union of all “gained” loop pixels upon CTCF depletion. Each row represents one CTCF-degron study, each column represents one loop pixel, and loop pixels are clustered based on their reproducibility. Numbers of pixels and median sizes of each cluster are also shown. Color scale: ratio increase of loop signal in log-scale. **b** Venn diagram showing the consistency of loop-gaining loci identified from three datasets. **c** Pie chart showing that most of the gained loops in CTCF-depleted cells have at least one CTCF loop anchor in between in wildtype cells. **d** A table summarizing the classification and recurrence of loop gaining loci between independent CTCF- or cohesin- degron studies. **e** Cartoons summarizing three possible ways to define insulators. **f** An example of non-TAD boundary FIN. The FIN is highlighted in pink. The tracks of InsScore and DI are also included. TAD boundaries determined from InsScore and DI are highlighted in purple and indicated with black arrows, respectively. **g** Ideogram showing the locations of loop-gaining loci from three studies (shown by different colors), TAD boundaries, and compartments on one chromosome. Color scale: fold increase of gained loop in log scales. **h** Pie chart showing the percentage of FINs that overlap TAD boundary. **i** Boxplot comparing the insulation scores at FINs and TAD boundaries before and after CTCF depletion (based on Nora et al.). Boxplots show median ± upper and lower quartiles with whiskers showing 5 and 95 percentiles. **** *p* < 0.0001 two-sided Wilcoxon rank-sum test (Exact p values: FINs: 1.45e–6, TADs: 1.19e–23). **j** Stack bar plot comparing the proportion of FINs and TAD boundaries in compartment A or B. **k** A model to explain why some of the newly gained E-P loops in CTCF-depleted cells are also observed in WAPL-depleted cells. **l** A list of TF motifs enriched or depleted in the mESC ATAC-seq peaks that will gain chromatin loops upon CTCF depletion. Enrichment or depletion are relative to all mESC ATAC-seq peaks.

Of the 8,141 “gained” loop pixels from all three CTCF-degron Hi-C/micro-C datasets, 4,944 pixels (Supplementary Data 13) were recurrent in at least two studies, with a median size of ~250 kb (Fig. 3a). In each dataset, the “gained” loop pixels were merged into ~300 “loop-gaining loci” (Methods), and there was substantial overlap between independent studies: 174 loop-gaining loci were shared by all three studies and 304 were shared by at least two studies (Fig. 3b). We hypothesized that these recurrent loop-gaining loci were caused by the loss of CTCF insulation; indeed, 4665 out of the 4944 loop-gaining pixels (94%) were insulated by at least one CTCF loop in wild-type cells (Fig. 3c).

We further curated the reproducible loop-gaining loci based on the types and numbers of *cis*-regulatory elements (CREs) involved (Fig. 3d, Supplementary Data 4). Nearly all the curated loci (283 of 299) gained E-P loops (two loci gained CTCF contacts, while the others could not be clearly classified). We further classified the E-P loop-gaining loci into three categories (Fig. 3d). In *Type I*, one of the two E/P sites was insulated inside one CTCF loop while the other site is not (exemplified in Supplementary Fig. 9a); *Type II* involves at least two CTCF loops insulating two E/P-sites (exemplified in Fig. 1e, Supplementary Fig. 9b). In contrast, the gained E-P loops in *Type III* do not have insulating CTCF loops (exemplified in Supplementary Fig. 9c). *Types I* and *II* represented most of the E-P loop-gaining loci (263 out of 283), while only 20 loci belonged to *Type III*, again suggesting that most loop gaining loci resulted from the removal of CTCF insulation.

Since our results highlighted a group of unique loci that undergo substantial E-P loop rewiring in response to CTCF depletion, we reasoned that it is necessary to revisit the relationship between 3D genome architecture and transcriptional insulators. The conventional model of defining insulators is based on the gain of expression upon the loss of insulator function (Fig. 3e, model *i*), but the current model, which defines insulators as TAD boundaries, is static and does not consider structural outcomes following insulator loss (Fig. 3e, model *ii*). We therefore propose defining “functional insulators” (FINs) as the sequences within “lost” loop anchors that, when bound by CTCF, actively block the interactions between flanking *cis*-regulatory elements (CREs) (Fig. 3e, model *iii*; Methods; Supplementary Data 14). In this model, FINs can be identified from “loop-gaining loci”. Indeed, because most “loop-gaining loci” contain at least one FIN, we hereafter refer to them as “FIN loci” (“Methods”). This definition of FINs is based on the gain of loops upon the loss of insulator function, a dynamic metric that is conceptually analogous to the conventional insulator model but distinct from the static TAD-based model.

### FINs are distinct from static TAD boundaries and reside within active compartments

In Fig. 3f, we show a FIN (highlighted in pink) that is not located at a TAD boundary. In this example, we also included the browser tracks for “insulation score” (InsScore)^34^ and “directionality index” (DI)^1^. TAD boundaries are traditionally defined as bins where InsScore reaches a local minimum or positions where the DI changes its sign (Supplementary Fig. 10a), but the FIN in this example fits neither criterion. There were much fewer FINs than TAD boundaries (Fig. 3g) and only a small percentage of FINs overlapped with TAD boundaries (Fig. 3h, Supplementary Fig. 10b). These observations indicate that most FINs reside within TADs and, conversely, that the loss of TAD boundaries usually does not cause loop formation across their flanking regions.

Furthermore, changes in InsScore did not predict FINs. Although we observed an overall trend of increased InsScore at FINs upon CTCF depletion, TAD boundaries exhibited the same trend and could not be distinguished from FINs (Fig. 3i, Supplementary Fig. 10c). Interestingly, most (96%) FINs were located within compartment A (Fig. 3j), indicating that active E-P blocking mainly occurs within euchromatic regions. Regardless, it is important to note that low-resolution 3D genome features, including compartments and TAD boundaries, lack the precision required to reveal the dynamic changes in loop profiles that we used to define FINs.

Cohesin degron Hi-C data further supported a cohesin-dependent mechanism for FIN function. We found that almost none of the curated loop-gaining events appeared in the RAD21-degron cells (Fig. 3d), indicating cohesin dependency. Interestingly, a significant fraction of the *Type I* and *II* (but not *Type III*) E-P loop-gaining loci were also observed in the WAPL-degron datasets (Fig. 3d). This phenomenon can be explained by previous findings that WAPL depletion allows loop extrusion to bypass CTCF blocks^14,15^. As shown in the schematic in Fig. 3k, because WAPL depletion stabilizes chromatin-bound cohesin and promotes longer loop extrusion, previously insulated enhancers and promoters could interact within the enlarged CTCF loop via “insulator skipping”, creating the same E-P loops observed in CTCF-depleted cells. All these results support FINs as bona fide insulators against cohesin-mediated loop extrusion.

To explore which types of enhancers or promoters gain chromatin loops upon CTCF depletion, we selected the FIN-blocked ATAC peaks in mESCs (n = 995) and scanned for enriched or depleted motifs compared to all mESC ATAC peaks as controls (n = 59,871). Interestingly, the loop-gaining ATAC peaks were enriched for many G-rich motifs but depleted of A/T-rich motifs (Fig. 3l). This result is reminiscent of previous reports that G-quadruplex (G4) structures may regulate chromatin looping^35,36^. Notably, many of the putative transcription factors binding to these G-rich motifs have been reported to regulate loop formation in various cell types, such as Kruppel-like factors^37–39^, SP1^40^, MAZ, and several zinc-finger proteins^41–43^. Therefore, our results suggest that G-rich CREs are more likely to act as new loop anchors upon CTCF depletion.

### Loss of insulation at FINs triggers reproducible and direct transcription activation

**Fig. 4:**
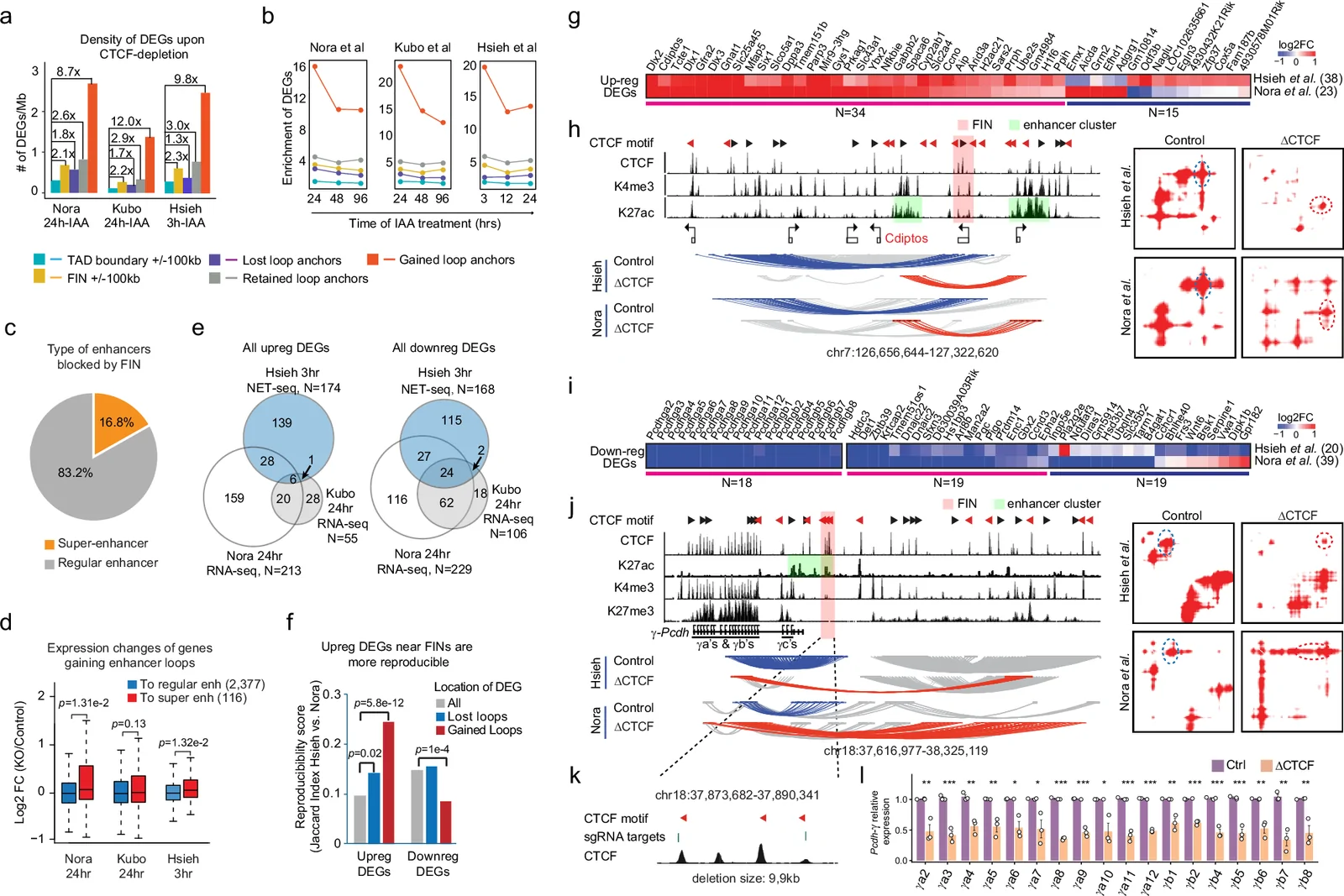
FINs are associated with consistent early transcriptional consequences in independent CTCF-degron studies. **a** Bar plots comparing the density of DEGs upon CTCF-depletion across different genomic regions in three CTCF-degron studies. **b** Enrichment of DEGs at different types of genomic regions compared to whole genome as background. X-axis: time of IAA treatment (hrs); y-axis: fold enrichment. **c** Pie chart showing the percentage of FINs that block super enhancers. **d** Boxplots comparing the expression changes of genes gaining enhancer loops. Red: genes gaining super-enhancer loop; Blue: genes gaining regular enhancer loop. P-values: two-sided Wilcoxon signed-rank test. Boxes: median ± upper and lower quartiles; whiskers: 5 and 95 percentiles. **e** Venn diagrams showing the overlap of up- (left) and down-regulated (right) DEGs between three CTCF-degron studies. **f** Bar plot comparing the reproducibility scores (measured by Jaccard Index) of up- or down-regulated DEGs between two CTCF-degron studies (Hsieh et al. and Nora et al.). *P* values: binomial test. **g** Heatmap showing the union of upregulated DEGs at the anchors of gained loops from Hsieh et al and Nora et al.; DEGs are clustered based on consistency. **h** Left: browser tracks near *Cdiptos* locus; right: *DeepLoop* heatmaps in the same region. The direction of CTCF motifs is indicated by triangles. Blue arches in tracks and blue dashed circles in heatmaps: lost loop pixels; red arches and red dashed circles: gained loop. **i** Heatmap showing the union of down-regulated DEGs at the anchors of gained loops from Hsieh et al. and Nora et al. **j** Similar as **h**, left: browser tracks near *Pcdh*-γ locus; right: Hi-C heatmaps. **k** Design of sgRNAs for insulator deletion. **l** RT-qPCR shows the down-regulation of *Pcdh*-γ genes in mESCs after insulator deletion (data normalized against *Actb* across three biological replicates). Error bars: mean ± SEM. * *p* < 0.05, ** *p* < 0.01, *** *p* < 0.001, two-sided *t* test. (Exact *p* values: γ*a2*: 8.0e–3; γ*a3*: 8.8e–4; γ*a4*: 3.5e–3; γ*a5*: 6.6e–3; γ*a6*: 1.0e–2; γ*a7*: 2.7e–2; γ*a8*: 1.8e–6; γ*a9*: 2.3e–4; γ*a10*: 1.8e–2; γ*a11*: 2.9e–4; γ*a12*: 4.4e–6; γ*b1*: 5.2e–3; γ*b2*: 5.7e–5; γ*b4*: 4.7e–4; γ*b5*: 9.6e–4; γ*b6*: 3.4e–3; γ*b7*: 3.2e–3; γ*b8*: 9.8e–3). Source data provided in the Source data file.

We found that in all CTCF-degron studies, FIN-proximal regions (±100 kb) are 2- to 3-fold more enriched with differentially expressed genes (DEGs) than TAD-boundary-proximal regions, and “gained” loop anchors were 10 times more enriched with DEGs (Fig. 4a). Furthermore, early-response DEGs were clearly more enriched at gained loop anchors (Fig. 4b), suggesting that FIN-blocked enhancers and promoters are direct targets of insulation. In 16.8% of FIN loci (49 out of 299), we observed the blocking of 43 super-enhancers (out of 231 total super-enhancers) (Fig. 4c). As expected, genes gaining super-enhancer interactions were more likely to be upregulated than genes gaining interactions with regular enhancers (Fig. 4d).

Using common cutoffs (twofold for RNA-seq and 1.5-fold for NET-seq, *p* < 0.05), each CTCF-degron study identified several hundred early-response DEGs; however, reproducibility between independent studies is often limited due to technical differences (Fig. 4e). Both Nora et al. and Kubo et al. performed RNA-seq and a 24-h timepoint after CTCF-depletion, which may include late-response DEGs caused by indirect mechanisms (e.g., loss of pluripotency). Among these two studies, Kubo et al. detected fewer DEGs than Nora et al., though 70% of the DEGs from Kubo et al. were also observed by Nora et al., including 47% of the upregulated DEGs and 81% of the downregulated DEGs (Fig. 4e). This observation is consistent with our earlier finding that CTCF depletion in Kubo et al. was less complete. Conversely, Hsieh et al. performed both NET-seq and RNA-seq after only 3-h CTCF-depletion. While this shorter treatment time targets direct responses, it may also reduce sensitivity. We utilize the NET-seq data because Hsieh et al. reported that it provides higher sensitivity in identifying DEGs^25^ by mapping newly synthesized RNA, whereas RNA-seq measures steady-state RNA levels.

We focused our comparison on Hsieh et al. and Nora et al. as they represent a more complete set of putative direct targets. We hypothesized that DEGs near FINs are more likely to be direct targets of CTCF-mediated E-P insulation and therefore should be more reproducible across studies. Indeed, upregulated DEGs near FINs (i.e., at “gained” loop anchors) were more reproducible, whereas downregulated DEGs near FINs were less reproducible than controls (Fig. 4f, red bars). In contrast, “lost” loop anchors did not predict DEG reproducibility. This suggests that transcriptional activation is the primary consequence of insulator loss, consistent with the classical concept that insulators block enhancer-mediated activation.

In Fig. 4g, we plotted 49 upregulated DEGs at FIN loci as putative “direct targets” of insulators (from Hsieh et al. or Nora et al.); of these, 34 are “high-confidence” targets supported by both studies. A representative example is shown in Fig. 4h and Supplementary Fig. 11a, where the gain of loop between two putative enhancer clusters upon CTCF depletion led to the upregulation of the *Cdiptos* gene. Similarly, we identified 37 reproducible downregulated DEGs near FINs (Fig. 4i). Strikingly, 18 of these recurrently down-regulated DEGs belong to the protocadherin-γ (*Pcdhg*) cluster (Fig. 4i,j). Protocadherin proteins play key roles in the formation of synaptic connections between neurons and the *Pcdh-*γ genes are distinguished by their variable promoters (exon1): γ*a*’s and γ*b*’s are stochastically expressed in individual neurons and the γ*c*’s are constitutively expressed^44–47^. In mESCs, the promoters of the γ*a* and γ*b* subfamilies are occupied by CTCF, H3K4me3, and the repressive H3K27me3 mark (Fig. 4j). A cluster of putative enhancers (H3K27ac) is present at the 3′ end of the γ*c* promoters (Fig. 4j, highlighted in green), forming loop interactions with the γ*a* and γ*b* promoters (Fig. 4j, blue arches) that are restricted by a FIN containing multiple CTCF peaks (Fig. 4j, pink). Upon CTCF depletion, these enhancer interactions were weakened, and the *Pcdh-*γ promoter loops extended into a distal, H3K27ac-poor region marked by H3K27me3 (Fig. 4j), consistent with the downregulation of γ*a* and γ*b* genes. To verify the function of the putative FIN, we used a pair of sgRNAs to delete the four CTCF peaks in the FIN and confirmed the down-regulation of all γ*a* and γ*b* genes (Fig. 4k-l). Taken together, these results support the transcription consequences of FINs and nominate specific direct targets of insulator function in mESCs.

### Targeted displacement of CTCF clusters confirms the causal role of FINs in loop rewiring and gene activation

**Fig. 5:**
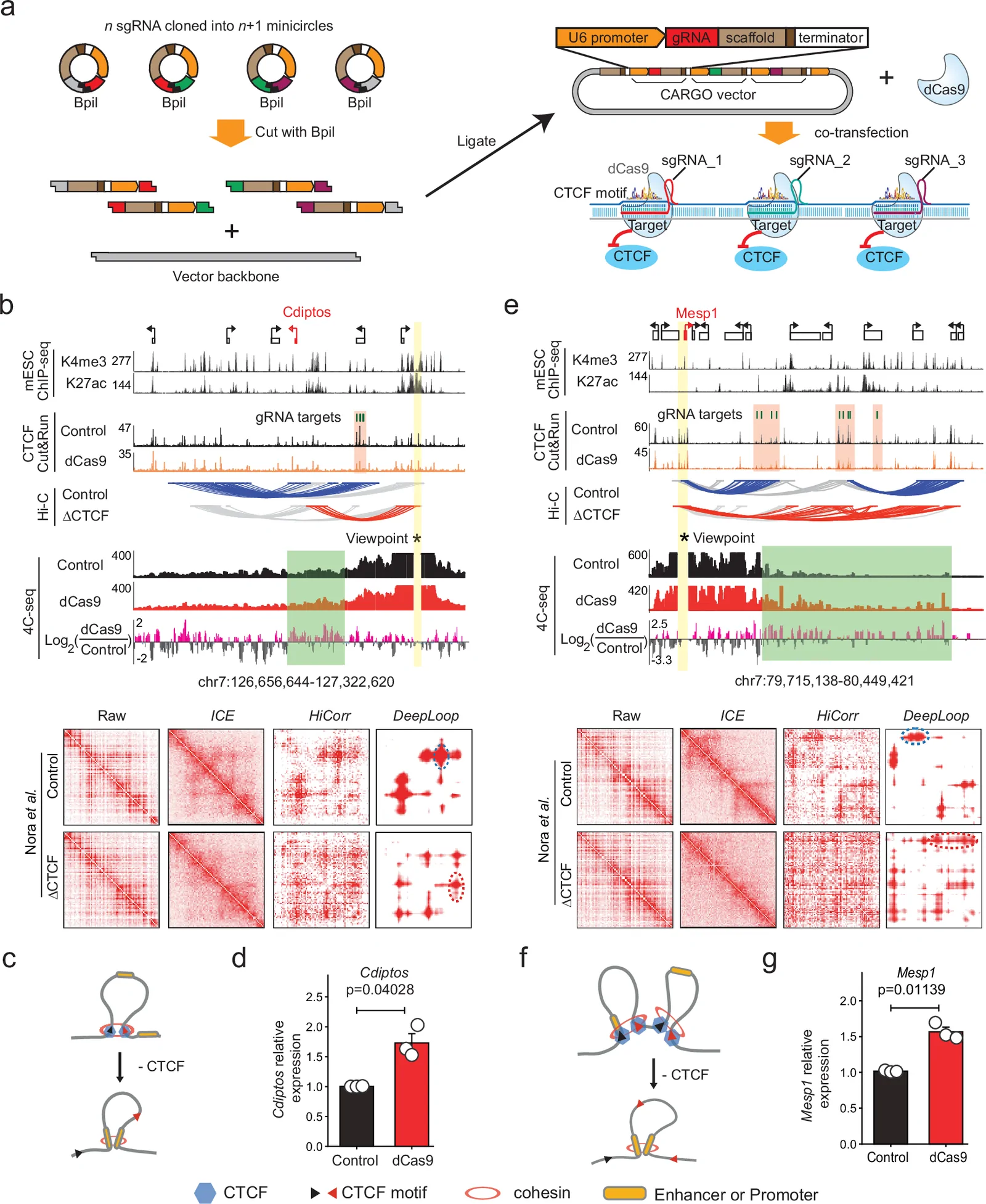
Validate FINs with multiplexed CTCF displacement assay. **a** Scheme of the CARGO vector that can express multiple sgRNAs. In this system, the sequences of *n* sgRNAs are broken into left and right halves and inserted into (*n* + 1) minicircles. The *i*-th minicircle contains the right half of the (*i-1*)-th sgRNA and the left half of the *i*-th sgRNA sequences divided by a *BpiI* restriction site. Final CARGO vector can be assembled by ligating the re-linearized minicircles and vector backbone; each sgRNA is driven by a U6 promoter. Co-transfect CARGO vector and a dCas9-expressing plasmid can displace multiple CTCF binding sites in the same cell. **b** Validation of a FIN at the *Cdiptos* locus. From the top, 1st and 2nd tracks are H3K4me3 and H3K27ac ChIP-seq data showing the promoter and enhancers in mESC. 3rd and 4th tracks show CTCF ChIP-seq data before and after targeting 4 CTCF motifs in FIN (highlighted in pink) with CARGO. 5th and 6th are Hi-C arches (*DeepLoop* pixels with data from Nora et al.) before and after CTCF-depletion (blue: lost loops; red: gained loops). The bottom 3 tracks show 4C-seq data (bait highlighted in yellow) before and after targeting CTCF sites; regions highlighted in green gain 4 C signal. The raw and *ICE*, *HiCorr*, or *DeepLoop* processed contact heatmaps are also included. **c** A model cartoon showing that the gained loop at this FIN locus only involves one CTCF loop. **d** RT-qPCR results showing the upregulation of *Cdiptos* after CTCF displacement. P values are from two-sided paired *t* test (three biological replicates. Each biological replicate is the average of three or four technical replicates). error bars: mean ± SEM. **e–g** Validation of a FIN at *Mesp1* locus similar as shown in (**b–d**). Note the gained loop at this FIN locus involves at least two CTCF loops (**f**). *P* values are from two-sided paired t-test (three biological replicates. Each biological replicate is the average of four technical replicates); error bars: mean ± SEM. Source data are provided for RT-qPCR data.

To validate the *cis*-regulatory functions of FINs, we sought to verify if the gained loops resulted directly from the loss of insulation. Because a FIN often involves multiple CTCF binding sites that contribute collectively to its insulating function, simultaneously perturbing multiple sites across an extended genomic region is necessary. To achieve this, we combined a multi-sgRNA delivery system, CARGO^48^, with a dCas9-based CTCF-displacement method^46,49^ to perturb multiple CTCF binding sites within the same cells. In this approach, four to ten sgRNAs designed to overlap the cognate motifs of multiple CTCF binding sites are expressed from a single CARGO plasmid. When co-transfected with a dCas9 construct, these sgRNAs displace CTCF from the genome at multiple target sites simultaneously (Fig. 5a).

We utilized this approach to validate loop-gaining events at two distinct FIN loci. The first FIN is located near *Cdiptos*, a recurrently upregulated DEG (Fig. 5b). This locus represents a simpler FIN configuration in which the newly gained loop (Fig. 5b, red arches) is blocked by a single FIN (Fig. 5c). Using a CARGO construct targeting four CTCF motifs, we confirmed via CUT&RUN that we successfully weakened the target CTCF peaks without affecting neighboring peaks in the region (Fig. 5b, highlighted in pink). 4C-seq analysis confirmed the gain of long-range chromatin interactions consistent with the Hi-C data (Fig. 5b, highlighted in green). Furthermore, we validated via RT-qPCR that *Cdiptos* was upregulated upon displacement of the target CTCF binding sites (Fig. 5d).

The second example exhibits a more complex FIN configuration involving multiple potential FINs (Fig. 5e, f). At this locus, *Mesp1* lost its loop interaction with a nearby FIN (Fig. 5e, blue arches) and, upon CTCF depletion, gained promiscuous distal interactions across a region of ~200 kb (Fig. 5e, red arches). We used a CARGO construct to target nine CTCF motifs, successfully weakened the target CTCF peaks (Fig. 5e, pink). Using 4C-seq, we confirmed the gain of interactions with the large distal region containing several clusters of putative enhancers (Fig. 5e, green). RT-qPCR confirmed the upregulation of *Mesp1* (Fig. 5g). Taken together, these experiments verify the insulating functions of the FINs identified through our CTCF-degron Hi-C meta-analysis.

## Discussion

In this study, we propose a functional definition for “functional insulators” (FINs) as a specific subset of CTCF binding sites that actively block loop formation between flanking *cis*-regulatory elements (CREs) (Fig. 3e). This definition aligns with the classical concept of insulators as DNA elements that prevent enhancer-mediated gene activation, but it shifts the focus from static domain boundaries to dynamic architectural rewiring. Mapping FINs requires the robust detection of newly gained E-P loops in CTCF-depleted cells, a task that has historically been challenging for standard Hi-C analysis due to the subtle nature of E-P interactions. By performing a meta-analysis of three independent CTCF-degron Hi-C datasets, we demonstrated that the gain-of-loop signals detected by *DeepLoop* are quantitatively robust across different Hi-C and Micro-C platforms. These results validate the feasibility of mapping FINs reliably using Hi-C at intermediate read depths, providing a systematic framework for identifying functional insulation across the genome.

Our findings indicate that the FIN model is fundamentally distinct from the common assumption that TAD boundaries serve as universal insulators. While most TADs disappear upon CTCF depletion, we typically do not observe the gain of new loops across these boundaries, suggesting they do not actively block E-P interactions. In contrast, most FINs function as CTCF loop anchors within TADs in the wild-type state; we define an anchor as a “FIN” only if its removal triggers the formation of new flanking loops (Fig. 3e). Importantly, we identified only a few hundred FINs in mESCs. This relative scarcity helps explain the long-standing paradox of why global CTCF depletion causes only modest transcriptomic changes: the vast majority of CTCF sites do not insulate local E-P interactions. Furthermore, while gained E-P loops do not always result in significant gene activation, likely due to the additive nature of enhancers where individual elements only exert small effect sizes^50,51^, we showed that FINs are significantly better predictors of DEGs, particularly recurrently upregulated genes, than TAD boundaries.

It remains unclear why only a small subset of CTCF loop anchors function as FINs. Why is loop gain observed only at specific loci? Our analysis provides several clues. First, the fact that nearly all FINs reside in Compartment A (euchromatin) suggests a dependency on the surrounding active chromatin, which is enriched with active promoters and enhancers. Because promoters and enhancers co-localize with cohesin and its loading factors and may play key roles in loop initiation or stalling^52^, it is highly likely that FIN function is governed by the context of these surrounding elements. Furthermore, since genome compartmentalization is a highly dynamic feature, we speculate that FINs likely exhibit cell-type- or state-specific activity. Second, our meta-analysis of RAD21- and WAPL-degron datasets demonstrates that newly gained E-P loops in CTCF-depleted cells do not form in cohesin-depleted cells and are likely regulated by cohesin turnover. It is therefore plausible that local cohesin activity is a key determinant of FIN function. Finally, the enrichment of G-rich TF motifs at the anchors of “gained loops” suggests that certain CREs possess an intrinsic predisposition to forming stable long-range interactions, a potential that is normally kept under tight control by insulators. Taken together, these observations suggest context-specific roles for FINs, and it will be compelling to explore how these elements exert their functions in the context of health and disease.

## Methods

### Experiments

#### Cell culture

Mouse embryonic stem cells (E14Tg2a) were cultured in DMEM+Glutamax (ThermoFisher, Cat#10566-016) with the addition of 15% Fetal Bovine Serum (ThermoFisher, Cat# SH30071.03), 550 µM β-mercaptoethanol (ThermoFisher, Cat#21985-023), 1 mM Sodium Pyruvate (ThermoFisher, Cat#11360-070), 1X non-essential amino acids (ThermoFisher, Cat#11140-50), and 10^4^ U of Leukemia inhibitory factor (Millipore, Cat#ESG1107). The cells were maintained at a density of 0.2–1.5 × 10^5^ cells/cm^2^ and were passaged every 24–48 h using TrypLE (ThermoFisher, Cat#12563011) on dishes coated with 0.1% gelatin (Millipore, Cat# ES-006-B) at 37 °C and 5% CO2. The medium was refreshed daily when cells were not being passaged. Regular checks for mycoplasma infection were performed every 3–4 months, with negative results.

#### 4C-seq

4C-seq was conducted following a published protocol^53^. Briefly, 5 million cells fixed with 2% formaldehyde, then quenched with 125 mM glycine. The fixed cells were lysed using a cell lysis buffer containing 50 mM Tris-Cl pH 7.5, 150 mM NaCl, 5 mM EDTA, 0.5% NP-40, 1% Triton X-100, and 1x protease inhibitor cocktail (Roche, Cat#11873580001) for 20–30 min on ice. Following lysis, nuclei were collected by centrifugation at 2500 × *g* for 5 min at 4 °C and washed once with 1x restriction enzyme buffer. The nuclei pellets were resuspended in 1x restriction enzyme buffer and treated with 0.3% SDS for 1 h at 37 °C with shaking, followed by an additional hour with 2.5% Triton X-100. Chromatin was then digested overnight with the appropriate restriction enzyme at the recommended temperature while rotating in an air bath. The specific restriction enzymes used for each locus are detailed in Supplementary Data 6. After digestion, the enzymes were inactivated by heating at 65 °C, and nuclei were ligated with 50 μl of T4 DNA ligase (Invitrogen, Cat# 15224–090) in a 7 ml ligation solution at 16 °C overnight. Reverse cross-linking was performed by treating the samples with proteinase K to isolate the proximity-ligated DNA. The purified DNA was quantified and subjected to secondary restriction enzyme digestion using roughly 1 unit of restriction enzyme per 1 μg of DNA at the recommended temperature overnight. After enzyme inactivation, the samples were self-ligated with T4 DNA ligase. The ligated DNA was recovered using sodium acetate and ethanol and quantified using the Qubit dsDNA HS assay kit (Thermo Fisher, Cat# Q32851). The 4 C templates were amplified using designed primers to generate sequencing libraries. The primer system was modified to be compatible with the Illumina Nextera system, using two sequential PCRs. Locus-specific inverse PCR primers are listed in Supplementary Data 6. For each locus, the 4 C templates were amplified with locus-specific primers using 200 ng of template per reaction, and products from five parallel amplifications were pooled to generate the final 4 C library. A 50 μl PCR product aliquot was purified with homemade Sera-Mag beads. One-fifth of the purified DNA was used for a second PCR with primers N7xx and N5xx, identical to Illumina Nextera sample preparation primers. The final products were purified and sequenced. Reads from the first cutting site were used for data analysis.

#### Plasmid cloning

The dCas9 expression vectors used in this study were created using the Cas9 expression vector backbone from the pX330 plasmid (Addgene; plasmid 42230) through the In-Fusion cloning technique (TaKaRa, Cat# 638948). The dCas9 genes were amplified from the pHAGE EF1α dCas9-KRAB plasmid (Addgene; plasmid 50919) via PCR and independently cloned into the AgeI (NEB, Cat# R3552L) and EcoRI (NEB, Cat# R3101T) sites of the pX330 plasmid, substituting the Cas9 ORF. Detailed information regarding the primers can be found in Supplementary Data 5. All sgRNAs in this study were designed using the CCTop-CRISPR/Cas9 target online predictor.

The Cas9 and *Pcdhg* loop anchor target gRNA expression vector (pDonor_Rosa26_Cas9) was constructed using the backbone of the plasmid pDonor MCS Rosa26 (Addgene, plasmid #37200). An EF-1α-Cas9-P2A fragment, amplified from LentiV_Cas9_Puro (Addgene, plasmid #108100), and a Puromycin-SV40 poly(A) signal fragment, derived from pR26 (Addgene, plasmid #127372), were inserted into the backbone. A paired *Pcdhg* loop anchor target gRNA expression cassette (sequences listed in Supplementary Data 5) was then introduced into the construct.

#### CARGO assembly

The CARGO array assembly follows a published protocol^48^. The protocol consists of two main steps: constant region preparation and array assembly. In the constant region preparation step, the constant region with proper sticky ends is released by digesting the pGEMT-hU6-spSL (provided by Dr. Joanna Wysocka) plasmid with BsmBI (NEB, Cat# R0580L). The released constant region is then separated from the pGEMT backbone on a 1% agarose gel and gel purified using a commercial kit (QIAGEN, Cat#28704). Oligonucleotides (50–60 bp) with variable regions were ordered through IDT (Supplementary Data 5). The forward and reverse oligos are annealed and phosphorylated and then stored at −20 °C.

In the array assembly step, the annealed chimeric oligo is mixed with the digested constant region, and a minimum of 4-h ligation with high-concentration T4 ligase (Thermofisher Scientific, Cat#EL0013) is performed. The ligation step yields ligation products, referred to as minicircles. After the stoichiometry check, individual minicircles are treated with plasmid-safe exonuclease (Epicenter, Cat# E3101K) to clean up the un-ligated products. Then, the plasmid-safe exonuclease treated individual minicircles are pooled together and purified. The pooled circle mixes are assembled directly into the digested destination vector pX332-mCherry (provided by Dr. Joanna Wysocka) through an optimized T7 DNA ligation reaction (NEB, Cat#M0318S). The product of the ligation reaction is then subjected to plasmid-safe exonuclease (Epicenter, Cat#E3101K) treatment. After that, the exonuclease-treated reaction is then transformed into competent cells (Bioline, Cat#BIO-85027). For each plate, individual colonies are picked for plasmid purification using a miniprep kit (QIAGEN, Cat#27204) and test digestion with Acc65I (NEB,Cat#R0599S), KpnI (NEB, Cat#R3142S), and XbaI (NEB, Cat#R0145S). Plasmids with the correct digestion pattern are then verified by Sanger sequencing.

#### ChIPmentation

We employed ChIPmentation^54^ to map CTCF in various samples. Briefly, cells and tissues were fixed in 1% formaldehyde at room temperature for 15 min, followed by glycine quenching. To isolate nuclei, we lysed cell with lysis buffer 1 (50 mM HEPES; pH 7.5, 140 mM NaCl, 1 mM EDTA, 10% glycerol, 0.5% Nonidet-40, 0.25% Triton X-100, and a protease inhibitor cocktail) for 10 min at 4 °C. The nuclei were pelleted at 1800 g for 10 min at 4 °C and then resuspended in nuclei lysis buffer (10 mM Tris-Cl pH 8.0, 100 mM NaCl, 1 mM EDTA, 0.5 mM EGTA, 0.1% Na-Deoxycholate, 0.5% N-lauroylsarcosine, and a protease inhibitor cocktail) for sonication. For CTCF pulldown, 100-150 µg of chromatin was used. Dynabeads M-280 (Life Technologies, Sheep Anti-Rabbit IgG, #11204D) were washed three times with 0.5 mg/mL of BSA/PBS on ice, then incubated with the CTCF antibody (Abcam, #ab70303) for at least 2 h at 4 °C. The beads/antibody complexes were then washed with BSA/PBS. Pulldown was performed in binding buffer (1% Triton-X 100, 0.1% Sodium Deoxycholate, and a protease inhibitor cocktail in 1X TE) by mixing the beads/antibody complexes with chromatin. After overnight incubation, the beads/antibody/chromatin complexes were washed with RIPA buffer (50 mM HEPES pH 8.0, 1% NP-40, 0.7% Sodium Deoxycholate, 0.5 M LiCl, 1 mM EDTA, and a protease inhibitor cocktail). The beads complexes were then incubated with homemade Tn5 transposase in tagmentation reaction buffer (10 mM Tris-Cl pH 8.0 and 5 mM MgCl2) for 10 min at 37 °C. To remove free DNA, the beads were washed twice with 1X TE on ice. The pulldown DNA was recovered by reversing crosslinking overnight, followed by purification with PCR Clean DX beads (Aline Biosciences, #C-1003-50). ChIP-seq libraries were generated by amplifying the pulldown DNA with Illumina Nextera primers. Size selection was then performed with PCR Clean DX beads to select fragments ranging from 100 bp to 1000 bp.

#### Pcdhg loop anchor deletion

Mouse embryonic stem cells (E14Tg2a) were transfected with the plasmid pX330-sgR26 (Addgene, plasmid #127376), which expresses mammalian codon-optimized Cas9 and the Rosa26 sgRNA, together with the pDonor_Rosa26_Cas9 plasmid, using Lipofectamine™ 3000 reagent (Invitrogen, Cat# L3000015) according to the manufacturer’s instructions. Two days after transfection, the cells were treated with puromycin (Sigma-Aldrich Cat#P8833) (2 µg/mL) for three days. Drug-resistant clones were then dissociated into single-cell suspensions, mixed thoroughly, and replated at low density to allow single-colony formation, while maintaining puromycin selection (2 µg/mL). After four days, individual colonies were picked for genotyping using primers specific for the *Pcdhg* loop anchor deletion (Supplementary Data 5). Knockout cell clones were identified and selected for subsequent experiments.

### Data analysis

#### Hi-C, micro-C, and 4C-seq

Hi-C and micro-C sequencing reads were mapped to mm10 reference genome separately using bowtie^55^ (v.1.1.2) as previously described^28^. After removing non-unique reads and PCR duplication reads, the bam files are used as input into *HiCorr* in ‘*Bam-process-HindIII*’, ‘*Bam-process-DpnII*’, and ‘*Bam-process-microC*’ modes for bias correction with conventional Hi-C, in-situ Hi-C, and micro-C, respectively. After bias correction, we enhance the Hi-C/micro-C data with *DeepLoop*. The *DeepLoop* package provides multiple models optimized for Hi-C data with different read depths^28^. (Note that we use the number of *cis*-contact within 2Mbp as the measurement of read depth.) To choose the appropriate model, we usually selected the model with the closest read depth. The models chosen for each dataset are listed in Supplementary Data 2. Because each dataset has a pair of Hi-C experiments (control and KO conditions), if the read depths of the two Hi-C experiments are significantly different, we will down-sample the high-depth dataset to lower depth to reduce potential biases (Supplementary Data 2).

4C-seq data were analyzed using pipe4C^53^ (v.1.1.3) to generate bam and bigwig files for data visualization on UCSC genome browser.

#### ChIP–seq and CUT&RUN

ChIP-seq and CUT&RUN data were mapped to mm10 (mouse) using bowtie. After mapping step, we remove PCR duplication reads and use Macs2^56^ (v.2.2.7.1) to call peak and generated bigwig to for data visualization on UCSC genome browser.

#### Motif analysis

Motif enrichment analysis was performed by comparing motif occurrences at ATAC peaks located on gained loop anchors against all ATAC peaks. Background motif frequencies were obtained by scanning all ATAC peak sequences using the mouse HOCOMOCO v11^57^ motif database with FIMO (v.4.11.2, MEME suite)^58^. Motif occurrences in ATAC peaks on gained loop anchors were similarly identified by FIMO scanning. Odds ratios were calculated using Fisher’s exact test to quantify relative motif enrichment. To determine whether motifs were significantly enriched or depleted, a binomial test was applied based on the observed verse expected motif frequencies, and the resulting *p*-values were adjusted to q-values for motif testing correction.

#### RNA-seq and mNET-seq

RNA-seq data are aligned to mm10 using HISAT2 (v.2.1.0)^59^ and read counts for each gene are called with featureCounts (v1.6.1)^60^. DESeq2 (v.1.26.0)^61^ is used to identify differential expressed genes (DEGs). The DEGs are identified using Wald test with the *p* value < 0.01 and log2FC > 1. For mNET-seq analysis, we followed the customized pipeline from the Hsieh et al.^25^. RNA-seq data are aligned to mm10 using HISAT2^59^ and read counts for each gene are called with featureCounts^60^. DESeq2^61^ is used to identify differential expressed genes (DEGs). The DEGs are identified using Wald test with the p-value < 0.01 and log2FC > 1. For NET-seq analysis, we followed the customized pipeline from Hsieh et al. with the following steps: (i) Trim the adapter using TrimGalore (v.0.6.7) (https://github.com/FelixKrueger/TrimGalore) to remove adapter sequences ‘AGATCGGAAGAGCACACGTCTGAACTCCAGTCAC’ and ‘GATCGTCGGACTGTAGAACTCTGAAC’ at each side of the reads; (ii) Map trimmed read to mm10 using STAR; (iii) Identify the last nucleotide incorporated by Pol II by using mNET_snr (https://github.com/tomasgomes/mNET_snr) to locate the 3′-nucleotide of the second read and the strand sign of the first read. (iv) Annotate and count the reads of each gene using FeatureCounts^60^; (v) Use DEseq2^61^ to quantify the changes of the mNET-seq signal at the gene body between control and KO cells.

#### Pairwise loop strength comparison

For each pair of CTCF/cohesin-degron study, we took top 500k loop pixels from the *DeepLoop* enhanced contact matrices of control and IAA/dTAG-treated samples separately, then union them together. The loop strength of each pixel in the two experiments are normalized linearly:

$${Y}_{i}\, \sim {k * X}_{i}+\varepsilon$$

$$i:A\,{given}\,{loop}\,{pixel}$$

$${X}_{i}:i\,{loop}\,{strength}\,{in}\,{WT}\,{condition}$$

$${Y}_{i}:i\,{loop}\,{strength}\,{in}\,{KO}\,{condition}$$

However, because CTCF- and RAD21- depletion leads to massive loss of loop signal, much fewer loop pixels are expected in the IAA/dTAG-treated cells than in control cells. To adjust for this, we have to manually pick a regression slope *k* based on the shape of the scatter plots.

The slopes we chose are listed in Supplementary Data 2. We then use the minimum strength of the control cell loop pixels to adjust the minimum cutoff to call loop pixels in KO samples: ${Y}_{\min }={k*\min ({X}_{i})}$. In another word, a pixel is called as loop pixel in KO sample only if its strength is bigger than ${Y}_{\min }$. This step leads to much fewer loop pixels in CTCF- and RAD21- depleted cells. We typically obtain ~600k chromatin loop for each study. We used a simple fold change cutoff (FC > 4) to define gained or lost loop pixels. Finally, we used the following criteria to call retained loops: (i) the strength of the pixel in both WT and KO conditions must be higher than their minimum cutoffs (${X}_{i} > {X}_{\min }\&{Y}_{i} > {Y}_{\min }$); (ii) the absolute value of residual must be higher than the adjusted minimum of loop pixels in KO samples ($\left|\varepsilon \right| < {Y}_{\min }$).

#### Classify chromatin loops based on epigenome data

For the purpose of being consistent, we intersected loop anchors with the same set of CTCF, H3K27ac, H3K4me3, H3K27me3 and H3K36me3 ChIP-seq peaks in wild type mESCs from ENCODE^62^ to classify the loop pixels. We classification loop pixels in the following order:

1. EP-only: both anchors have either H3K4me3 or H3K27ac mark, but they do not have CTCF peaks.
2. CTCF-EP: after excluding EP-only, look for pixels which only one anchor has CTCF, and the other anchor has either H3K4me3 or H3K27ac marker.
3. CTCF-only: after excluding EP-only and CTCF-EP, look for pixels with at least one anchor bound by CTCF.
4. H3K27me3: after excluding (1)–(3) above, look for pixels with at least one anchor bound by H3K27me3.
5. H3K36me3: after excluding (1)–(4) above, look for pixels with at least one anchor bound by H3K36me3.
6. The rest pixels are classified as “other”.

Notably, this classification is performed with ChIP-seq data from wild-type mESC, including the CTCF-peaks. Presumably, no CTCF-peaks will be present if CTCF-depletion is complete. If we use ChIP-seq data from CTCF-depleted cells for the loop classification, there will be no “CTCF-only” and “CTCF-EP” loops, making the pie-chart comparison in Fig. 2b and Supplementary Fig. 5b, e meaningless. Furthermore, it remains possible that some of “retained” or “gained” loops forms at residual CTCF peaks from incomplete CTCF-depletion. Therefore, it is important to use the same set of ChIP-seq peaks, especially the CTCF peaks in wild type cells, for loop classification to make fair comparisons.

#### Define Loop-gaining loci, FINs, FIN loci, and FIN-blocked regular enhancers and super enhancers (SE)

Loop-gaining loci are defined from “gained” loop pixels in CTCF-depleted cells. We identified thousands of “gained” loop pixels and each pixel represents a pair of 5kb-bins. However, these pixels often occupy overlapping regions. We therefore merge all overlapping pixels into one region and define them as loop-gaining loci. This step allows us to count the number of regions in the genome containing “gained” loops. In our study, we have ~300 loop-gaining loci after merging ~5000 recurrent “gained” loop pixels.

We define FINs as a subset of CTCF binding sites that satisfy the following criteria: (i) they located at the anchors of CTCF loops that are lost upon CTCF depletion; (ii) the FIN should be inside a newly gained loop in CTCF-depleted cells; (iii) the other anchor of the CTCF loop should be outside of the newly gained loop; (iv) CTCF sites satisfying (i-iii) within 30 kb of each other will be merged into one FIN. Note that we do not have limits on the number of CTCF sites to define FINs. Therefore, although a FIN can be just one single CTCF site, most FINs have multiple CTCF sites.

As described above, all FINs must be inside loop-gaining loci. “FIN loci” are “loop-gaining loci” that containing FINs. In fact, almost all the “loop-gaining loci” are also “FIN loci”. We therefore often use the two terms interchangeably.

We call enhancers as H3K27ac peaks and use a published list of super enhancers in mESCs^63^ (Supplementary Data 10 & 11). This allows us to determine the types of enhancers blocked by FINs.

#### Recurrent loop-gaining pixels and loop-gaining loci in CTCF depleted cells

A recurrent loop-gaining pixel is called if it satisfies FC > 4 in at least two studies. We define the location of each loop pixel as the entire region between the left and right anchors (including the anchors themselves). To define loop-gaining loci, we merge all gained loop pixels from the CTCF-degron studies using the “merge” option in *bedtools* (v.2.25.0); each loop-gaining locus usually has multiple loop pixels. Loop loci with gap size <20 kb will be merge into one locus. In this study, we merged all the recurrent loop-gaining pixels to obtain 299 recurrent loop events. To test the consistency of loop-gaining loci between independent CTCF-degron study, we call loop-gaining loci for each individual CTCF-degron study and the share loop-gaining loci from two different studies are called if they have at least one base overlap.

#### Test the recurrence of loop-gaining loci from CTCF-degron in RAD21- and WAPL- degron data

Nearly all loop-gaining loci observed in Hi-C degron studies have at least one E-P loop pixels. We further classify the loop-gaining loci depending on the number of CTCF loops separating the two anchors of the gained E-P loops. For all loci, we also manually examined all loci to confirm the classification. Several loci cannot be clearly classified into the categories shown in Fig. 3d because they have different marks or have multiple events. To test if a loop gaining locus is recurrent in one of the CTCF/cohesin-degron study in Fig. 3d, we examine all loop-gaining pixels in the locus and if one of pixels also gain signal it will be considered recurrent.

#### Compartment and TADs in mESCs

Hi-C compartment analysis is performed in WT mESCs following previous publication^64^. After QC step, we constructed 500 kb resolution contact matrices for each chromosome and then normalized them using the genome distance. We next generated correlation matrices for each chromosome and performed the principal component analysis on the correlation matrix and then assigned the genome into two compartments by using PC1 (Supplementary Data 12). We used the density of transcription starting sites (TSS) of each 500-kb bin to determine compartment A and compartment B.

#### TAD boundary calling with Insulation score or Directionality index (DI) methods

Insulation scores were computed for contact matrices using 40 kb bins and 2 Mb sliding window using the *cooltools* (v.0.7.1)^65^ package. Insulating loci were identified through a local minima detection procedure based on peak prominence. Highly insulating regions corresponding to strong TAD boundaries were defined using the Li thresholding method implemented in the *cooltools* insulation score module. In this method, TAD boundaries are a list of 40 kb bins.

We also called TAD domains with control mESC Hi-C data using the DI method described by Dixon et al.^1^, which quantifies the degree of upstream or downstream interaction bias for each genome bin. Directionality indices were calculated also with 40 kb bins using a 2 Mb sliding window. A hidden Markov model (HMM) was applied to infer hidden bias states across the genome, classifying each bin as upstream-biased, downstream-biased, or neutral. Consecutive bins sharing the same bias state were merged into domains. The start and end positions of each domain are defined as TAD boundaries. In this way, the TAD boundaries will be also a list of single bases. The TADs from the WT samples of the three datasets are listed in Supplementary Data 9.

To compare the TAD boundaries from two methods, we consider two TAD boundaries overlapping if their distance gap is smaller than 40 kb. The same distance cutoff (40 kb) is also used to decide if a FIN overlaps TAD boundary.

#### Ideogram plot

We generate ideogram plots with Rideogram (v.0.2.2)^66^ to visualized the locations of recurrent gaining-loop pixels, compartment, and TADs.

#### Density and enrichment of DEGs upon CTCF-depletion

For any region in the genome, the density of DEGs is defined as the average number of DEGs per Mb-region. We computed DEG density for TAD boundary ±100 kb, FIN ±  100 kb, gained loop anchors, lost loop anchors, and retained loop anchors. We also compute the enrichment of DEGs at regions of interest as their DEG density divided by the DEG density in the whole genome.

#### Hi-C data visualization

Heatmap was used to visualize the raw, ratio, and *DeepLoop*-enhanced heatmaps. We use the following strategies as described before^28^ to determine the color scales these heatmaps. (i) Raw or *ICE*-normalized heatmaps represent raw read counts; the brightest red color indicates the 98th percentile of the contact matrix. Color is proportionally scaled down to one read (white). (ii) HiCorr heatmap represents the ratio value after Hi-C bias correction; the brightest red color indicates at least twofold enrichment of a given pixel comparing to its background. Color is proportionally scaled down to onefold (no enrichment). (iii) DeepLoop heatmaps outputs ‘transformed fold change’ that represents only relative levels of signal enrichment (for example, a value of one may no longer represents no enrichment). We therefore set the brightest red color as the lower limit of the top 300k pixels genome wide. Color is proportionally scaled down to half of that lower limit or onefold, whichever is higher. All loop arches shown in figures were generated with UCSC Genome Browser^67^.

### Reporting summary

Further information on research design is available in the Nature Portfolio Reporting Summary linked to this article.

## Supplementary information

Supplementary Information

Description of Additional Supplementary Files

Supplementary Data 1-14

Reporting Summary

Transparent Peer Review file

## Source data

Source Data

## Data Availability

The raw 4C-seq and ChIP-seq data generated in this study, together with re-analysed published datasets, have been deposited in GEO under accession code GSE243728. Accession numbers for third-party data used in this study are listed in Supplementary Data 1. The 18 Hi-C and micro-C datasets analyzed by *DeepLoop* can be found at https://hiview.case.edu/public/DeepLoop/. Source data are provided with this paper.

## Code availability

The code is available is available at github (https://github.com/JinLabBioinfo/FIN_project). A specific version for this publication is archived on Zenodo^68^ (10.5281/zenodo.19699815).

## References

[1] Dixon, J. R. et al. Topological domains in mammalian genomes identified by analysis of chromatin interactions. *Nature* **485**, 376–380 (2012). 22495300 10.1038/nature11082 PMC3356448

[2] Nora, E. P. et al. Spatial partitioning of the regulatory landscape of the X-inactivation centre. *Nature* **485**, 381–385 (2012). 22495304 10.1038/nature11049 PMC3555144

[3] Bickmore, W. A. & van Steensel, B. Genome architecture: domain organization of interphase chromosomes. *Cell* **152**, 1270–1284 (2013). 23498936 10.1016/j.cell.2013.02.001

[4] Dekker, J. & Mirny, L. The 3D Genome as Moderator of Chromosomal Communication. *Cell* **164**, 1110–1121 (2016). 26967279 10.1016/j.cell.2016.02.007 PMC4788811

[5] McCord, R. P., Kaplan, N. & Giorgetti, L. Chromosome Conformation Capture and Beyond: Toward an Integrative View of Chromosome Structure and Function. *Mol. Cell* **77**, 688–708 (2020). 32001106 10.1016/j.molcel.2019.12.021 PMC7134573

[6] Merkenschlager, M. & Nora, E. P. C. T. C. F. and Cohesin in Genome Folding and Transcriptional Gene Regulation. *Annu Rev. Genomics Hum. Genet* **17**, 17–43 (2016). 27089971 10.1146/annurev-genom-083115-022339

[7] Nora, E. P. et al. Targeted degradation of CTCF decouples local insulation of chromosome domains from genomic compartmentalization. *Cell* **169**, 930–944.e922 (2017). 28525758 10.1016/j.cell.2017.05.004 PMC5538188

[8] Rao, S. S. P. et al. Cohesin loss eliminates all loop domains. *Cell* **171**, 305–320.e324 (2017). 28985562 10.1016/j.cell.2017.09.026 PMC5846482

[9] Nasmyth, K. & Haering, C. H. Cohesin: its roles and mechanisms. *Annu Rev. Genet* **43**, 525–558 (2009). 19886810 10.1146/annurev-genet-102108-134233

[10] Chan, K. L. et al. exit gate is distinct from its entrance gate and is regulated by acetylation. *Cell* **150**, 961–974 (2012). 22901742 10.1016/j.cell.2012.07.028 PMC3485559

[11] Huis in ‘t Veld, P. J. et al. Characterization of a DNA exit gate in the human cohesin ring. *Science* **346**, 968–972 (2014). 10.1126/science.1256904 25414306

[12] Kueng, S. et al. Wapl controls the dynamic association of cohesin with chromatin. *Cell* **127**, 955–967 (2006). 17113138 10.1016/j.cell.2006.09.040

[13] Schwarzer, W. et al. Two independent modes of chromatin organization revealed by cohesin removal. *Nature* **551**, 51–56 (2017). 29094699 10.1038/nature24281 PMC5687303

[14] Haarhuis, J. H. I. et al. The cohesin release factor WAPL restricts chromatin loop extension. *Cell* **169**, 693–707 e614 (2017). 28475897 10.1016/j.cell.2017.04.013 PMC5422210

[15] Wutz, G. et al. Topologically associating domains and chromatin loops depend on cohesin and are regulated by CTCF, WAPL, and PDS5 proteins. *EMBO J.* **36**, 3573–3599 (2017). 29217591 10.15252/embj.201798004 PMC5730888

[16] Wallace, J. A. & Felsenfeld, G. We gather together: Insulators and genome organization. *Curr. Opin. Genet Dev.* **17**, 400–407 (2007). 17913488 10.1016/j.gde.2007.08.005 PMC2215060

[17] Wendt, K. S. et al. Cohesin mediates transcriptional insulation by CCCTC-binding factor. *Nature* **451**, 796–801 (2008). 18235444 10.1038/nature06634

[18] Parelho, V. et al. Cohesins functionally associate with CTCF on mammalian chromosome arms. *Cell* **132**, 422–433 (2008). 18237772 10.1016/j.cell.2008.01.011

[19] Franke, M. et al. Formation of new chromatin domains determines pathogenicity of genomic duplications. *Nature* **538**, 265–269 (2016). 27706140 10.1038/nature19800

[20] Weischenfeldt, J. et al. Pan-cancer analysis of somatic copy-number alterations implicates IRS4 and IGF2 in enhancer hijacking. *Nat. Genet.* **49**, 65–74 (2017). 27869826 10.1038/ng.3722 PMC5791882

[21] Lupianez, D. G. et al. Disruptions of topological chromatin domains cause pathogenic rewiring of gene-enhancer interactions. *Cell* **161**, 1012–1025 (2015). 25959774 10.1016/j.cell.2015.04.004 PMC4791538

[22] Anania, C. et al. In vivo dissection of a clustered-CTCF domain boundary reveals developmental principles of regulatory insulation. *Nat. Genet.* **54**, 1026–1036 (2022). 35817979 10.1038/s41588-022-01117-9 PMC9279147

[23] Zuin, J. et al. Nonlinear control of transcription through enhancer-promoter interactions. *Nature* **604**, 571–577 (2022). 35418676 10.1038/s41586-022-04570-y PMC9021019

[24] Kubo, N. et al. Promoter-proximal CTCF binding promotes distal enhancer-dependent gene activation. *Nat. Struct. Mol. Biol.* **28**, 152–161 (2021). 33398174 10.1038/s41594-020-00539-5 PMC7913465

[25] Hsieh, T. S. et al. Enhancer-promoter interactions and transcription are largely maintained upon acute loss of CTCF, cohesin, WAPL or YY1. *Nat. Genet.* **54**, 1919–1932 (2022). 36471071 10.1038/s41588-022-01223-8 PMC9729117

[26] Akgol Oksuz B. et al. Systematic evaluation of chromosome conformation capture assays. *Nat. Methods* **18**, 1046–1055 (2021). 10.1038/s41592-021-01248-7 PMC8446342 34480151

[27] Lu, L. et al. Robust Hi-C maps of enhancer-promoter interactions reveal the function of non-coding genome in neural development and diseases. *Mol. Cell* **79**, 521–534.e515 (2020). 32592681 10.1016/j.molcel.2020.06.007 PMC7415676

[28] Zhang, S. et al. DeepLoop robustly maps chromatin interactions from sparse allele-resolved or single-cell Hi-C data at kilobase resolution. *Nat. Genet.* **54**, 1013–1025 (2022). 35817982 10.1038/s41588-022-01116-w PMC10082397

[29] Kriz, A. J., Colognori, D., Sunwoo, H., Nabet, B. & Lee, J. T. Balancing cohesin eviction and retention prevents aberrant chromosomal interactions, Polycomb-mediated repression, and X-inactivation. *Mol. Cell* **81**, 1970–1987.e1979 (2021). 33725485 10.1016/j.molcel.2021.02.031 PMC8106664

[30] Rhodes, J. D. P. et al. Cohesin Disrupts Polycomb-Dependent Chromosome Interactions in Embryonic Stem Cells. *Cell Rep.* **30**, 820–835.e810 (2020). 31968256 10.1016/j.celrep.2019.12.057 PMC6988126

[31] Liu, N. Q. et al. WAPL maintains a cohesin loading cycle to preserve cell-type-specific distal gene regulation. *Nat. Genet.* **53**, 100–109 (2021). 33318687 10.1038/s41588-020-00744-4 PMC7610352

[32] Imakaev, M. et al. Iterative correction of Hi-C data reveals hallmarks of chromosome organization. *Nat. Methods* **9**, 999–1003 (2012). 22941365 10.1038/nmeth.2148 PMC3816492

[33] Yaffe, E. & Tanay, A. Probabilistic modeling of Hi-C contact maps eliminates systematic biases to characterize global chromosomal architecture. *Nat. Genet.* **43**, 1059–1065 (2011). 22001755 10.1038/ng.947

[34] Crane, E. et al. Condensin-driven remodelling of X chromosome topology during dosage compensation. *Nature* **523**, 240–244 (2015). 26030525 10.1038/nature14450 PMC4498965

[35] Li, L. et al. YY1 interacts with guanine quadruplexes to regulate DNA looping and gene expression. *Nat. Chem. Biol.* **17**, 161–168 (2021). 33199912 10.1038/s41589-020-00695-1 PMC7854983

[36] Wulfridge, P. et al. G-quadruplexes associated with R-loops promote CTCF binding. *Mol. Cell* **83**, 3064–3079.e3065 (2023). 37552993 10.1016/j.molcel.2023.07.009 PMC10529333

[37] Schoenfelder, S. et al. Preferential associations between co-regulated genes reveal a transcriptional interactome in erythroid cells. *Nat. Genet.* **42**, 53–61 (2010). 20010836 10.1038/ng.496 PMC3237402

[38] Di Giammartino, D. C. et al. KLF4 is involved in the organization and regulation of pluripotency-associated three-dimensional enhancer networks. *Nat. Cell Biol.* **21**, 1179–1190 (2019). 31548608 10.1038/s41556-019-0390-6 PMC7339746

[39] Wei, Z. et al. Klf4 organizes long-range chromosomal interactions with the oct4 locus in reprogramming and pluripotency. *Cell Stem Cell* **13**, 36–47 (2013). 23747203 10.1016/j.stem.2013.05.010

[40] Bryan, A. F., Justice, M., Stutzman, A. V., McKay, D. J. & Dowen, J. M. Cohesin stabilization at promoters and enhancers by common transcription factors and chromatin regulators. *Epigenetics Chromatin* **18**, 33 (2025). 40490791 10.1186/s13072-025-00598-2 PMC12147376

[41] Ortabozkoyun, H. et al. CRISPR and biochemical screens identify MAZ as a cofactor in CTCF-mediated insulation at Hox clusters. *Nat. Genet.* **54**, 202–212 (2022). 35145304 10.1038/s41588-021-01008-5 PMC8837555

[42] Xiao, T., Li, X. & Felsenfeld, G. The Myc-associated zinc finger protein (MAZ) works together with CTCF to control cohesin positioning and genome organization. *Proc. Natl. Acad. Sci. USA* **118**, e2023127118 (2021). 10.1073/pnas.2023127118 PMC7896315 33558242

[43] Ortabozkoyun, H. et al. Members of an array of zinc-finger proteins specify distinct Hox chromatin boundaries. *Mol. Cell* **84**, 3406–3422.e3406 (2024). 39173638 10.1016/j.molcel.2024.08.007 PMC11613955

[44] Hirayama, T. & Yagi, T. Regulation of clustered protocadherin genes in individual neurons. *Semin Cell Dev. Biol.* **69**, 122–130 (2017). 28591566 10.1016/j.semcdb.2017.05.026

[45] Flaherty, E. & Maniatis, T. The role of clustered protocadherins in neurodevelopment and neuropsychiatric diseases. *Curr. Opin. Genet Dev.* **65**, 144–150 (2020). 32679536 10.1016/j.gde.2020.05.041 PMC7750918

[46] Jia, Z. et al. Tandem CTCF sites function as insulators to balance spatial chromatin contacts and topological enhancer-promoter selection. *Genome Biol.* **21**, 75 (2020). 32293525 10.1186/s13059-020-01984-7 PMC7087399

[47] Hirayama, T., Tarusawa, E., Yoshimura, Y., Galjart, N. & Yagi, T. CTCF is required for neural development and stochastic expression of clustered Pcdh genes in neurons. *Cell Rep.* **2**, 345–357 (2012). 22854024 10.1016/j.celrep.2012.06.014

[48] Gu, B. et al. Transcription-coupled changes in nuclear mobility of mammalian cis-regulatory elements. *Science* **359**, 1050–1055 (2018). 29371426 10.1126/science.aao3136 PMC6590518

[49] Tarjan, D. R., Flavahan, W. A. & Bernstein, B. E. Epigenome editing strategies for the functional annotation of CTCF insulators. *Nat. Commun.* **10**, 4258 (2019). 31534142 10.1038/s41467-019-12166-w PMC6751197

[50] Pollex, T. et al. Enhancer-promoter interactions become more instructive in the transition from cell-fate specification to tissue differentiation. *Nat. Genet.* **56**, 686–696 (2024). 38467791 10.1038/s41588-024-01678-x PMC11018526

[51] Fulco, C. P. et al. Activity-by-contact model of enhancer-promoter regulation from thousands of CRISPR perturbations. *Nat. Genet.* **51**, 1664–1669 (2019). 31784727 10.1038/s41588-019-0538-0 PMC6886585

[52] Kagey, M. H. et al. Mediator and cohesin connect gene expression and chromatin architecture. *Nature* **467**, 430–435 (2010). 20720539 10.1038/nature09380 PMC2953795

[53] Krijger, P. H. L., Geeven, G., Bianchi, V., Hilvering, C. R. E. & de Laat, W. 4C-seq from beginning to end: A detailed protocol for sample preparation and data analysis. *Methods* **170**, 17–32 (2020). 31351925 10.1016/j.ymeth.2019.07.014

[54] Schmidl, C., Rendeiro, A. F., Sheffield, N. C. & Bock, C. ChIPmentation: fast, robust, low-input ChIP-seq for histones and transcription factors. *Nat. Methods* **12**, 963–965 (2015). 26280331 10.1038/nmeth.3542 PMC4589892

[55] Langmead, B. & Salzberg, S. L. Fast gapped-read alignment with Bowtie 2. *Nat. methods* **9**, 357–359 (2012). 22388286 10.1038/nmeth.1923 PMC3322381

[56] Zhang, Y. et al. Model-based analysis of ChIP-Seq (MACS). *Genome Biol.* **9**, R137 (2008). 18798982 10.1186/gb-2008-9-9-r137 PMC2592715

[57] Vorontsov, I. E. et al. HOCOMOCO in 2024: A rebuild of the curated collection of binding models for human and mouse transcription factors. *Nucleic Acids Res* **52**, D154–D163 (2024). 37971293 10.1093/nar/gkad1077 PMC10767914

[58] Grant, C. E., Bailey, T. L. & Noble, W. S. FIMO: Scanning for occurrences of a given motif. *Bioinformatics* **27**, 1017–1018 (2011). 21330290 10.1093/bioinformatics/btr064 PMC3065696

[59] Kim, D., Langmead, B. & Salzberg, S. L. HISAT: a fast spliced aligner with low memory requirements. *Nat. Methods* **12**, 357–360 (2015). 25751142 10.1038/nmeth.3317 PMC4655817

[60] Liao, Y., Smyth, G. K. & Shi, W. featureCounts: An efficient general purpose program for assigning sequence reads to genomic features. *Bioinformatics* **30**, 923–930 (2014). 24227677 10.1093/bioinformatics/btt656

[61] Love, M. I., Huber, W. & Anders, S. Moderated estimation of fold change and dispersion for RNA-seq data with DESeq2. *Genome Biol.* **15**, 550 (2014). 25516281 10.1186/s13059-014-0550-8 PMC4302049

[62] Yue, F. et al. A comparative encyclopedia of DNA elements in the mouse genome. *Nature* **515**, 355–364 (2014). 25409824 10.1038/nature13992 PMC4266106

[63] Hnisz, D. et al. Super-enhancers in the control of cell identity and disease. *Cell* **155**, 934–947 (2013). 24119843 10.1016/j.cell.2013.09.053 PMC3841062

[64] Lieberman-Aiden, E. et al. Comprehensive mapping of long-range interactions reveals folding principles of the human genome. *Science* **326**, 289–293 (2009). 19815776 10.1126/science.1181369 PMC2858594

[65] Abdennur, N. & Mirny, L. A. Cooler: Scalable storage for Hi-C data and other genomically labeled arrays. *Bioinformatics* **36**, 311–316 (2020). 31290943 10.1093/bioinformatics/btz540 PMC8205516

[66] Hao, Z. et al. RIdeogram: drawing SVG graphics to visualize and map genome-wide data on the idiograms. *PeerJ Comput Sci.* **6**, e251 (2020). 33816903 10.7717/peerj-cs.251 PMC7924719

[67] Navarro Gonzalez, J. et al. The UCSC Genome Browser database: 2021 update. *Nucleic Acids Res* **49**, D1046–D1057 (2021). 33221922 10.1093/nar/gkaa1070 PMC7779060

[68] Xu W. JinLabBioinfo/FIN_project: Functional insulator code. (2026).

