# Supplementary material for: Cross-platform Hi-C meta-analysis identifies functional insulators that actively block enhancer-promoter interactions
Source: Nat Commun. 2026 Jul 16;17:8709. doi: 10.1038/s41467-026-75801-3 (PMC13490593; doi:10.1038/s41467-026-75801-3)
Supplement: Supplementary file 1 [file 41467_2026_75801_MOESM1_ESM.pdf]

**Cross-platform Hi-C meta-analysis identifies functional insulators that actively block enhancer-promoter interactions**

Jian Cui[1,*], Wanying Xu[1,2,*], Xiuyuan Lang[1,2,*], Shanshan Zhang[1,2], Leina Lu[1], Xiaoxiao Liu[1], Yan Li[1,#], Fulai Jin[1,3,4,5,#]

[#] Corresponding authors: Yan Li (yxl1379@case.edu); Fulai Jin (fxj45@case.edu)

**Supplementary Information (11 supplementary figures)**

**Supplementary Figure 1.** Compare the compartments and TADs between orthogonal CTCF-degron datasets from Hi-C, in situ Hi-C, and micro-C.

**Supplementary Figure 2.** Compare the loop-level analyses of orthogonal Hi-C datasets with *ICE, HiCorr*, and *DeepLoop*, related to Fig. 1b.

**Supplementary Figure 3.** Correlation matrices comparing the profiles of dynamic loops from all nine pairs of degron experiments, related to Fig. 1c.

**Supplementary Figure 4.** Recurrent gain of E-P loop at *Notch1* locus between independent CTCF-degron Hi-C studies, related to Fig. 1d,e.

**Supplementary Figure 5.** Meta-analysis of independent CTCF-degron Hi-C datasets to examine the consistency and epigenetic features of dynamic loops.

**Supplementary Figure 6.** Meta-analysis of independent RAD21- and WAPL- degron Hi-C datasets to examine the consistency and epigenetic features of dynamic loops.

**Supplementary Figure 7.** "Gained" long-range H3K27me3 loops in RAD21-depleted cells from Rhodes *et al*. exist before RAD21-depletion in two other studies.

**Supplementary Figure 8.** Meta-analysis reveals the fate of dynamic loops from CTCF-degron studies upon depletion of cohesin factors.

**Supplementary Figure 9.** Examples of loop gaining loci, related to Fig. 3d.

**Supplementary Figure 10.** Limited overlap between FINs and TAD boundaries, related to Fig. 3f,h,i.

**Supplementary Figure 11.** Example contact heatmaps at putative insulator target genes, related to Fig. 4h,j.

Supplementary Figure 1

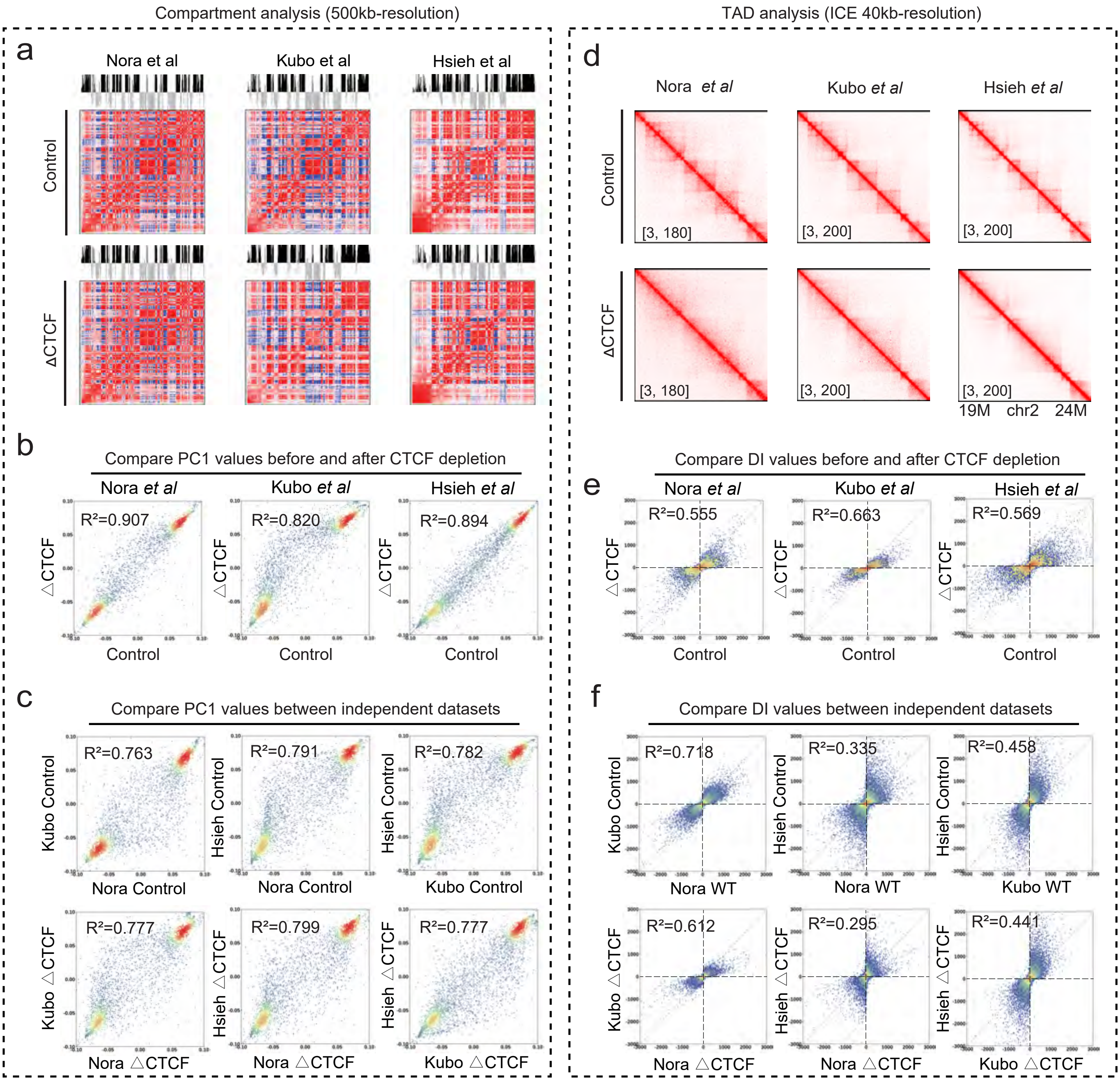

**Supplementary Figure 1. Compare the compartments and TADs between orthogonal CTCF-degron datasets from Hi-C, in situ Hi-C, and micro-C.**

**a-c,** Compartment analysis of CTCF-degron Hi-C datasets at 500kb resolution. **a,** Correlation heatmaps of the three CTCF-degron Hi-C datasets at 500kb resolution showing the compartments in control and CTCF-depleted cells. PC1 values are shown on top of each heatmap indicating compartment A (positive PC1) and B (negative PC1). **b**, Scatter plots comparing the PC1 values for each 500kb bin before and after CTCF depletion. **c,** Top row: scatter plots comparing the PC1 values of Hi-C data in control cells from three independent studies; bottom row: scatter plots comparing the PC1 values of Hi-C data in CTCF-depleted cells from three studies. **d-f,** TAD analysis of CTCF-degron Hi-C datasets at 40kb resolution. **d,** *ICE* normalized contact heatmaps of the three CTCF-degron Hi-C datasets at 40kb resolution. TADs are recognizable as squares along the diagonals. Note the loss of TADs in CTCF-depleted cells. **e,** Scatter plots compare the directionality index (DI) values of all 40kb bins before and after CTCF-depletion in all three studies. Note that the DI values of many bins become close to zero in CTCF-depleted cells, indicating that these bins lost directional biases, consistent with massive loss-of-TAD upon CTCF-depletion. **f,** Top row: scatter plots comparing the DI values of all 40kb bins in control cells from three independent studies; bottom row: scatter plots comparing the DI values of all 40kb bins in CTCF-depleted cells from three studies. Note that although the R-squares between Nora *et al*. and Kubo *et al*. are rather high (R-square 0.718 and 0.612 for control and △CTCF cells), the DI values from micro-C data (Hsieh *et al.*) deviate significantly from the other two studies (R-square 0.295~0.458). In micro-C data, more bins show polarized DI values (*i.e.*, stronger directionality biases), consistent with the general belief that with *ICE*-based pipeline, micro-C identifies more loops than Hi-C or *in situ* Hi-C.

# Supplementary Figure 2

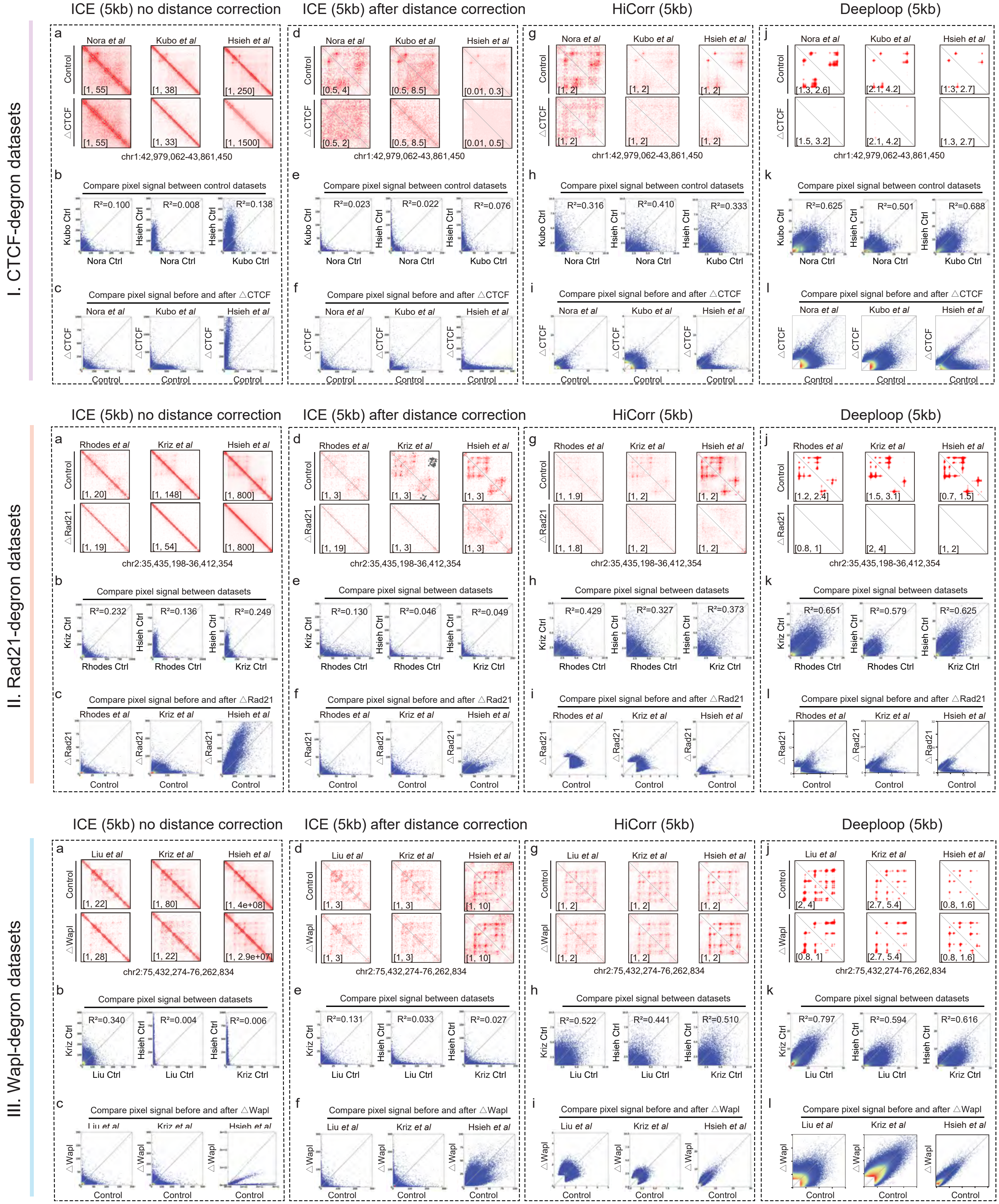

**Supplementary Figure 2. Compare the loop-level analyses of orthogonal Hi-C datasets with ICE, HiCorr, and DeepLoop, related to Fig. 1b.**

**Part I.** Cross-platform comparison of CTCF-degron datasets. **a-c,** Loop analysis of three CTCF-degron Hi-C datasets with *ICE*. **a,** *ICE* heatmaps of the three CTCF-degron Hi-C datasets. Hi-C (Nora) and *in situ* Hi-C (Kubo) heatmaps show more noise than micro-C (Hsieh) heatmaps. **b,** Scatter plots of *ICE* heatmap pixels comparing the control contact heatmaps from three studies. The consistency between orthogonal Hi-C protocols is low (R-square values close to zero). **c**, Scatter plots comparing the *ICE* heatmaps of control and ΔCTCF cells in three studies. Every dot represents one pixel in the contact matrix and the x-axis and y-axis indicate the intensity of the pixel in two experiments. △CTCF cells have many pixels stronger than WT cells because the read-depth of micro-C data in △CTCF cells is higher than in control cells. **d-f,** Similar as **a-c** but the comparisons are between *ICE* heatmaps after correcting distance biases, which are observed/expected ratio heatmaps. Note that in **f**, the control vs. △CTCF scatter plot for Hsieh *et al.* correctly shows stronger loop pixels in control than △CTCF cells. However, the scatter plots in **e** show poor cross-study consistency with R-square values close to zero, indicating that the pixel-level correlations are dominated by random noise pixels. **g-i,** the same comparisons between *HiCorr*-corrected heatmaps, which are also distance-corrected ratio heatmaps. The scatter plots in **i** show that in each of the control vs. △CTCF comparisons, and we start to observe retained pixels with similar strength before and after CTCF depletion. Furthermore, the scatter plots in **h** also show that *HiCorr* achieves higher R-square values across different Hi-C platforms. **j-l,** comparisons between *DeepLoop* heatmaps. *DeepLoop* highlights loop pixels and suppresses noise pixels (**j**). The retained pixels and weakened pixels are more clearly recognizable in each of the control vs. △CTCF scatter plots (**l**). *DeepLoop* shows the best cross-platform consistency at pixel level (**k**).

**Part II.** Cross-platform comparison of RAD21-degron datasets. Same as Part I and all conclusions are the same.

**Part III.** Cross-platform comparison of WAPL-degron datasets. Same as Part I and all conclusions are the same.

# Supplementary Figure 3

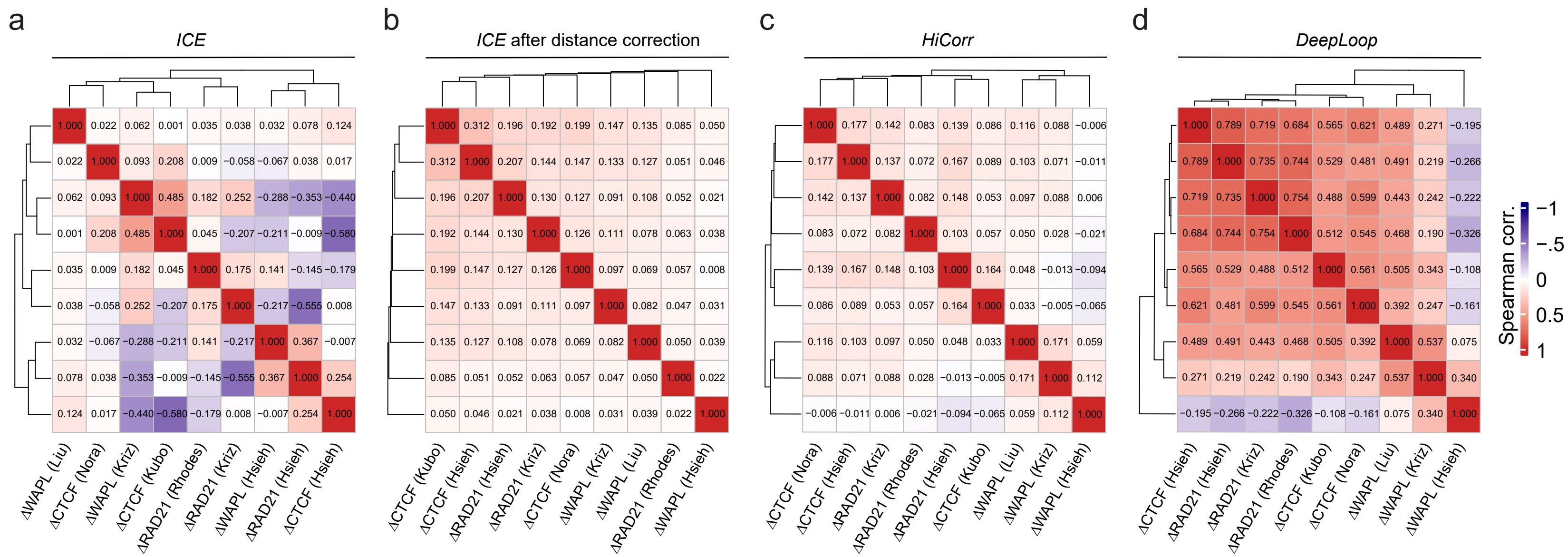

**Supplementary Figure 3. Correlation matrices comparing the profiles of dynamic loops from all nine pairs of degron experiments, related to Fig. 1c.**

For each degron experiment, we first obtain a pair of 5kb resolution Hi-C contact matrices in control and depletion cells. Next, we computed the delta matrices (depletion – control) for all nine experiments. A correlation matrix is then generated between the nine delta matrices. **a-d** are correlation matrices when contact matrices are generated with different pipelines.

# Supplementary Figure 4

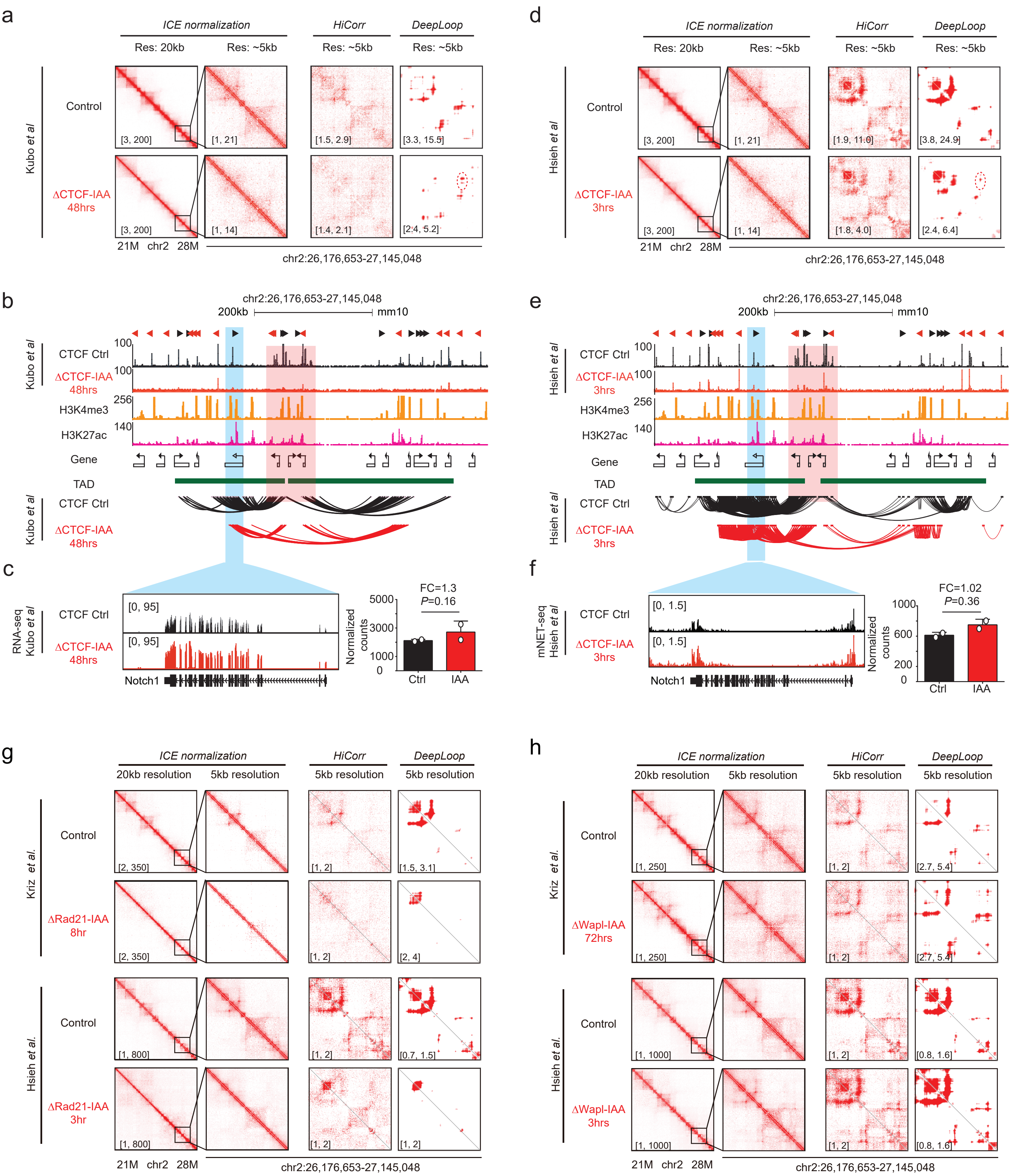

**Supplementary Figure 4. Recurrent gain of E-P loop at *Notch1* locus between independent CTCF-degron Hi-C studies, related to Fig. 1d,f.**

**a,** Contact heatmaps of the CTCF-degron Hi-C data by Kubo *et al.* at *Notch1* locus with *ICE*, *HiCorr* and *DeepLoop*. Red dashed circle highlights the same locations where we observed a gained E-P loop in the contact heatmap from Nora *et al.* (shown in Fig. 1d). **b**, Genome browser tracks showing ChIP-seq data and Hi-C loops from Kubo *et al.* Blue highlighted region indicates *Notch1* gene. Pink region highlights the potential insulator region that blocks the formation of *Notch1* E-P loop. **c,** RNA-seq results from Kubo *et al.* are also shown as bar plot. p-value: one-side Wald test provided by DE-seq2. **d-f,** the same analysis as **a-c** at *Notch1* locus using data from Hsieh *et al.* Note that Hsieh *et al.* used mNET-seq for transcriptome profiling. **g,** Contact heatmaps of *Notch1* locus from two RAD21-degron datasets (Kriz *et al.* and Hsieh *et al.*) in addition to the one included in Fig. 1f (Rhodes *et al.*). **h,** Contact heatmaps of *Notch1* locus from two WAPL- degron datasets (Kriz *et al.* and Hsieh *et al.*) in addition to the one included in Fig. 1f (Liu *et al.*).

# Supplementary Figure 5

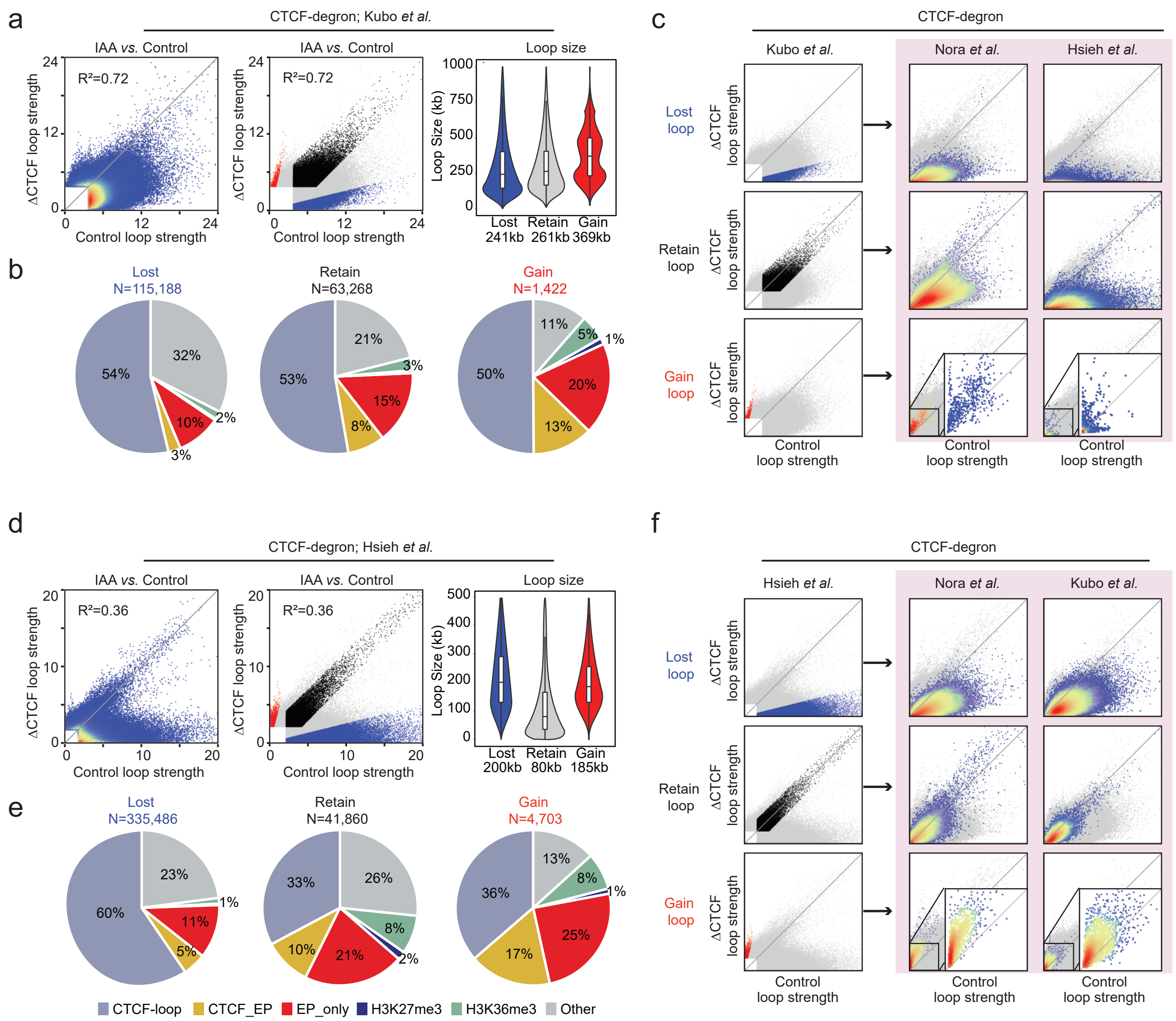

**Supplementary Figure 5. Meta-analysis of independent CTCF-degron Hi-C datasets to examine the consistency and epigenetic features of dynamic loops.**

**a**, Using the *in situ* Hi-C data from the CTCF-degron study by Kubo *et al.*, left: scatterplot comparing the strength of loop pixels between WT and CTCF-depleted mESCs; middle: the same scatterplot as left but highlight the lost (blue), retained (black), and gained (red) loop pixels; right: violin plot comparing the size of loop pixels. **b,** Pie charts showing the components of lost, retained and gained loop pixels based on epigenetic marks. **c**, Scatter plots in the left column highlight the lost, retained, and gained loop pixels from Kubo *et al*., the right two columns show the quantitative changes of the corresponding pixels in Nora *et al.* or Hsieh *et al*. **d-f,** The same analyses as **a-c** are performed with CTCF-degron micro-C data by Hsieh *et al.*

# Supplementary Figure 6

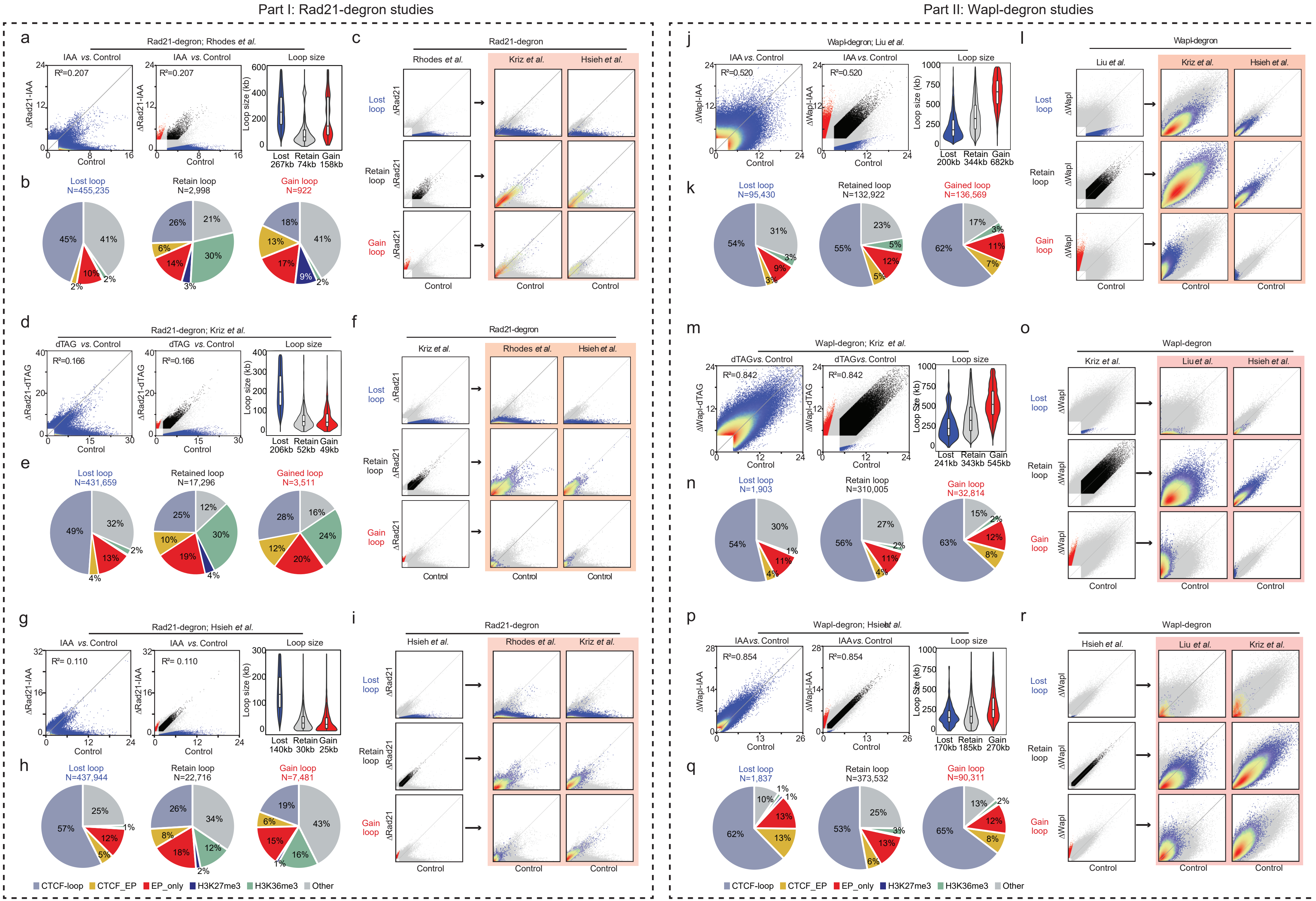

**Supplementary Figure 6. Meta-analysis of independent RAD21- and WAPL- degron Hi-C datasets to examine the consistency and epigenetic features of dynamic loops.**

**Part I:** Meta-analysis of RAD21-degron datasets. **a,** Left and middle panels define the lost, retained, and gained loop pixels upon RAD21-depletion from the study by Rhodes *et al.*, and the right panel compares their loop size distribution. **b,** Pie charts showing the components of lost, retained and gained loops based on epigenetic marks. **c,** Scatter plots testing if the lost, retained and gained loop pixels from Rhodes *et al.* are quantitatively consistent in the other two RAD21-degron studies. **d-f,** the same analyses as **a-c** are performed with RAD21-degron Hi-C data by Kriz *et al.* **g-i,** the same analyses as **a-c** are performed with RAD21-degron micro-C data by Hsieh *et al.*

**Part II:** Meta-analysis of WAPL-degron datasets. **j-r,** Same as Part I except that the analyses were done with WAPL-degron datasets. Note there is an overall trend that WAPL-depletion causes more “gained” loops.

Supplementary Figure 7

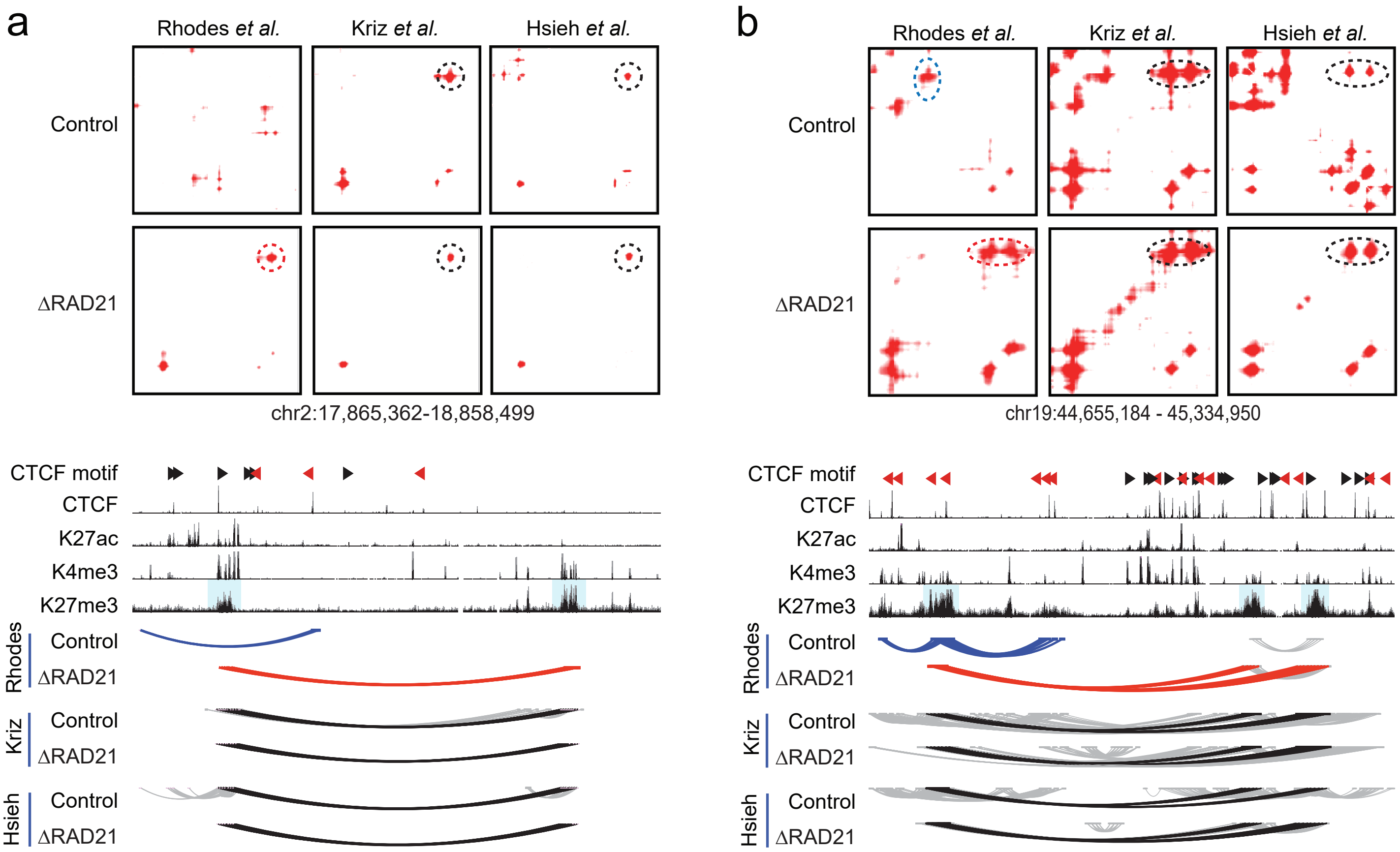

**Supplementary Figure 7. “Gained” long-range H3K27me3 loops in RAD21-depleted cells from Rhodes *et al.* exist before RAD21-depletion in two other studies.**

Two examples are shown in **a** and **b**. In each example, Hi-C data are shown as both contact heatmaps (*DeepLoop* enhanced) and loop arches. Browser tracks of CTCF, H3K27ac, H3K4me3, and H3K27me3 are also included. Note that the gained H3K27me3 loop in Rhodes et al. already exist before RAD21-depletion in two other studies.

Supplementary Figure 8

a

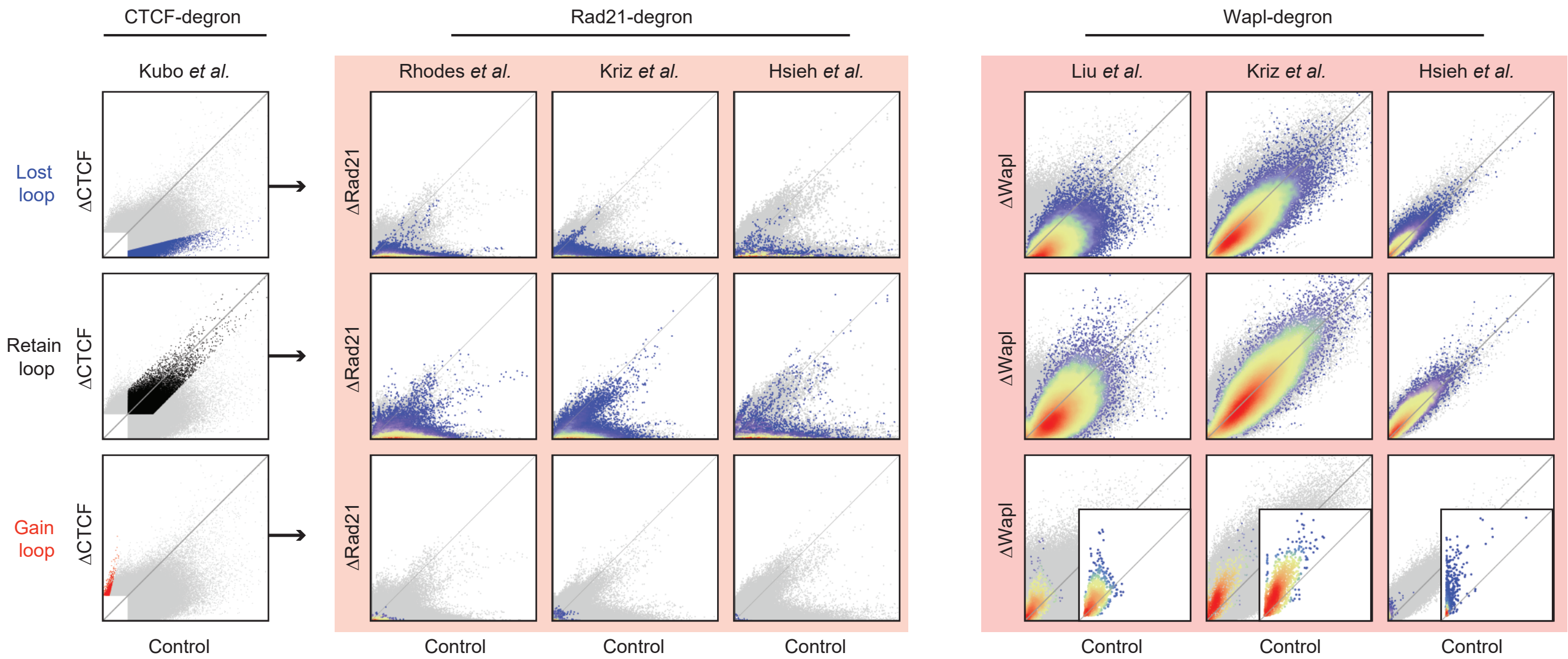


b

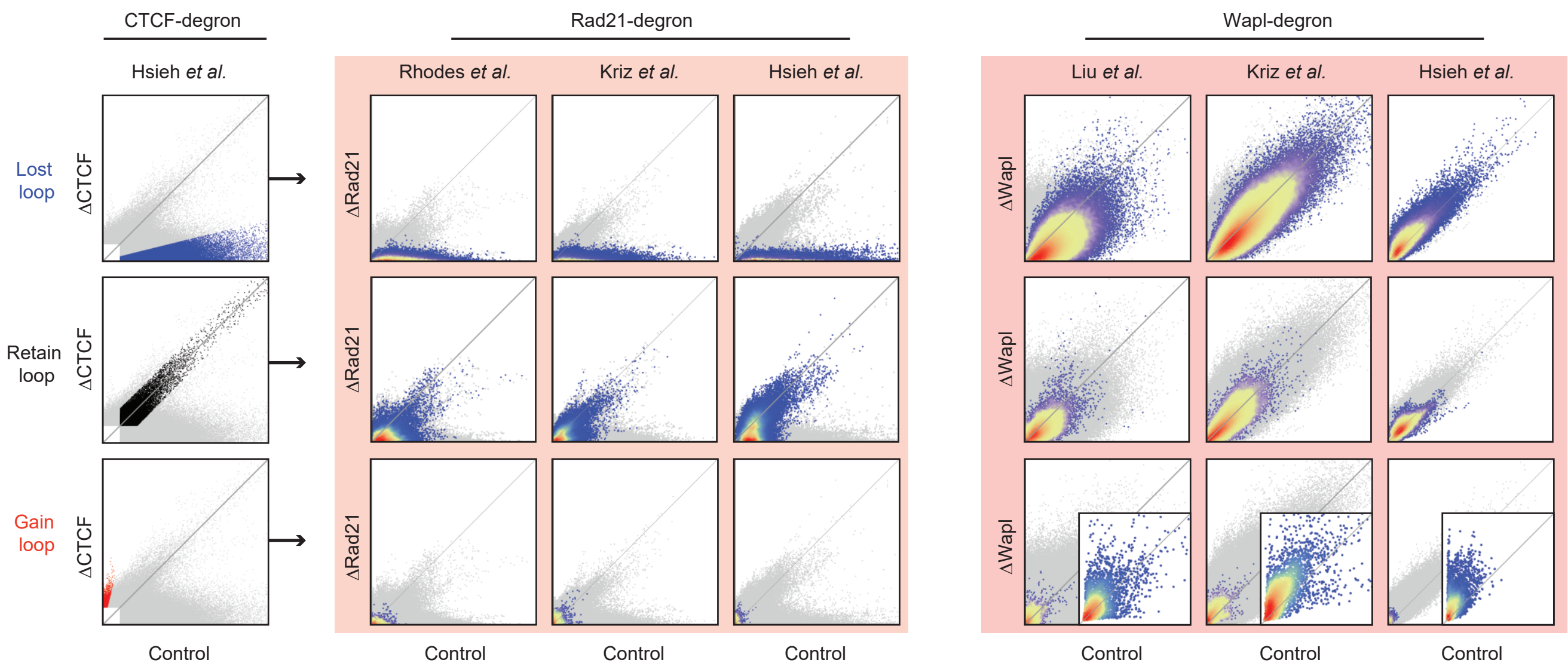

**Supplementary Figure 8. Meta-analysis reveals the fate of dynamic loops from CTCF-degron studies upon depletion of cohesin factors.**

**a,** The lost, retained, and gained loop pixels from one CTCF-degron Hi-C data (Kubo *et al.*) are plotted onto Hi-C scatter plots from RAD21-or WAPL-degron studies to examine their quantitative changes. **b,** Same analyses are done with the lost, retained, and gained loop pixels from the CTCF-degron Hi-C data by Hsieh *et al.*

## Supplementary Figure 9

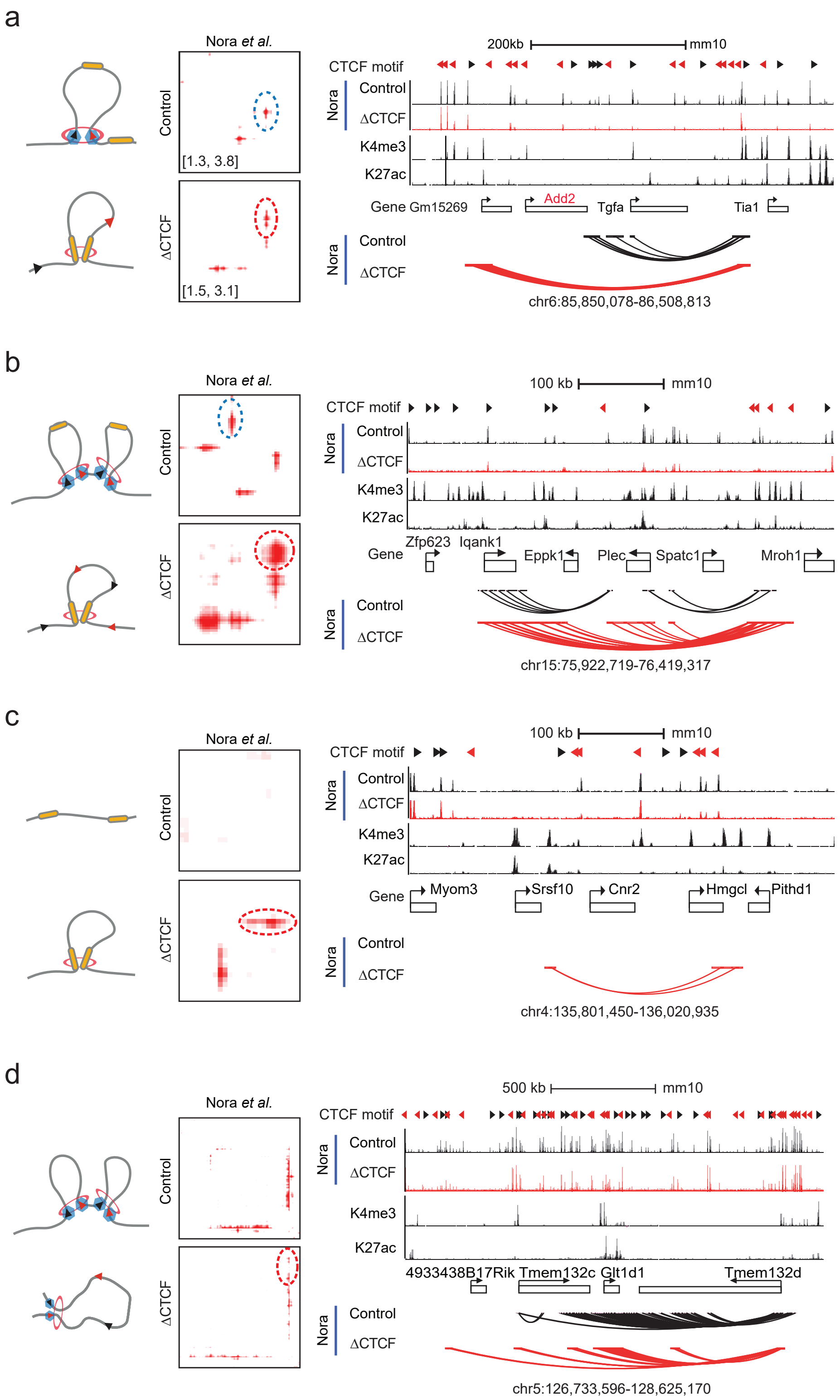

**Supplementary Figure 9. Examples of loop gaining loci, related to Fig. 3d.**

**a,** An example of Type I EP loop gaining loci, in which one of the two E/P sites is insulated in a CTCF loop while the other site is outside the CTCF loop; **b,** An example of Type II EP-loop gaining loci, in which two E/P sites are insulated in two CTCF loops; **c,** An example of Type III EP loop gaining loci, in which neither of the two E/P sites are insulated by CTCF loops; **d,** An example of CTCF loop gaining loci, in which the newly gained loop are between incompletely degraded CTCF sites.

# Supplementary Figure 10

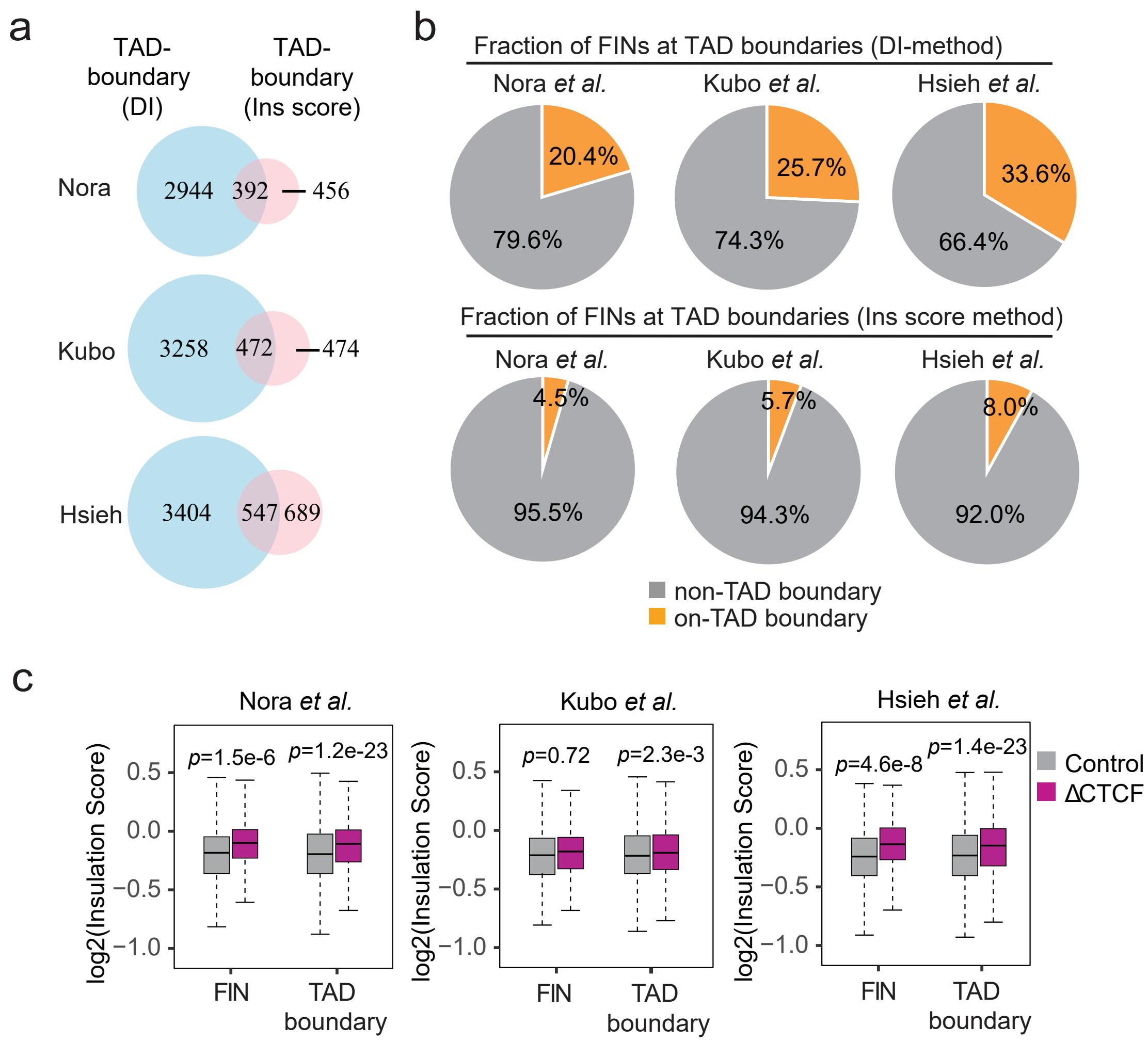

**Supplementary Figure 10. Limited overlap between FINs and TAD boundaries, related to Fig. 3f,h,i.**

**a,** Venn diagrams comparing the TAD boundaries called by two methods from three studies. **b,** Pie chart showing the percentage of FINs that overlap TAD boun-23daries called from different datasets and algorithms. **c,** Compare insulation scores at FINs and TAD boundaries in different studies. Boxplots show median ± upper and lower quartiles. P-values: two-sided Wilcoxon rank-sum test.

# Supplementary Figure 11

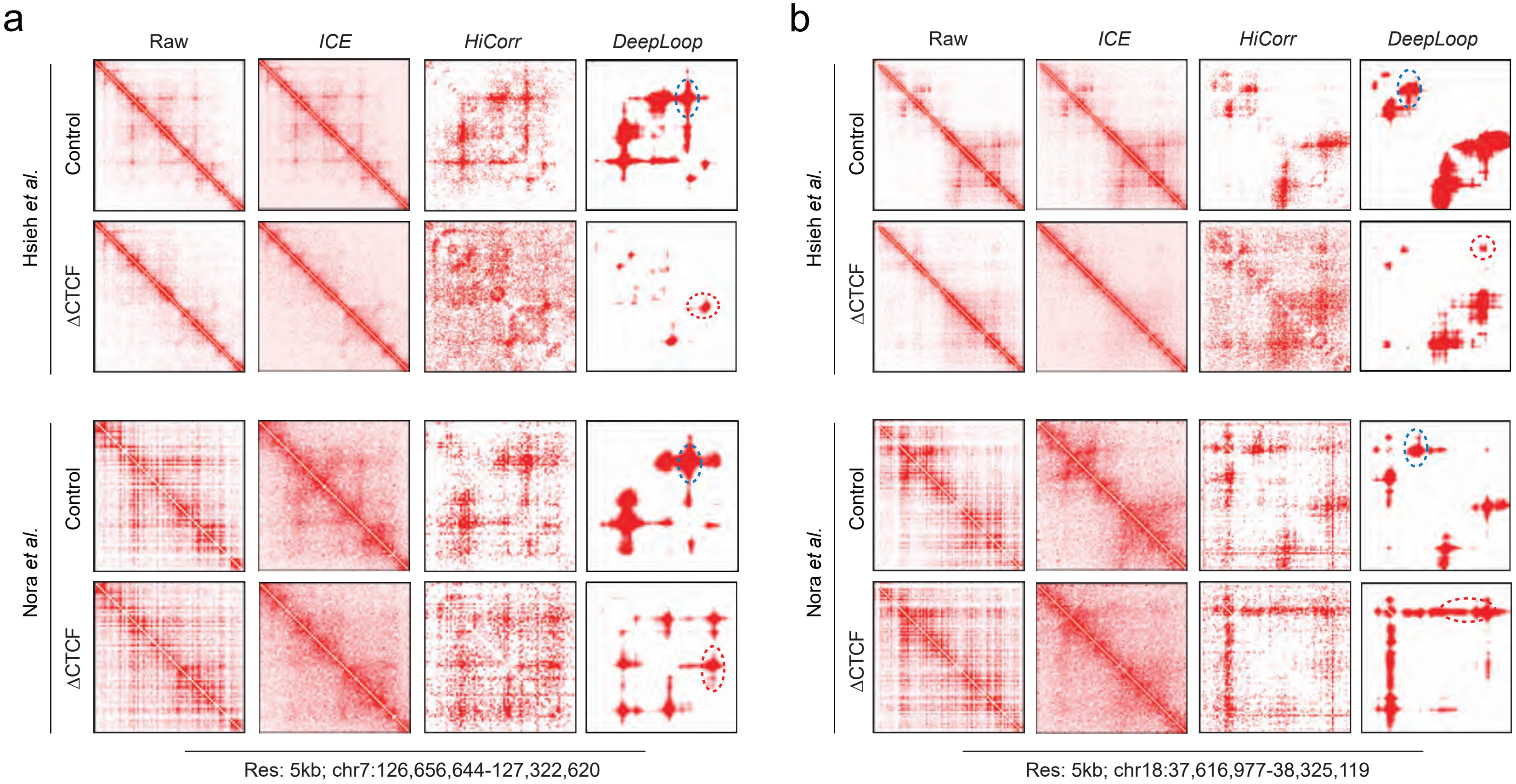

**Supplementary Figure 11. Example contact heatmaps at putative insulator target genes, related to Fig. 4h,j.**

**a,** Raw, *ICE*, *HiCorr*, and *DeepLoop* contact heatmaps at *Cdiptos* locus in control and CTCF-depleted cells from Hsieh *et al.* and Nora *et al.* **b,** Similar as **a** but the heatmaps are at the *Pcdh*-γ locus.
