# Supplementary material for: Cross-platform Hi-C meta-analysis identifies functional insulators that actively block enhancer-promoter interactions
Source: Nat Commun. 2026 Jul 16;17:8709. doi: 10.1038/s41467-026-75801-3 (PMC13490593; doi:10.1038/s41467-026-75801-3)
Supplement: Supplementary file 2 [file 41467_2026_75801_MOESM2_ESM.pdf]

Description of Additional Supplementary Files

Supplementary Data 1. Availability of public Hi-C/micro-C data: The accession number for reanalyzed data.

Supplementary Data 2. Summary of reanalyzed Hi-C/micro-C data: Read summary and regression slope for pair-wise loop comparison

Supplementary Data 3. Integrated table of all RNA-seq data generated by public architectural protein KO paper

Supplementary Data 4. Reproducing loop-gaining loci: summary of identified reproducing loop-gaining loci on genome scale within mESCs

Supplementary Data 5. sgRNA, CARGO and dCas9 sequence designed for FINs validation

Supplementary Data 6. Restriction enzyme and PCR primers of 4C experiments

Supplementary Data 7. CTCF ChIP-seq and CUT&RUN data summary of two FINs

Supplementary Data 8. qt-PCR summary of FIN target gene at two validated FINs

Supplementary Data 9. Topological associated domains (TADs) list in mESCs

Supplementary Data 10. Super Enhancer list in mESCs

Supplementary Data 11. Regular enhancer peak list in mESC

Supplementary Data 12. Compartment calling of WT-mESC

Supplementary Data 13. List of recurrent gained loop pixels

Supplementary Data 14. FINs: list of all FINs identified in mESCs
