# Supplementary material for: Cross-platform Hi-C meta-analysis identifies functional insulators that actively block enhancer-promoter interactions
Source: Nat Commun. 2026 Jul 16;17:8709. doi: 10.1038/s41467-026-75801-3 (PMC13490593; doi:10.1038/s41467-026-75801-3)
Supplement: Supplementary file 3 [file 41467_2026_75801_MOESM4_ESM.pdf]

nature portfolio

Corresponding author(s): Fulai Jin, Yan Li

Last updated by author(s): Apr 7, 2026

Reporting Summary

Nature Portfolio wishes to improve the reproducibility of the work that we publish. This form provides structure for consistency and transparency in reporting. For further information on Nature Portfolio policies, see our Editorial Policies and the Editorial Policy Checklist.

Statistics

For all statistical analyses, confirm that the following items are present in the figure legend, table legend, main text, or Methods section.

n/a	Confirmed	
☐	☒	The exact sample size (*n*) for each experimental group/condition, given as a discrete number and unit of measurement
☐	☒	A statement on whether measurements were taken from distinct samples or whether the same sample was measured repeatedly
☐	☒	The statistical test(s) used AND whether they are one- or two-sided<br>*Only common tests should be described solely by name; describe more complex techniques in the Methods section.*
☒	☐	A description of all covariates tested
☒	☐	A description of any assumptions or corrections, such as tests of normality and adjustment for multiple comparisons
☐	☒	A full description of the statistical parameters including central tendency (e.g. means) or other basic estimates (e.g. regression coefficient) AND variation (e.g. standard deviation) or associated estimates of uncertainty (e.g. confidence intervals)
☐	☒	For null hypothesis testing, the test statistic (e.g. *F*, *t*, *r*) with confidence intervals, effect sizes, degrees of freedom and *P* value noted<br>*Give P values as exact values whenever suitable.*
☒	☐	For Bayesian analysis, information on the choice of priors and Markov chain Monte Carlo settings
☒	☐	For hierarchical and complex designs, identification of the appropriate level for tests and full reporting of outcomes
☐	☒	Estimates of effect sizes (e.g. Cohen's *d*, Pearson's *r*), indicating how they were calculated

Our web collection on statistics for biologists contains articles on many of the points above.

Software and code

Policy information about availability of computer code

Data collection	No software used
Data analysis	Softwares: bowtie (v.1.1.2), Deeploop, pipe4C (v.1.1.3), HISAT2(v.2.1.0), DESeq2(v.1.26.0), TrimGalore (v.0.6.7), FeatureCounts (v.1.6.1), MACS2(v.2.2.7.1), FIMO (v.4.11.2), HOCOMOCO v11, cooltools (v.0.7.1), Rideogram (v.0.2.2),bedtools (v.2.25.0), pipe4C (v.1.1.3)<br>Custom code: https://github.com/JinLabBioinfo/FIN_project

For manuscripts utilizing custom algorithms or software that are central to the research but not yet described in published literature, software must be made available to editors and reviewers. We strongly encourage code deposition in a community repository (e.g. GitHub). See the Nature Portfolio guidelines for submitting code & software for further information.

Data

Policy information about availability of data

All manuscripts must include a data availability statement. This statement should provide the following information, where applicable:

- Accession codes, unique identifiers, or web links for publicly available datasets
- A description of any restrictions on data availability
- For clinical datasets or third party data, please ensure that the statement adheres to our policy

The raw data of 4C-seq and ChIP-seq generated in this study, and reanalyzed published data can be found at accession number GSE243728

Research involving human participants, their data, or biological material

Policy information about studies with human participants or human data. See also policy information about sex, gender (identity/presentation), and sexual orientation and race, ethnicity and racism.

Reporting on sex and gender	Does not apply
Reporting on race, ethnicity, or other socially relevant groupings	Does not apply
Population characteristics	Does not apply
Recruitment	Does not apply
Ethics oversight	Does not apply

Note that full information on the approval of the study protocol must also be provided in the manuscript.

Field-specific reporting

Please select the one below that is the best fit for your research. If you are not sure, read the appropriate sections before making your selection.

☒ Life sciences ☐ Behavioural & social sciences ☐ Ecological, evolutionary & environmental sciences

For a reference copy of the document with all sections, see nature.com/documents/nr-reporting-summary-flat.pdf

Life sciences study design

All studies must disclose on these points even when the disclosure is negative.

Sample size	Sample size was not predetermined by statistical methods. As this study analyzes public Hi-C and RNA-seq datasets, sample sizes were determined entirely by the availability of public data
Data exclusions	No data exclusions
Replication	We analyzed publicly available Hi-C and RNA-seq datasets, most of which contained at least two independent biological replicates. The use of multiple replicates enabled us to verify the reproducibility of our findings, and all replicated analyses produced consistent results
Randomization	Randomization was not relevant to this study.
Blinding	Blinding was not applicable to this study because we are analyzing published datasets. There are no group allocations

Reporting for specific materials, systems and methods

We require information from authors about some types of materials, experimental systems and methods used in many studies. Here, indicate whether each material, system or method listed is relevant to your study. If you are not sure if a list item applies to your research, read the appropriate section before selecting a response.

Materials & experimental systems

n/a	Involved in the study	
☐	☒	Antibodies
☐	☒	Eukaryotic cell lines
☒	☐	Palaeontology and archaeology
☒	☐	Animals and other organisms
☒	☐	Clinical data
☒	☐	Dual use research of concern
☒	☐	Plants

Methods

n/a	Involved in the study	
☐	☒	ChIP-seq
☒	☐	Flow cytometry
☒	☐	MRI-based neuroimaging

Antibodies

Antibodies used	CTCF antibody: Abcam, #ab70303

Validation	CTCF (Abcam, #ab70303): Manufacturer's website states the antibody is specific for human and mouse, validated for Western Blot, IP, IHC-P

Eukaryotic cell lines

Policy information about cell lines and Sex and Gender in Research

Cell line source(s)	E14Tg2a (CRL-1821) were ordered from ATCC
Authentication	The E14Tg2a cell line was not independently authenticated by STR profiling or other genotyping methods
Mycoplasma contamination	The E14Tg2a cell line tested negative for mycoplasma contamination.
Commonly misidentified lines (See ICLAC register)	No commonly misidentified cell lines

Plants

Seed stocks	Does not apply
Novel plant genotypes	Does not apply
Authentication	Does not apply

ChIP-seq

Data deposition

☒ Confirm that both raw and final processed data have been deposited in a public database such as GEO.

☒ Confirm that you have deposited or provided access to graph files (e.g. BED files) for the called peaks.

Data access links *May remain private before publication.*	https://www.ncbi.nlm.nih.gov/geo/query/acc.cgi?acc=GSE243728
Files in database submission	raw data, processed data (bed file)
Genome browser session (e.g. UCSC)	https://genome.ucsc.edu/cgi-bin/hgTracks?db=mm10&lastVirtModeType=default&lastVirtModeExtraState=&virtModeType=default&virtMode=0&nonVirtPosition=&position=chr18%3A37616977%2D38325119&hgsid=3395591143_JIK7qIbsAAU7aqvr8r9aI4oWRXfn

Methodology

Replicates	NA
Sequencing depth	CTCF ChIP-seq (Control): 28M raw reads, 12M uniquely mapped reads<br>CTCF ChIP-seq (dCas9): 20M raw reads, 7M uniquely mapped reads<br>CTCF CUT&RUN (Control): 41M raw reads, 6M uniquely mapped reads<br>CTCF CUT&RUN (dCas9): 98M raw reads, 13M uniquely mapped reads
Antibodies	CTCF (Abcam, #ab70303)
Peak calling parameters	macs2 callpeak -f BAM -t file.duplicatedRemoved.bam -n file
Data quality	NA
Software	MACS2, bowtie
