# Supplementary material for: Cross-platform Hi-C meta-analysis identifies functional insulators that actively block enhancer-promoter interactions
Source: Nat Commun. 2026 Jul 16;17:8709. doi: 10.1038/s41467-026-75801-3 (PMC13490593; doi:10.1038/s41467-026-75801-3)
Supplement: Supplementary file 4 [file 41467_2026_75801_MOESM5_ESM.pdf]

nature portfolio

Peer Review File

Cross-platform Hi-C meta-analysis identifies functional insulators that actively block enhancer-promoter interactions

Corresponding Author: Dr Fulai Jin

Parts of this Peer Review File have been redacted as indicated to remove third-party material.

This file contains all reviewer reports in order by version, followed by all author rebuttals in order by version.

Version 0:

Reviewer comments:

Reviewer #1

(Remarks to the Author)
Summary:

The paper entitled « Map functional insulators with cross-platform Hi-C meta-analysis » by Wanying Xu and colleagues describes a new computational approach to analyze chromatin conformation data, in particular Hi-C and micro-C across platforms, identifies putative functional insulators in the genome using this approach, and validates insulator loci experimentally at selected loci. The computational approach used for cross-platform data analysis primarily relies on the DeepLoop pipeline in order to identify loop interactions that are more robust across assays than using conventional methods such as ICE normalization. Applied on datasets from two published Hi-C studies and one micro-C study, DeepLoop identified retained and differential (lost or gained) loop interactions between control conditions and conditions in which either the mammalian insulator protein CTCF, the cohesin subunit RAD21, or the cohesin unloading factor WAPL were depleted. The differential loops are mostly consistent across assays for given conditions, and a subset of loops gained upon CTCF depletion bring together loop anchors that are characterized by epigenetic features associated with promoters and enhancers (cis-regulatory elements), specifically H3K4me3 and H3K27ac. The study hypothesizes that these loops represent newly established enhancer-promoter contacts, which were previously prevented or « blocked » by nearby CTCF-mediated contact points, and proposes the latter contact points as functional insulators (FINs). The study proposes a model of FIN function dependent on loop extrusion, whereby cohesin-mediated contacts between CTCF-bound anchor points at FINs prevent contacts and regulatory communication between cis-regulatory elements (e.g., an enhancer or group of enhancers, and the promoter of the target gene) that are separated by such FIN elements. Differentially expressed genes (DEGs) are enriched at gained loops and the DEGs that are most reproducible between studies tend to be located near FINs. Detailed examination at selected loci including Cdiptos and Pcdh-gamma shows loss of FIN-anchored interactions upon CTCF depletion that coincides with gain in interaction between distal H3K27ac-marked regions and promoter-proximal regions of DEGs. Interfering with CTCF binding using sgRNA/dCas9 at specific CTCF sites in selected candidate FIN regions result in a similar impact at the targeted loci than global CTCF depletion, including gene upregulation and gain of distal interactions of DEGs with H3K27ac-marked regions, thus validating the role of CTCF in the function of these specific FINs. The results of the study suggest that FIN are cis-acting insulators of enhancer-promoter communication in untreated cells.

Overall assessment:

The findings of the study are important for the fields of 3D genome and gene regulation, and could also have a broad impact on the scientific community in general, however there are major concerns with the presentation, analysis and interpretation of the results in the current state of the manuscript. At multiple points throughout the manuscript, some results are over-interpreted, hypotheses are not sufficiently tested to support the conclusions, and there is a lack of consideration for equally plausible alternative explanations to some of the findings. Cause-and-effect implications are often prematurely drawn in the absence of the required functional validation, resulting in missing evidence to support some of the main conclusions of the paper. In addition, the manuscript lacks essential yet basic analyses, such as TAD and compartment calling in the first section, misses or provides only cryptic informations about important analysis parameters (e.g., it is unclear how the overlap between TAD boundaries and FINs was assessed, and the main text lacks basic information about which epigenetic

features were considered to identify putative E-P loops). For these reasons, I advise a major revision of the manuscript according to the point-by-point comments listed below, organized in chronological order with respect to the main text.

Point-by-point comments:

RESULTS

(Major) Line 78: The statement that « for Hi-C and in situ Hi-C studies, conventional ICE-normalization only support compartment- or TAD-level analysis at 20kb or lower resolution due to the lack of read depth » lacks supporting evidence from TAD and compartment calling.

(Major) Line 83: In addition to visual inspection at selected examples of chromosomal regions, the robustness of DeepLoop in cross-platform analysis should also be supported by correlating the DeepLoop signal (e.g., pixel intensity) for the same region pairs between assays, and by contrasting this correlation to the those obtained with standard ICE normalization or HiCorr correction only.

Fig. 1d: Y-axis labels should indicate « loop strength » after « Control » and « deltaCTCF-IAA » conditions.

(Major) Fig. 1e,f: The epigenetic features of promoter and enhancers used to define E-P loops should be summarized directly in the result section. This information is currently only present in the method section.

For contact heatmaps, the meaning of the numbers between brackets should be should be indicated in the figure legend.

For plots of loop size, the figure legend should explicitely indicate that the median loop size is given underneath the plot for each loop categories.

Line 121: For more clarity, modifying the sentence as follows is recommended: « some of them lose strength [upon CTCF degradation] in Hsieh et al. …» .

(Major) Line 126: The data presented are not sufficient to conclude that « Hsieh et al. achieves the best CTCF depletion, perhaps because it uses N-terminal degron system ». Assay-specific biases, differences in sensitivity and in the depth of sequencing could also explain at least some of the discrepancies observed between studies.

Line 132: For more accuracy, modifying the sentence should be modified as follows « Nevertheless, [most of] the "gained pixels" from Nora et al. … ».

Line 138: It is not clear what is meant by « affected genes … are likely to be the direct targets of insulators ». How/why are the affected genes likely to be the targets of insulators?

(Major) Line 143: About the observation that « "retained" and "gained" loop pixels in Rad21-depleted cells are much smaller (mostly within 100kb) than those in CTCF-depleted cells, indicating that cohesin-independent loops are short-range », it is also conceivable that decreasing the pool of available cohesin through depletion of RAD21 allows for only rare and short-lived extrusion events (assuming unchanged levels of the cohesin unloader WAPL upon RAD21 depletion compared to untreated cells), thereby creating smaller-sized, yet still cohesin-dependent loops. The above possibility should be acknowledged in the manuscript.

Line 151: It is not clear what is « the conclusion from the original study ».

Line 147: For accuracy, the following changes are recommended: « The "retained" and "gained" loop pixels in Kriz et al. and Hsieh et al., and "retained" loop pixels in Rhodes et al. In Rad21-depleted cells… »

(Major) The use of the terms « dependent » and « independent » (e.g.,whole paragraph starting line 165) with respect to CTCF or cohesin should be avoided given the possibility that residual amount of these factors could be present in the depleted cells, and the observation that, for example, some loops retained upon CTCF depletion display CTCF peaks at both anchors (44% of retained loops in Nora et al.) and are hence likely to be CTCF-dependent. The use of the terms « retained », « gained » or « lost » upon certain conditions should be favored for accuracy.

(Major) Lines 172-175: The conclusion that « recurrently gained E-P interactions upon CTCF depletion are created by loop extrusion » because « "gained" loops in CTCF-depleted cells do not gain strength in Rad21-depleted cells » seems far-fetched, because these loops also do not show clear loss of strength upon RAD21 depletion (or this result is not properly presented and discusssed), which would be expected if they are created by loop extrusion.

Line 177: « Supp Fig. 5a, b, third row » is mislabelled as « Supp Fig. 5b, d, third row ».

(Major) Fig. 3b: It is unclear how the FIN loci were identified, e.g. how many CTCF sites or loops are required to define such loci, and within regions of what size? This is especially important as FIN loci as identified at this stage then become the focus of the rest of the study (e.g., red highlight in Fig. 4h,j).

(Major) Line 202: It is not clear how the overlap between TAD boundaries and FINs was analysed. Was it perfect overlap only, or overlap allowing for certain shifts? This is important as TAD and boundary calling is highly dependent on what algorithm (e.g., based on switch in contact directionality, or based on local dips of contact density) and which parameters (e.g., bin size) were used.

Fig. 4d: Acute CTCF depletion or degradation is not equivalent to a « KO » (genetic loss of function) condition, and therefore should not be abbreviated as such. Along the same line, control cells should also not be abbreviated « WT ».

Line 228: The statement that « Using NET-seq, Hsieh et al. identified more DEGs than RNA-seq despite shorter time of CTCF depletion (3hr) » is inconsistent with the data presented in Fig. 4e, showing that more DEGs are identified in Nora et al. (213 upreg, 229 downreg) compared to Hsieh et al. (174 upreg, 168 downreg).

(Major) Line 241: The 49 DEGs at FIN loci are only potential or putative direct targets of insulators. Language should be adjusted accordingly.

(Major) Line 256: While the gain in interactions with the distal H3K27me3-marked region might explain Pcdh-gamma gene downregulation, further investigation of the locus, e.g. by deleting the distal H3K27me3-marked region, is required to validate that interactions with the distal H3K27me3-marked region does indeed cause Pcdh-gamma gene downregulation. In the present situation, only an association or coincidence may be claimed, but no cause-and-effect relationship.

Line 272 and Fig. 5c: While the text refers to CUT&RUN of CTCF, the axis label in Fig. 5c indicates CTCF ChIP-seq.

(Major) Line 279: The sentence should describe the gained loop interactions more explicitly: which regions do they connect? What are the characteristics of the connected regions? In addition, the end of the sentence stating that Mesp1 is upregulated « SINCE the new loops bring distal enhancers to its proximity » requires functional validation, typically by targeted deletion of the putative enhancer regions.

Line 281: The sentence should be toned down to « strongly suggest » (that FINs execute their insulating functions in cis) or similar, without further evidence with allele-specific CTCF perturbation and allele-specific gene expression measurements.

DISCUSSION

Line 293: Mentioning that « most TAD boundaries do not gain cross-boundary loops upon CTCF depletion » to contrast them with FINs is highly confusing, because neither do most FINs gain loops in the same condition. Instead, new loops may be gained between other regions that were previously blocked by FINs. This point needs to be clarified to avoid any confusion, especially between FINs and the gained putative E-P interactions.

(Major) Line 304: The sentence is not sufficiently supported by data, as explained in the comment on lines 172-175 above.

Line 306: The sentence describes a very weak and unconvinving evidence for cohesin dependency, through localization of FINs almost essentially in compartment A.

(Major) Without functional validation, E-P loops in this study should be considered as putative, as the enhancers' activity and capacity to regulate the considered genes were not formally assessed.

METHODS

(Major) Line 639: What is meant by « For TADs and their boundaries, we directly use the TADs in mESCs defined by Dixon et al. »? Was the procedure re-used, or the same actual TAD and boundary calls were used? In the latter case, the use of TAD and boundary calls from a different study, Hi-C protocol and mESC line (J1 mouse ES cells in Dixon et al.) could readily explain the poor overlap between FINs and TAD boundaries. To ensure comparability, TADs/boundaries and FINs should be called from the exact same datasets.

(Remarks on code availability)

Reviewer #2

(Remarks to the Author)
Map Functional Insulators with Cross-Platform Hi-C Meta-Analysis

General Thoughts:
Xu et al. characterize functional insulating regions of the genome (termed FINs) that contribute to gene regulation. They begin by analyzing multiple Hi-C and Micro-C datasets involving CTCF, Rad21, or Wapl depletions (Figure 1), using HiCorr and DeepLoop to characterize loop dynamics and identify gained loops upon CTCF depletion. They extend this analysis to

other depletion datasets and compare loop dynamics across CTCF, Wapl, and Rad21 perturbations (Figure 2). FINs are defined insulating regions where de-novo loops form between CREs upon CTCF depletion and are categorized into three types based on CTCF presence (Figure 3). They further link these regions to expression changes and describe new loop dynamics at FIN-containing regions (Figure 4). Finally, they use a dCas9 system to functionally validate selected FINs by targeting CTCF sites and observing transcriptional changes at loci such as Cdiptos and Mesp1 (Figure 5).
This study addresses an important question: why perturbations to chromatin conformation have profound effects in some genomic contexts but not others. While the authors attempt to define FINs in mESCs, the work would benefit from a deeper investigation into what makes an insulating region functional. Additionally, the figures often feel incomplete—more extensive display of the Hi-C/Micro-C matrices would add clarity to the results. Although the authors leverage multiple dataset, the cross-study comparisons are at times underutilized. Overall this work would benefit by further analysis into the FINs they define.

Major comments:
Figure 1: The signal for Rhodes et al. seems very sparse. It would be helpful to show how these regions appear across other datasets, not just in CTCF depletion. Additionally, the general comparisons between datasets are limited to coverage metrics and a few loci. Performing PCA or a similar analysis on the different Hi-C/Micro-C experiments would better contextualize discrepancies between results and substantiate claims about differential depletion across studies.

It's a bit unclear what a pixel represents—I'm assuming one bin? What is the bin size, 5kb? These were then merged into regions down the line, but this is also not clearly explained as to how, or what exactly that means.

I wonder if the longer gained loops being captured in Rhodes et al. (Supp Fig 3 – lines 150) are seemingly longer because the data is too scarce to capture short range loops that might need more coverage to be visible regardless of bias correction or DeepLoop.

In lines 192-196, the term CTCF loop is brought up then switched to just insulation while this neither are really shown, more than just CTCF sites. It would be good to have an example region for each type of loop. TADs are also inconsistently discussed but not shown. Moreover, since TAD-calling can be noisy, more insulation score analysis at FIN vs non-FIN sites could strengthen the work.

This paper generally lacks in depth characterization of FIN's. If there is ATAC-seq, for example, footprinting analysis would be interesting even in just wildtype mESC, to ask if there are any specific motifs or promoter types enriched at these regions which are being functionally insulated in mESCs? Moreover, Are these FINs dynamic across cell types, or stable but functionally relevant only in specific developmental contexts? Some of this is touched on in the discussion but could at least be looked at by analyzing a none-mESC Hi-C or Micro-C dataset.

For Figure 3g: How does a FIN at a non-TAD boundary looks like, is it just because a TAD-call is not there or is it because this region shows no insulation (which is then a bit perplexing). While the schematics are helpful, real data should be shown. I would think that would be much more important for a main figure as a sanity check to the classifications than panel d for example.

Many of the figures need much better background and Supplemental to back up the claims being made. Few examples:
1. A bit more about the enhance models, since these were specific to each experiment.
2. Classification of chromatin loops based on the epigenetic data, it would be helpful to see more examples of loop pixels within each group, and to see what "other" is, is it noisy regions of the genome for example or just regions with no CHIP coverage?
3. A Supplement for Figure 4 could have easily incorporated the Hi-C maps between controls and degradation experiments without DeepLoop models, are they still visible?
4. How was the RNA-seq normalized and how does that affect DEGs. Are ERCC spike-ins better than using empirical genes in RUVseq for example?

Figure 4f isn't clear Also, switching between Nora/Kudo and Hsieh datasets across subpanels (f–j) is confusing.

The functional validation experiments are nice. However, matrix views of the targeted regions should be shown in addition to the DeepLoop model outputs. I think it would be also interesting to do this on a down regulated gene.

Minor comments:
The title needs improvement.

Supplemental Figures 3 and 4 should be made into a single supplementary figure.

Line 180 ("more analysis below") can be deleted.

Figure 4c could be moved to the supplemental section; the plot is confusing and adds limited value.

For Figure 4e, adding NET-seq data next to Hsieh's data and RNA-seq results for other experiments would make comparisons easier.

In Line 232, it's overstated that "most" DEGs overlap between Kudo et al. and Nora et al.—especially considering

upregulated genes.

(Remarks on code availability)
The code is clear and well annotated, the main issue is that the basic descriptions in the paper is lacking.

Version 1:

Reviewer comments:

Reviewer #1

(Remarks to the Author)
All my previous points have been satisfactorily addressed and I recommend the manuscript for publication.

(Remarks on code availability)

Reviewer #2

(Remarks to the Author)
Revision 2
Cross-platform Hi-C meta-analysis identifies functional insulators that actively block enhancer-promoter interactions
Cui, Xu, Lang et al.

Summary:
The authors have added substantial new analysis and some experiments to the work. They have also improved the overall discussion of their results, which initially contained too much over-interpretation with limited support, as also noted by Reviewer #1.
While the manuscript has improved, figure presentation for easier interpretability can still be improved.

Minor Points:
1. Sentence 44-46:
"TADs disappear upon loss.....loop extrusion" could be rewritten to be a bit more readable.
2. Section 116-121
Current text:
"Only the matrices from HiCorr and DeepLoop clustered CTCF- and Rad21-degron datasets together and Wapl-degron datasets are separated out (Fig. 1c, Supplementary Fig. 3). This is consistent with the knowledge that depletion of CTCF or Rad21, but not Wapl, causes massive loss of TADs and CTCF loops. Taken together, these results support the robustness of DeepLoop in cross-platform analysis regardless of read depth."
I think it would be worth noting that both HiCorr and DeepLoop signals appear to saturate toward looping (as one would expect of DeepLoop for example). In contrast, ICE/ICE+distance correction may retain additional or broader structural features, which could explain the lack of clustering/correlation. Clarifying this would strengthen the interpretation.
3. Figure interpretability:
I think a simple heatmap (as in deeptools) of different tracks (H3K27ac, H3K27me3, RNA-seq, etc.) at loop anchors of FINs (perhaps even separated by class type) could be useful to visually summarize the features associated with dynamic loops.
I appreciate the new analysis in Supplemental Figure 1. I do think a simple heatmap showing insulation at gained, lost, and maintained anchors would be interesting. This could help clarify whether these are regions that are already prepped but blocked, or if it's a mix of multiple dynamics including de novo gain of regulation.
I would suggest using anchors rather than merged regions, as otherwise there may be too few regions and mixed sizes - but not sure.
4. Figure Organization:
Figure 1: I would move Nora et al. to the bottom to be closer to the tracks in panel e.
Figure 2: Could incorporate the panels currently in Supplemental Figure 6. This would clarify and simplify interpretation for the reader.
I would consider including EP loops, H3K27me3 loops, and CTCF loops, with matrices of treatment/depletion overlaid.
I think the manuscript generally lacked showing the underlying data. While this has improved, there is still room to expand this further.
5. The Pcdh locus is interesting - could this be due to mis-regulation of Polycomb domain?
6. Given the targets in the motif enrichment, I’m curious to whether any of these factors are differentially regulated upon CTCF/Rad depletion – in the depleted anchors for example, there is a considerable amount of homeobox/homeodomain containing factors.

(Remarks on code availability)
I have partially looked at the code, but I have not re-run or done in depth line by line review.

Open Access This Peer Review File is licensed under a Creative Commons Attribution 4.0 International License, which permits use, sharing, adaptation, distribution and reproduction in any medium or format, as long as you give appropriate credit to the original author(s) and the source, provide a link to the Creative Commons license, and indicate if changes were made.
In cases where reviewers are anonymous, credit should be given to 'Anonymous Referee' and the source.
The images or other third party material in this Peer Review File are included in the article's Creative Commons license, unless indicated otherwise in a credit line to the material. If material is not included in the article's Creative Commons license and your intended use is not permitted by statutory regulation or exceeds the permitted use, you will need to obtain permission directly from the copyright holder.
To view a copy of this license, visit https://creativecommons.org/licenses/by/4.0/

We sincerely thank both reviewers for their time and efforts. Their constructive suggestions have significantly improved our manuscript. As detailed below, we have added new experiments, new data analysis, new figures, and modified the text to strengthen our points and clarify our findings. We believe we have addressed all reviewer's comments and concerns. The following are the major changes:

- A new title.
- One new validation experiment (**Fig. 4k,l**) in which we show downregulation of Pcdh-γ genes upon deletion of a putative functional insulator (FIN).
- A new **Supp. Fig. 1** in which we compared the compartment A/B and TADs across three CTCF-degron Hi-C datasets.
- A new section in the main text with new figures **Fig. 1b-c** and **Supp. Fig. 2-3** demonstrating that DeepLoop achieves best consistency in cross-platform Hi-C loop analysis.
- A comprehensive TAD-boundary analysis using control mESC data from all three CTCF-degron study with both "Directionality index" and "Insulation score" measurement. We now have more convincing results showing that regardless of datasets or algorithm, low resolution TAD-level features cannot represent the FINs we identified from high-resolution dynamic loop analysis. These results are shown in the new **Fig. 3f,h,l** and **Supp. Fig. 11**.
- An interesting new motif analysis results in **Fig. 3l** showing that G-rich cis-regulatory elements are more likely to act as new loop anchors upon CTCF-depletion.
- A new **Supp. Fig. 8** in which we show two examples that although Rhodes *et al.* showed gain of long-range interactions between H3K27me3-occupied regions upon Rad21-depletion, the long-range H3K27me3 loops already exist in the control cells from Kriz *et al.* and Hsieh *et al.* before *Rad21*-depletion. This finding argues against the conclusion of Rhodes *et al.* that cohesin prevents the formation of long-range H3K27me3 loops.
- We also added many new contact heatmaps or examples as the reviewers requested in **Fig. 5b,e**, **Supp. Fig. 4g,h**, **Supp. Fig. 6**, **Supp. Fig. 10**, **Supp. Fig. 12**.

In the main text file, we have highlighted major changes in yellow. All changes are also explained in the point-by-point responses to the reviewers' comments. The reviewer's comments are in black and our response in blue.

Reviewer #1 (Remarks to the Author)

Summary:

The paper entitled « Map functional insulators with cross-platform Hi-C meta-analysis » by Wanying Xu and colleagues describes a new computational approach to analyze chromatin conformation data, in particular Hi-C and micro-C across platforms, identifies putative functional insulators in the genome using this approach, and validates insulator loci experimentally at selected loci. The computational approach used for cross-platform data analysis primarily relies on the DeepLoop pipeline in order to identify loop interactions that are more robust across assays than using conventional methods such as ICE normalization. Applied on datasets from two published Hi-C studies and one micro-C study, DeepLoop identified retained and differential (lost or gained) loop interactions between control conditions and conditions in which either the mammalian insulator protein CTCF, the cohesin subunit RAD21, or the cohesin unloading factor WAPL were depleted. The differential loops are mostly consistent across assays for given conditions, and a subset of loops gained upon CTCF depletion bring together loop anchors that are characterized by epigenetic features associated with promoters and enhancers (cis-regulatory elements), specifically H3K4me3 and H3K27ac. The study hypothesizes that these loops represent newly established enhancer-promoter contacts, which were previously prevented or « blocked » by nearby CTCF-mediated contact points, and proposes the latter contact points as functional insulators (FINs). The study proposes a model of FIN function dependent on loop extrusion, whereby cohesin-mediated contacts between CTCF-bound anchor points at FINs prevent contacts and regulatory communication between cis-regulatory elements (e.g., an enhancer or group of enhancers, and the promoter of the target gene) that are separated by such FIN elements. Differentially expressed genes (DEGs) are enriched at gained loops and the DEGs that are most reproducible between studies tend to be located near FINs. Detailed examination at selected loci including Cdiptos and Pcdh-gamma shows loss of FIN-anchored interactions upon CTCF depletion that coincides with gain in interaction between distal H3K27ac-marked regions and promoter-proximal regions of DEGs. Interfering with CTCF binding using sgRNA/dCas9 at specific CTCF sites in selected candidate FIN regions result in a similar impact at the targeted loci than global CTCF depletion, including gene upregulation and gain of distal interactions of DEGs with H3K27ac-marked regions, thus validating the role of CTCF in the function of these specific FINs. The results of the study suggest that FIN are cis-acting insulators of enhancer-promoter communication in untreated cells.

Overall assessment:

The findings of the study are important for the fields of 3D genome and gene regulation, and could also have a broad impact on the scientific community in general, however there are major concerns with the presentation, analysis and interpretation of the results in the current state of the manuscript. At multiple points throughout the manuscript, some results are over-interpreted, hypotheses are not sufficiently tested to support the conclusions, and there is a lack of consideration for equally plausible alternative explanations to some of the findings. Cause-and-effect implications are often prematurely drawn in the absence of the required functional validation, resulting in missing evidence to support some of the main conclusions of the paper. In addition, the manuscript lacks essential yet basic analyses, such as TAD and compartment calling in the first section, misses or provides only cryptic informations about important analysis parameters (e.g., it is unclear how the overlap between TAD boundaries and FINs was assessed, and the main text lacks basic information about which epigenetic features were considered to identify putative E-P loops). For these reasons, I advise a major revision of the manuscript according to the point-by-point comments listed below, organized in chronological order with respect to the main text.

We really appreciate the reviewer’s comprehensive summary and careful review of our work. We also thank the reviewer for recognizing our work’s potential to have “a broad impact on the scientific community in general”. Below we have carefully addressed the reviewer’s point-by-point comments. Following the reviewers’ suggestions, we have added new experiments and analyses into the revised manuscript.

Point-by-point comments:

RESULTS

(Major) Line 78: The statement that « for Hi-C and in situ Hi-C studies, conventional ICE-normalization only support compartment- or TAD-level analysis at 20kb or lower resolution due to the lack of read depth » lacks supporting evidence from TAD and compartment calling.

We have performed compartment- and TAD-level analysis using conventional pipelines on the three CTCF-AID datasets. The results are shown in a new **Supp. Fig. 1**.

At compartment level (500kb resolution), in all three datasets the contact heatmaps and PC1 profiles (compartment A/B) are largely unchanged after CTCF-depletion (**Supp. Fig. 1a**), and this conclusion is further supported by scatter plots of PC1 values showing the high correlation between control and △CTCF cells within each of the studies (R-square 0.820~0.907, **Supp. Fig. 1b**). These results indicate that the compartmental organization is largely preserved after CTCF depletion, consistent with the original studies. In cross-platform comparisons, we found that the heatmaps are quite similar although the R-square values are slightly lower (0.763~0.791 for control cells and 0.777~0.799 in △CTCF cells, **Supp. Fig. 1c**). These results support the robustness of compartment analysis at low-resolution across different Hi-C protocols.

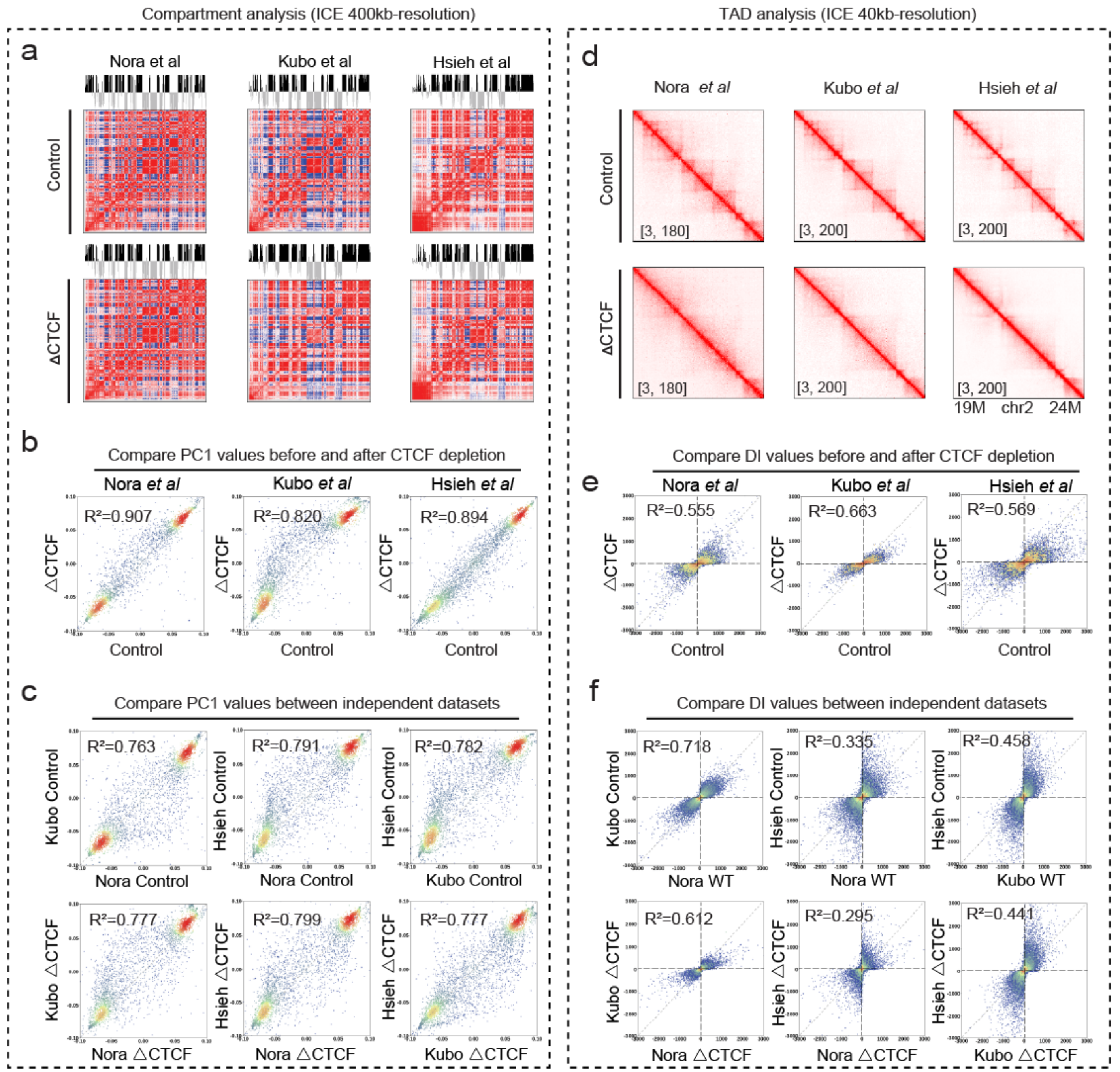

Supp. Fig. 1. Compartment and TAD analysis of the three independent CTCF-depletion studies.

At the TADs level (40kb resolution), the loss of TADs upon CTCF depletion is also obvious in all studies (**Supp. Fig. 1d**), consistent with the original reports. Loss-of-TAD is also reflected in scatter plots of DI (directionality index) of all 40kb bins, in which the DI values of many bins become close to zero upon CTCF-depletion (**Supp. Fig. 1e**). The heatmaps of Nora et al. and Kubo et al. are slightly noisier than Hsieh et al. (**Supp. Fig. 1d**). We also compare the DI values between independent HiC/in situ Hi-C/micro-C studies (**Supp. Fig. 1f**): although the R-squares between the Hi-C and in situ Hi-C (Nora et al. and Kubo et al.) are rather high (R-square 0.718 and 0.612 for control and △CTCF cells), the DI values from micro-C data (Hsieh et al.) deviate significantly from the other two studies (R-square 0.295~0.458). In micro-C data, more bins show polarized DI values indicating stronger directionality biases. This is consistent with the general belief that with ICE-based pipeline, micro-C identifies more loops than Hi-C or in situ Hi-C.

Taking together, these analyses confirmed the lack of compartment changes and the loss of TADs upon CTCF-depletion. However, the commonly used scores (PC1 or DI) from standard ICE pipelines show variation between independent studies. The platform variation in TAD-level analysis is higher than compartment level analysis.

At loop level (5kb resolution), the variation between independent studies is much bigger. We will discuss below in response to the reviewer's next comment.

(Major) Line 83: In addition to visual inspection at selected examples of chromosomal regions, the robustness of DeepLoop in cross-platform analysis should also be supported by correlating the *DeepLoop* signal (e.g., pixel intensity) for the same region pairs between assays, and by contrasting this correlation to the those obtained with standard ICE normalization or HiCorr correction only.

We thank reviewer for the suggestion. We added three new figures in the revision demonstrating this point:

A. Supplementary Figure 2.

We added a massive new **Supp. Fig. 2** to compare the performance of ICE, ICE with distance correction, HiCorr, and DeepLoop in quantifying loop strength with all datasets. Part of **Supp. Fig. 2** (only for CTCF-degron datasets) are shown below. In this figure we included: (i) example heatmaps at 5kb-resolution; (ii) scatter plots comparing control cell heatmaps from independent studies to evaluate each pipeline's consistency in cross-platform analysis; (iii) scatter plots comparing each pair of heatmaps before and after protein depletion. For (ii) we only compare control cell data because before protein depletion, the control cells from all studies are expected to be similar at the stem cell state. Protein-depleted cells from independent studies are biologically more diverse due to many reasons such as time-span of protein depletion, incomplete protein depletion, loss of stemness, etc.

When resolution increases to 5kb, the contact heatmaps from ICE (with or without distance correction) and HiCorr are highly variable between independent datasets. There are more noises in Hi-C and *in situ* Hi-C heatmaps than in micro-C heatmaps (**Supp. Fig. 2a,d,g**). However, the heatmaps from *DeepLoop* are much more consistent between platforms because DeepLoop enhances loop pixels and removes most of the noise pixels (**Supp. Fig. 2j**).

For cross-platform consistency analysis in control cells, we found that although conventional ICE pipelines show poor cross-platform consistency with R-square values often close to zero, HiCorr and DeepLoop clearly improved the consistency with much higher cross-platform R-square values (**Supp. Fig. 2b,e,h,k**).

The comparisons between control and degron cells also support the cross-platform consistency. For example, if we use ICE without distance-correction to analyze the micro-C data by Hsieh *et al.*, △CTCF cells have many pixels stronger than control cells (**Supp. Fig. 2c** below), which is opposite to the knowledge that CTCF-depletion leads to loss of loops. This is because the read-depth of micro-C data in △CTCF cells is higher than in control cells (830M vs. 291M). If we perform distance correction after ICE-normalization, which generates observed/expected ratio heatmaps. The control vs. △CTCF scatter plot for Hsieh *et al.* correctly shows more loop pixels in control cells (**Supp. Fig. 2f**). However, the scatter plots from ICE-based pipelines lack pixels that retain strength upon CTCF-depletion, which is in sharp contrast to HiCorr and DeepLoop scatter plots, in which the retained pixels are clearly recognizable (**Supp. Fig. 2i, l**).

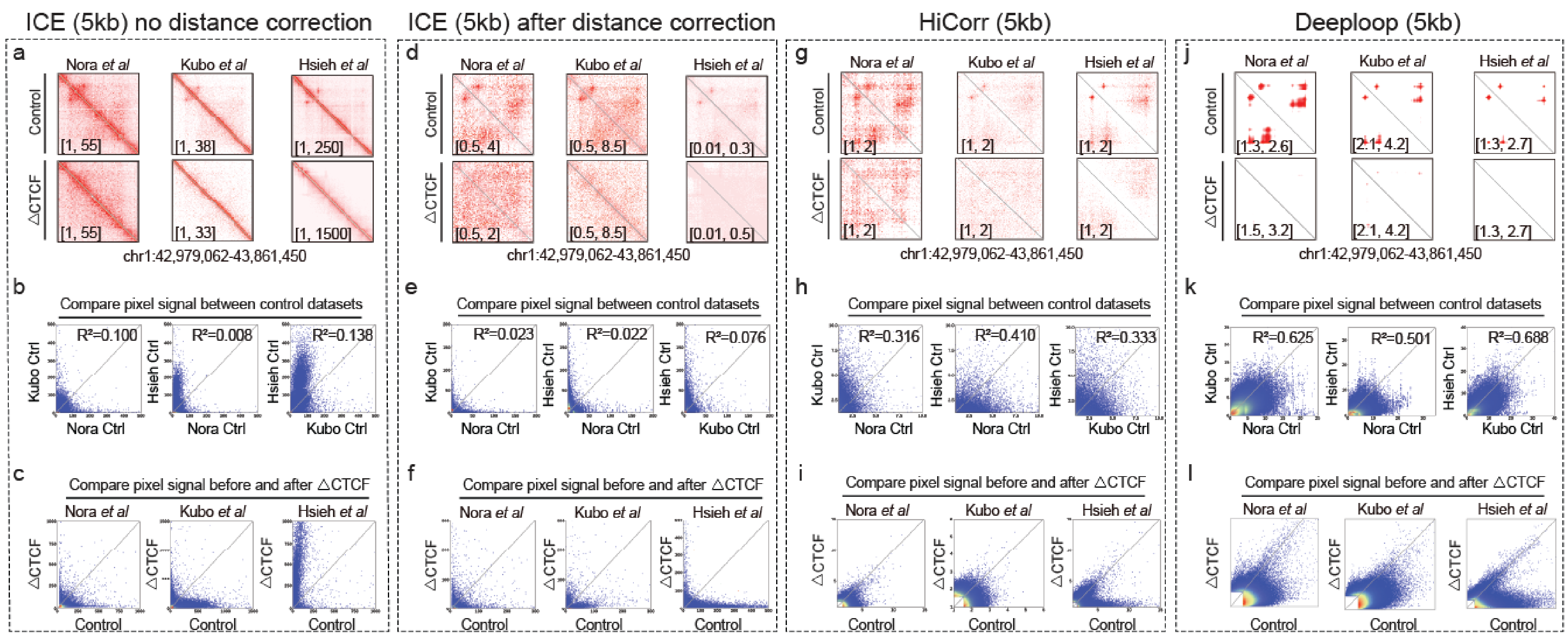

Supp. Fig. 2. Loop-level analysis (5kb resolution) of the three CTCF-degron studies with ICE (with or without distance correction), HiCorr, and DeepLoop.

B. Figure 1b.

In the main figure, we also added **Fig. 1b** (shown on the right) to summarize the results about cross-platform consistency as discussed above in **Supp. Fig. 2**. We found that when applying different pipelines to control mESC data from all datasets, DeepLoop achieves the best cross-platform consistency between orthogonal contact heatmaps.

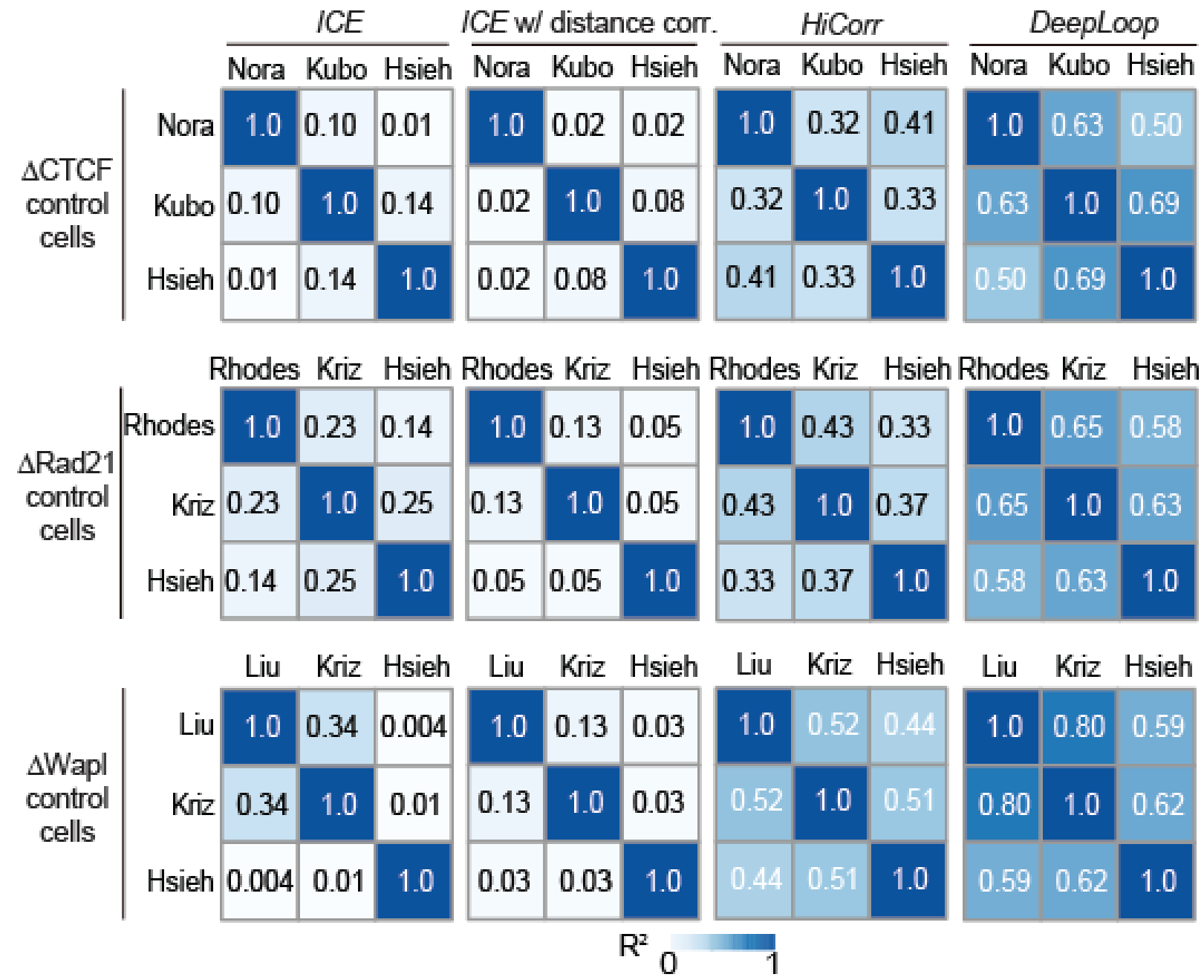

Fig. 1b. R-square matrices comparing Hi-C heatmaps in control mESC cells from different datasets when analyzed by different pipelines.

C. Supplementary Figure 3 and Figure 1c.

We also added a new **Supp. Fig. 3** showing the cross-platform consistency when applying different pipelines to analyze dynamic loops. In this analysis we first generate differential heatmaps at 5kb resolution by subtracting each pair of contact heatmaps (depletion - control), then compute the correlations between the differential heatmaps from all nine pairs of degron experiments. Only *HiCorr* and *DeepLoop* cluster CTCF- and Rad21-degron datasets together, and Wapl-degron datasets are separated (**Supp. Fig. 3**). This is consistent with the knowledge that depletion of either CTCF or Rad21, but not Wapl, causes massive loss of TADs and CTCF loops. **Fig. 1c** in the main figure includes two panels of **Supp. Fig. 3** (panel **a** and **d**).

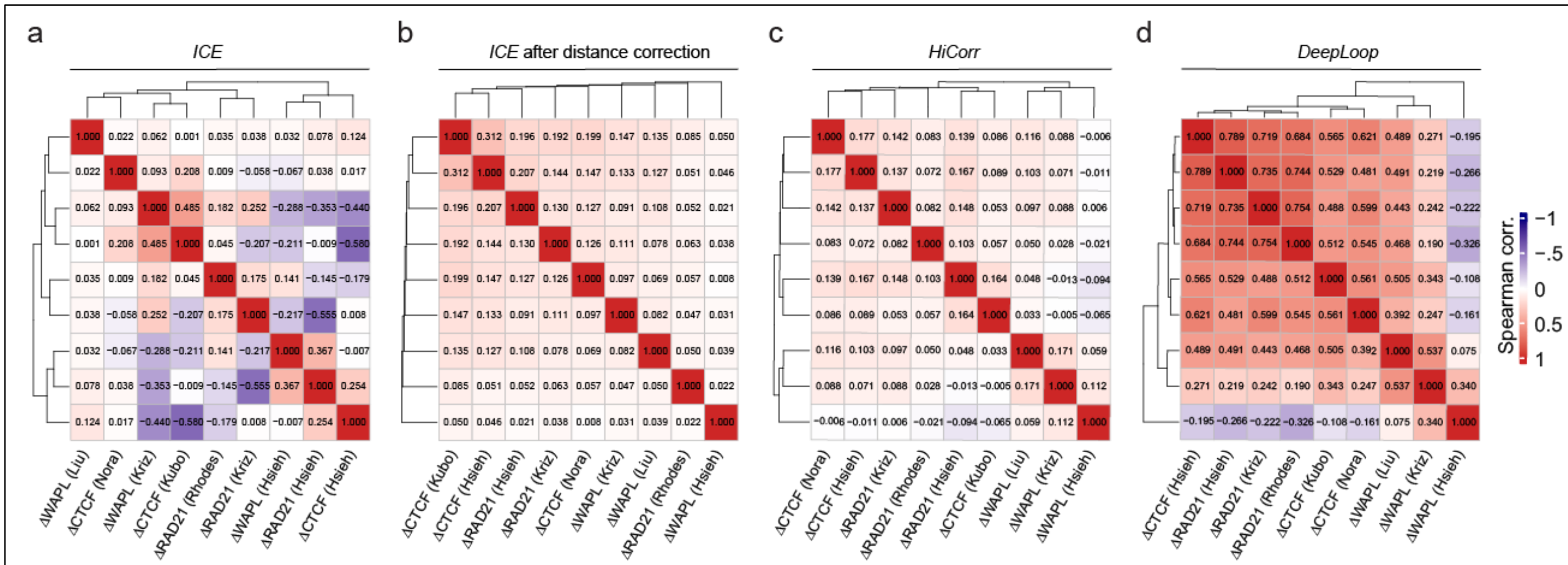

Supp. Fig. 3. Correlation matrices comparing the differential contact heatmaps at 5kb resolution from all nine pairs of degron experiments.

Taking together, all these results support the robustness of DeepLoop in cross-platform analysis.

Fig. 1d: Y-axis labels should indicate « loop strength » after « Control » and « deltaCTCF-IAA » conditions.

The old **Fig. 1d** is now **Fig. 2a**.

We have updated the labels in **Fig. 2a** and other figure panels as the reviewer suggested.

(Major) Fig. 1e,f: The epigenetic features of promoter and enhancers used to define E-P loops should be summarized directly in the result section. This information is currently only present in the method section.

The old **Fig. 1e,f** is now **Fig. 2b,c**.

We have now summarized the epigenetic features used to define the E-P loops in the results section as the reviewer suggested. We also added a new **Supp. Fig. 6** showing examples of EP-loop, CTCF-EP loop, CTCF loop, H3K27me3 loop, and H3K36me3 loop.

For contact heatmaps, the meaning of the numbers between brackets should be indicated in the figure legend.

We have revised the figure legend for the contact heatmaps to clarify the meaning of the numbers shown in brackets, which refer to the scale of heatmaps.

For plots of loop size, the figure legend should explicitly indicate that the median loop size is given underneath the plot for each loop categories.

We have revised the figure legend of **Fig. 2a** to explicitly state that the median loop size is shown below each plot for the corresponding loop category.

Line 121: For more clarity, modifying the sentence as follows is recommended: « some of them lose strength [upon CTCF degradation] in Hsieh et al. …»

We have modified this sentence as recommended (line 160-161).

(Major) Line 126: The data presented are not sufficient to conclude that « Hsieh et al. achieves the best CTCF depletion, perhaps because it uses N-terminal degron system ». Assay-specific biases, differences in sensitivity and in the depth of sequencing could also explain at least some of the discrepancies observed between studies.

We agree and have revised the text to tune down the conclusion and acknowledge these potential confounding factors (line 166-169)

Line 132: For more accuracy, modifying the sentence should be modified as follows « Nevertheless, [most of] the "gained pixels" from Nora et al. … ».

We have revised this sentence as recommended (line 174).

Line 138: It is not clear what is meant by « affected genes … are likely to be the direct targets of insulators ». How/why are the affected genes likely to be the targets of insulators?

We have revised this statement down to:

*"…affected genes such as Notch1 in **Fig. 1** are putative target genes of CTCF-mediated insulation."* (line 179-180)

(Major) Line 143: About the observation that « "retained" and "gained" loop pixels in Rad21-depleted cells are much smaller (mostly within 100kb) than those in CTCF-depleted cells, indicating that cohesin-independent loops are short-range », it is also conceivable that decreasing the pool of available cohesin through depletion of RAD21 allows for only rare and short-lived extrusion events (assuming unchanged levels of the cohesin unloader WAPL

upon RAD21 depletion compared to untreated cells), thereby creating smaller-sized, yet still cohesin-dependent loops. The above possibility should be acknowledged in the manuscript.

We agree and thank the reviewer for the insights. In the revision we have now acknowledged this alternative explanation. (line 189-191)

Line 147: For accuracy, the following changes are recommended: « The “retained” and “gained” loop pixels in Kriz et al. and Hsieh et al., and “retained” loop pixels in Rhodes et al. In Rad21-depleted cells… »

We thank the reviewer for the suggestion and have revised the sentence to exclude the “gained” loop pixels in Rhodes et al. (line 186-187)

Line 151: It is not clear what is « the conclusion from the original study ».

We apologize for the ambiguity and have revised this sentence to explicitly state the conclusion of Rhodes *et al.*: “…cohesin removal enhances long-range interactions between a subset of polycomb-occupied regions…” (line 198-199)

We want to add that in this revision we have examined the “gained” H3K27me3 loops in Rad21-depleted cells from Rhodes et al. and found that in almost all cases, the “gained” long-range H3K27me3 loops are already present in the control cells from Kriz *et al*. and Hsieh *et al.* before Rad21 depletion (two examples are shown in a new **Supp. Fig. 8**). Therefore, at least in the control mESCs from Kriz et al. and Hsieh et al., cohesin does not prevent the formation of long-range H3K27me3 loops.

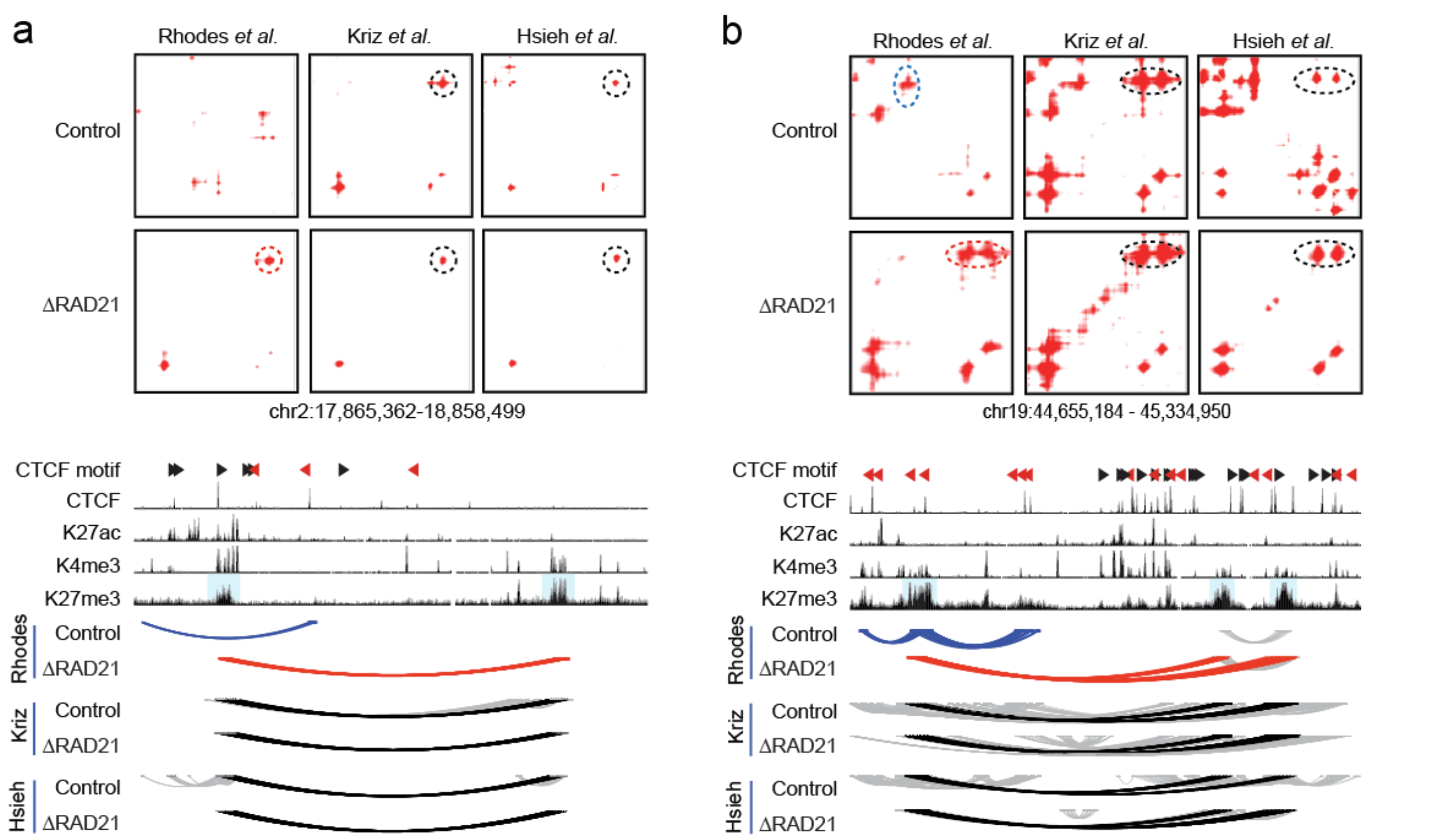

Supp. Fig. 8. “Gained” long-range H3K27me3 loops in Rad21-depleted cells from Rhodes et al. exist before Rad21-depletion in two other studies.

(Major) The use of the terms « dependent » and « independent » (e.g.,whole paragraph starting line 165) with respect to CTCF or cohesin should be avoided given the possibility that residual amount of these factors could

be present in the depleted cells, and the observation that, for example, some loops retained upon CTCF depletion display CTCF peaks at both anchors (44% of retained loops in Nora et al.) and are hence likely to be CTCF-dependent. The use of the terms « retained », « gained » or « lost » upon certain conditions should be favored for accuracy.

We appreciate the suggestion and have refrained the usage of "dependent" and "independent" in the revision.

(Major) Lines 172-175: The conclusion that « recurrently gained E-P interactions upon CTCF depletion are created by loop extrusion » because « "gained" loops in CTCF-depleted cells do not gain strength in Rad21-depleted cells » seems far-fetched, because these loops also do not show clear loss of strength upon RAD21 depletion (or this result is not properly presented and discussed, which would be expected if they are created by loop extrusion.

Both CTCF- and Rad21-depletion cause massive loss of TAD structure and nearly all CTCF loops. In this work, we argue that only CTCF-depletion also leads to recurrent gain of a small number of EP loops.

These "gained" loops in CTCF-degron data are expected to absent in all control cells, including both the control cells from CTCF-degron studies and the control cells from Rad21-degron studies. If these "gained" pixels in ΔCTCF cells are cohesin-dependent, they should remain weak in ΔRad21 cells, which is what we observed.

Here the reviewer argues that we cannot tell for sure those gained loops are created by cohesin unless we can see them getting lost upon Rad21 depletion. With current datasets we cannot test this because these loops do not exist in Rad21-degron control cells; we cannot test if these loops lose strength upon Rad21 depletion if they do not exist before Rad21 depletion. The only way to test this is to deplete CTCF first, wait till the cells gain the new loops, then deplete Rad21 and check if the gained loops disappear. This will require a cell line in which we can deplete both CTCF and Rad21 sequentially. Unfortunately, we do not have such a cell line which is beyond our current capability and the scope of this paper, but this is certainly an interesting experiment for the future.

On the other hand, we also observed that many of the "gained" loop pixels in ΔCTCF cells also gain strength in Δ*Wapl* cells (**Fig. 2d**, last row and **Supp. Fig. 9**, last row). This is further supported by the results in **Fig. 3d**, in which we found a significant fraction of loop gaining events also occurs in Wapl-depleted cells but almost none in Rad21-depleted cells. These observations in Wapl-degron data, together with the results from Rad21-degron data, suggest that cohesin plays a role in the formation of the "gained" loops in ΔCTCF cells: Rad21-depletion disables the loop formation and Wapl-depletion allows loop formation.

Regardless, we have revised the text to tune down the statement and reflect on the discussions above:

"…the "gained" loops in CTCF-degron studies, which are weak in both CTCF-degron and Rad21-degron control mESCs, do not gain strength upon Rad21-depletion cells, suggesting that the formation of these "gained" loops requires cohesin. In contrast, we observed that these "gained" loops in CTCF-depleted cells showed an apparent upward bias in the scatter plots of Wapl-degron studies. This observation…further supports a role of cohesin in forming those "gained" loops…" (line 232-238)

Line 177: « Supp Fig. 5a, b, third row » is mislabelled as « Supp Fig. 5b, d, third row ».

We thank the reviewer for pointing out this issue and have corrected these typos in line 236.

(Major) Fig. 3b: It is unclear how the FIN loci were identified, e.g. how many CTCF sites or loops are required to define such loci, and within regions of what size? This is especially important as FIN loci as identified at this stage then become the focus of the rest of the study (e.g., red highlight in Fig. 4h,j).

We apologize for the confusion. In this study we defined a few names: "loop-gaining locus", "FIN", and "FIN locus".

Loop-gaining locus is defined from "gained" loop pixels in CTCF-depleted cells. We identified thousands of "gained" loop pixels and each pixel represents a pair of 5kb-bins. However, these pixels often occupy overlapping regions. We define loop gaining loci for the convenience of unique counting. In **Review Fig. R1** on the right, we show three "gained" loops in ΔCTCF cells and the loop gaining locus is the entire region covered by all three arches. In our study, we have ~300 FIN loci after merging ~5,000 recurrent "gained" loop pixels.

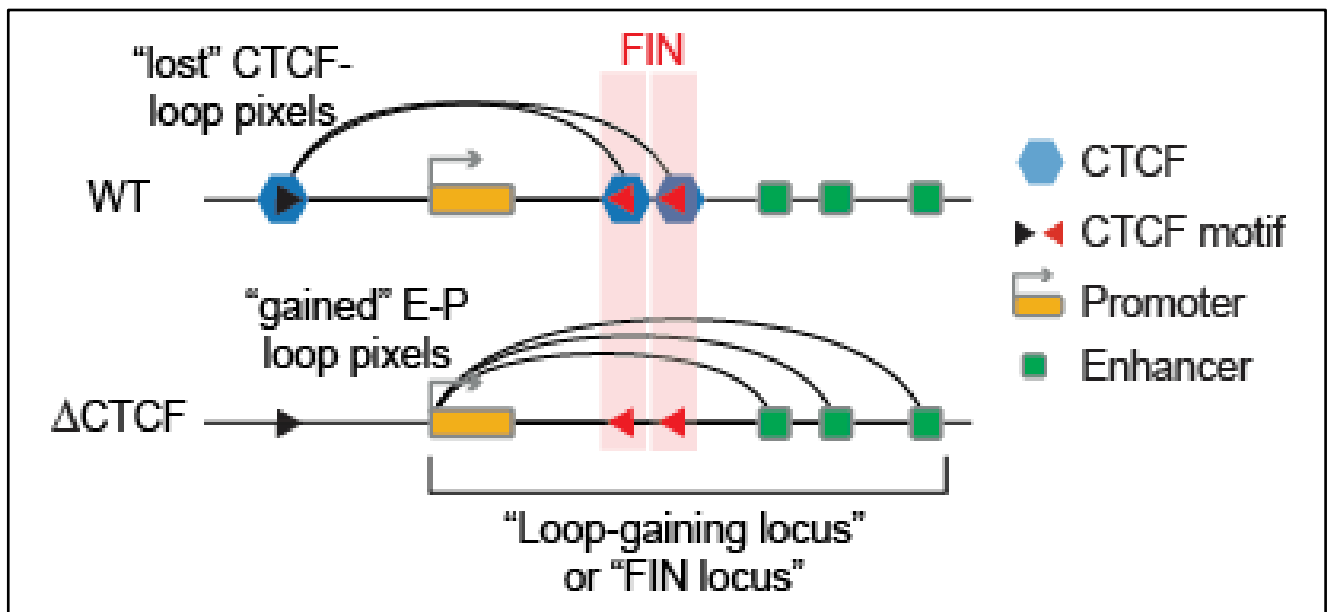

Review Fig. 1. Scheme to define Loop-gaining locus, FIN locus and FIN.

FINs are CTCF binding sites that satisfy the following criteria (highlighted in pink in **Reviewer Fig. 1**):
(i) Located at the "lost" loop anchors in CTCF-depleted cells.
(ii) Located inside a newly "gained" loop in CTCF-depleted cells.
(iii) The other anchor of the "lost" CTCF loop is outside of the newly "gained" loop.
(iv) CTCF sites satisfying (i-iii) within 20kb of each other will be merged into one FIN. We do not have limits on the number of CTCF sites to define FINs. Therefore, although a FIN can be just one single CTCF site, most FINs have multiple CTCF sites.

FIN loci. As described above, all FINs must be inside loop-gaining loci. "FIN loci" are "loop-gaining loci" that containing FINs. In fact, almost all the "loop-gaining loci" are also "FIN loci".

We have modified the text to clarify the definition and added more detailed description in the **Methods** section.

(Major) Line 202: It is not clear how the overlap between TAD boundaries and FINs was analyzed. Was it perfect overlap only, or overlap allowing for certain shifts? This is important as TAD and boundary calling is highly dependent on what algorithm (e.g., based on switch in contact directionality, or based on local dips of contact density) and which parameters (e.g., bin size) were used.

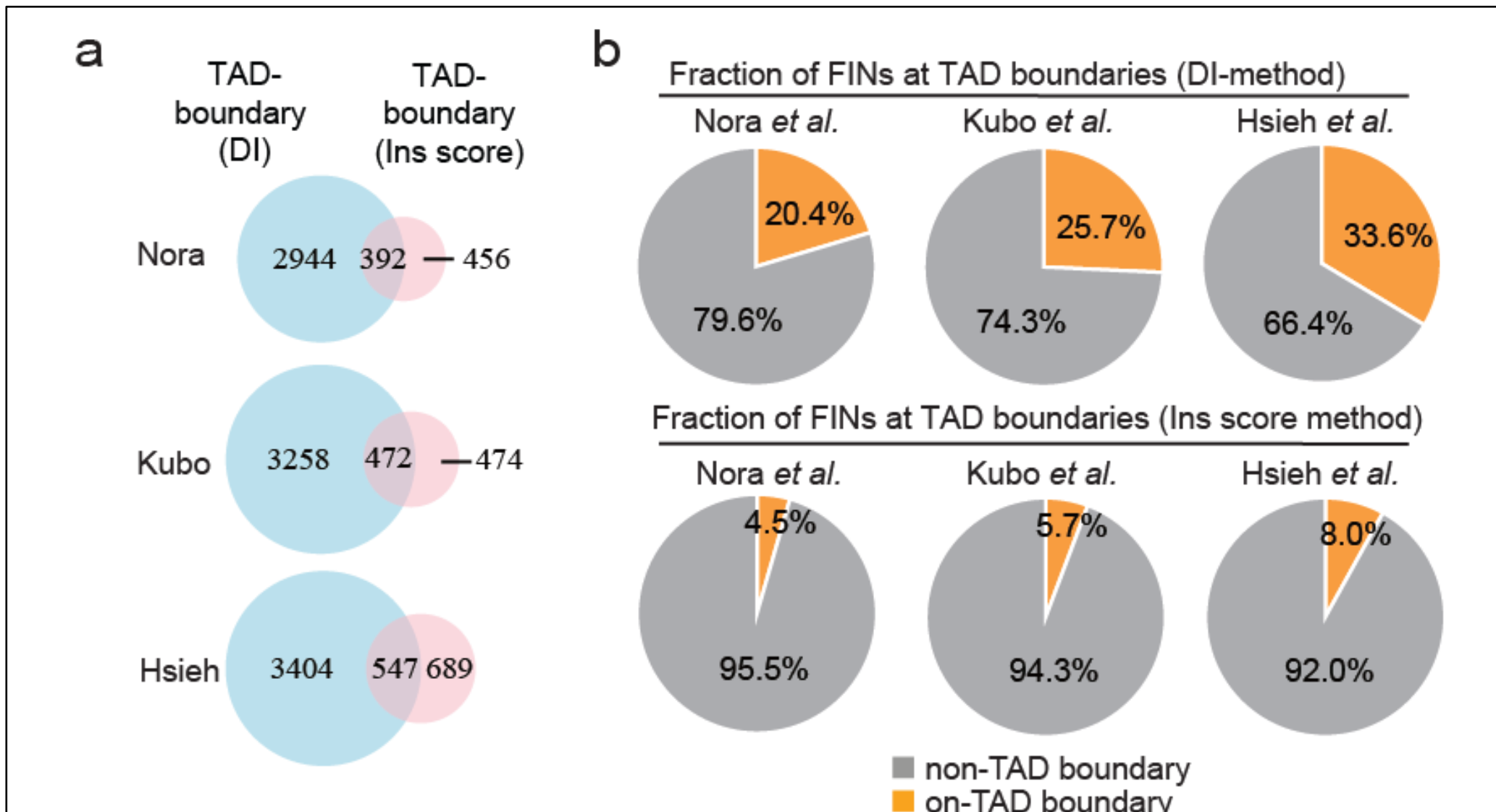

Supp. Fig. 11. (a) Compare the overlap between TAD boundaries called from DI and Insulation score. **(b)** Pie chart showing the percentage of FINs that overlap TAD boundaries called from different algorithms.

We defined overlapped FINs and TAD boundaries if they are within 40kb of each other. Therefore, most of the FINs are at least 40kb away from TAD boundaries. This information has been added to **Methods**.

Our previous version directly used the TADs in mESCs defined by *Dixon et al.* (PMID: 22495300) and concluded that only a small fraction of FINs overlaps TAD boundaries. In this revision, we now directly call TAD boundaries from the three datasets at 40kb resolution with 2Mb sliding window using two methods: (i) "Directionality index" (DI) -based method described by Dixon et al.; (ii) "insulation score"-based method (Crane et al., PMID: 26030525) using *Cooltools*. We added a new **Supp. Fig. 11** in this revision.

Indeed, as the reviewer commented, boundaries calling is highly dependent on the data and algorithm. DI-method calls many more TAD boundaries than InsScore method. The number of TAD boundaries also tends to

be higher when using more frequent Hi-C cutters (MicroC > in situ Hi-C > Hi-C) (**Supp. Fig. 11a**). As expected, the overlap between TAD boundaries and FINs increases if more TAD boundaries are called but regardless of the method of choice, only a fraction of FINs overlapped with TAD boundaries (**Supp. Fig. 11b**).

In this revision we also added a new **Fig.3f** with a FIN example (highlighted in pink) that is not at TAD boundary. In this figure we also included the tracks for "insulation score" and "directionality index". The two TAD boundaries in this example reach a local minimum of InsScore but the FIN does not. Similarly, using DI-based method, TAD boundaries are defined as locations where DI changes sign, but in this example, the FIN is located inside TAD, and its DI does not change sign.

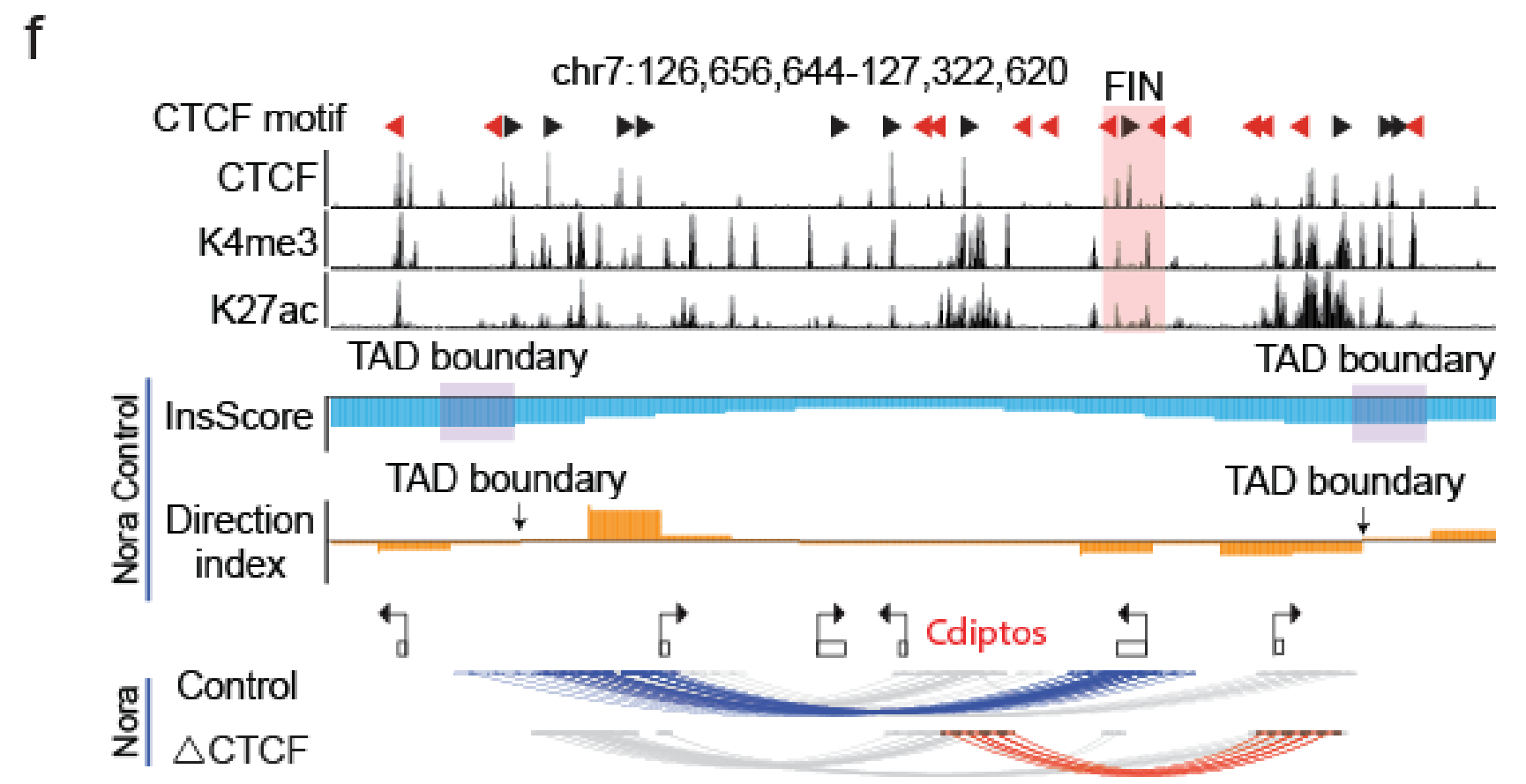

Fig. 3f. An example of non-TAD boundary FIN.

Fig. 4d: Acute CTCF depletion or degradation is not equivalent to a « KO » (genetic loss of function) condition,ot be abbreviated as such. Along the same line, control cells should also not be abbreviated « WT ».

We agree and thank the reviewer for pointing this out. We have revised all figures and text using "control" instead of "WT" and using "CTCF-depletion" or "ΔCTCF" instead of "KO" to indicating protein depletion.

Line 228: The statement that « Using NET-seq, Hsieh et al. identified more DEGs than RNA-seq despite shorter time of CTCF depletion (3hr) » is inconsistent with the data presented in Fig. 4e, showing that more DEGs are identified in Nora et al. (213 upreg, 229 downreg) compared to Hsieh et al. (174 upreg, 168 downreg).

We apologize for the confusion.

This statement was intended to compare the NET-seq and RNA-seq reported by Hsieh *et al.* upon 3-hour CTCF depletion. With 3-hour depletion, RNA-seq data from Hsieh *et al.* has 57 upregulated and 45 downregulated DEGs, while NET-seq data has 174 upregulated genes and 168 downregulated DEGs.

To make it clearer, we have revised the text to (line 319-324):

"…Hsieh et al. performed both NET-seq and RNA-seq after only 3hr CTCF-depletion, which should include fewer late-response genes, but the short treatment time may also reduce the sensitivity of DEG detection. We use NET-seq data because Hsieh et al. reported that NET-seq has higher sensitivity in identifying DEGs as it maps newly synthesized RNA, while RNA-seq measures the amount of steady state RNA in the cells…"

(Major) Line 241: The 49 DEGs at FIN loci are only potential or putative direct targets of insulators. Language should be adjusted accordingly.

We agree and have adjusted the language here as "putative direct targets" (line 335).

(Major) Line 256: While the gain in interactions with the distal H3K27me3-marked region might explain Pcdh-gamma gene downregulation, further investigation of the locus, e.g. by deleting the distal H3K27me3-marked region, is required to validate those interactions with the distal H3K27me3-marked region does indeed cause Pcdh-gamma gene downregulation. In the present situation, only an association or coincidence may be claimed, but no cause-and-effect relationship.

We thank the reviewer for the comments. In our manuscript we reported:

*"…A cluster of putative enhancers marked by H3K27ac is present at the 3' of γc promoters (**Fig. 4j**, highlighted in green), forming loop interactions with the promoters of γa's and γb's (**Fig. 4j**, blue arches), which are restricted by a FIN with multiple CTCF peaks (**Fig. 4j**, highlighted in pink). Upon CTCF depletion, the H3K27ac-interactions are weakened, and the Pcdh-γ promoter loops extend into a distal H3K27ac-poor region marked by H3K27me3, which explains the downregulation of γa and γb genes..."*

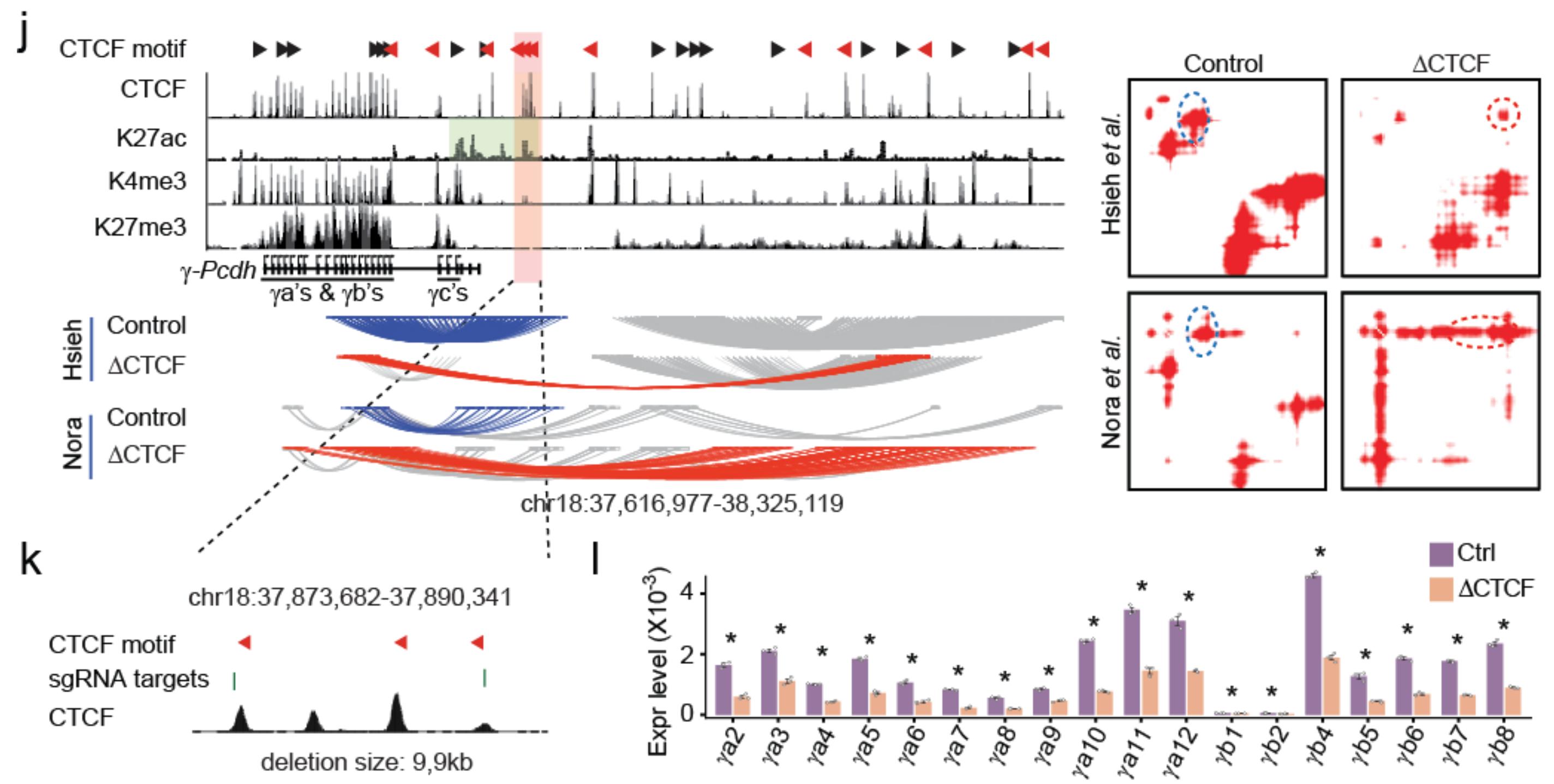

Fig. 4j,k,l. (j) Left: browser tracks near *Pcdh-γ* locus; right: Hi-C heatmaps from two CTCF-degron studies. **(k)** Design of sgRNAs for insulator deletion. (**l**) RT-qPCR shows the down-regulation of *Pcdh-γ* genes in mESCs after insulator deletion.

Here we reported two events upon CTCF depletion: loss of nearby enhancer loop and gain of distal H3K27me3 loop. Both are consistent with the downregulation of γa and γb genes. To avoid making causal inferences, in the revision we have rephrase from "…which explains the downregulation of γa and γb genes…" to "…consistent with the downregulation of γa and γb genes…" (line 351)

Additionally, we added new **Fig.4k,l** showing that deletion of the putative insulator indeed leads to down-regulation of γa and γb genes. However, we still cannot tell if the loss-of-enhancer-loop or gain-of-H3K27me3-loop (or both) causes the transcription changes, which require more extensive experiments.

Line 272 and Fig. 5c: While the text refers to CUT&RUN of CTCF, the axis label in Fig. 5c indicates CTCF ChIP-seq.

We thank the reviewer for picking up this typo and have updated the figure labels to CUT&RUN.

(Major) Line 279: The sentence should describe the gained loop interactions more explicitly: which regions do they connect? What are the characteristics of the connected regions? In addition, the end of the sentence stating that Mesp1 is upregulated « SINCE the new loops bring distal enhancers to its proximity » requires functional validation, typically by targeted deletion of the putative enhancer regions.

We have revised this paragraph as below providing more detail about the regions connected by the gained loops.

*"…In this example Mesp1 lost its loop interaction to a nearby FIN (**Fig. 5e**, blue arches) and upon CTCF depletion, gained promiscuous distal interactions to a large region that is ~200kb in size (**Fig. 5e**, red arches)..."* (line 375-377)

We also rephrase the text as below which describes the enhancer interactions without causation inference.

*"…Again, using 4C-seq we confirmed the gain of interactions to the large distal region containing several enhancer clusters (**Fig. 5e**, highlighted in green), and confirmed the upregulation of Mesp1 with RT-qPCR (**Fig. 5g**)…"* (line 379-382)

Line 281: The sentence should be toned down to « strongly suggest » (that FINs execute their insulating functions in cis) or similar, without further evidence with allele-specific CTCF perturbation and allele-specific gene expression measurements.

We agree "in cis" is not well supported and have revised this sentence as below:

"*…Taking together, these experiments verified the insulating functions of FINs identified from CTCF-degron Hi-C data…*" (line 381-382)

DISCUSSION

Line 293: Mentioning that « most TAD boundaries do not gain cross-boundary loops upon CTCF depletion » to contrast them with FINs is highly confusing, because neither do most FINs gain loops in the same condition. Instead, new loops may be gained between other regions that were previously blocked by FINs. This point needs to be clarified to avoid any confusion, especially between FINs and the gained putative E-P interactions.

We apologize for the confusion and thank the reviewer for pointing this out. To make it clear, we have rephrased in the revision:

"…Most TADs disappear upon CTCF depletion, and we typically do not observe gain of new loops across TAD boundaries. In contrast, most of the FINs are anchors of CTCF loops inside TADs in control cells, and we call an CTCF loop anchor "FIN" only if its flanking sequences gain new loops…" (line 393-396)

(Major) Line 304: The sentence is not sufficiently supported by data, as explained in the comment on lines 172-175 above.

This sentence was:

"…meta-analysis with Rad21- and Wapl-degron Hi-C data shows that the newly gained E-P loops in CTCF-depleted cells are cohesin-dependent and governed by cohesin regulators…"

In response to the reviewer's comments above. In this revision we have rephrase this sentence to:

"…meta-analysis with Rad21- and Wapl-degron Hi-C data shows that the newly gained E-P loops in CTCF-depleted cells do not form in cohesin-depleted cells and are also likely to be governed by cohesin regulators…" (line 410-412)

Line 306: The sentence describes a very weak and unconvincing evidence for cohesin dependency, through localization of FINs almost essentially in compartment A.

We have rephrased this sentence.

From:
"…the fact that almost all FINs are in compartment A (euchromatin) is also consistent with high cohesin activity because cohesin and its loading factors are known to co-localize with promoters and enhancers…"

To:
"…the fact that almost all FINs are in compartment A (euchromatin) suggests their dependency on surrounding active chromatin. Notably, promoters and enhancers may play key roles in loop initiation and stalling and are known to co-localize with cohesin and its loading factors…" (line 405-408)

(Major) Without functional validation, E-P loops in this study should be considered as putative, as the enhancers' activity and capacity to regulate the considered genes were not formally assessed.

We thank the reviewer for the comments.

Our validation experiments focus on disrupting insulators. **Figure 5** validated two FINs in which genes are upregulated when CTCF are displaced. The new **Fig. 4k-l** validated one more FIN at the downregulated Pcdh-gamma locus with CRISPR deletion assay. In all experiments we observed gain/loss of putative EP interactions with consistent transcription changes.

We agree with the reviewer that EP relationship remains putative without formally assessing enhancer functions with techniques such as dCas9-KRAB-based epigenome editing. We have adjusted the language throughout the manuscript using "putative enhancers" or "H3K27ac peaks" when mentioning candidate enhancer regions marked by H3K27ac.

METHODS

(Major) Line 639: What is meant by « For TADs and their boundaries, we directly use the TADs in mESCs defined by Dixon et al. »? Was the procedure re-used, or the same actual TAD and boundary calls were used? In the latter case, the use of TAD and boundary calls from a different study, Hi-C protocol and mESC line (J1 mouse ES cells in Dixon et al.) could readily explain the poor overlap between FINs and TAD boundaries. To ensure comparability, TADs/boundaries and FINs should be called from the exact same datasets.

Our previous submission used the same actual TAD calls by Dixon *et al.* from J1 mESCs.

In the revision, we now report TAD boundaries directly called from the control cell data in the CTCF-degron datasets. The new results are shown in **Fig. 3f-i** and **Supp. Fig. 11**. The methods have been updated as well.

Reviewer #2 (Remarks to the Author):

Map Functional Insulators with Cross-Platform Hi-C Meta-Analysis

General Thoughts:

Xu et al. characterize functional insulating regions of the genome (termed FINs) that contribute to gene regulation. They begin by analyzing multiple Hi-C and Micro-C datasets involving CTCF, Rad21, or Wapl depletions (Figure 1), using HiCorr and DeepLoop to characterize loop dynamics and identify gained loops upon CTCF depletion. They extend this analysis to other depletion datasets and compare loop dynamics across CTCF, Wapl, and Rad21 perturbations (Figure 2).

FINs are defined insulating regions where de-novo loops form between CREs upon CTCF depletion and are categorized into three types based on CTCF presence (Figure 3). They further link these regions to expression changes and describe new loop dynamics at FIN-containing regions (Figure 4). Finally, they use a dCas9 system to functionally validate selected FINs by targeting CTCF sites and observing transcriptional changes at loci such as Cdiptos and Mesp1 (Figure 5).

This study addresses an important question: why perturbations to chromatin conformation have profound effects in some genomic contexts but not others. While the authors attempt to define FINs in mESCs, the work would benefit from a deeper investigation into what makes an insulating region functional. Additionally, the figures often feel incomplete—more extensive display of the Hi-C/Micro-C matrices would add clarity to the results. Although the authors leverage multiple dataset, the cross-study comparisons are at times underutilized. Overall this work would benefit by further analysis into the FINs they define.

We thank the reviewer for recognizing our work's significance and the careful review of our work. We also appreciate the suggestions and below we have carefully answered the reviewer's comments and added new experiments and analyses into the revised manuscript.

Major comments:

Figure 1: The signal for Rhodes et al. seems very sparse. It would be helpful to show how these regions appear across other datasets, not just in CTCF depletion. Additionally, the general comparisons between datasets are limited to coverage metrics and a few loci. Performing PCA or a similar analysis on the different Hi-C/Micro-C experiments would better contextualize discrepancies between results and substantiate claims about differential depletion across studies.

Fig.1d already included the heatmaps of one CTCF-degron, one Rad21-degron, and one Wapl-degron datasets at *Notch1* locus. Our old **Supp. Fig. 1** (now **Supp. Fig. 4**) also showed two other CTCF degron datasets. We have now added new **Supp. Fig. 4g-h** which are heatmaps from the other two Rad21-degron and the other two Wapl-degron studies.

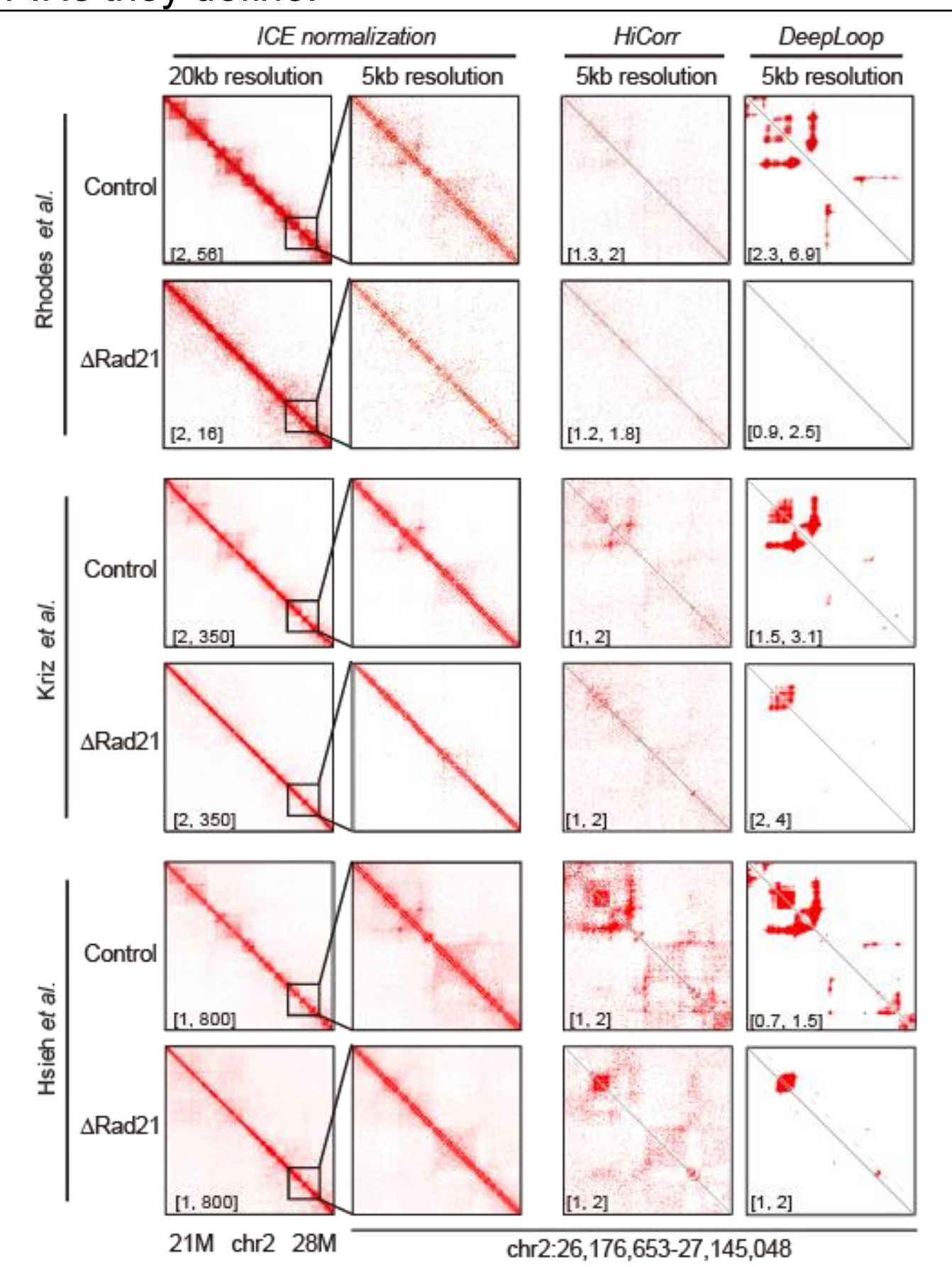

Reviewer Fig. 2. Contact heatmaps of the Rad21- Hi-C data at Notch1 locus generated from ICE, HiCorr and DeepLoop.

Reviewer Fig. 2 (panels from **Fig.1d** and **Supp. Fig. 4g**) on the right shows the Rad21-degron heatmaps at Notch1 locus. From this figure we can see that although

the signal for Rhodes *et al*. is indeed sparse, *DeepLoop* can robustly enhance the loop signal to the level comparable to Kriz and Hsieh (see the heatmaps of control cells in **Reviewer Fig. 2**).

In fact, because the control cells from all datasets are equivalent to wild type mESCs, their *DeepLoop* heatmaps are very similar. To quantify the consistency between orthogonal Hi-C experiments, we added a massive new **Supp. Fig. 2** in which we show more heatmaps and used scatter plots to compare the intensity of pixels (each pixel represents a pair of 5kb bins) between independent contact heatmaps. We also added a new **Fig.1b** (shown on the right) summarizing the overall correlation between control cells from different studies. Although conventional ICE-based pipelines show poor pixel-by-pixel correlation with R-square values often close to zero, HiCorr and DeepLoop clearly improved the consistency between orthogonal Hi-C/micro-C experiments with much better cross-platform R-square values (**Fig. 1b**).

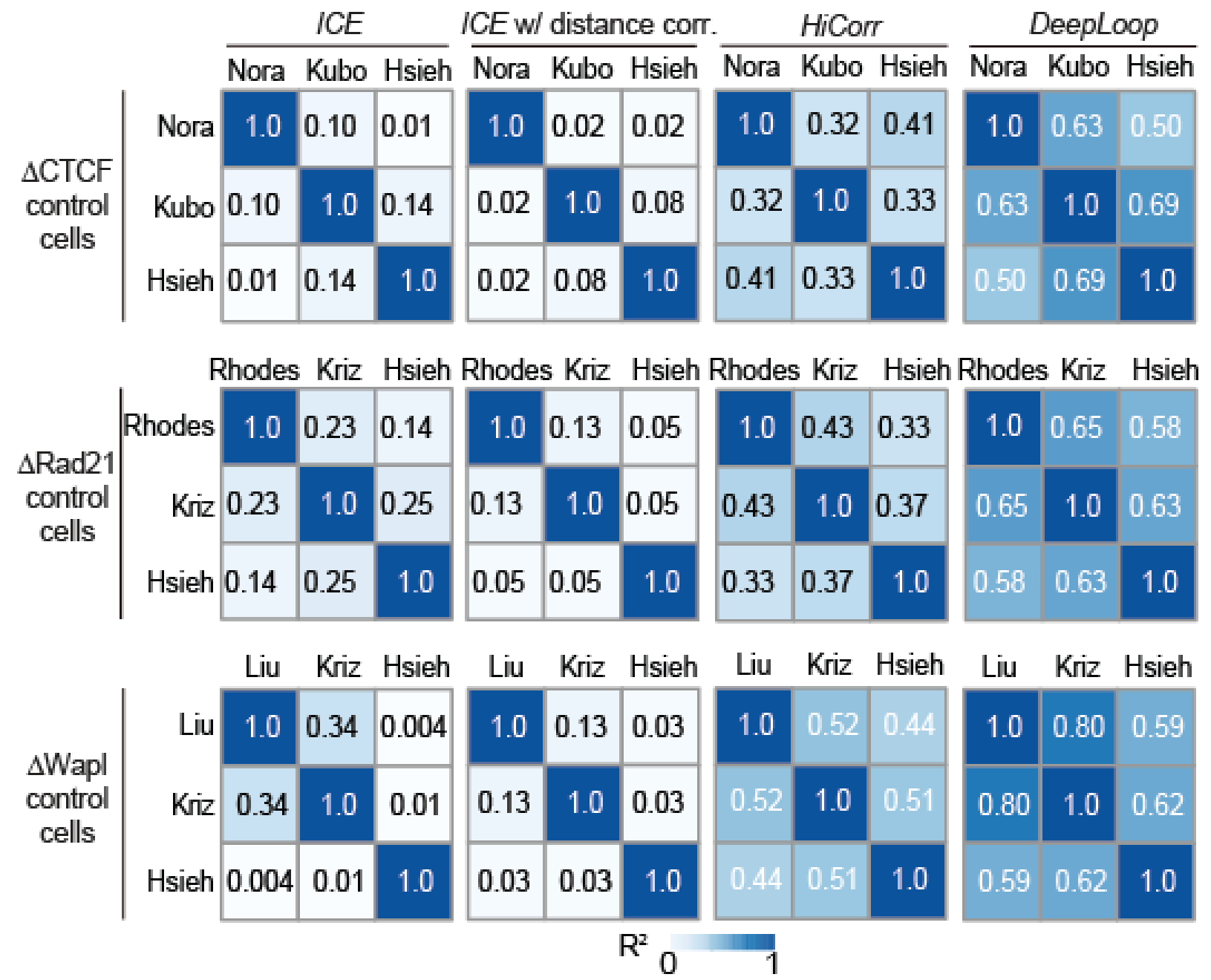

Fig. 1b. R-square matrices comparing Hi-C heatmaps in control mESC cells from different datasets when analyzed by different pipelines.

As the reviewer suggested, we also added a new **Supp. Fig. 3** below showing the cross-platform consistency when applying different pipelines to analyze <u>dynamic loops</u>. In this analysis we first generate "differential heatmaps" at 5kb resolution by subtracting each pair of contact heatmaps (depletion - control), then compute the correlations between the "differential heatmaps" from all nine pairs of degron experiments. Only *HiCorr* and *DeepLoop* pipelines can cluster CTCF- and Rad21-degron datasets together, and Wapl-degron datasets are separated (**Supp. Fig. 3**). This is consistent with the knowledge that depletion of either CTCF or Rad21, but not Wapl, causes massive loss of TADs and CTCF loops. Please note that we also included **Supp. Fig. 3a,d** into a new **Fig. 1c** in the main figure.

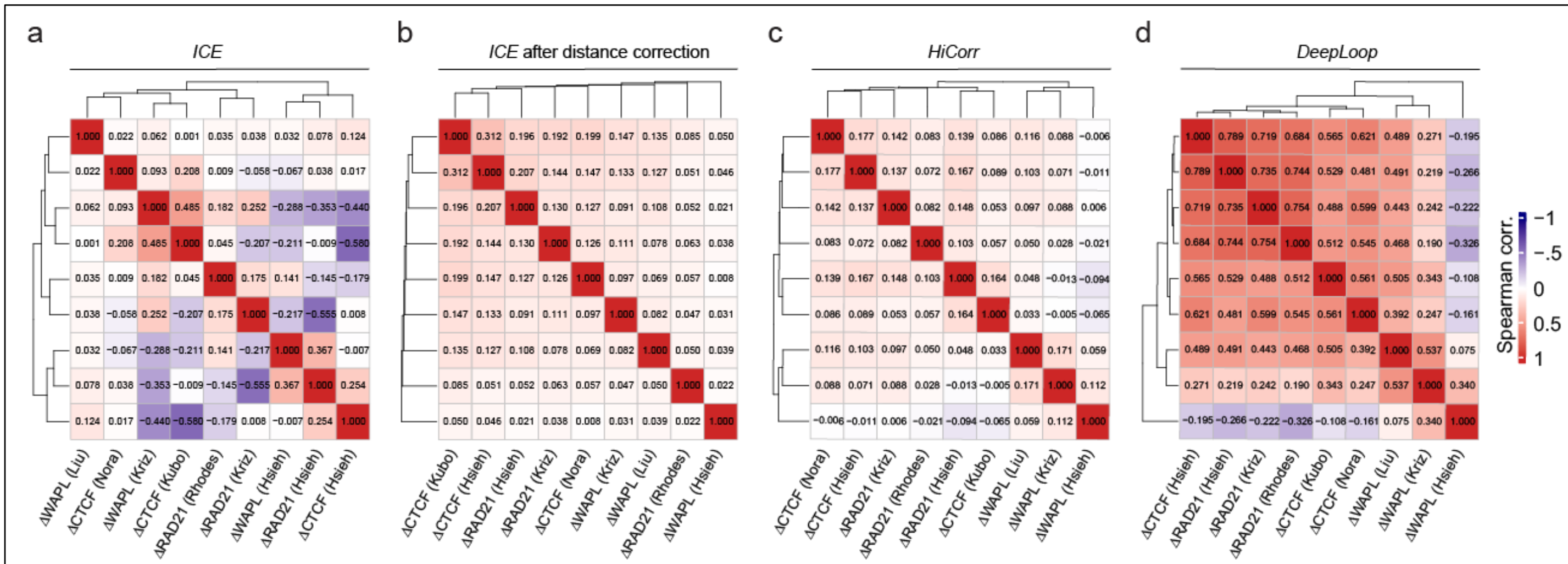

Supp. Fig. 3. Correlation matrices comparing the profiles of dynamic loops from all nine pairs of degron experiments.

It's a bit unclear what a pixel represents—I'm assuming one bin? What is the bin size, 5kb? These were then merged into regions down the line, but this is also not clearly explained as to how, or what exactly that means.

When talking about a loop pixel, we are referring to a pixel in the 5kb resolution contact heatmaps, which represents a pair of 5kb-bins.

We merge pixels to count genomic regions of interest. Specifically, we have ~5,000 “gained” loop pixels but many of these pixels occupy overlapping regions. We therefore merge all overlapping loops into one region and define them as “loop-gaining loci”. This step allows us to count the number of regions of interest in the genome. In our study, we have ~300 loop-gaining loci after merging ~5,000 recurrent “gained” loop pixels.

We have clarified the definition of loop pixels and described how to merge pixels in the revised main text and Methods.

I wonder if the longer gained loops being captured in Rhodes et al. (Supp Fig 3 – lines 150) are seemingly longer because the data is too scarce to capture short range loops that might need more coverage to be visible regardless of bias correction or DeepLoop.

In **Reviewer. Fig. 3** on the right, we compare the size of “lost”, “retained”, and “gained” loops from three Rad21-degron datasets (panels from **Supp. Fig. 7a,d,g**). We reported the “gained” loops from Rhodes *et al.* are longer, which is consistent with the conclusion by Rhodes *et al.* that cohesin removal enhances a series of long-range interactions between a subset of polycomb-occupied regions.

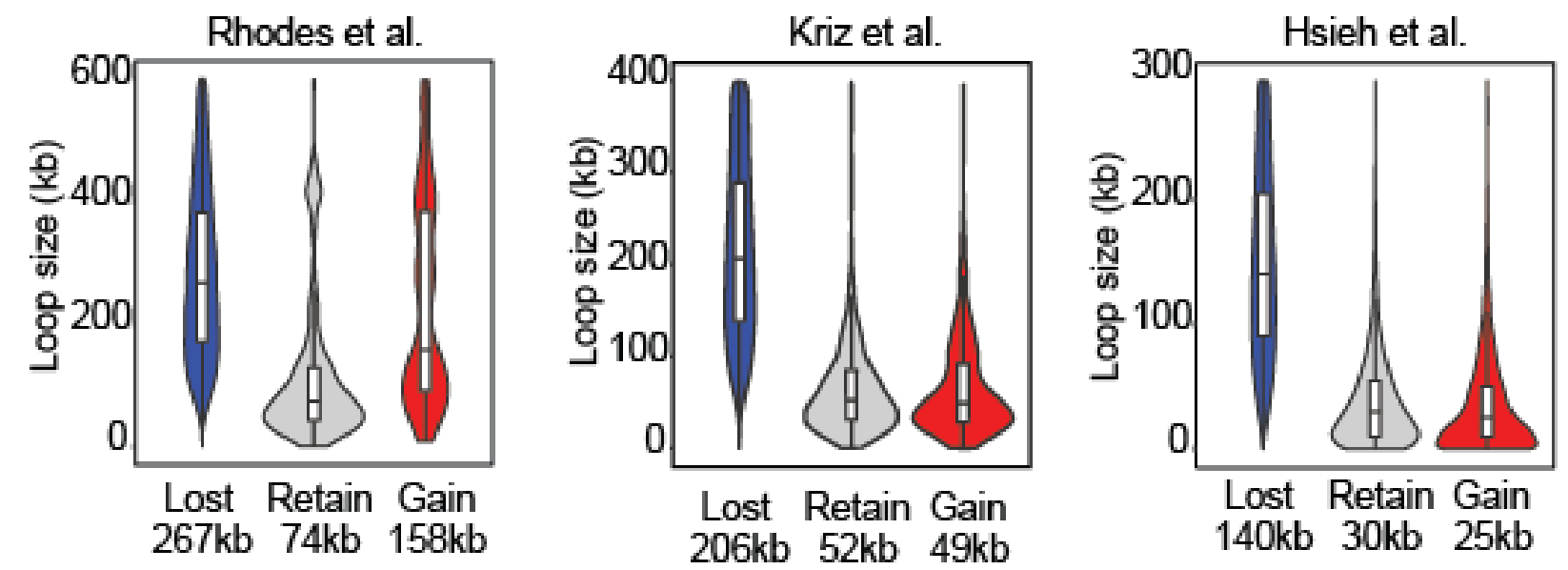

Reviewer Fig. 3. Loop size analysis in three Rad21-degron datasets.

Note that the “gained” loops in Rhodes *et al.* are longer than the “retained” loops in the same study. Data sparsity cannot explain this difference because the “retained” loops from Rhodes *et al.* are also called from low-depth Hi-C data (both control and ΔRad21 Hi-C data from Rhodes *et al.* are low-depth). In contrast, the “gained” loops in Kriz *et al.* and Hsieh *et al.* are smaller than “retained” loops from the same studies (**Reviewer. Fig. 3**).

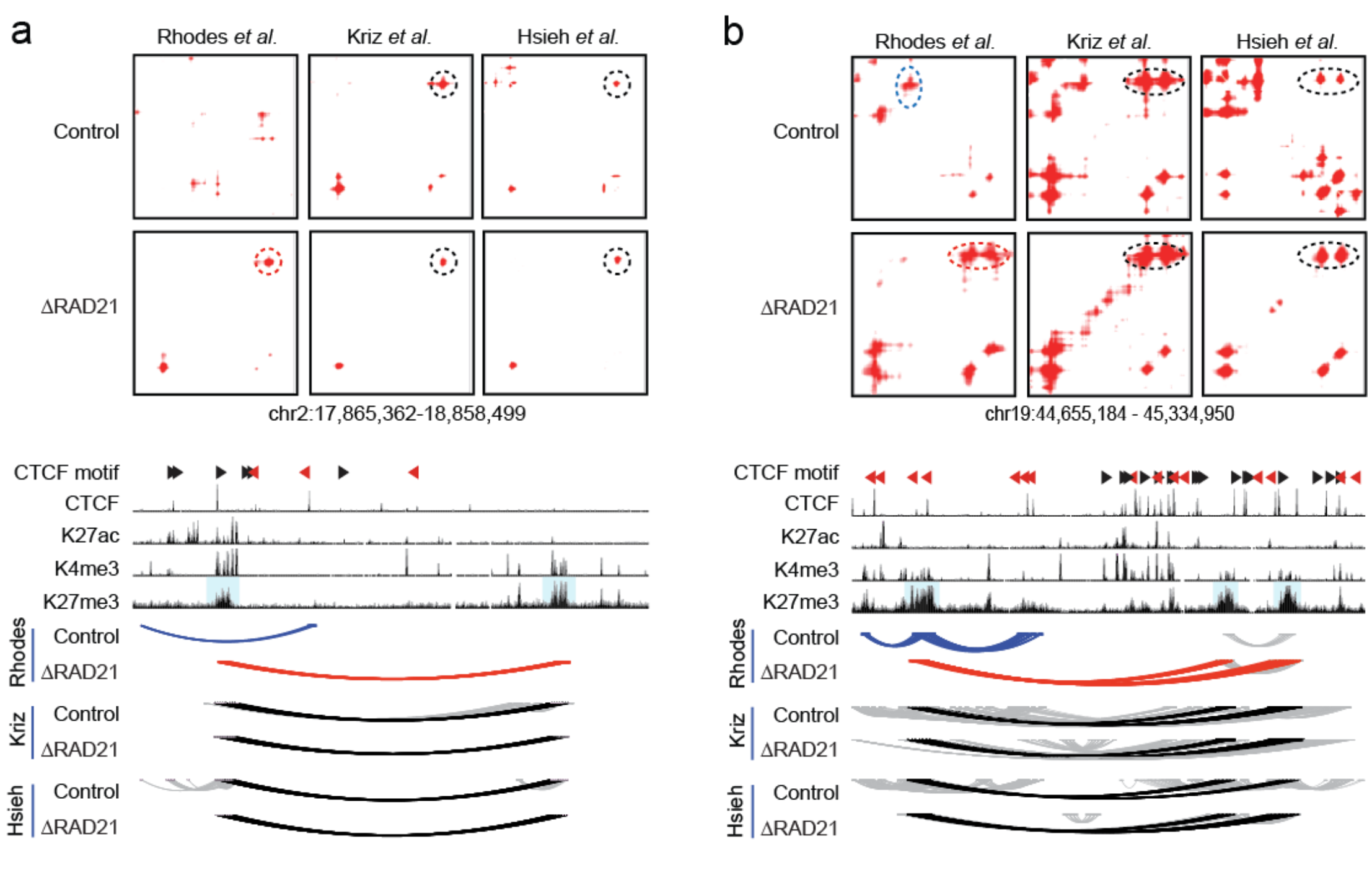

Supp. Fig. 8. “Gained” long-range H3K27me3 loops in Rad21-depleted cells from Rhodes et al. already exist before Rad21-depletion in two other studies.

We further examined the “gained” long H3K27me3 loops in Rad21-depleted cells from Rhodes *et al.* and found that in almost all cases, the “gained” long-range H3K27me3 loops are already present in the control cells from Kriz *et al*. and Hsieh *et al.* before Rad21-depletion (two examples are shown in a new **Supp. Fig. 8** above). Therefore, at least in the control mESCs from Kriz et al. and Hsieh et al., cohesin does not prevent the formation of these long-range H3K27me3 loops.

In lines 192-196, the term CTCF loop is brought up then switched to just insulation while this neither are really shown, more than just CTCF sites. It would be good to have an example region for each type of loop. TADs are also inconsistently discussed but not shown. Moreover, since TAD-calling can be noisy, more insulation score analysis at FIN vs non-FIN sites could strengthen the work.

We added a new **Supplementary Fig. 10** showing examples for each category of events discussed in **Fig. 3d**.

In this revision we also performed a much more comprehensive TAD level analysis shown in the new **Fig. 3f-i** and **Supp. Fig. 11.** We now call TAD boundaries in control cells using data from three CTCF-degron studies at 40kb resolution with two methods: (i) “Directionality index” (DI) -based method as described by Dixon et al.; (ii) “insulation score”-based method (Crane et al., PMID: 26030525) using *Cooltools*.

Indeed, as the reviewer commented, TAD boundaries calling can be quite variable depending on the dataset and algorithm. DI-method calls many more TAD boundaries than InsScore method. The number of TAD boundaries also tends to be higher when using more frequent Hi-C cutters (Micro-C > in situ Hi-C > Hi-C) (**Supp. Fig. 11a**). Unsurprisingly, the overlap between TAD boundaries and FINs increases if more TAD boundaries are called but regardless of the method of choice, it should be noted that TAD boundary always out-numbers FIN and only a fraction of FINs overlapped with TAD boundaries (**Supp. Fig. 11b**).

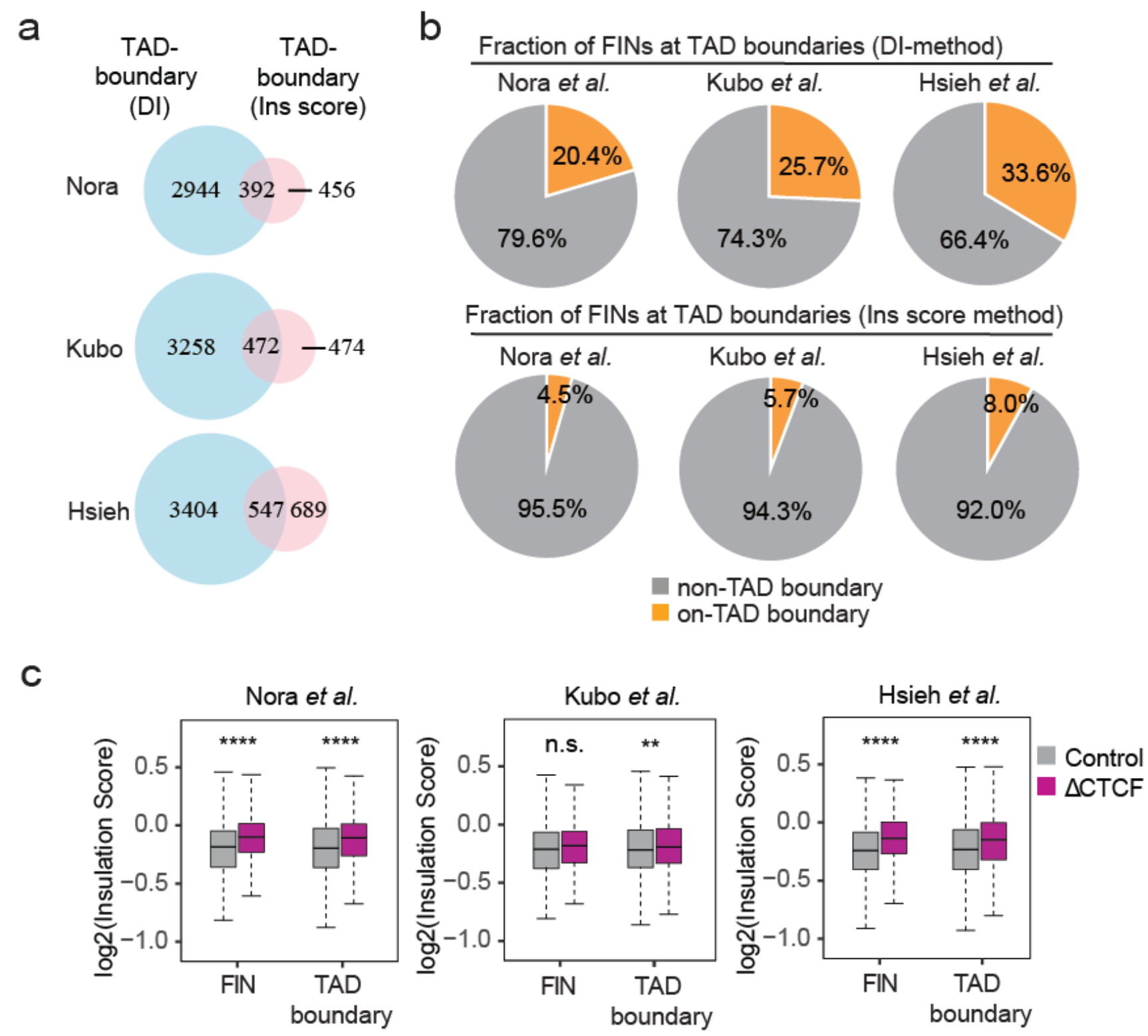

Supp. Fig. 11. (a) Compare the overlap between TAD boundaries called from DI and Insulation score. **(b)** Pie chart showing the percentage of FINs that overlap TAD boundaries called from different algorithms. **(c)** Compare insulation scores at FINs and TAD boundaries in different studies.

In this revision we also added a new **Fig.3f** with a FIN example (highlighted in pink) that is not at TAD boundary. Here the FIN is at the right anchor of a CTCF loop in control cells (blue arches). Upon CTCF depletion, the blue arches disappear and new loops (red arches) form connecting two clusters of

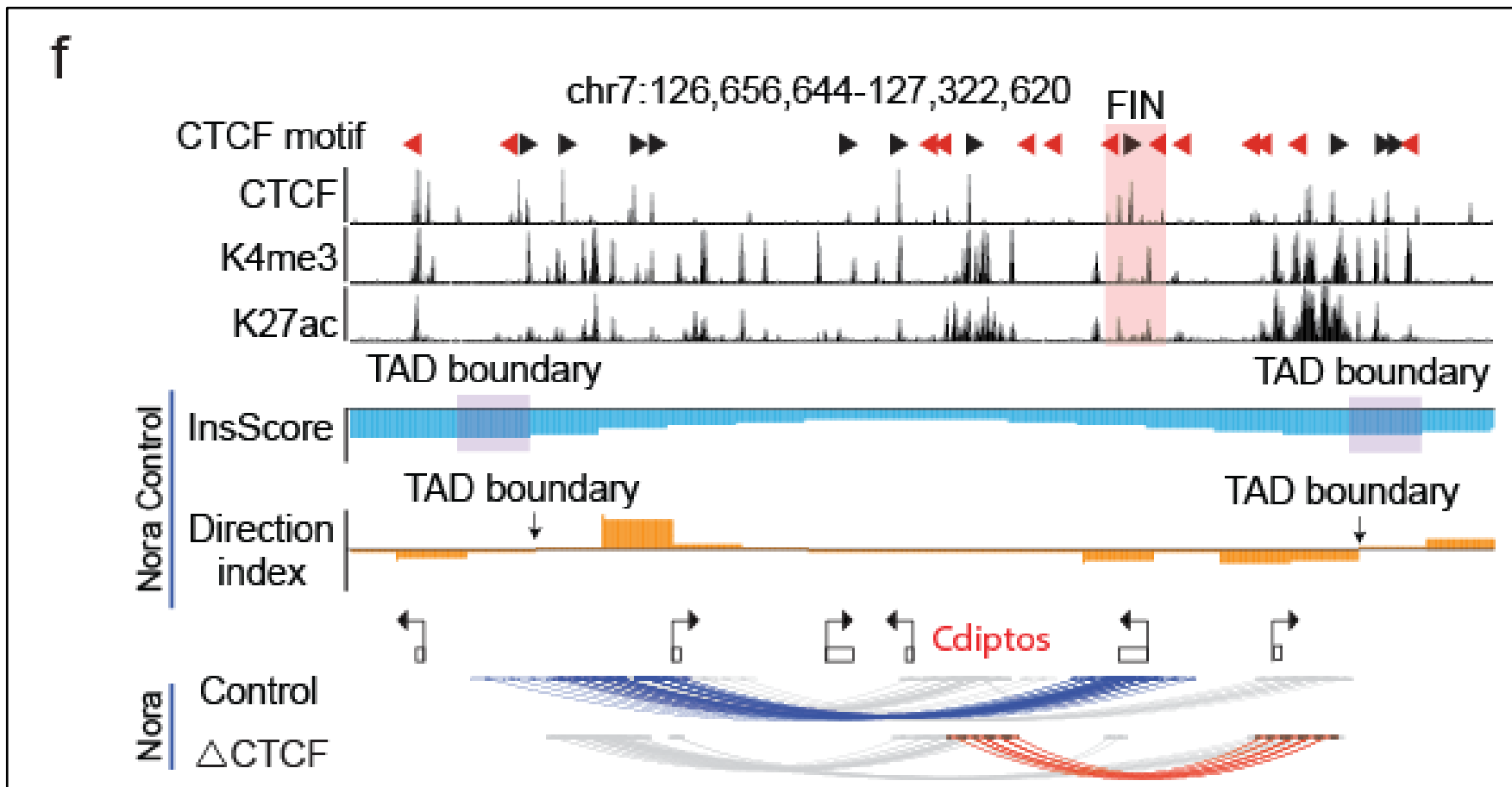

Fig. 3f. An example of non-TAD boundary FIN.

H3K27ac peaks flanking the FIN. In this figure we also included the tracks of “insulation score” and “directionality index”. Importantly, the two TAD boundaries in this example reach local minimum in the InsScore track but the FIN is not a local minimum (**Fig. 3f**). Similarly, using DI-based method, TAD boundaries are defined as locations where DI changes its sign, but in this example, the FIN is located inside a TAD and its DI does not change sign.

Change of InsScore also does not predict FINs. Although we observed an overall trend of increased InsScore at FINs upon CTCF depletion (except that the increase of “insulation score” in Kubo et al. is less significant) TAD boundaries also show the same trend and cannot be distinguished from FINs (**Supp. Fig. 11c**). Taking together, low-resolution TAD features lack the precision to reveal the dynamic changes of loop profiles as we used to define FINs.

This paper generally lacks in depth characterization of FIN’s. If there is ATAC-seq, for example, footprinting analysis would be interesting even in just wildtype mESC, to ask if there are any specific motifs or promoter types enriched at these regions which are being functionally insulated in mESCs? Moreover, Are these FINs dynamic across cell types, or stable but functionally relevant only in specific developmental contexts? Some of this is touched on in the discussion but could at least be looked at by analyzing a none-mESC Hi-C or Micro-C dataset.

We thank the reviewer for the great suggestions.

Following the reviewer’s suggestion, we added a new **Fig. 3l** on the right showing motifs that are enriched or depleted at loop-gaining ATAC-peaks in CTCF-depleted cells. Interestingly, the loop-gaining ATAC-peaks are enriched with many G-rich motifs but depleted with A/T-rich motifs (**Fig. 3l**). This result is reminiscent of previous reports that G-quadruplexes (G4) structure may regulate chromatin looping (Li et al., PMID:33199912; Wulfridge et al., PMID:37552993). Notably, many of the putative transcription factors binding to these G-rich motifs have been shown to regulate loop formation in various cell types, such as KLFs (Schoenfelder et al., PMID:20010836; Wei et al., PMID:23747203; Di Giammartino et al., PMID:31548608), SP1 (Bryan et al., PMID:40490791), MAZ and several zinc-finger proteins (Ortabozkoyun et al., PMID: 35145304, 39173638; Xiao et al., PMID: 33558242). Therefore, our results suggest that G-rich *cis*-regulatory elements are more likely to act as new loop anchors upon CTCF-depletion. We have added these discussions into the revised main text.

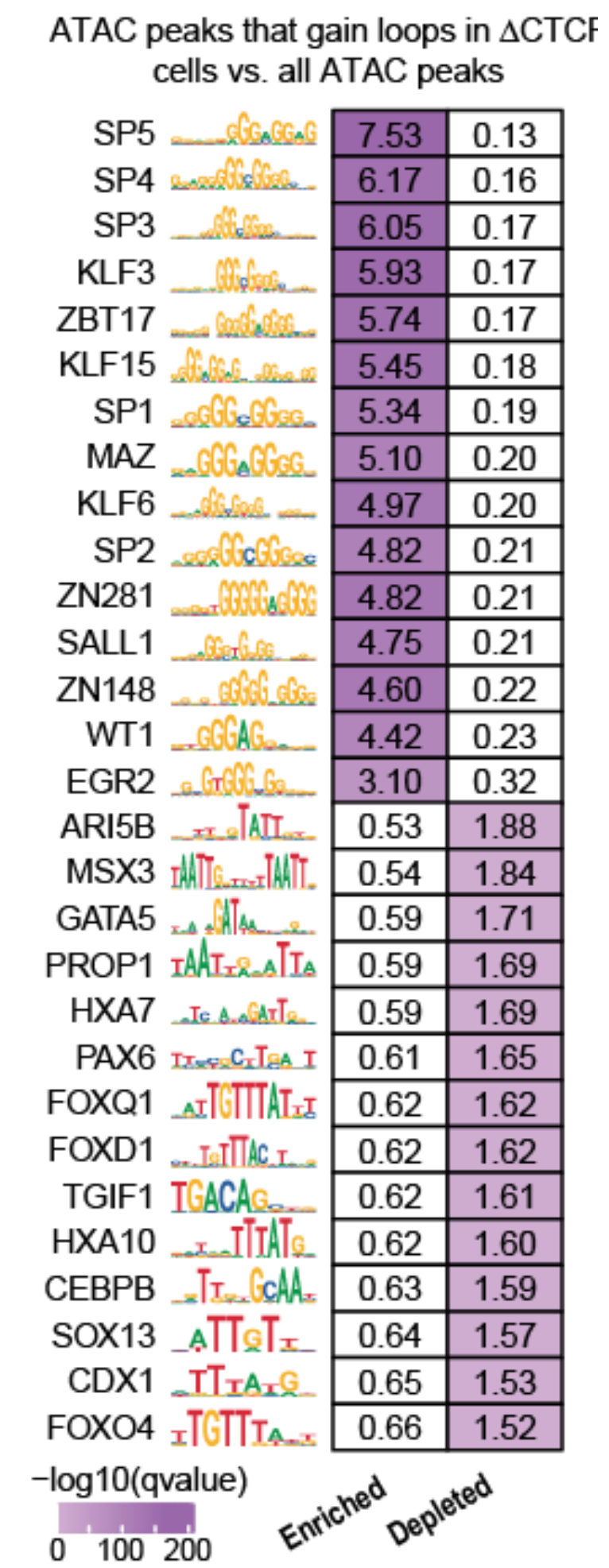

Fig. 3l. Motifs analysis at gained E-P loops in CTCF-depleted cells.

Regarding cell type specific FINs, we speculated in the discussion that FINs are likely to be cell type- or cell state-specific. However, mapping cell type-specific FINs requires CTCF-degron Hi-C data in a different mouse cell type, but to our knowledge, there’s no public CTCF-depletion Hi-C data in non-ES mouse cells yet. It will be certainly interesting to further explore the context specific roles of FINs in health and diseases in the future.

For Figure 3g: How does a FIN at a non-TAD boundary looks like, is it just because a TAD-call is not there or is it because this region shows no insulation (which is then a bit perplexing). While the schematics are helpful, real data should be shown. I would think that would be much more important for a main figure as a sanity check to the classifications than panel d for example.

We have already discussed on the last page of this document that we included a new **Fig. 3f** showing a non-boundary FIN example. This FIN is near the *Cdiptos* locus which is also shown in **Fig. 4g** and used for validation in **Fig. 5b-d**. **Fig. 3f** has included tracks of “insulation score” or “directionality index” with which we show that the FIN cannot be called by these measurements. Regardless of using “insulation score” or

“directionality index” based methods, TAD boundaries outnumber FINs, and only a minority of FINs overlapped with TAD boundaries. We note that FINs are defined based on dynamic change of high-resolution loop profiles and cannot be simply represented as TADs or TAD boundaries because after all, TADs or TAD boundaries are low-resolution 3D genome features.

Many of the figures need much better background and Supplemental to back up the claims being made. Few examples:

1. A bit more about the enhance models, since these were specific to each experiment.

We have added more descriptions about how we choose LoopEnhance models for different Hi-C datasets in Method section.

2. Classification of chromatin loops based on the epigenetic data, it would be helpful to see more examples of loop pixels within each group, and to see what “other” is, is it noisy regions of the genome for example or just regions with no CHIP coverage?

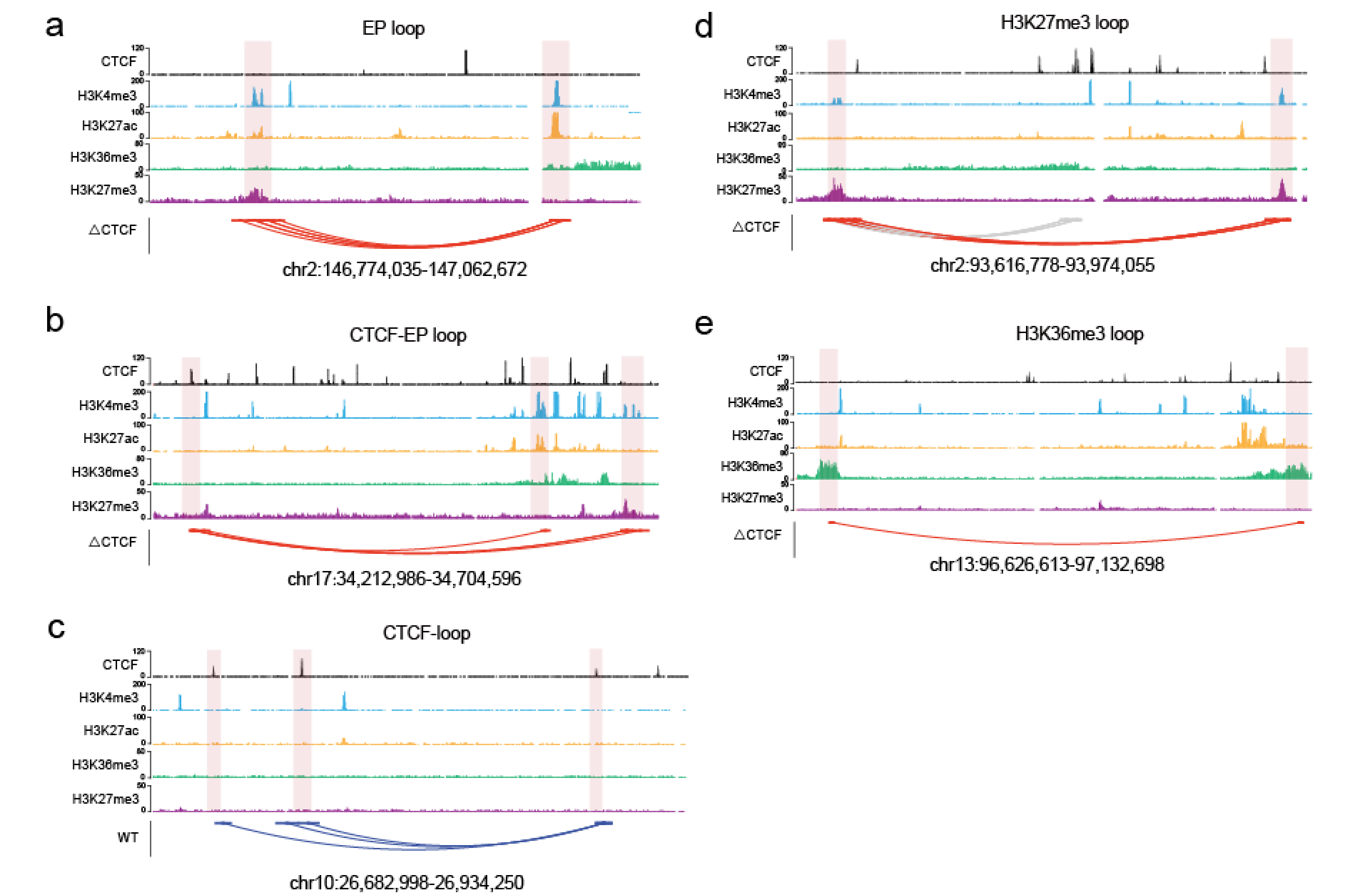

Supp. Fig. 6. Examples of chromatin loop classification.

We have included a new **Supplement Fig. 6** showing different types of loops. “Other” loops are pixels that neither end has ChIP-seq signal. We found many “other” pixels are “bystanders” that are next to a strong loop pixel (*e.g.*, a CTCF loop) but the two anchors do not have any ChIP-seq peaks.

3. A Supplement for Figure 4 could have easily incorporated the Hi-C maps between controls and degradation experiments without DeepLoop models, are they still visible?

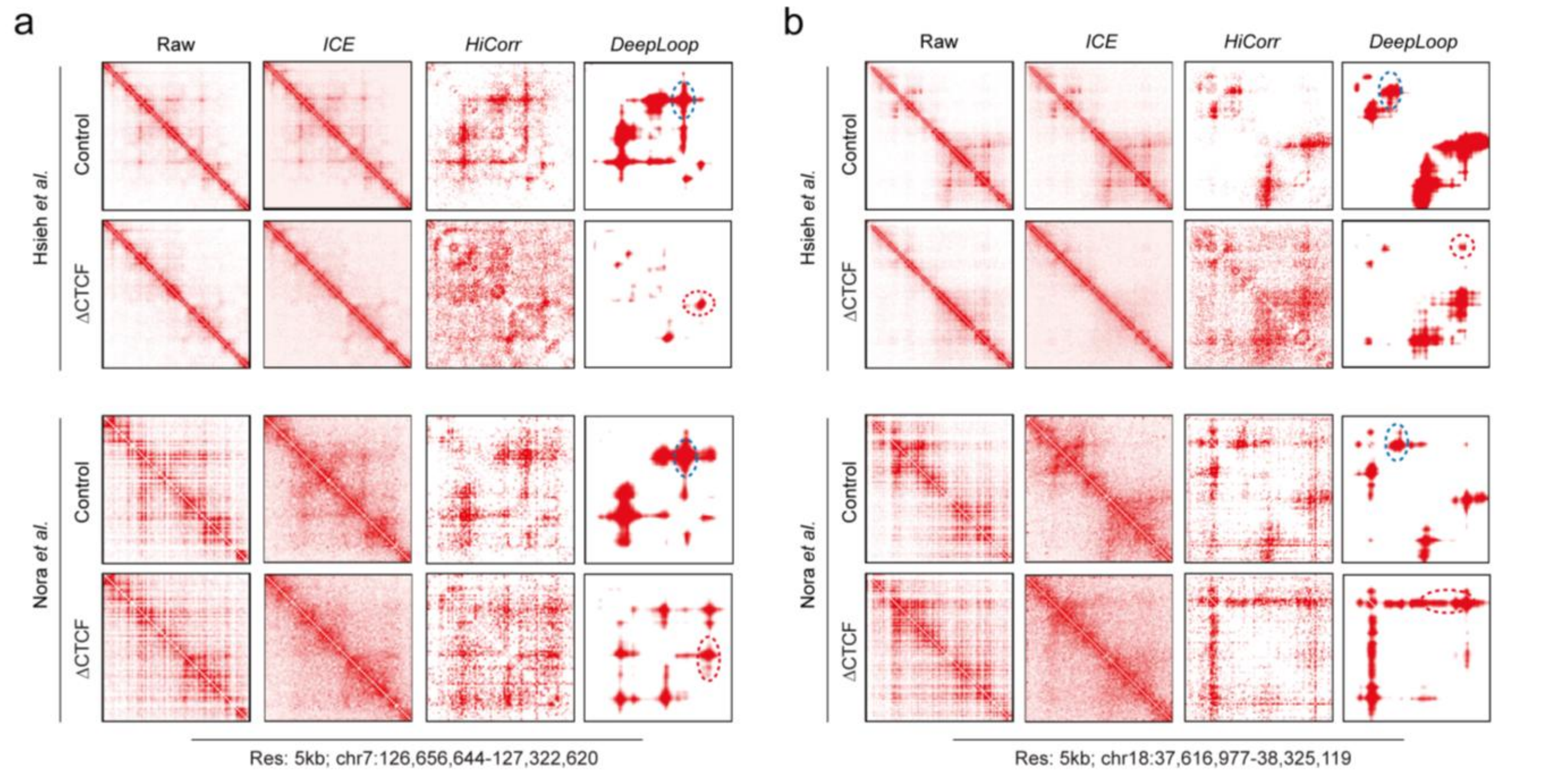

Supp. Fig. 12. Example contact heatmaps at putative insulator target genes Cdiptos and Pcdh-γ.

As suggested, we added a new **Supp. Fig. 12** showing Raw, ICE, *HiCorr*, and *DeepLoop* heatmaps for examples in **Fig. 4**. In all these examples, loop profiles are much cleaner in *DeepLoop*.

4. How was the RNA-seq normalized and how does that affect DEGs. Are ERCC spike-ins better than using empirical genes in RUVseq for example?

We performed differential expression analysis using *DESeq2*, which applies a robust normalization method based on scaling factors estimated from the median ratio of gene counts across samples. This approach assumes that most genes are not differentially expressed and is one of the most widely used methods in bulk RNA-seq studies.

RNA-seq data from Nora *et al.* and Kubo *et al.* do not have ERCC spike-ins. In **Reviewer Fig. 4**, we compared differentially expressed genes (DEGs) called with *DESeq2* and *RUVseq* packages**.** Applying RUVseq without ERCC spike-ins is tricky because we need to empirically pick some genes as negative controls. Here we used four housekeeping genes (*Actb*, *Gapdh*, *Pgk1*, *Atp5f1*) to estimate unwanted

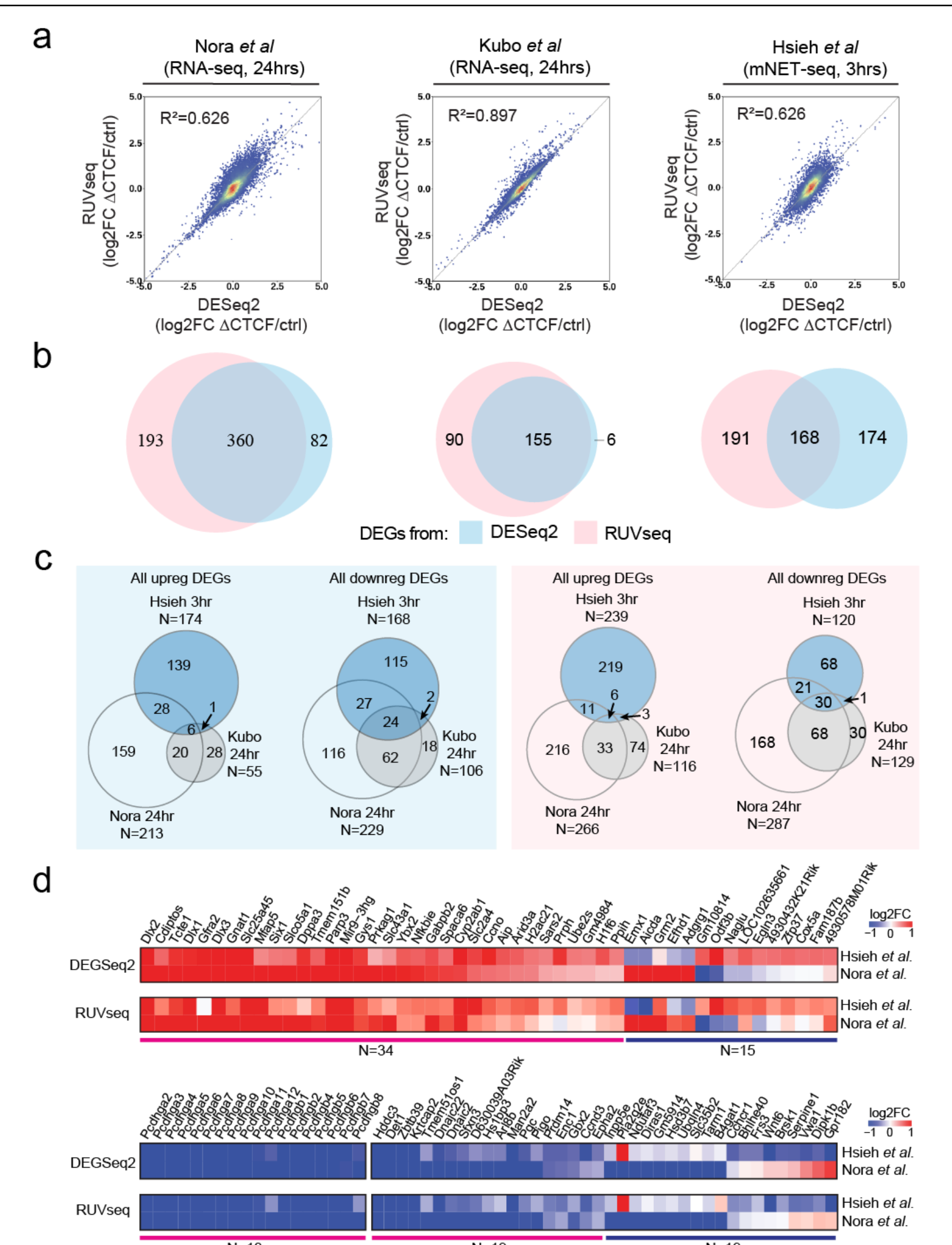

Reviewer Fig. 4. Compare DESeq2 and RUVseq in the differentially expressed gene analysis.

variations for RUVseq and compare the results to those from DESeq2.

We compared the fold-change values (ΔCTCF/control) of all genes from DESeq2 and RUVseq for all studies and the fold-change values are highly consistent with each other (**Reviewer Fig. 4a**). DEGs called from the two pipelines overlapped well. The overlap of Hsieh et al. data is lower because for NET-seq data we use looser cutoff for fold changes (we used 2-fold and $p<0.05$ for RNA-seq and 1.5-fold and $p<0.05$ for NET-seq to call DEGs) (**Reviewer Fig. 4b**). Regardless of DEseq2 or RUVseq, the reproducibility between the three independent studies is limited (**Reviewer Fig. 4c**). Finally, the putative FIN target genes (which are DEGs at "gained" loop anchors upon CTCF depletion) show consistent transcription changes regardless of using DESeq2 or RUVseq (**Reviewer Fig. 4d**). We therefore believe that using different RNA-seq analysis pipelines do not affect our conclusions.

Figure 4f isn't clear. Also, switching between Nora/Kudo and Hsieh datasets across subpanels (f–j) is confusing.

As we showed in **Fig. 4e**, the reproducibility of DEGs is limited between independent CTCF-degron studies due to experimental variations and indirect effects caused following CTCF-depletion. Our assumption is that experimental variations and indirect effects are less likely to be reproducible.

We hypothesize that DEGs near FINs are more likely to be the direct targets of CTCF-mediated enhancer-promoter insulation. If this hypothesis is true, DEGs near FINs should be more reproducible between independent CTCF-degron studies. Indeed, the upregulated DEGs near FINs (i.e., at "gained" loop anchors upon CTCF depletion) are more reproducible, but downregulated DEGs near FINs are less reproducible than controls (**Fig. 4f**, red bars). We have revised the main text with this explanation in line 326-332.

We also revise the text further explaining why we focus on the comparison of RNA-seq data from Nora (RNA-seq, 24hr) and Hsieh (NET-seq, 3hr) as following:

"…Both Nora et al. and Kubo et al. performed RNA-seq and the earliest timepoint was 24hr CTCF-depletion, which may still include late-response DEGs caused by indirect mechanisms (e.g., loss of pluripotency). Among the two RNA-seq studies, Kubo et al. detected fewer DEGs than Nora et al., but 70% of Kubo et al.'s DEGs are also observed in Nora et al. (47% of the upregulated DEGs and 81% of the downregulated DEGs, Fig. 4e). This observation is consistent with our discussion above that CTCF depletion in Kubo et al. was less complete. Hsieh et al. performed both NET-seq and RNA-seq after only 3hr CTCF-depletion, which should include fewer late-response genes, but the short treatment time may also reduce the sensitivity of DEG detection. We choose to use NET-seq data because the Hsieh et al. reported that NET-seq has higher sensitivity in identifying DEGs as it maps newly synthesized RNA, while RNA-seq measures the amount of steady state RNA in the cells…" (line 313-324)

The functional validation experiments are nice. However, matrix views of the targeted regions should be shown in addition to the *DeepLoop* model outputs. I think it would be also interesting to do this on a down regulated gene.

As the reviewer requested, we have added raw, ICE, HiCorr, and DeepLoop heatmaps for these two examples into **Fig. 5b,e**.

We also performed a new validation experiment showing that deleting a putative insulator indeed leads to down-regulation of γa and γb genes. The results are now included in the new **Fig. 4k-l** shown below.

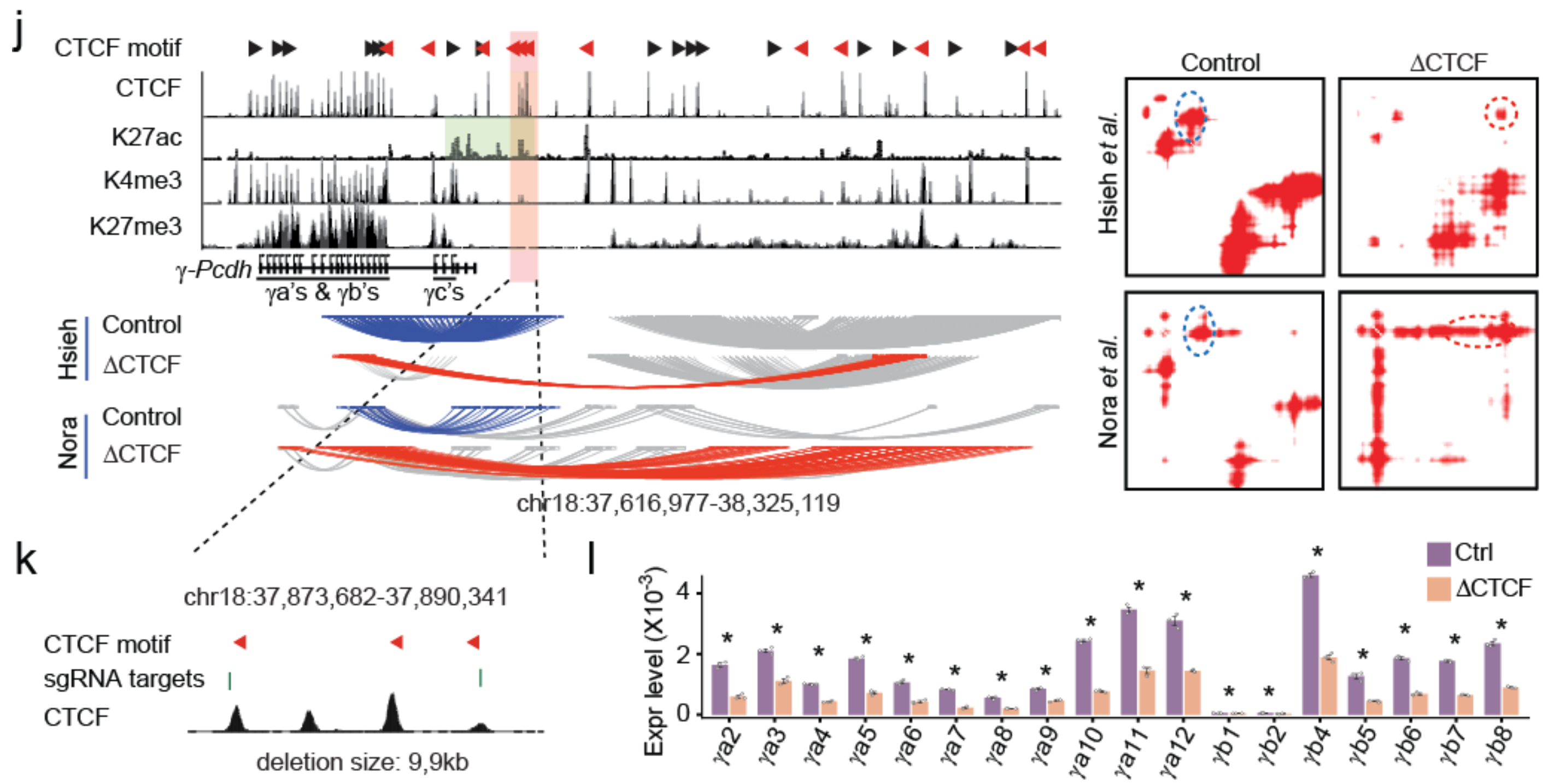

Fig. 4j,k,l. **(j)** Left: browser tracks near *Pcdh-γ* locus; right: Hi-C heatmaps from two CTCF-degron studies. **(k)** Design of sgRNAs for insulator deletion. (**l**) RT-qPCR shows the down-regulation of *Pcdh-γ* genes in mESCs after insulator deletion.

Minor comments:
The title needs improvement.

We have changed the title to:
"Cross-platform Hi-C meta-analysis identifies functional insulators that actively block enhancer-promoter interactions."

Supplemental Figures 3 and 4 should be made into a single supplementary figure.

We have merged these two supplementary figures into a new **Supplementary Fig. 7** as the reviewer suggested.

Line 180 ("more analysis below") can be deleted.

This sentence has been deleted.

Figure 4c could be moved to the supplemental section; the plot is confusing and adds limited value.

We have revised **Fig. 4c** to include only one pie chart showing that 16.8% of enhancers blocked by FIN are super enhancers.

For Figure 4e, adding NET-seq data next to Hsieh's data and RNA-seq results for other experiments would make comparisons easier.

We have revised **Fig. 4e** as advised with labeling of "NET-seq" for Hsieh's transcriptome data and "RNA-seq" for Nora's and Kubo's transcriptome data.

In Line 232, it's overstated that "most" DEGs overlap between Kudo et al. and Nora et al.—especially considering upregulated genes.

We have revised the sentence to make it clear that 47% of the upregulated DEGs and 81% of the downregulated DEGs from Kubo's data are also among Nora's DEGs (line 317-318).

Reviewer #1 (Remarks to the Author)

All my previous points have been satisfactorily addressed, and I recommend the manuscript for publication.

Reviewer #2 (Remarks to the Author):

Cross-platform Hi-C meta-analysis identifies functional insulators that actively block enhancer-promoter interactions Cui, Xu, Lang et al.

General Thoughts:

The authors have added substantial new analysis and some experiments to the work. They have also improved the overall discussion of their results, which initially contained too much over-interpretation with limited support, as also noted by Reviewer #1. While the manuscript has improved, figure presentation for easier interpretability can still be improved.

We sincerely thank the reviewer for the time and constructive feedback. We are pleased to see that the additional experiments, expanded analyses, and the refined discussion have addressed the previous concerns regarding interpretation. Following the reviewer's latest suggestions, we have further revised the manuscript and figure presentation to improve interpretability.

Minor comments:
1. Sentence 44-46:
"TADs disappear upon loss of NIPBL, while loss of WAPL causes bigger loops because cohesin can pass through CTCF blocks due to prolonged loop extrusion" could be rewritten to be a bit more readable.

We thank the reviewer for the suggestion. We have revised the text to improve flow and readability as follows (Line 46-49):

"…While deleting MAU2 or NIPBL results in the disappearance of TADs and a massive reduction of chromatin loops, the loss of WAPL produces the opposite effect, generating larger loops by enabling cohesin to bypass CTCF barriers through prolonged loop extrusion…"

2. Section 116-121
Current text:
"Only the matrices from HiCorr and DeepLoop clustered CTCF- and Rad21-degron datasets together and Wapl-degron datasets are separated out (Fig. 1c, Supplementary Fig. 3). This is consistent with the knowledge that depletion of CTCF or Rad21, but not Wapl, causes massive loss of TADs and CTCF loops. Taken together, these results support the robustness of DeepLoop in cross-platform analysis regardless of read depth."
I think it would be worth noting that both HiCorr and DeepLoop signals appear to saturate toward looping (as one would expect of DeepLoop for example). In contrast, ICE/ICE+distance correction may retain additional or broader structural features, which could explain the lack of clustering/correlation. Clarifying this would strengthen the interpretation.

We agree that the divergent performance of these methods partly reflects their different priorities: while ICE-based normalization preserves more architectural features, the HiCorr/DeepLoop pipeline is specifically optimized to prioritize discrete loop signals. We have added the following clarification to the manuscript (Line 131-139):

"…We attribute this improved robustness to the fact that the HiCorr/DeepLoop pipeline prioritizes loop signals through rigorous bias correction and normalization. This process effectively eliminates signals arising from both platform-specific technical biases (e.g., restriction site density and distribution) and non-loop chromatin interactions, including non-specific local chromatin compaction and other broader structural features. While the non-loop architectural signals retained by ICE-based normalization may carry biological meaning, they are

often highly sensitive to protocol variations in kilobase-resolution analyses. At this scale, such features can act as confounding noise, compromising the consistent and accurate quantification of loop strength across disparate datasets…"

3. Figure interpretability:
I think a simple heatmap (as in deeptools) of different tracks (H3K27ac, H3K27me3, RNA-seq, etc.) at loop anchors of FINs (perhaps even separated by class type) could be useful to visually summarize the features associated with dynamic loops.
I appreciate the new analysis in Supplemental Figure 1. I do think a simple heatmap showing insulation at gained, lost, and maintained anchors would be interesting. This could help clarify whether these are regions that are already prepped but blocked, or if it's a mix of multiple dynamics including de novo gain of regulation. I would suggest using anchors rather than merged regions, as otherwise there may be too few regions and mixed sizes - but not sure.

We thank the reviewer for this excellent suggestion. We have now added a new panel to **Figure 2b** (shown as **Reviewer Fig. 1** on the right) that displays the ChIP-seq signals for various histone marks centered on the anchors of "lost," "retained," and "gained" loops. Following the reviewer's recommendation, we performed this analysis using discrete loop anchors rather than merged regions to maintain spatial precision. We found that RNA-seq data was unfortunately too sparse and too diverse to provide a clear visual summary in this format.

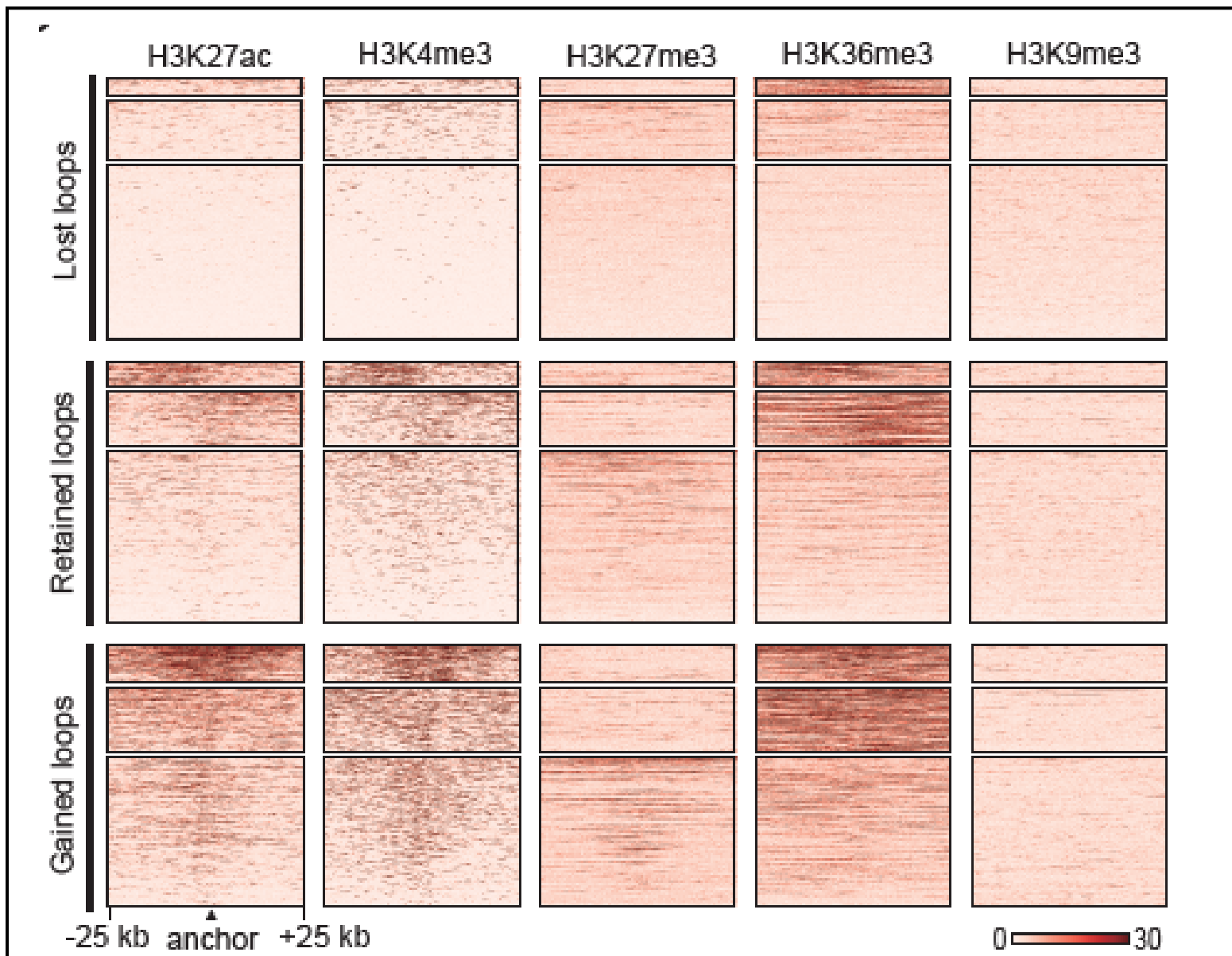

Reviewer Fig. 1. Heatmaps of ChIP-seq signal for histone marks at the anchors of lost, retained, and gained loop pixels in CTCF-depleted cells. The centers of loop anchors are aligned and the heatmaps plotted the regions ± 25kb of the anchor centers. Adopted from **Fig. 2b** in our manuscript.

As shown in the new Figure 2b, the anchors of "retained" and "gained" loops exhibit significantly higher enrichment of H3K27ac, H3K4me3, H3K27me3, and H3K36me3 compared to "lost" loop anchors. This enrichment indicates that, in the absence of CTCF, chromatin loops preferentially anchor at these epigenetically marked or "prepped" regions. This result is highly consistent with our loop classification in **Figure 2c**, which highlights a higher proportion of enhancer-associated loops within the retained and gained populations.

Regarding the question of "prepped but blocked" versus *de novo* regulation, the presence of these marks in the baseline (wild-type) state suggests that these regions are functionally "prepped" for interaction prior to CTCF loss. While we cannot directly measure histone mark changes following acute depletion (as these data were not generated in the original studies), the rapid timescale of CTCF degradation suggests that the observed loop rewiring primarily utilizes this pre-existing epigenetic landscape rather than relying on *de novo* chromatin remodeling.

4. Figure Organization:
Figure 1: I would move Nora et al. to the bottom to be closer to the tracks in panel e.

We thank the reviewer for this helpful suggestion.

To ensure the Nora et al. heatmaps are positioned close to the genome tracks in Figure 1e, we reorganized the layout as follows:

- Figure 1d has been revised to exclusively display the Nora et al. heatmaps, positioning them directly above the tracks in panel e for easier comparison.
- The heatmaps for Rhodes et al. (Rad21-AID) and Liu et al. (Wapl-AID) have been moved to a new panel, Figure 1f, located below panel e.

We believe this restructured layout significantly improves the interpretability of the multi-omic data integration in this figure.

Figure 2: Could incorporate the panels currently in Supplemental Figure 6. This would clarify and simplify interpretation for the reader. I would consider including EP loops, H3K27me3 loops, and CTCF loops, with matrices of treatment/depletion overlaid. I think the manuscript generally lacked showing the underlying data. While this has improved, there is still room to expand this further.

As the reviewer suggested, we have now incorporated all panels in Supplemental Figure 6 into Figure 2d, with added heatmaps. These examples are included to show how we classified loops based on their epigenetic marks.

In addition, please note that the figure panels in Fig. 1e, 3f, 4h, 4j, 5b, 5e, and Supplementary Fig. 9a, 9b, 9d are all examples of FINs loci.

5. The Pcdh locus is interesting - could this be due to mis-regulation of Polycomb domain?

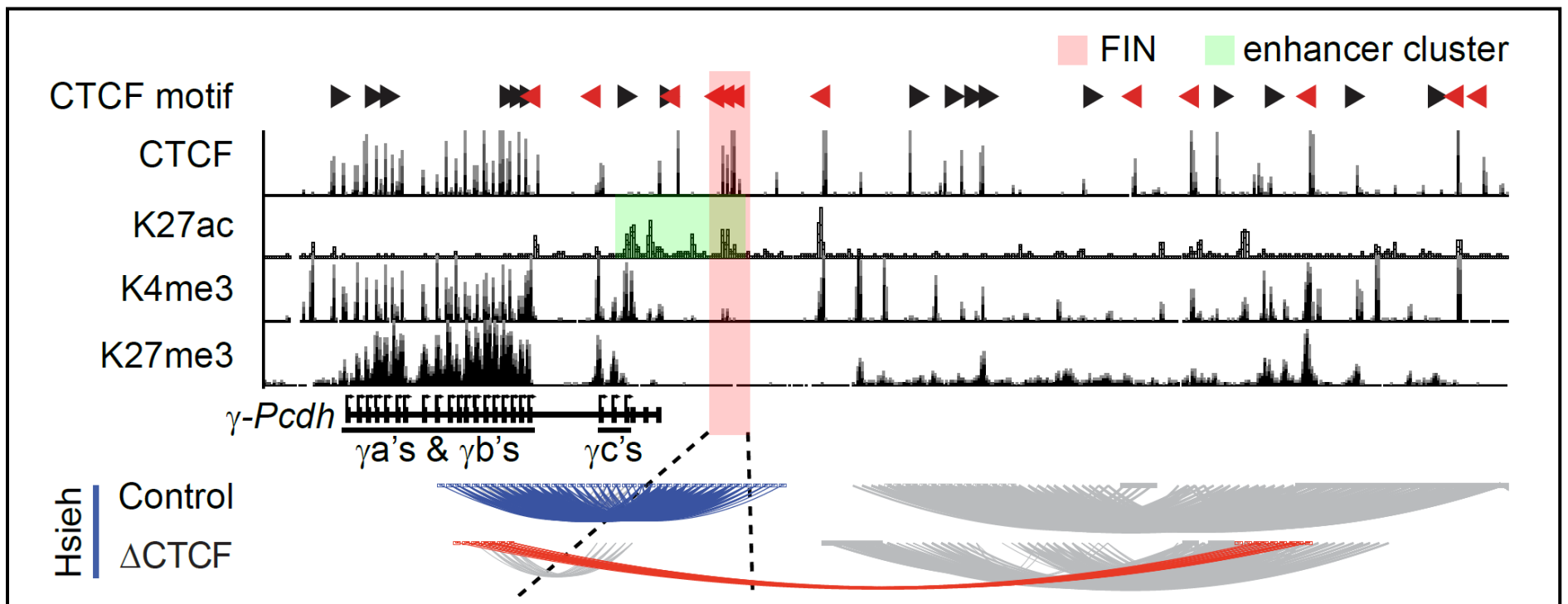

Reviewer Fig. 2. Many H3K27me3 peaks at Pcdh-γ locus form a domain-like structure. Note that this locus also have high density of H3K4me3 and CTCF peaks. Figure adopted from **Fig. 4j** in our manuscript.

[REDACTED]

Reviewer Fig. 3. A classic example of "polycomb domain" at HoxA locus. In motor neuron, the polycomb domain marked by H3K27me3 lacks H3K4me3 mark, and the genes (from HoxA7 to HoxA13) covered by this polycomb domain are all repressed. Figure adopted from Narendra et al., Science 2015, PMID:25722416.

We appreciate the reviewer's insight regarding the H3K27me3 enrichment at the *Pcdh* locus.

While the cluster of H3K27me3 peaks at the *Pcdh-γ* locus resembles a Polycomb-repressed domain, this locus is also marked by prominent H3K4me3 and CTCF peaks (**Reviewer Fig. 2**). Furthermore, the *Pcdh-γ* genes exhibit detectable transcription in wild-type mESCs (**Fig. 4l**). In contrast, canonical polycomb domains (e.g., the HoxA cluster, **Reviewer Fig. 3**) lack active H3K4me3 marks and remain transcriptionally silent. Therefore, we interpret the H3K27me3 peaks at *Pcdh-γ* genes as a cluster of "bivalent" or "poised" promoters (Bernstein et al., Cell 2006, PMID: 16630819) rather than a constitutive Polycomb-repressed domain.

Our data support a model of localized loop mis-regulation rather than a domain-wide epigenetic shift. Under baseline conditions, the *Pcdh-γ* genes interact with a nearby enhancer cluster (highlighted in green, **Reviewer Fig. 2**) that is spatially confined by a FIN (highlighted in pink, **Reviewer Fig. 2**). Following CTCF depletion, the loss of this FIN-mediated insulation disrupts these enhancer-promoter interactions, leading to the observed downregulation of

Pcdh-γ genes. This mechanism is directly supported by our experimental validation, in which the targeted deletion of the FIN resulted in the downregulation of *Pcdh-γ* genes (**Fig. 4k,l**).

6. Given the targets in the motif enrichment, I'm curious to whether any of these factors are differentially regulated upon CTCF/Rad depletion – in the depleted anchors for example, there is a considerable amount of homeobox/homeodomain containing factors.

This is an interesting question. In our manuscript (**Fig. 3l**), we show that loop-gaining ATAC-seq peaks are enriched with G-rich motifs and depleted of A/T-rich motifs (such as those for homeodomain factors). Our interpretation is that G-rich enhancers and promoters are intrinsically more likely to act as loop anchors upon CTCF depletion due to their superior capability to stall cohesin, assuming relatively stable expression of the associated transcription factors (TFs).

To address the review's question, we examined the expression changes of these putative TFs across three independent CTCF-depletion datasets (**Reviewer Fig. 4**). We found that none of the TFs associated with "enriched" motifs show more than a 2-fold change in any of the three datasets. While a few "depleted" TFs show changes exceeding 2-fold, these are often inconsistent across studies—for instance, *Gata5* is downregulated in the Nora et al. dataset but upregulated in Hsieh et al. (**Reviewer Fig. 4**). Additionally, although two TFs (*Foxd1* and *Cdx1*) are upregulated in two of the three studies, the magnitude of these changes is modest at slightly more than 2-fold. These observations suggest that TF expression fluctuations are unlikely to drive the loop rewiring observed in CTCF-depleted cells.

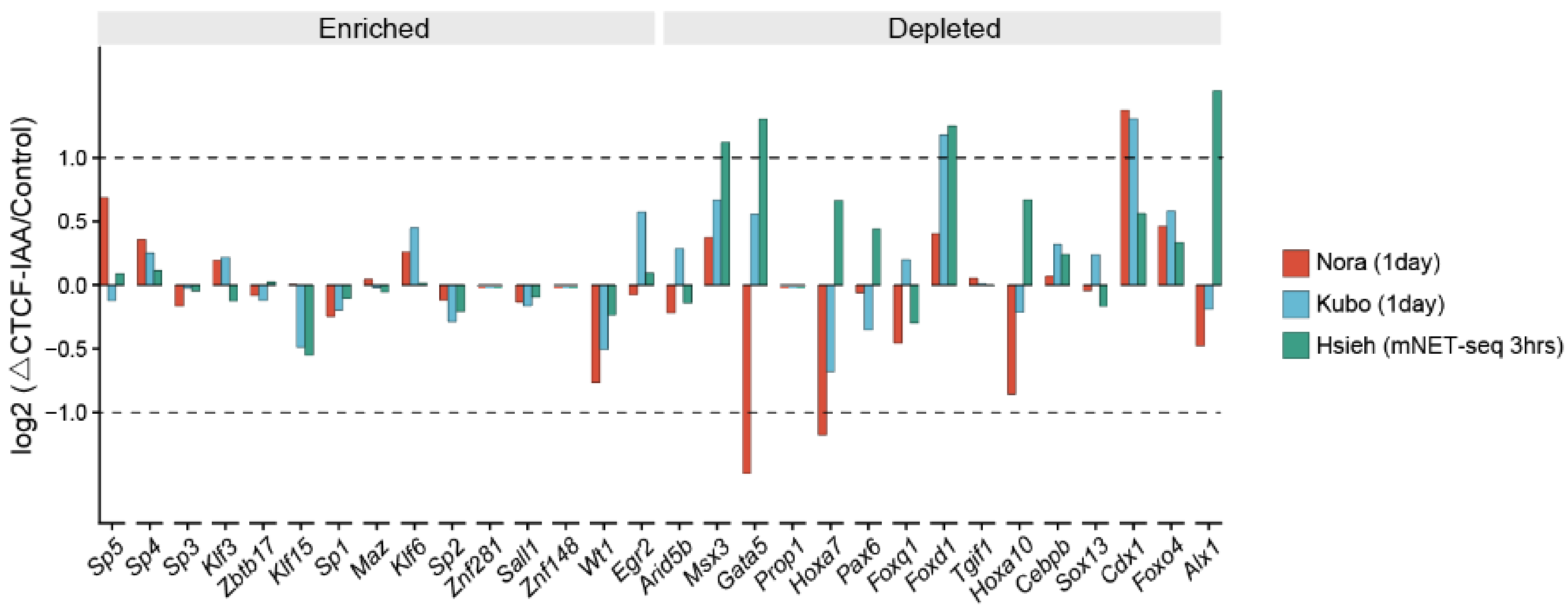

Reviewer Fig. 4. The expression changes of TFs across three CTCF-degron studies. Enriched/Depleted groups are the TFs listed in **Fig. 3l** that are enriched/depleted at loop-gaining ATAC-peaks upon CTCF-depletion.

nature portfolio

Peer Review File

Cross-platform Hi-C meta-analysis identifies functional insulators that actively block enhancer-promoter interactions

Corresponding Author: Dr Fulai Jin

This file contains all reviewer reports in order by version, followed by all author rebuttals in order by version.

Version 0:

Reviewer comments:

Reviewer #1

(Remarks to the Author)
Summary:

The paper entitled « Map functional insulators with cross-platform Hi-C meta-analysis » by Wanying Xu and colleagues describes a new computational approach to analyze chromatin conformation data, in particular Hi-C and micro-C across platforms, identifies putative functional insulators in the genome using this approach, and validates insulator loci experimentally at selected loci. The computational approach used for cross-platform data analysis primarily relies on the DeepLoop pipeline in order to identify loop interactions that are more robust across assays than using conventional methods such as ICE normalization. Applied on datasets from two published Hi-C studies and one micro-C study, DeepLoop identified retained and differential (lost or gained) loop interactions between control conditions and conditions in which either the mammalian insulator protein CTCF, the cohesin subunit RAD21, or the cohesin unloading factor WAPL were depleted. The differential loops are mostly consistent across assays for given conditions, and a subset of loops gained upon CTCF depletion bring together loop anchors that are characterized by epigenetic features associated with promoters and enhancers (cis-regulatory elements), specifically H3K4me3 and H3K27ac. The study hypothesizes that these loops represent newly established enhancer-promoter contacts, which were previously prevented or « blocked » by nearby CTCF-mediated contact points, and proposes the latter contact points as functional insulators (FINs). The study proposes a model of FIN function dependent on loop extrusion, whereby cohesin-mediated contacts between CTCF-bound anchor points at FINs prevent contacts and regulatory communication between cis-regulatory elements (e.g., an enhancer or group of enhancers, and the promoter of the target gene) that are separated by such FIN elements. Differentially expressed genes (DEGs) are enriched at gained loops and the DEGs that are most reproducible between studies tend to be located near FINs. Detailed examination at selected loci including Cdiptos and Pcdh-gamma shows loss of FIN-anchored interactions upon CTCF depletion that coincides with gain in interaction between distal H3K27ac-marked regions and promoter-proximal regions of DEGs. Interfering with CTCF binding using sgRNA/dCas9 at specific CTCF sites in selected candidate FIN regions result in a similar impact at the targeted loci than global CTCF depletion, including gene upregulation and gain of distal interactions of DEGs with H3K27ac-marked regions, thus validating the role of CTCF in the function of these specific FINs. The results of the study suggest that FIN are cis-acting insulators of enhancer-promoter communication in untreated cells.

Overall assessment:

The findings of the study are important for the fields of 3D genome and gene regulation, and could also have a broad impact on the scientific community in general, however there are major concerns with the presentation, analysis and interpretation of the results in the current state of the manuscript. At multiple points throughout the manuscript, some results are over-interpreted, hypotheses are not sufficiently tested to support the conclusions, and there is a lack of consideration for equally plausible alternative explanations to some of the findings. Cause-and-effect implications are often prematurely drawn in the absence of the required functional validation, resulting in missing evidence to support some of the main conclusions of the paper. In addition, the manuscript lacks essential yet basic analyses, such as TAD and compartment calling in the first section, misses or provides only cryptic informations about important analysis parameters (e.g., it is unclear how the overlap between TAD boundaries and FINs was assessed, and the main text lacks basic information about which epigenetic features were considered to identify putative E-P loops). For these reasons, I advise a major revision of the manuscript according to the point-by-point comments listed below, organized in chronological order with respect to the main text.

Point-by-point comments:

RESULTS

(Major) Line 78: The statement that « for Hi-C and in situ Hi-C studies, conventional ICE-normalization only support compartment- or TAD-level analysis at 20kb or lower resolution due to the lack of read depth » lacks supporting evidence from TAD and compartment calling.

(Major) Line 83: In addition to visual inspection at selected examples of chromosomal regions, the robustness of DeepLoop in cross-platform analysis should also be supported by correlating the DeepLoop signal (e.g., pixel intensity) for the same region pairs between assays, and by contrasting this correlation to the those obtained with standard ICE normalization or HiCorr correction only.

Fig. 1d: Y-axis labels should indicate « loop strength » after « Control » and « deltaCTCF-IAA » conditions.

(Major) Fig. 1e,f: The epigenetic features of promoter and enhancers used to define E-P loops should be summarized directly in the result section. This information is currently only present in the method section.

For contact heatmaps, the meaning of the numbers between brackets should be should be indicated in the figure legend.

For plots of loop size, the figure legend should explicitely indicate that the median loop size is given underneath the plot for each loop categories.

Line 121: For more clarity, modifying the sentence as follows is recommended: « some of them lose strength [upon CTCF degradation] in Hsieh et al. …» .

(Major) Line 126: The data presented are not sufficient to conclude that « Hsieh et al. achieves the best CTCF depletion, perhaps because it uses N-terminal degron system ». Assay-specific biases, differences in sensitivity and in the depth of sequencing could also explain at least some of the discrepancies observed between studies.

Line 132: For more accuracy, modifying the sentence should be modified as follows « Nevertheless, [most of] the "gained pixels" from Nora et al. … ».

Line 138: It is not clear what is meant by « affected genes … are likely to be the direct targets of insulators ». How/why are the affected genes likely to be the targets of insulators?

(Major) Line 143: About the observation that « "retained" and "gained" loop pixels in Rad21-depleted cells are much smaller (mostly within 100kb) than those in CTCF-depleted cells, indicating that cohesin-independent loops are short-range », it is also conceivable that decreasing the pool of available cohesin through depletion of RAD21 allows for only rare and short-lived extrusion events (assuming unchanged levels of the cohesin unloader WAPL upon RAD21 depletion compared to untreated cells), thereby creating smaller-sized, yet still cohesin-dependent loops. The above possibility should be acknowledged in the manuscript.

Line 151: It is not clear what is « the conclusion from the original study ».

Line 147: For accuracy, the following changes are recommended: « The "retained" and "gained" loop pixels in Kriz et al. and Hsieh et al., and "retained" loop pixels in Rhodes et al. In Rad21-depleted cells… »

(Major) The use of the terms « dependent » and « independent » (e.g.,whole paragraph starting line 165) with respect to CTCF or cohesin should be avoided given the possibility that residual amount of these factors could be present in the depleted cells, and the observation that, for example, some loops retained upon CTCF depletion display CTCF peaks at both anchors (44% of retained loops in Nora et al.) and are hence likely to be CTCF-dependent. The use of the terms « retained », « gained » or « lost » upon certain conditions should be favored for accuracy.

(Major) Lines 172-175: The conclusion that « recurrently gained E-P interactions upon CTCF depletion are created by loop extrusion » because « "gained" loops in CTCF-depleted cells do not gain strength in Rad21-depleted cells » seems far-fetched, because these loops also do not show clear loss of strength upon RAD21 depletion (or this result is not properly presented and discusssed), which would be expected if they are created by loop extrusion.

Line 177: « Supp Fig. 5a, b, third row » is mislabelled as « Supp Fig. 5b, d, third row ».

(Major) Fig. 3b: It is unclear how the FIN loci were identified, e.g. how many CTCF sites or loops are required to define such loci, and within regions of what size? This is especially important as FIN loci as identified at this stage then become the focus of the rest of the study (e.g., red highlight in Fig. 4h,j).

(Major) Line 202: It is not clear how the overlap between TAD boundaries and FINs was analysed. Was it perfect overlap

only, or overlap allowing for certain shifts? This is important as TAD and boundary calling is highly dependent on what algorithm (e.g., based on switch in contact directionality, or based on local dips of contact density) and which parameters (e.g., bin size) were used.

Fig. 4d: Acute CTCF depletion or degradation is not equivalent to a « KO » (genetic loss of function) condition, and therefore should not be abbreviated as such. Along the same line, control cells should also not be abbreviated « WT ».

Line 228: The statement that « Using NET-seq, Hsieh et al. identified more DEGs than RNA-seq despite shorter time of CTCF depletion (3hr) » is inconsistent with the data presented in Fig. 4e, showing that more DEGs are identified in Nora et al. (213 upreg, 229 downreg) compared to Hsieh et al. (174 upreg, 168 downreg).

(Major) Line 241: The 49 DEGs at FIN loci are only potential or putative direct targets of insulators. Language should be adjusted accordingly.

(Major) Line 256: While the gain in interactions with the distal H3K27me3-marked region might explain Pcdh-gamma gene downregulation, further investigation of the locus, e.g. by deleting the distal H3K27me3-marked region, is required to validate that interactions with the distal H3K27me3-marked region does indeed cause Pcdh-gamma gene downregulation. In the present situation, only an association or coincidence may be claimed, but no cause-and-effect relationship.

Line 272 and Fig. 5c: While the text refers to CUT&RUN of CTCF, the axis label in Fig. 5c indicates CTCF ChIP-seq.

(Major) Line 279: The sentence should describe the gained loop interactions more explicitly: which regions do they connect? What are the characteristics of the connected regions? In addition, the end of the sentence stating that Mesp1 is upregulated « SINCE the new loops bring distal enhancers to its proximity » requires functional validation, typically by targeted deletion of the putative enhancer regions.

Line 281: The sentence should be toned down to « strongly suggest » (that FINs execute their insulating functions in cis) or similar, without further evidence with allele-specific CTCF perturbation and allele-specific gene expression measurements.

DISCUSSION

Line 293: Mentioning that « most TAD boundaries do not gain cross-boundary loops upon CTCF depletion » to contrast them with FINs is highly confusing, because neither do most FINs gain loops in the same condition. Instead, new loops may be gained between other regions that were previously blocked by FINs. This point needs to be clarified to avoid any confusion, especially between FINs and the gained putative E-P interactions.

(Major) Line 304: The sentence is not sufficiently supported by data, as explained in the comment on lines 172-175 above.

Line 306: The sentence describes a very weak and unconvinving evidence for cohesin dependency, through localization of FINs almost essentially in compartment A.

(Major) Without functional validation, E-P loops in this study should be considered as putative, as the enhancers' activity and capacity to regulate the considered genes were not formally assessed.

METHODS

(Major) Line 639: What is meant by « For TADs and their boundaries, we directly use the TADs in mESCs defined by Dixon et al. »? Was the procedure re-used, or the same actual TAD and boundary calls were used? In the latter case, the use of TAD and boundary calls from a different study, Hi-C protocol and mESC line (J1 mouse ES cells in Dixon et al.) could readily explain the poor overlap between FINs and TAD boundaries. To ensure comparability, TADs/boundaries and FINs should be called from the exact same datasets.

(Remarks on code availability)

Reviewer #2

(Remarks to the Author)
Map Functional Insulators with Cross-Platform Hi-C Meta-Analysis

General Thoughts:
Xu et al. characterize functional insulating regions of the genome (termed FINs) that contribute to gene regulation. They begin by analyzing multiple Hi-C and Micro-C datasets involving CTCF, Rad21, or Wapl depletions (Figure 1), using HiCorr and DeepLoop to characterize loop dynamics and identify gained loops upon CTCF depletion. They extend this analysis to other depletion datasets and compare loop dynamics across CTCF, Wapl, and Rad21 perturbations (Figure 2).
FINs are defined insulating regions where de-novo loops form between CREs upon CTCF depletion and are categorized

into three types based on CTCF presence (Figure 3). They further link these regions to expression changes and describe new loop dynamics at FIN-containing regions (Figure 4). Finally, they use a dCas9 system to functionally validate selected FINs by targeting CTCF sites and observing transcriptional changes at loci such as Cdiptos and Mesp1 (Figure 5).
This study addresses an important question: why perturbations to chromatin conformation have profound effects in some genomic contexts but not others. While the authors attempt to define FINs in mESCs, the work would benefit from a deeper investigation into what makes an insulating region functional. Additionally, the figures often feel incomplete—more extensive display of the Hi-C/Micro-C matrices would add clarity to the results. Although the authors leverage multiple dataset, the cross-study comparisons are at times underutilized. Overall this work would benefit by further analysis into the FINs they define.

Major comments:
Figure 1: The signal for Rhodes et al. seems very sparse. It would be helpful to show how these regions appear across other datasets, not just in CTCF depletion. Additionally, the general comparisons between datasets are limited to coverage metrics and a few loci. Performing PCA or a similar analysis on the different Hi-C/Micro-C experiments would better contextualize discrepancies between results and substantiate claims about differential depletion across studies.

It's a bit unclear what a pixel represents—I'm assuming one bin? What is the bin size, 5kb? These were then merged into regions down the line, but this is also not clearly explained as to how, or what exactly that means.

I wonder if the longer gained loops being captured in Rhodes et al. (Supp Fig 3 – lines 150) are seemingly longer because the data is too scarce to capture short range loops that might need more coverage to be visible regardless of bias correction or DeepLoop.

In lines 192-196, the term CTCF loop is brought up then switched to just insulation while this neither are really shown, more than just CTCF sites. It would be good to have an example region for each type of loop. TADs are also inconsistently discussed but not shown. Moreover, since TAD-calling can be noisy, more insulation score analysis at FIN vs non-FIN sites could strengthen the work.

This paper generally lacks in depth characterization of FIN's. If there is ATAC-seq, for example, footprinting analysis would be interesting even in just wildtype mESC, to ask if there are any specific motifs or promoter types enriched at these regions which are being functionally insulated in mESCs? Moreover, Are these FINs dynamic across cell types, or stable but functionally relevant only in specific developmental contexts? Some of this is touched on in the discussion but could at least be looked at by analyzing a none-mESC Hi-C or Micro-C dataset.

For Figure 3g: How does a FIN at a non-TAD boundary looks like, is it just because a TAD-call is not there or is it because this region shows no insulation (which is then a bit perplexing). While the schematics are helpful, real data should be shown. I would think that would be much more important for a main figure as a sanity check to the classifications than panel d for example.

Many of the figures need much better background and Supplemental to back up the claims being made. Few examples:
1. A bit more about the enhance models, since these were specific to each experiment.
2. Classification of chromatin loops based on the epigenetic data, it would be helpful to see more examples of loop pixels within each group, and to see what "other" is, is it noisy regions of the genome for example or just regions with no CHIP coverage?
3. A Supplement for Figure 4 could have easily incorporated the Hi-C maps between controls and degradation experiments without DeepLoop models, are they still visible?
4. How was the RNA-seq normalized and how does that affect DEGs. Are ERCC spike-ins better than using empirical genes in RUVseq for example?

Figure 4f isn't clear Also, switching between Nora/Kudo and Hsieh datasets across subpanels (f–j) is confusing.

The functional validation experiments are nice. However, matrix views of the targeted regions should be shown in addition to the DeepLoop model outputs. I think it would be also interesting to do this on a down regulated gene.

Minor comments:
The title needs improvement.

Supplemental Figures 3 and 4 should be made into a single supplementary figure.

Line 180 ("more analysis below") can be deleted.

Figure 4c could be moved to the supplemental section; the plot is confusing and adds limited value.

For Figure 4e, adding NET-seq data next to Hsieh's data and RNA-seq results for other experiments would make comparisons easier.

In Line 232, it's overstated that "most" DEGs overlap between Kudo et al. and Nora et al.—especially considering upregulated genes.

(Remarks on code availability)
The code is clear and well annotated, the main issue is that the basic descriptions in the paper is lacking.

Version 1:

Reviewer comments:

Reviewer #1

(Remarks to the Author)
All my previous points have been satisfactorily addressed and I recommend the manuscript for publication.

(Remarks on code availability)

Reviewer #2

(Remarks to the Author)
Revision 2
Cross-platform Hi-C meta-analysis identifies functional insulators that actively block enhancer-promoter interactions
Cui, Xu, Lang et al.

Summary:
The authors have added substantial new analysis and some experiments to the work. They have also improved the overall discussion of their results, which initially contained too much over-interpretation with limited support, as also noted by Reviewer #1.
While the manuscript has improved, figure presentation for easier interpretability can still be improved.

Minor Points:
1. Sentence 44-46:
"TADs disappear upon loss.....loop extrusion" could be rewritten to be a bit more readable.
2. Section 116-121
Current text:
"Only the matrices from HiCorr and DeepLoop clustered CTCF- and Rad21-degron datasets together and Wapl-degron datasets are separated out (Fig. 1c, Supplementary Fig. 3). This is consistent with the knowledge that depletion of CTCF or Rad21, but not Wapl, causes massive loss of TADs and CTCF loops. Taken together, these results support the robustness of DeepLoop in cross-platform analysis regardless of read depth."
I think it would be worth noting that both HiCorr and DeepLoop signals appear to saturate toward looping (as one would expect of DeepLoop for example). In contrast, ICE/ICE+distance correction may retain additional or broader structural features, which could explain the lack of clustering/correlation. Clarifying this would strengthen the interpretation.
3. Figure interpretability:
I think a simple heatmap (as in deeptools) of different tracks (H3K27ac, H3K27me3, RNA-seq, etc.) at loop anchors of FINs (perhaps even separated by class type) could be useful to visually summarize the features associated with dynamic loops.
I appreciate the new analysis in Supplemental Figure 1. I do think a simple heatmap showing insulation at gained, lost, and maintained anchors would be interesting. This could help clarify whether these are regions that are already prepped but blocked, or if it's a mix of multiple dynamics including de novo gain of regulation.
I would suggest using anchors rather than merged regions, as otherwise there may be too few regions and mixed sizes - but not sure.
4. Figure Organization:
Figure 1: I would move Nora et al. to the bottom to be closer to the tracks in panel e.
Figure 2: Could incorporate the panels currently in Supplemental Figure 6. This would clarify and simplify interpretation for the reader.
I would consider including EP loops, H3K27me3 loops, and CTCF loops, with matrices of treatment/depletion overlaid.
I think the manuscript generally lacked showing the underlying data. While this has improved, there is still room to expand this further.
5. The Pcdh locus is interesting - could this be due to mis-regulation of Polycomb domain?
6. Given the targets in the motif enrichment, I'm curious to whether any of these factors are differentially regulated upon CTCF/Rad depletion – in the depleted anchors for example, there is a considerable amount of homeobox/homeodomain containing factors.

(Remarks on code availability)
I have partially looked at the code, but I have not re-run or done in depth line by line review.

Open Access This Peer Review File is licensed under a Creative Commons Attribution 4.0 International License, which permits use, sharing, adaptation, distribution and reproduction in any medium or format, as long as you give appropriate credit to the original author(s) and the source, provide a link to the Creative Commons license, and indicate if changes were made.
In cases where reviewers are anonymous, credit should be given to 'Anonymous Referee' and the source.
The images or other third party material in this Peer Review File are included in the article's Creative Commons license, unless indicated otherwise in a credit line to the material. If material is not included in the article's Creative Commons license and your intended use is not permitted by statutory regulation or exceeds the permitted use, you will need to obtain permission directly from the copyright holder.
To view a copy of this license, visit https://creativecommons.org/licenses/by/4.0/

We sincerely thank both reviewers for their time and efforts. Their constructive suggestions have significantly improved our manuscript. As detailed below, we have added new experiments, new data analysis, new figures, and modified the text to strengthen our points and clarify our findings. We believe we have addressed all reviewer's comments and concerns. The following are the major changes:

- A new title.
- One new validation experiment (**Fig. 4k,l**) in which we show downregulation of Pcdh-γ genes upon deletion of a putative functional insulator (FIN).
- A new **Supp. Fig. 1** in which we compared the compartment A/B and TADs across three CTCF-degron Hi-C datasets.
- A new section in the main text with new figures **Fig. 1b-c** and **Supp. Fig. 2-3** demonstrating that DeepLoop achieves best consistency in cross-platform Hi-C loop analysis.
- A comprehensive TAD-boundary analysis using control mESC data from all three CTCF-degron study with both "Directionality index" and "Insulation score" measurement. We now have more convincing results showing that regardless of datasets or algorithm, low resolution TAD-level features cannot represent the FINs we identified from high-resolution dynamic loop analysis. These results are shown in the new **Fig. 3f,h,l** and **Supp. Fig. 11**.
- An interesting new motif analysis results in **Fig. 3l** showing that G-rich cis-regulatory elements are more likely to act as new loop anchors upon CTCF-depletion.
- A new **Supp. Fig. 8** in which we show two examples that although Rhodes *et al.* showed gain of long-range interactions between H3K27me3-occupied regions upon Rad21-depletion, the long-range H3K27me3 loops already exist in the control cells from Kriz *et al.* and Hsieh *et al.* before *Rad21*-depletion. This finding argues against the conclusion of Rhodes *et al.* that cohesin prevents the formation of long-range H3K27me3 loops.
- We also added many new contact heatmaps or examples as the reviewers requested in **Fig. 5b,e**, **Supp. Fig. 4g,h**, **Supp. Fig. 6**, **Supp. Fig. 10**, **Supp. Fig. 12**.

In the main text file, we have highlighted major changes in yellow. All changes are also explained in the point-by-point responses to the reviewers' comments. The reviewer's comments are in black and our response in blue.

Reviewer #1 (Remarks to the Author)

Summary:

The paper entitled « Map functional insulators with cross-platform Hi-C meta-analysis » by Wanying Xu and colleagues describes a new computational approach to analyze chromatin conformation data, in particular Hi-C and micro-C across platforms, identifies putative functional insulators in the genome using this approach, and validates insulator loci experimentally at selected loci. The computational approach used for cross-platform data analysis primarily relies on the DeepLoop pipeline in order to identify loop interactions that are more robust across assays than using conventional methods such as ICE normalization. Applied on datasets from two published Hi-C studies and one micro-C study, DeepLoop identified retained and differential (lost or gained) loop interactions between control conditions and conditions in which either the mammalian insulator protein CTCF, the cohesin subunit RAD21, or the cohesin unloading factor WAPL were depleted. The differential loops are mostly consistent across assays for given conditions, and a subset of loops gained upon CTCF depletion bring together loop anchors that are characterized by epigenetic features associated with promoters and enhancers (cis-regulatory elements), specifically H3K4me3 and H3K27ac. The study hypothesizes that these loops represent newly established enhancer-promoter contacts, which were previously prevented or « blocked » by nearby CTCF-mediated contact points, and proposes the latter contact points as functional insulators (FINs). The study proposes a model of FIN function dependent on loop extrusion, whereby cohesin-mediated contacts between CTCF-bound anchor points at FINs prevent contacts and regulatory communication between cis-regulatory elements (e.g., an enhancer or group of enhancers, and the promoter of the target gene) that are separated by such FIN elements. Differentially expressed genes (DEGs) are enriched at gained loops and the DEGs that are most reproducible between studies tend to be located near FINs. Detailed examination at selected loci including Cdiptos and Pcdh-gamma shows loss of FIN-anchored interactions upon CTCF depletion that coincides with gain in interaction between distal H3K27ac-marked regions and promoter-proximal regions of DEGs. Interfering with CTCF binding using sgRNA/dCas9 at specific CTCF sites in selected candidate FIN regions result in a similar impact at the targeted loci than global CTCF depletion, including gene upregulation and gain of distal interactions of DEGs with H3K27ac-marked regions, thus validating the role of CTCF in the function of these specific FINs. The results of the study suggest that FIN are cis-acting insulators of enhancer-promoter communication in untreated cells.

Overall assessment:

The findings of the study are important for the fields of 3D genome and gene regulation, and could also have a broad impact on the scientific community in general, however there are major concerns with the presentation, analysis and interpretation of the results in the current state of the manuscript. At multiple points throughout the manuscript, some results are over-interpreted, hypotheses are not sufficiently tested to support the conclusions, and there is a lack of consideration for equally plausible alternative explanations to some of the findings. Cause-and-effect implications are often prematurely drawn in the absence of the required functional validation, resulting in missing evidence to support some of the main conclusions of the paper. In addition, the manuscript lacks essential yet basic analyses, such as TAD and compartment calling in the first section, misses or provides only cryptic informations about important analysis parameters (e.g., it is unclear how the overlap between TAD boundaries and FINs was assessed, and the main text lacks basic information about which epigenetic features were considered to identify putative E-P loops). For these reasons, I advise a major revision of the manuscript according to the point-by-point comments listed below, organized in chronological order with respect to the main text.

We really appreciate the reviewer's comprehensive summary and careful review of our work. We also thank the reviewer for recognizing our work's potential to have "a broad impact on the scientific community in general". Below we have carefully addressed the reviewer's point-by-point comments. Following the reviewers' suggestions, we have added new experiments and analyses into the revised manuscript.

Point-by-point comments:

RESULTS

(Major) Line 78: The statement that « for Hi-C and in situ Hi-C studies, conventional ICE-normalization only support compartment- or TAD-level analysis at 20kb or lower resolution due to the lack of read depth » lacks supporting evidence from TAD and compartment calling.

We have performed compartment- and TAD-level analysis using conventional pipelines on the three CTCF-AID datasets. The results are shown in a new **Supp. Fig. 1**.

At compartment level (500kb resolution), in all three datasets the contact heatmaps and PC1 profiles (compartment A/B) are largely unchanged after CTCF-depletion (**Supp. Fig. 1a**), and this conclusion is further supported by scatter plots of PC1 values showing the high correlation between control and △CTCF cells within each of the studies (R-square 0.820~0.907, **Supp. Fig. 1b**). These results indicate that the compartmental organization is largely preserved after CTCF depletion, consistent with the original studies. In cross-platform comparisons, we found that the heatmaps are quite similar although the R-square values are slightly lower (0.763~0.791 for control cells and 0.777~0.799 in △CTCF cells, **Supp. Fig. 1c**). These results support the robustness of compartment analysis at low-resolution across different Hi-C protocols.

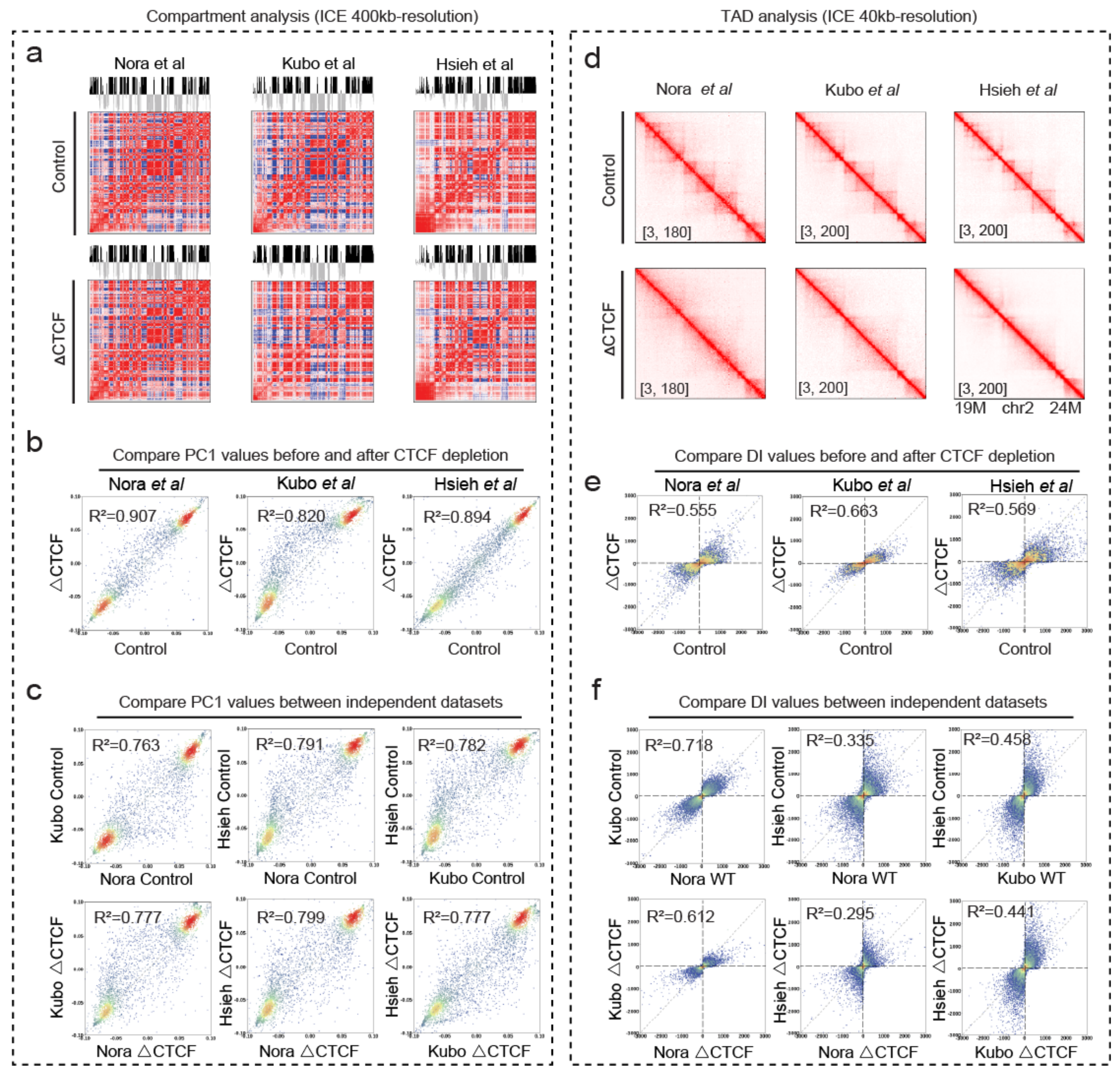

Supp. Fig. 1. Compartment and TAD analysis of the three independent CTCF-depletion studies.

At the TADs level (40kb resolution), the loss of TADs upon CTCF depletion is also obvious in all studies (**Supp. Fig. 1d**), consistent with the original reports. Loss-of-TAD is also reflected in scatter plots of DI (directionality index) of all 40kb bins, in which the DI values of many bins become close to zero upon CTCF-depletion (**Supp. Fig. 1e**). The heatmaps of Nora et al. and Kubo et al. are slightly noisier than Hsieh et al. (**Supp. Fig. 1d**). We also compare the DI values between independent HiC/in situ Hi-C/micro-C studies (**Supp. Fig. 1f**): although the R-squares between the Hi-C and in situ Hi-C (Nora et al. and Kubo et al.) are rather high (R-square 0.718 and 0.612 for control and △CTCF cells), the DI values from micro-C data (Hsieh et al.) deviate significantly from the other two studies (R-square 0.295~0.458). In micro-C data, more bins show polarized DI values indicating stronger directionality biases. This is consistent with the general belief that with ICE-based pipeline, micro-C identifies more loops than Hi-C or in situ Hi-C.

Taking together, these analyses confirmed the lack of compartment changes and the loss of TADs upon CTCF-depletion. However, the commonly used scores (PC1 or DI) from standard ICE pipelines show variation between independent studies. The platform variation in TAD-level analysis is higher than compartment level analysis.

At loop level (5kb resolution), the variation between independent studies is much bigger. We will discuss below in response to the reviewer's next comment.

(Major) Line 83: In addition to visual inspection at selected examples of chromosomal regions, the robustness of DeepLoop in cross-platform analysis should also be supported by correlating the *DeepLoop* signal (e.g., pixel intensity) for the same region pairs between assays, and by contrasting this correlation to the those obtained with standard ICE normalization or HiCorr correction only.

We thank reviewer for the suggestion. We added three new figures in the revision demonstrating this point:

A. Supplementary Figure 2.

We added a massive new **Supp. Fig. 2** to compare the performance of ICE, ICE with distance correction, HiCorr, and DeepLoop in quantifying loop strength with all datasets. Part of **Supp. Fig. 2** (only for CTCF-degron datasets) are shown below. In this figure we included: (i) example heatmaps at 5kb-resolution; (ii) scatter plots comparing control cell heatmaps from independent studies to evaluate each pipeline's consistency in cross-platform analysis; (iii) scatter plots comparing each pair of heatmaps before and after protein depletion. For (ii) we only compare control cell data because before protein depletion, the control cells from all studies are expected to be similar at the stem cell state. Protein-depleted cells from independent studies are biologically more diverse due to many reasons such as time-span of protein depletion, incomplete protein depletion, loss of stemness, etc.

When resolution increases to 5kb, the contact heatmaps from ICE (with or without distance correction) and HiCorr are highly variable between independent datasets. There are more noises in Hi-C and *in situ* Hi-C heatmaps than in micro-C heatmaps (**Supp. Fig. 2a,d,g**). However, the heatmaps from *DeepLoop* are much more consistent between platforms because DeepLoop enhances loop pixels and removes most of the noise pixels (**Supp. Fig. 2j**).

For cross-platform consistency analysis in control cells, we found that although conventional ICE pipelines show poor cross-platform consistency with R-square values often close to zero, HiCorr and DeepLoop clearly improved the consistency with much higher cross-platform R-square values (**Supp. Fig. 2b,e,h,k**).

The comparisons between control and degron cells also support the cross-platform consistency. For example, if we use ICE without distance-correction to analyze the micro-C data by Hsieh *et al.*, △CTCF cells have many pixels stronger than control cells (**Supp. Fig. 2c** below), which is opposite to the knowledge that CTCF-depletion leads to loss of loops. This is because the read-depth of micro-C data in △CTCF cells is higher than in control cells (830M vs. 291M). If we perform distance correction after ICE-normalization, which generates observed/expected ratio heatmaps. The control vs. △CTCF scatter plot for Hsieh *et al.* correctly shows more loop pixels in control cells (**Supp. Fig. 2f**). However, the scatter plots from ICE-based pipelines lack pixels that retain strength upon CTCF-depletion, which is in sharp contrast to HiCorr and DeepLoop scatter plots, in which the retained pixels are clearly recognizable (**Supp. Fig. 2i, l**).

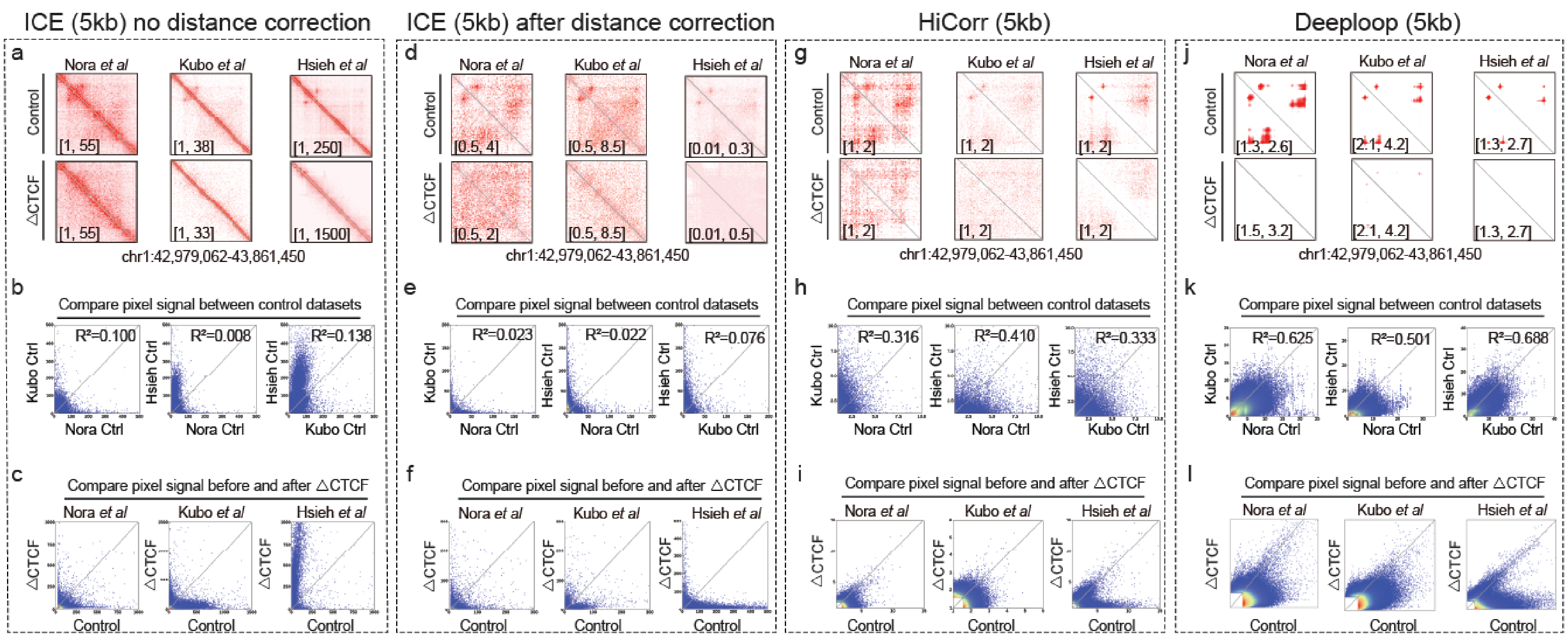

Supp. Fig. 2. Loop-level analysis (5kb resolution) of the three CTCF-degron studies with ICE (with or without distance correction), HiCorr, and DeepLoop.

B. Figure 1b.

In the main figure, we also added **Fig. 1b** (shown on the right) to summarize the results about cross-platform consistency as discussed above in **Supp. Fig. 2**. We found that when applying different pipelines to control mESC data from all datasets, DeepLoop achieves the best cross-platform consistency between orthogonal contact heatmaps.

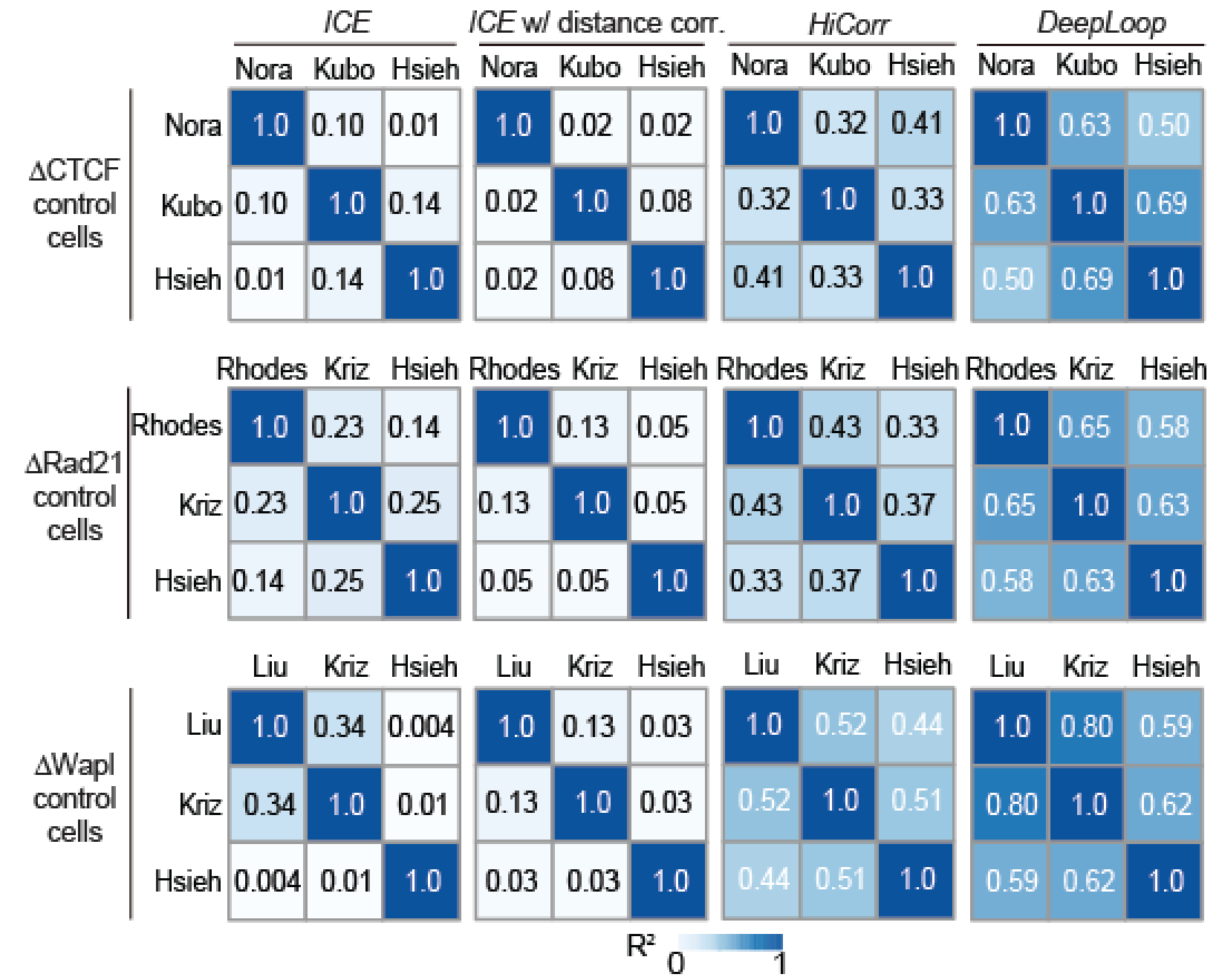

Fig. 1b. R-square matrices comparing Hi-C heatmaps in control mESC cells from different datasets when analyzed by different pipelines.

C. Supplementary Figure 3 and Figure 1c.

We also added a new **Supp. Fig. 3** showing the cross-platform consistency when applying different pipelines to analyze dynamic loops. In this analysis we first generate differential heatmaps at 5kb resolution by subtracting each pair of contact heatmaps (depletion - control), then compute the correlations between the differential heatmaps from all nine pairs of degron experiments. Only *HiCorr* and *DeepLoop* cluster CTCF- and Rad21-degron datasets together, and Wapl-degron datasets are separated (**Supp. Fig. 3**). This is consistent with the knowledge that depletion of either CTCF or Rad21, but not Wapl, causes massive loss of TADs and CTCF loops. **Fig. 1c** in the main figure includes two panels of **Supp. Fig. 3** (panel **a** and **d**).

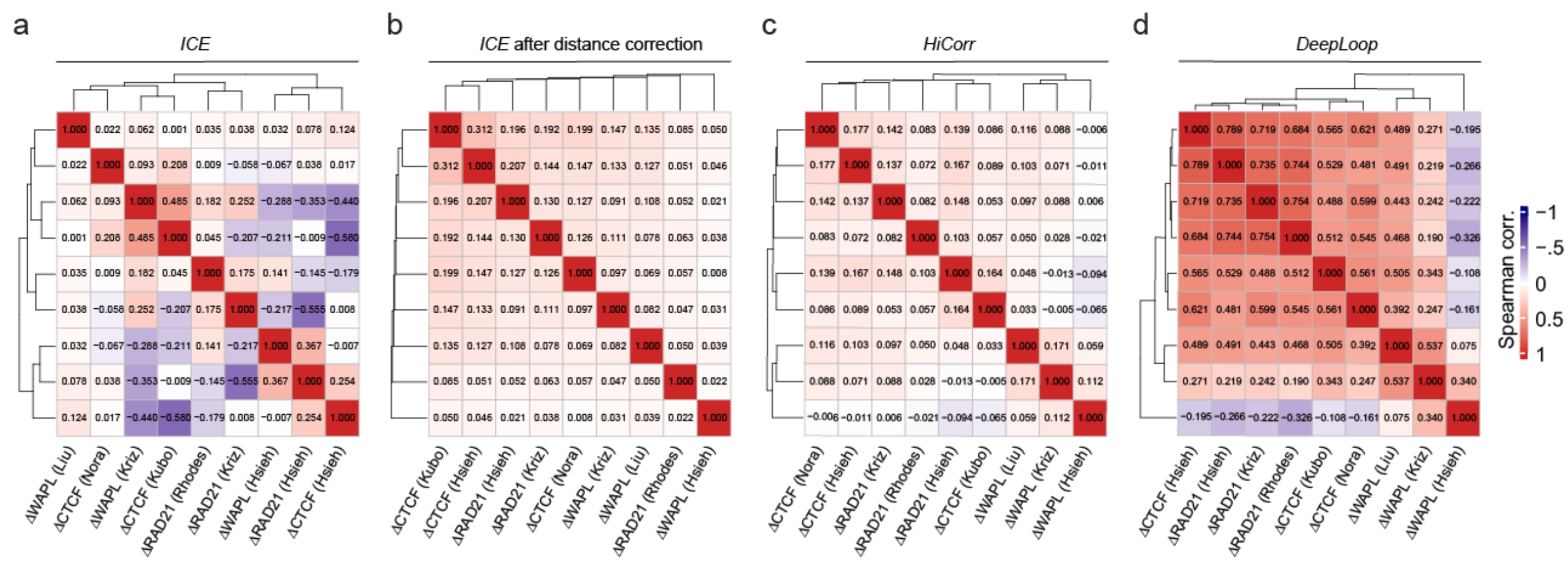

Supp. Fig. 3. Correlation matrices comparing the differential contact heatmaps at 5kb resolution from all nine pairs of degron experiments.

Taking together, all these results support the robustness of DeepLoop in cross-platform analysis.

Fig. 1d: Y-axis labels should indicate « loop strength » after « Control » and « deltaCTCF-IAA » conditions.

The old **Fig. 1d** is now **Fig. 2a**.

We have updated the labels in **Fig. 2a** and other figure panels as the reviewer suggested.

(Major) Fig. 1e,f: The epigenetic features of promoter and enhancers used to define E-P loops should be summarized directly in the result section. This information is currently only present in the method section.

The old **Fig. 1e,f** is now **Fig. 2b,c**.

We have now summarized the epigenetic features used to define the E-P loops in the results section as the reviewer suggested. We also added a new **Supp. Fig. 6** showing examples of EP-loop, CTCF-EP loop, CTCF loop, H3K27me3 loop, and H3K36me3 loop.

For contact heatmaps, the meaning of the numbers between brackets should be indicated in the figure legend.

We have revised the figure legend for the contact heatmaps to clarify the meaning of the numbers shown in brackets, which refer to the scale of heatmaps.

For plots of loop size, the figure legend should explicitly indicate that the median loop size is given underneath the plot for each loop categories.

We have revised the figure legend of **Fig. 2a** to explicitly state that the median loop size is shown below each plot for the corresponding loop category.

Line 121: For more clarity, modifying the sentence as follows is recommended: « some of them lose strength [upon CTCF degradation] in Hsieh et al. …»

We have modified this sentence as recommended (line 160-161).

(Major) Line 126: The data presented are not sufficient to conclude that « Hsieh et al. achieves the best CTCF depletion, perhaps because it uses N-terminal degron system ». Assay-specific biases, differences in sensitivity and in the depth of sequencing could also explain at least some of the discrepancies observed between studies.

We agree and have revised the text to tune down the conclusion and acknowledge these potential confounding factors (line 166-169)

Line 132: For more accuracy, modifying the sentence should be modified as follows « Nevertheless, [most of] the "gained pixels" from Nora et al. … ».

We have revised this sentence as recommended (line 174).

Line 138: It is not clear what is meant by « affected genes … are likely to be the direct targets of insulators ». How/why are the affected genes likely to be the targets of insulators?

We have revised this statement down to:

*"…affected genes such as Notch1 in **Fig. 1** are putative target genes of CTCF-mediated insulation."* (line 179-180)

(Major) Line 143: About the observation that « "retained" and "gained" loop pixels in Rad21-depleted cells are much smaller (mostly within 100kb) than those in CTCF-depleted cells, indicating that cohesin-independent loops are short-range », it is also conceivable that decreasing the pool of available cohesin through depletion of RAD21 allows for only rare and short-lived extrusion events (assuming unchanged levels of the cohesin unloader WAPL

upon RAD21 depletion compared to untreated cells), thereby creating smaller-sized, yet still cohesin-dependent loops. The above possibility should be acknowledged in the manuscript.

We agree and thank the reviewer for the insights. In the revision we have now acknowledged this alternative explanation. (line 189-191)

Line 147: For accuracy, the following changes are recommended: « The "retained" and "gained" loop pixels in Kriz et al. and Hsieh et al., and "retained" loop pixels in Rhodes et al. In Rad21-depleted cells… »

We thank the reviewer for the suggestion and have revised the sentence to exclude the "gained" loop pixels in Rhodes et al. (line 186-187)

Line 151: It is not clear what is « the conclusion from the original study ».

We apologize for the ambiguity and have revised this sentence to explicitly state the conclusion of Rhodes *et al.*: "…cohesin removal enhances long-range interactions between a subset of polycomb-occupied regions…" (line 198-199)

We want to add that in this revision we have examined the "gained" H3K27me3 loops in Rad21-depleted cells from Rhodes et al. and found that in almost all cases, the "gained" long-range H3K27me3 loops are already present in the control cells from Kriz *et al*. and Hsieh *et al.* before Rad21 depletion (two examples are shown in a new **Supp. Fig. 8**). Therefore, at least in the control mESCs from Kriz et al. and Hsieh et al., cohesin does not prevent the formation of long-range H3K27me3 loops.

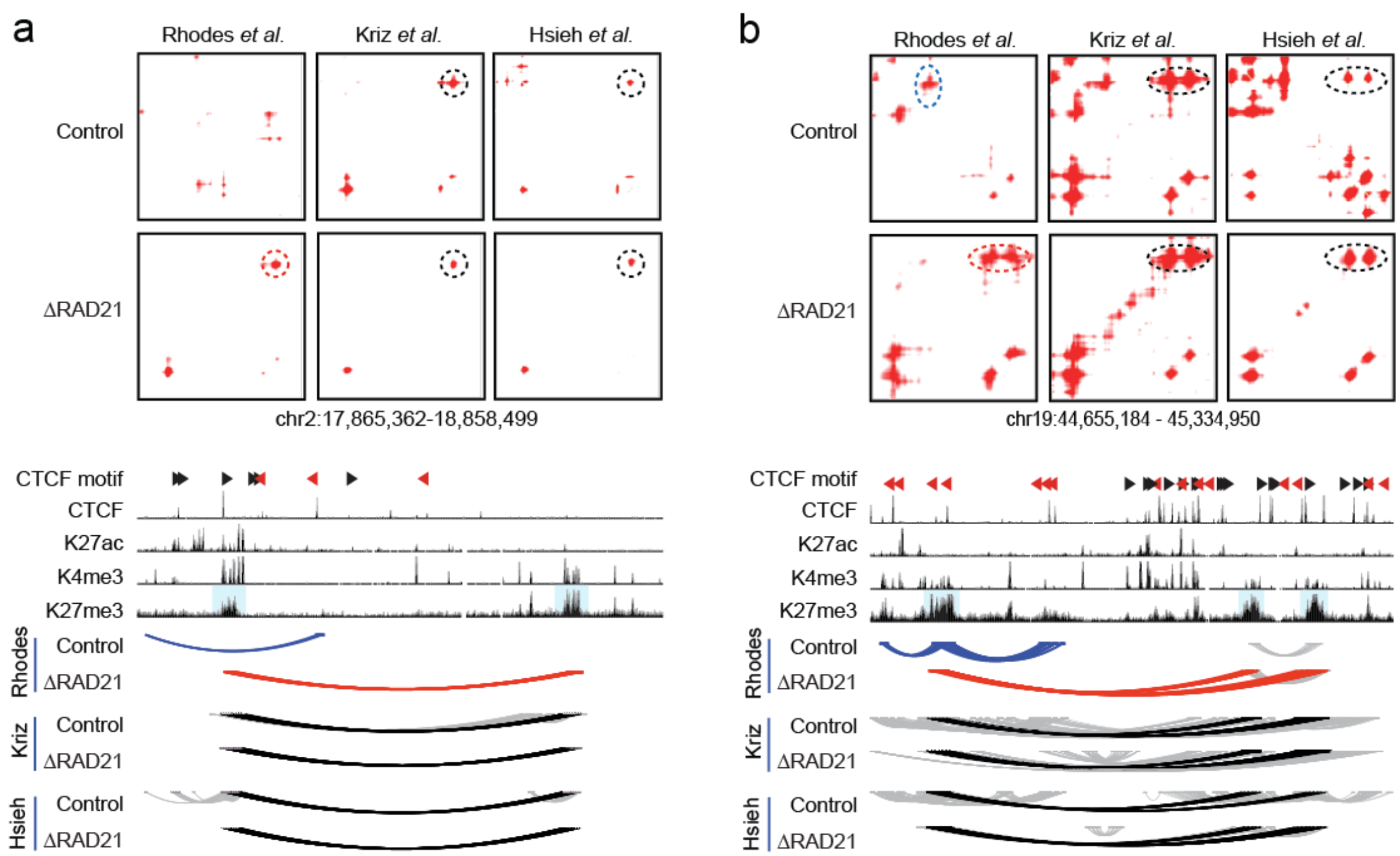

Supp. Fig. 8. "Gained" long-range H3K27me3 loops in Rad21-depleted cells from Rhodes et al. exist before Rad21-depletion in two other studies.

(Major) The use of the terms « dependent » and « independent » (e.g.,whole paragraph starting line 165) with respect to CTCF or cohesin should be avoided given the possibility that residual amount of these factors could

be present in the depleted cells, and the observation that, for example, some loops retained upon CTCF depletion display CTCF peaks at both anchors (44% of retained loops in Nora et al.) and are hence likely to be CTCF-dependent. The use of the terms « retained », « gained » or « lost » upon certain conditions should be favored for accuracy.

We appreciate the suggestion and have refrained the usage of "dependent" and "independent" in the revision.

(Major) Lines 172-175: The conclusion that « recurrently gained E-P interactions upon CTCF depletion are created by loop extrusion » because « "gained" loops in CTCF-depleted cells do not gain strength in Rad21-depleted cells » seems far-fetched, because these loops also do not show clear loss of strength upon RAD21 depletion (or this result is not properly presented and discussed, which would be expected if they are created by loop extrusion.

Both CTCF- and Rad21-depletion cause massive loss of TAD structure and nearly all CTCF loops. In this work, we argue that only CTCF-depletion also leads to recurrent gain of a small number of EP loops.

These "gained" loops in CTCF-degron data are expected to absent in all control cells, including both the control cells from CTCF-degron studies and the control cells from Rad21-degron studies. If these "gained" pixels in ΔCTCF cells are cohesin-dependent, they should remain weak in ΔRad21 cells, which is what we observed.

Here the reviewer argues that we cannot tell for sure those gained loops are created by cohesin unless we can see them getting lost upon Rad21 depletion. With current datasets we cannot test this because these loops do not exist in Rad21-degron control cells; we cannot test if these loops lose strength upon Rad21 depletion if they do not exist before Rad21 depletion. The only way to test this is to deplete CTCF first, wait till the cells gain the new loops, then deplete Rad21 and check if the gained loops disappear. This will require a cell line in which we can deplete both CTCF and Rad21 sequentially. Unfortunately, we do not have such a cell line which is beyond our current capability and the scope of this paper, but this is certainly an interesting experiment for the future.

On the other hand, we also observed that many of the "gained" loop pixels in ΔCTCF cells also gain strength in Δ*Wapl* cells (**Fig. 2d**, last row and **Supp. Fig. 9**, last row). This is further supported by the results in **Fig. 3d**, in which we found a significant fraction of loop gaining events also occurs in Wapl-depleted cells but almost none in Rad21-depleted cells. These observations in Wapl-degron data, together with the results from Rad21-degron data, suggest that cohesin plays a role in the formation of the "gained" loops in ΔCTCF cells: Rad21-depletion disables the loop formation and Wapl-depletion allows loop formation.

Regardless, we have revised the text to tune down the statement and reflect on the discussions above:

"…the "gained" loops in CTCF-degron studies, which are weak in both CTCF-degron and Rad21-degron control mESCs, do not gain strength upon Rad21-depletion cells, suggesting that the formation of these "gained" loops requires cohesin. In contrast, we observed that these "gained" loops in CTCF-depleted cells showed an apparent upward bias in the scatter plots of Wapl-degron studies. This observation…further supports a role of cohesin in forming those "gained" loops…" (line 232-238)

Line 177: « Supp Fig. 5a, b, third row » is mislabelled as « Supp Fig. 5b, d, third row ».

We thank the reviewer for pointing out this issue and have corrected these typos in line 236.

(Major) Fig. 3b: It is unclear how the FIN loci were identified, e.g. how many CTCF sites or loops are required to define such loci, and within regions of what size? This is especially important as FIN loci as identified at this stage then become the focus of the rest of the study (e.g., red highlight in Fig. 4h,j).

We apologize for the confusion. In this study we defined a few names: "loop-gaining locus", "FIN", and "FIN locus".

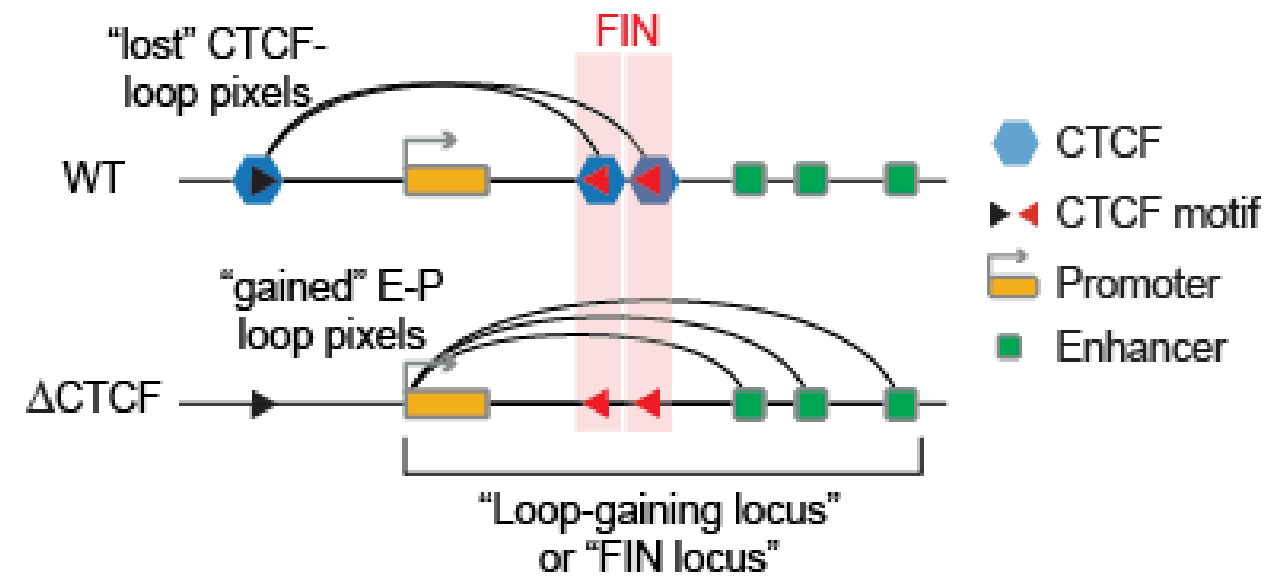

Review Fig. 1. Scheme to define Loop-gaining locus, FIN locus and FIN.

Loop-gaining locus is defined from "gained" loop pixels in CTCF-depleted cells. We identified thousands of "gained" loop pixels and each pixel represents a pair of 5kb-bins. However, these pixels often occupy overlapping regions. We define loop gaining loci for the convenience of unique counting. In **Review Fig. R1** on the right, we show three "gained" loops in ΔCTCF cells and the loop gaining locus is the entire region covered by all three arches. In our study, we have ~300 FIN loci after merging ~5,000 recurrent "gained" loop pixels.

FINs are CTCF binding sites that satisfy the following criteria (highlighted in pink in **Reviewer Fig. 1**):
(i) Located at the "lost" loop anchors in CTCF-depleted cells.
(ii) Located inside a newly "gained" loop in CTCF-depleted cells.
(iii) The other anchor of the "lost" CTCF loop is outside of the newly "gained" loop.
(iv) CTCF sites satisfying (i-iii) within 20kb of each other will be merged into one FIN. We do not have limits on the number of CTCF sites to define FINs. Therefore, although a FIN can be just one single CTCF site, most FINs have multiple CTCF sites.

FIN loci. As described above, all FINs must be inside loop-gaining loci. "FIN loci" are "loop-gaining loci" that containing FINs. In fact, almost all the "loop-gaining loci" are also "FIN loci".

We have modified the text to clarify the definition and added more detailed description in the **Methods** section.

(Major) Line 202: It is not clear how the overlap between TAD boundaries and FINs was analyzed. Was it perfect overlap only, or overlap allowing for certain shifts? This is important as TAD and boundary calling is highly dependent on what algorithm (e.g., based on switch in contact directionality, or based on local dips of contact density) and which parameters (e.g., bin size) were used.

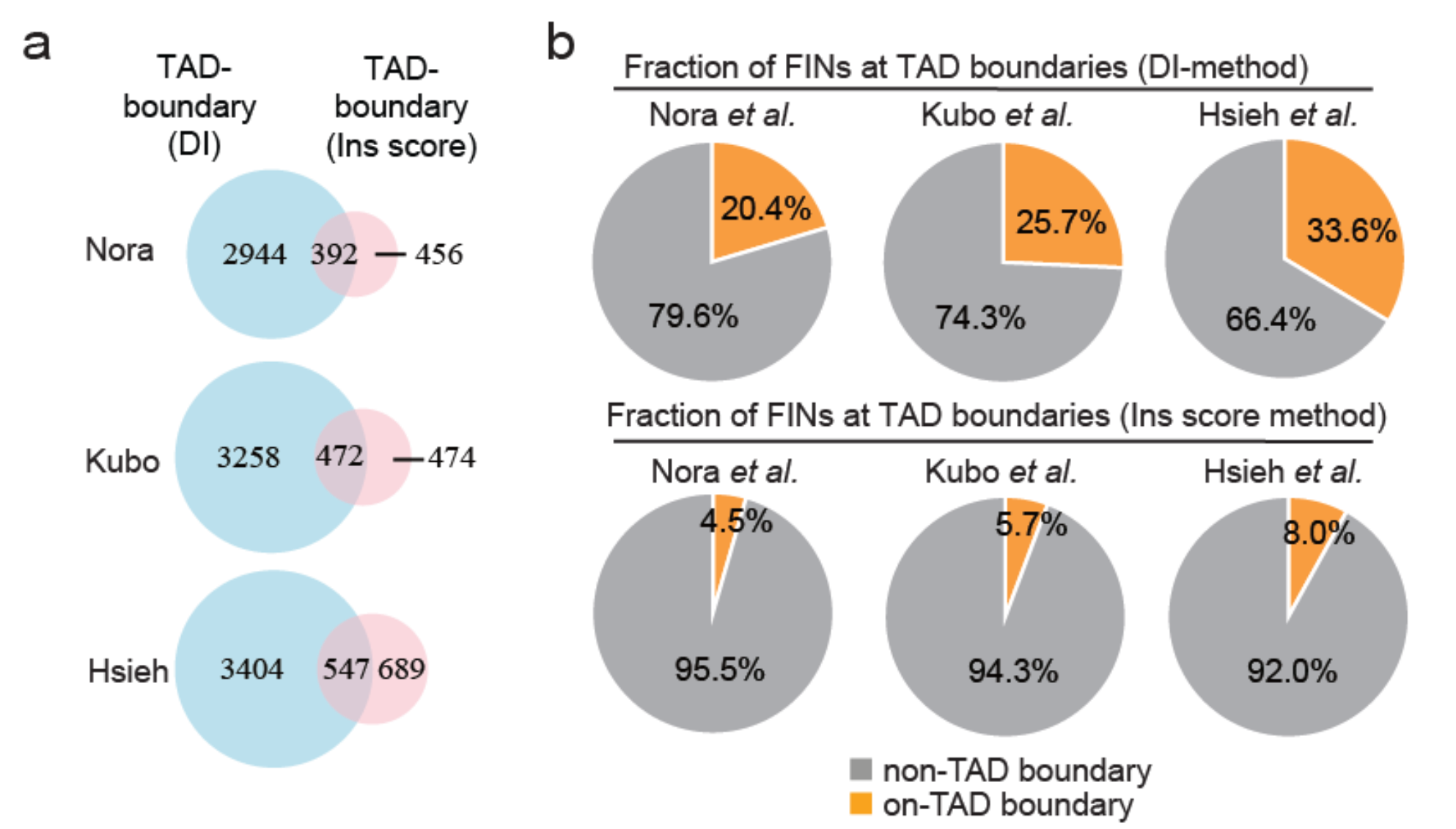

Supp. Fig. 11. (a) Compare the overlap between TAD boundaries called from DI and Insulation score. **(b)** Pie chart showing the percentage of FINs that overlap TAD boundaries called from different algorithms.

We defined overlapped FINs and TAD boundaries if they are within 40kb of each other. Therefore, most of the FINs are at least 40kb away from TAD boundaries. This information has been added to **Methods**.

Our previous version directly used the TADs in mESCs defined by *Dixon et al.* (PMID: 22495300) and concluded that only a small fraction of FINs overlaps TAD boundaries. In this revision, we now directly call TAD boundaries from the three datasets at 40kb resolution with 2Mb sliding window using two methods: (i) "Directionality index" (DI) -based method described by Dixon et al.; (ii) "insulation score"-based method (Crane et al., PMID: 26030525) using *Cooltools*. We added a new **Supp. Fig. 11** in this revision.

Indeed, as the reviewer commented, boundaries calling is highly dependent on the data and algorithm. DI-method calls many more TAD boundaries than InsScore method. The number of TAD boundaries also tends to

be higher when using more frequent Hi-C cutters (MicroC > in situ Hi-C > Hi-C) (**Supp. Fig. 11a**). As expected, the overlap between TAD boundaries and FINs increases if more TAD boundaries are called but regardless of the method of choice, only a fraction of FINs overlapped with TAD boundaries (**Supp. Fig. 11b**).

In this revision we also added a new **Fig.3f** with a FIN example (highlighted in pink) that is not at TAD boundary. In this figure we also included the tracks for "insulation score" and "directionality index". The two TAD boundaries in this example reach a local minimum of InsScore but the FIN does not. Similarly, using DI-based method, TAD boundaries are defined as locations where DI changes sign, but in this example, the FIN is located inside TAD, and its DI does not change sign.

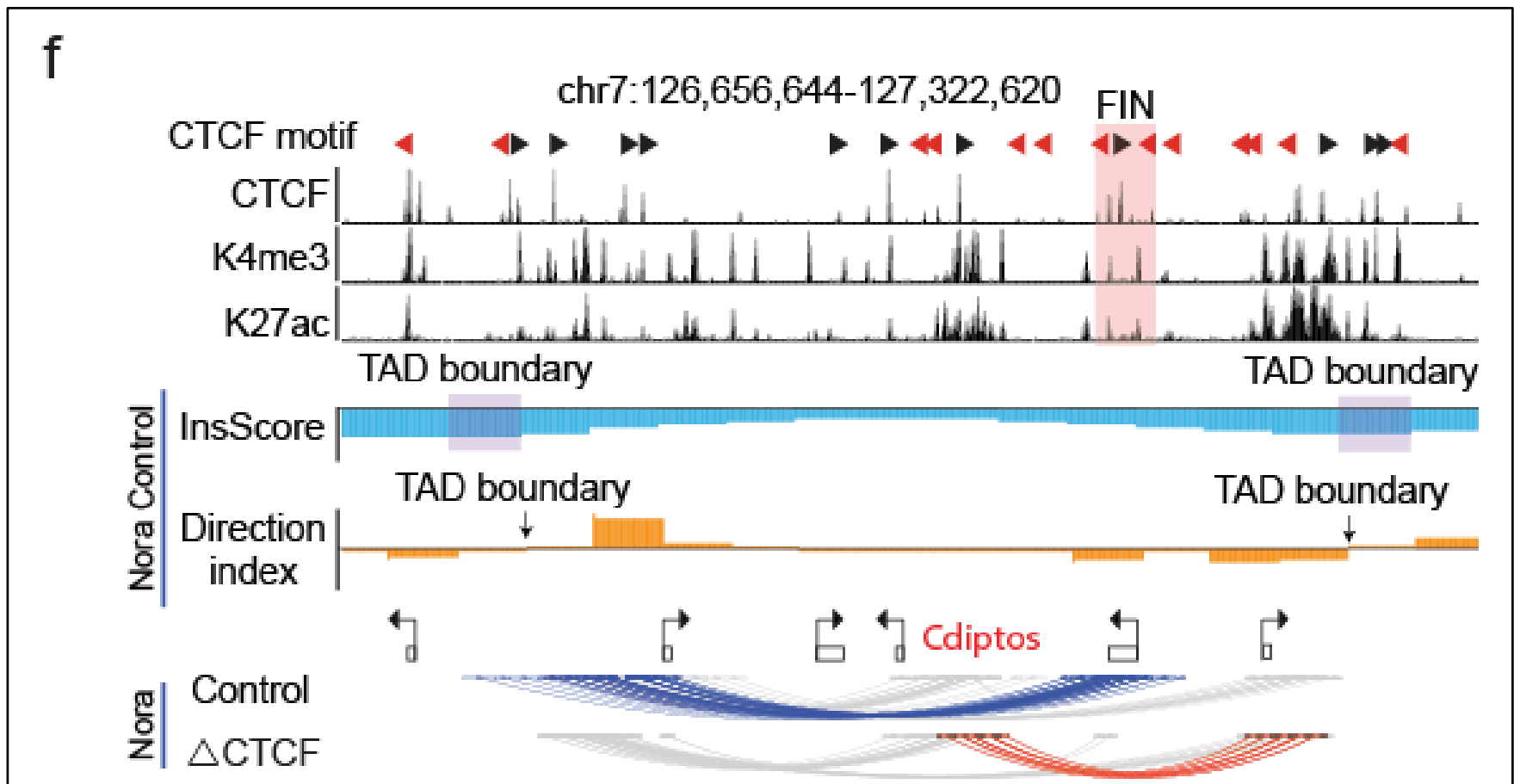

Fig. 3f. An example of non-TAD boundary FIN.

Fig. 4d: Acute CTCF depletion or degradation is not equivalent to a « KO » (genetic loss of function) condition,ot be abbreviated as such. Along the same line, control cells should also not be abbreviated « WT ».

We agree and thank the reviewer for pointing this out. We have revised all figures and text using "control" instead of "WT" and using "CTCF-depletion" or "ΔCTCF" instead of "KO" to indicating protein depletion.

Line 228: The statement that « Using NET-seq, Hsieh et al. identified more DEGs than RNA-seq despite shorter time of CTCF depletion (3hr) » is inconsistent with the data presented in Fig. 4e, showing that more DEGs are identified in Nora et al. (213 upreg, 229 downreg) compared to Hsieh et al. (174 upreg, 168 downreg).

We apologize for the confusion.

This statement was intended to compare the NET-seq and RNA-seq reported by Hsieh *et al.* upon 3-hour CTCF depletion. With 3-hour depletion, RNA-seq data from Hsieh *et al.* has 57 upregulated and 45 downregulated DEGs, while NET-seq data has 174 upregulated genes and 168 downregulated DEGs.

To make it clearer, we have revised the text to (line 319-324):

"…Hsieh et al. performed both NET-seq and RNA-seq after only 3hr CTCF-depletion, which should include fewer late-response genes, but the short treatment time may also reduce the sensitivity of DEG detection. We use NET-seq data because Hsieh et al. reported that NET-seq has higher sensitivity in identifying DEGs as it maps newly synthesized RNA, while RNA-seq measures the amount of steady state RNA in the cells…"

(Major) Line 241: The 49 DEGs at FIN loci are only potential or putative direct targets of insulators. Language should be adjusted accordingly.

We agree and have adjusted the language here as "putative direct targets" (line 335).

(Major) Line 256: While the gain in interactions with the distal H3K27me3-marked region might explain Pcdh-gamma gene downregulation, further investigation of the locus, e.g. by deleting the distal H3K27me3-marked region, is required to validate those interactions with the distal H3K27me3-marked region does indeed cause Pcdh-gamma gene downregulation. In the present situation, only an association or coincidence may be claimed, but no cause-and-effect relationship.

We thank the reviewer for the comments. In our manuscript we reported:

*"…A cluster of putative enhancers marked by H3K27ac is present at the 3' of γc promoters (**Fig. 4j**, highlighted in green), forming loop interactions with the promoters of γa's and γb's (**Fig. 4j**, blue arches), which are restricted by a FIN with multiple CTCF peaks (**Fig. 4j**, highlighted in pink). Upon CTCF depletion, the H3K27ac-interactions are weakened, and the Pcdh-γ promoter loops extend into a distal H3K27ac-poor region marked by H3K27me3, which explains the downregulation of γa and γb genes..."*

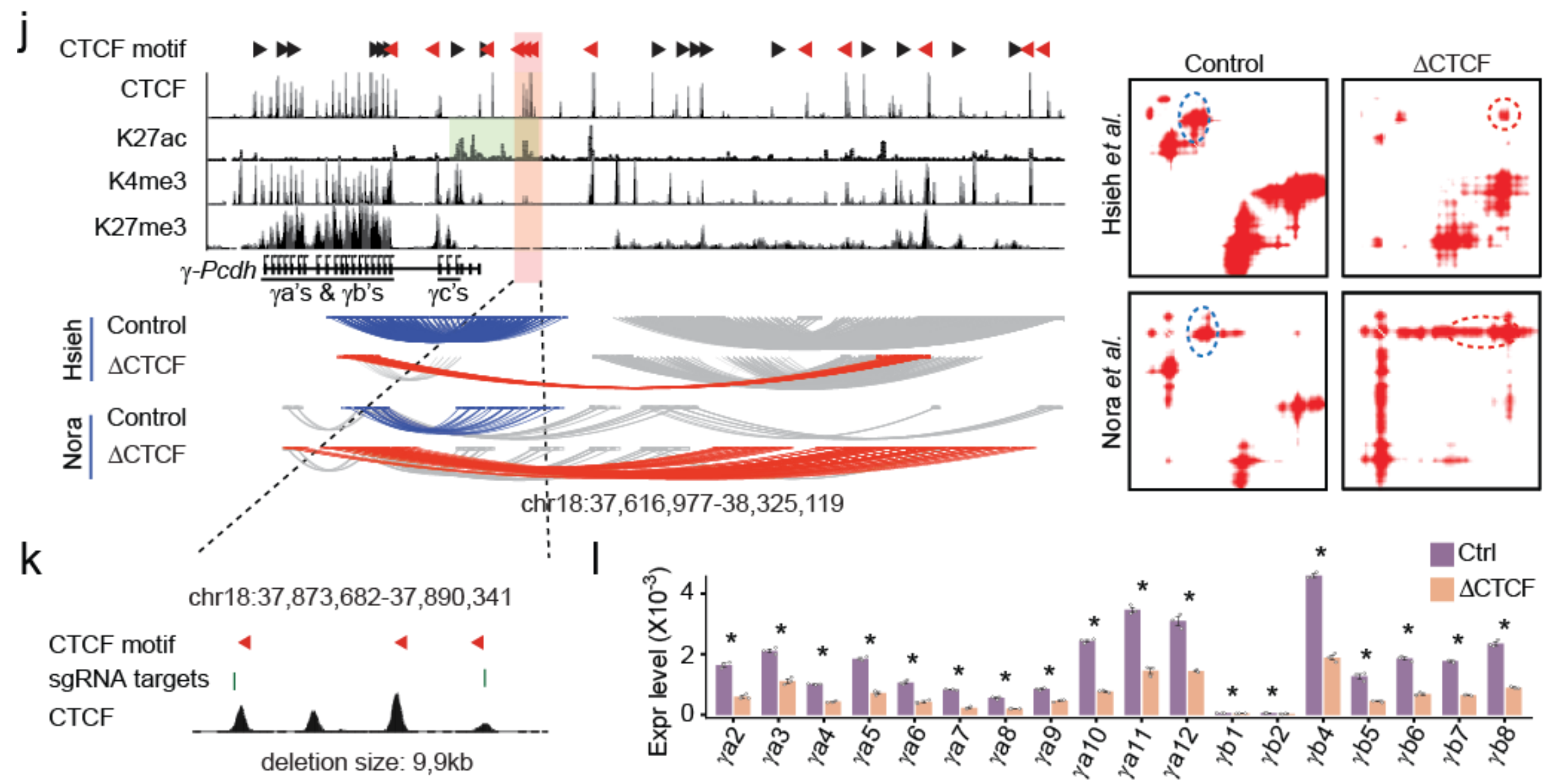

Fig. 4j,k,l. (j) Left: browser tracks near *Pcdh-γ* locus; right: Hi-C heatmaps from two CTCF-degron studies. **(k)** Design of sgRNAs for insulator deletion. (**l**) RT-qPCR shows the down-regulation of *Pcdh-γ* genes in mESCs after insulator deletion.

Here we reported two events upon CTCF depletion: loss of nearby enhancer loop and gain of distal H3K27me3 loop. Both are consistent with the downregulation of γa and γb genes. To avoid making causal inferences, in the revision we have rephrase from "…which explains the downregulation of γa and γb genes…" to "…consistent with the downregulation of γa and γb genes…" (line 351)

Additionally, we added new **Fig.4k,l** showing that deletion of the putative insulator indeed leads to down-regulation of γa and γb genes. However, we still cannot tell if the loss-of-enhancer-loop or gain-of-H3K27me3-loop (or both) causes the transcription changes, which require more extensive experiments.

Line 272 and Fig. 5c: While the text refers to CUT&RUN of CTCF, the axis label in Fig. 5c indicates CTCF ChIP-seq.

We thank the reviewer for picking up this typo and have updated the figure labels to CUT&RUN.

(Major) Line 279: The sentence should describe the gained loop interactions more explicitly: which regions do they connect? What are the characteristics of the connected regions? In addition, the end of the sentence stating that Mesp1 is upregulated « SINCE the new loops bring distal enhancers to its proximity » requires functional validation, typically by targeted deletion of the putative enhancer regions.

We have revised this paragraph as below providing more detail about the regions connected by the gained loops.

*"…In this example Mesp1 lost its loop interaction to a nearby FIN (**Fig. 5e**, blue arches) and upon CTCF depletion, gained promiscuous distal interactions to a large region that is ~200kb in size (**Fig. 5e**, red arches)..."* (line 375-377)

We also rephrase the text as below which describes the enhancer interactions without causation inference.

*"…Again, using 4C-seq we confirmed the gain of interactions to the large distal region containing several enhancer clusters (**Fig. 5e**, highlighted in green), and confirmed the upregulation of Mesp1 with RT-qPCR (**Fig. 5g**)…"* (line 379-382)

Line 281: The sentence should be toned down to « strongly suggest » (that FINs execute their insulating functions in cis) or similar, without further evidence with allele-specific CTCF perturbation and allele-specific gene expression measurements.

We agree "in cis" is not well supported and have revised this sentence as below:

"*…Taking together, these experiments verified the insulating functions of FINs identified from CTCF-degron Hi-C data…*" (line 381-382)

DISCUSSION

Line 293: Mentioning that « most TAD boundaries do not gain cross-boundary loops upon CTCF depletion » to contrast them with FINs is highly confusing, because neither do most FINs gain loops in the same condition. Instead, new loops may be gained between other regions that were previously blocked by FINs. This point needs to be clarified to avoid any confusion, especially between FINs and the gained putative E-P interactions.

We apologize for the confusion and thank the reviewer for pointing this out. To make it clear, we have rephrased in the revision:

"…Most TADs disappear upon CTCF depletion, and we typically do not observe gain of new loops across TAD boundaries. In contrast, most of the FINs are anchors of CTCF loops inside TADs in control cells, and we call an CTCF loop anchor "FIN" only if its flanking sequences gain new loops…" (line 393-396)

(Major) Line 304: The sentence is not sufficiently supported by data, as explained in the comment on lines 172-175 above.

This sentence was:

"…meta-analysis with Rad21- and Wapl-degron Hi-C data shows that the newly gained E-P loops in CTCF-depleted cells are cohesin-dependent and governed by cohesin regulators…"

In response to the reviewer's comments above. In this revision we have rephrase this sentence to:

"…meta-analysis with Rad21- and Wapl-degron Hi-C data shows that the newly gained E-P loops in CTCF-depleted cells do not form in cohesin-depleted cells and are also likely to be governed by cohesin regulators…" (line 410-412)

Line 306: The sentence describes a very weak and unconvincing evidence for cohesin dependency, through localization of FINs almost essentially in compartment A.

We have rephrased this sentence.

From:
"…the fact that almost all FINs are in compartment A (euchromatin) is also consistent with high cohesin activity because cohesin and its loading factors are known to co-localize with promoters and enhancers…"

To:
"…the fact that almost all FINs are in compartment A (euchromatin) suggests their dependency on surrounding active chromatin. Notably, promoters and enhancers may play key roles in loop initiation and stalling and are known to co-localize with cohesin and its loading factors…" (line 405-408)

(Major) Without functional validation, E-P loops in this study should be considered as putative, as the enhancers' activity and capacity to regulate the considered genes were not formally assessed.

We thank the reviewer for the comments.

Our validation experiments focus on disrupting insulators. **Figure 5** validated two FINs in which genes are upregulated when CTCF are displaced. The new **Fig. 4k-l** validated one more FIN at the downregulated Pcdh-gamma locus with CRISPR deletion assay. In all experiments we observed gain/loss of putative EP interactions with consistent transcription changes.

We agree with the reviewer that EP relationship remains putative without formally assessing enhancer functions with techniques such as dCas9-KRAB-based epigenome editing. We have adjusted the language throughout the manuscript using "putative enhancers" or "H3K27ac peaks" when mentioning candidate enhancer regions marked by H3K27ac.

METHODS

(Major) Line 639: What is meant by « For TADs and their boundaries, we directly use the TADs in mESCs defined by Dixon et al. »? Was the procedure re-used, or the same actual TAD and boundary calls were used? In the latter case, the use of TAD and boundary calls from a different study, Hi-C protocol and mESC line (J1 mouse ES cells in Dixon et al.) could readily explain the poor overlap between FINs and TAD boundaries. To ensure comparability, TADs/boundaries and FINs should be called from the exact same datasets.

Our previous submission used the same actual TAD calls by Dixon *et al.* from J1 mESCs.

In the revision, we now report TAD boundaries directly called from the control cell data in the CTCF-degron datasets. The new results are shown in **Fig. 3f-i** and **Supp. Fig. 11**. The methods have been updated as well.

Reviewer #2 (Remarks to the Author):

Map Functional Insulators with Cross-Platform Hi-C Meta-Analysis

General Thoughts:

Xu et al. characterize functional insulating regions of the genome (termed FINs) that contribute to gene regulation. They begin by analyzing multiple Hi-C and Micro-C datasets involving CTCF, Rad21, or Wapl depletions (Figure 1), using HiCorr and DeepLoop to characterize loop dynamics and identify gained loops upon CTCF depletion. They extend this analysis to other depletion datasets and compare loop dynamics across CTCF, Wapl, and Rad21 perturbations (Figure 2).

FINs are defined insulating regions where de-novo loops form between CREs upon CTCF depletion and are categorized into three types based on CTCF presence (Figure 3). They further link these regions to expression changes and describe new loop dynamics at FIN-containing regions (Figure 4). Finally, they use a dCas9 system to functionally validate selected FINs by targeting CTCF sites and observing transcriptional changes at loci such as Cdiptos and Mesp1 (Figure 5).

This study addresses an important question: why perturbations to chromatin conformation have profound effects in some genomic contexts but not others. While the authors attempt to define FINs in mESCs, the work would benefit from a deeper investigation into what makes an insulating region functional. Additionally, the figures often feel incomplete—more extensive display of the Hi-C/Micro-C matrices would add clarity to the results. Although the authors leverage multiple dataset, the cross-study comparisons are at times underutilized. Overall this work would benefit by further analysis into the FINs they define.

We thank the reviewer for recognizing our work's significance and the careful review of our work. We also appreciate the suggestions and below we have carefully answered the reviewer's comments and added new experiments and analyses into the revised manuscript.

Major comments:

Figure 1: The signal for Rhodes et al. seems very sparse. It would be helpful to show how these regions appear across other datasets, not just in CTCF depletion. Additionally, the general comparisons between datasets are limited to coverage metrics and a few loci. Performing PCA or a similar analysis on the different Hi-C/Micro-C experiments would better contextualize discrepancies between results and substantiate claims about differential depletion across studies.

Fig.1d already included the heatmaps of one CTCF-degron, one Rad21-degron, and one Wapl-degron datasets at *Notch1* locus. Our old **Supp. Fig. 1** (now **Supp. Fig. 4**) also showed two other CTCF degron datasets. We have now added new **Supp. Fig. 4g-h** which are heatmaps from the other two Rad21-degron and the other two Wapl-degron studies.

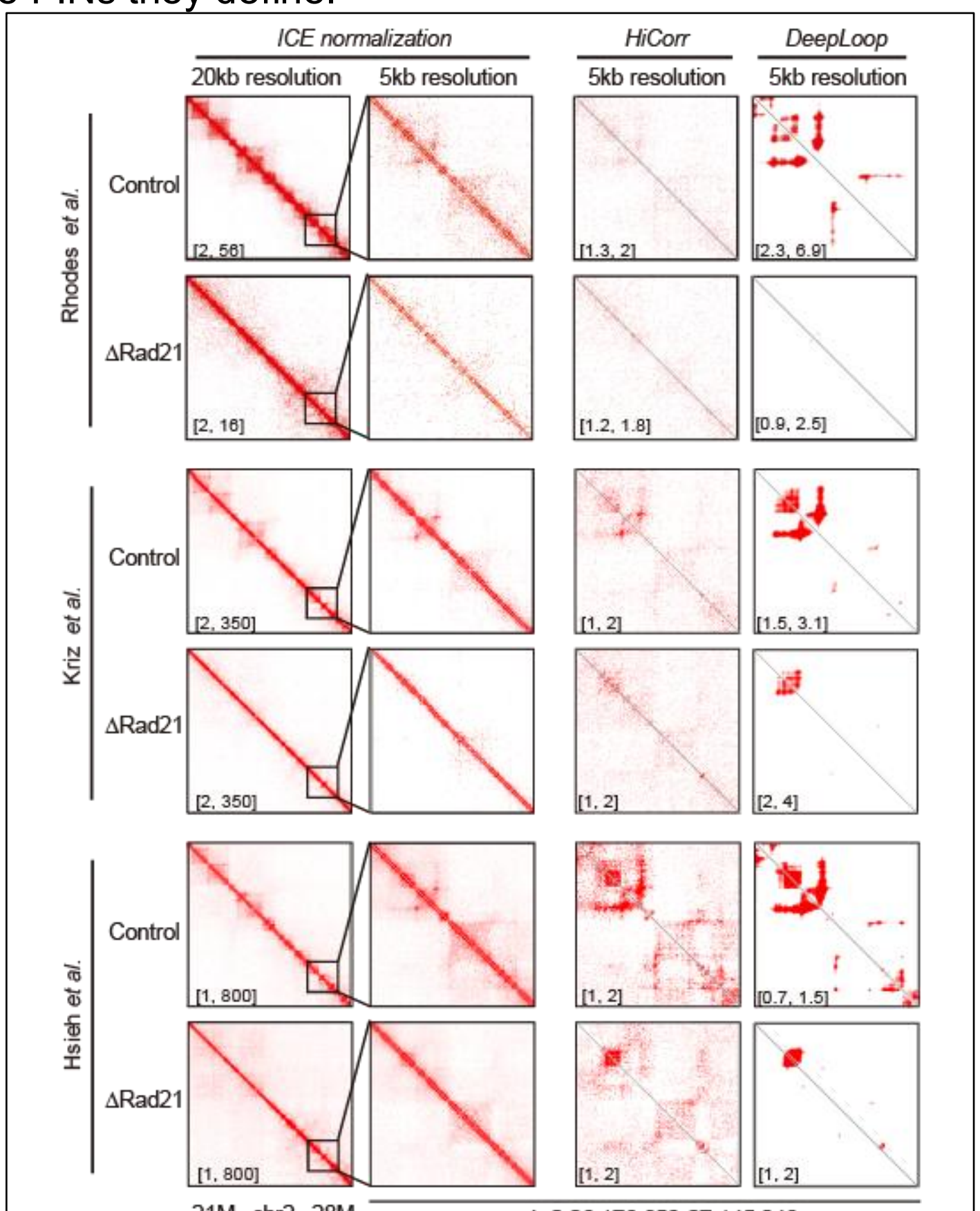

Reviewer Fig. 2. Contact heatmaps of the Rad21- Hi-C data at Notch1 locus generated from ICE, HiCorr and DeepLoop.

Reviewer Fig. 2 (panels from **Fig.1d** and **Supp. Fig. 4g**) on the right shows the Rad21-degron heatmaps at Notch1 locus. From this figure we can see that although

the signal for Rhodes *et al*. is indeed sparse, *DeepLoop* can robustly enhance the loop signal to the level comparable to Kriz and Hsieh (see the heatmaps of control cells in **Reviewer Fig. 2**).

In fact, because the control cells from all datasets are equivalent to wild type mESCs, their *DeepLoop* heatmaps are very similar. To quantify the consistency between orthogonal Hi-C experiments, we added a massive new **Supp. Fig. 2** in which we show more heatmaps and used scatter plots to compare the intensity of pixels (each pixel represents a pair of 5kb bins) between independent contact heatmaps. We also added a new **Fig.1b** (shown on the right) summarizing the overall correlation between control cells from different studies. Although conventional ICE-based pipelines show poor pixel-by-pixel correlation with R-square values often close to zero, HiCorr and DeepLoop clearly improved the consistency between orthogonal Hi-C/micro-C experiments with much better cross-platform R-square values (**Fig. 1b**).

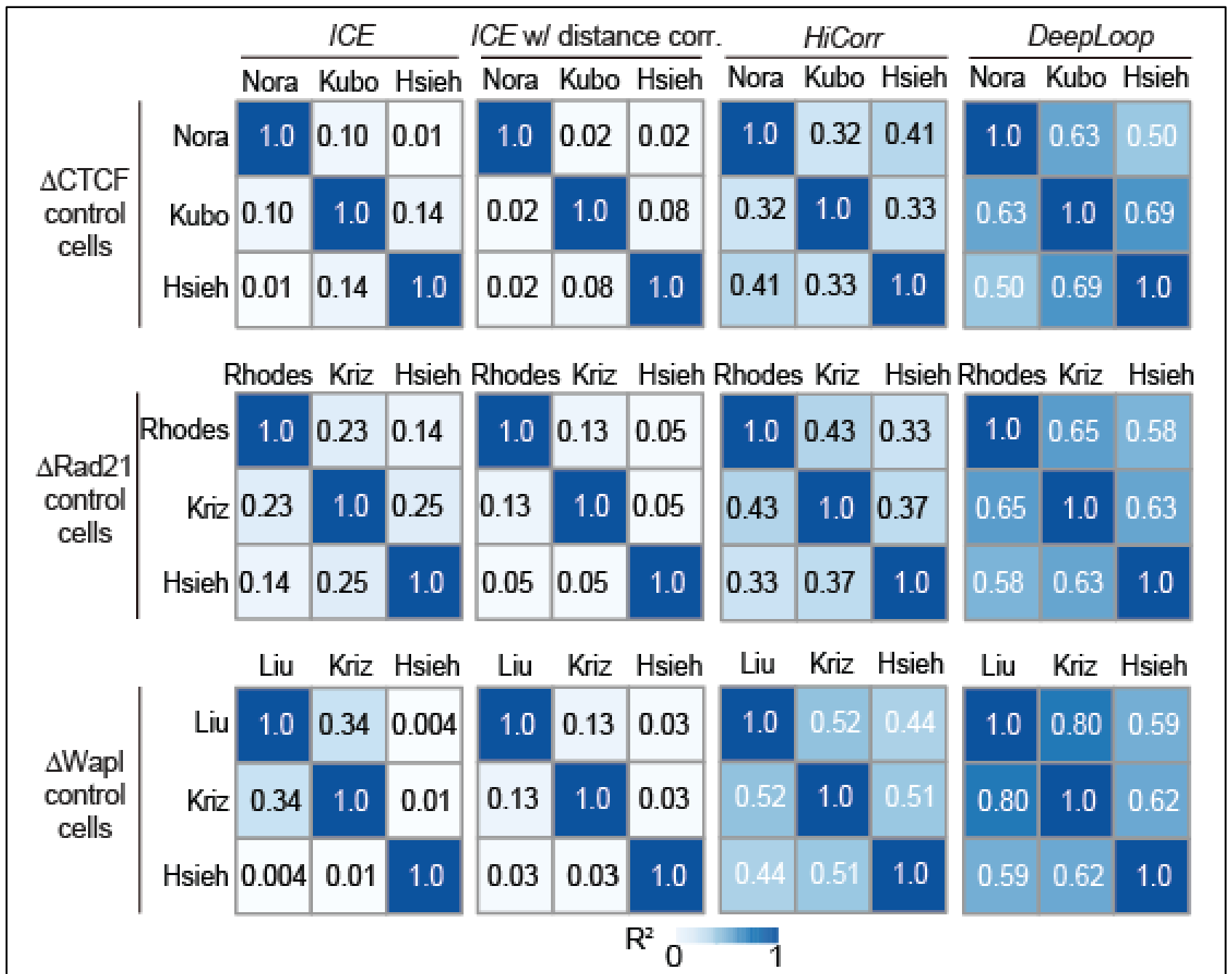

Fig. 1b. R-square matrices comparing Hi-C heatmaps in control mESC cells from different datasets when analyzed by different pipelines.

As the reviewer suggested, we also added a new **Supp. Fig. 3** below showing the cross-platform consistency when applying different pipelines to analyze dynamic loops. In this analysis we first generate "differential heatmaps" at 5kb resolution by subtracting each pair of contact heatmaps (depletion - control), then compute the correlations between the "differential heatmaps" from all nine pairs of degron experiments. Only *HiCorr* and *DeepLoop* pipelines can cluster CTCF- and Rad21-degron datasets together, and Wapl-degron datasets are separated (**Supp. Fig. 3**). This is consistent with the knowledge that depletion of either CTCF or Rad21, but not Wapl, causes massive loss of TADs and CTCF loops. Please note that we also included **Supp. Fig. 3a,d** into a new **Fig. 1c** in the main figure.

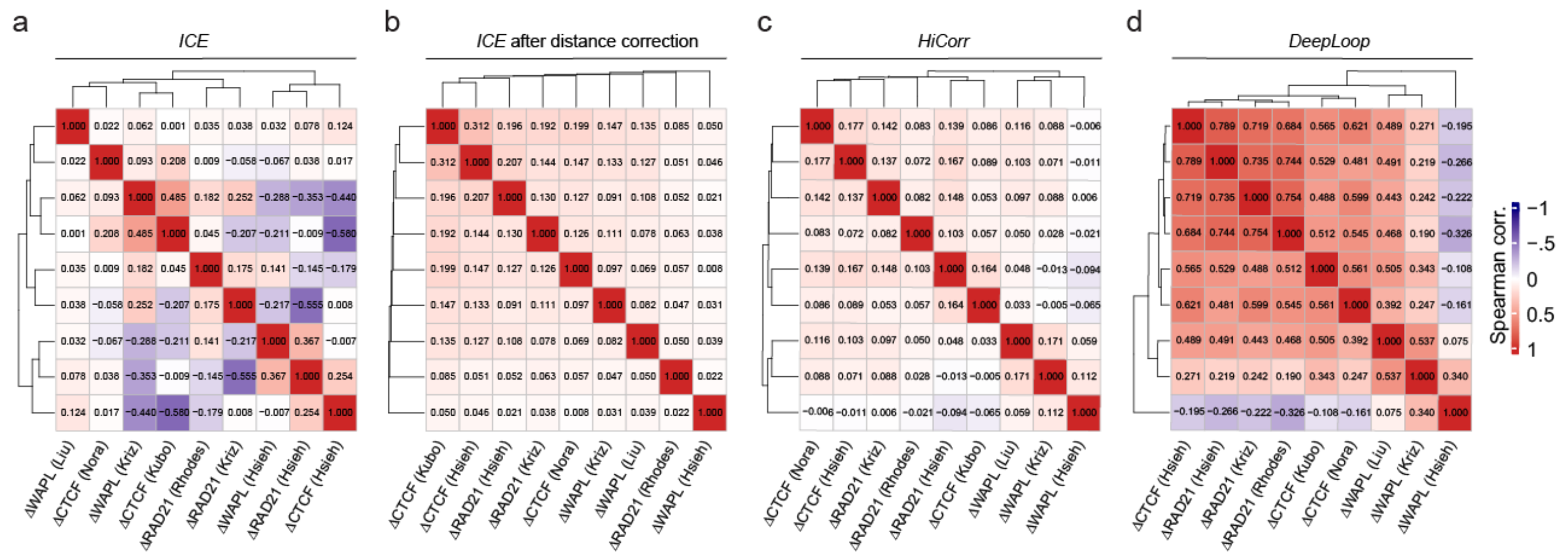

Supp. Fig. 3. Correlation matrices comparing the profiles of dynamic loops from all nine pairs of degron experiments.

It's a bit unclear what a pixel represents—I'm assuming one bin? What is the bin size, 5kb? These were then merged into regions down the line, but this is also not clearly explained as to how, or what exactly that means.

When talking about a loop pixel, we are referring to a pixel in the 5kb resolution contact heatmaps, which represents a pair of 5kb-bins.

We merge pixels to count genomic regions of interest. Specifically, we have ~5,000 “gained” loop pixels but many of these pixels occupy overlapping regions. We therefore merge all overlapping loops into one region and define them as “loop-gaining loci”. This step allows us to count the number of regions of interest in the genome. In our study, we have ~300 loop-gaining loci after merging ~5,000 recurrent “gained” loop pixels.

We have clarified the definition of loop pixels and described how to merge pixels in the revised main text and Methods.

I wonder if the longer gained loops being captured in Rhodes et al. (Supp Fig 3 – lines 150) are seemingly longer because the data is too scarce to capture short range loops that might need more coverage to be visible regardless of bias correction or DeepLoop.

In **Reviewer. Fig. 3** on the right, we compare the size of “lost”, “retained”, and “gained” loops from three Rad21-degron datasets (panels from **Supp. Fig. 7a,d,g**). We reported the “gained” loops from Rhodes *et al.* are longer, which is consistent with the conclusion by Rhodes *et al.* that cohesin removal enhances a series of long-range interactions between a subset of polycomb-occupied regions.

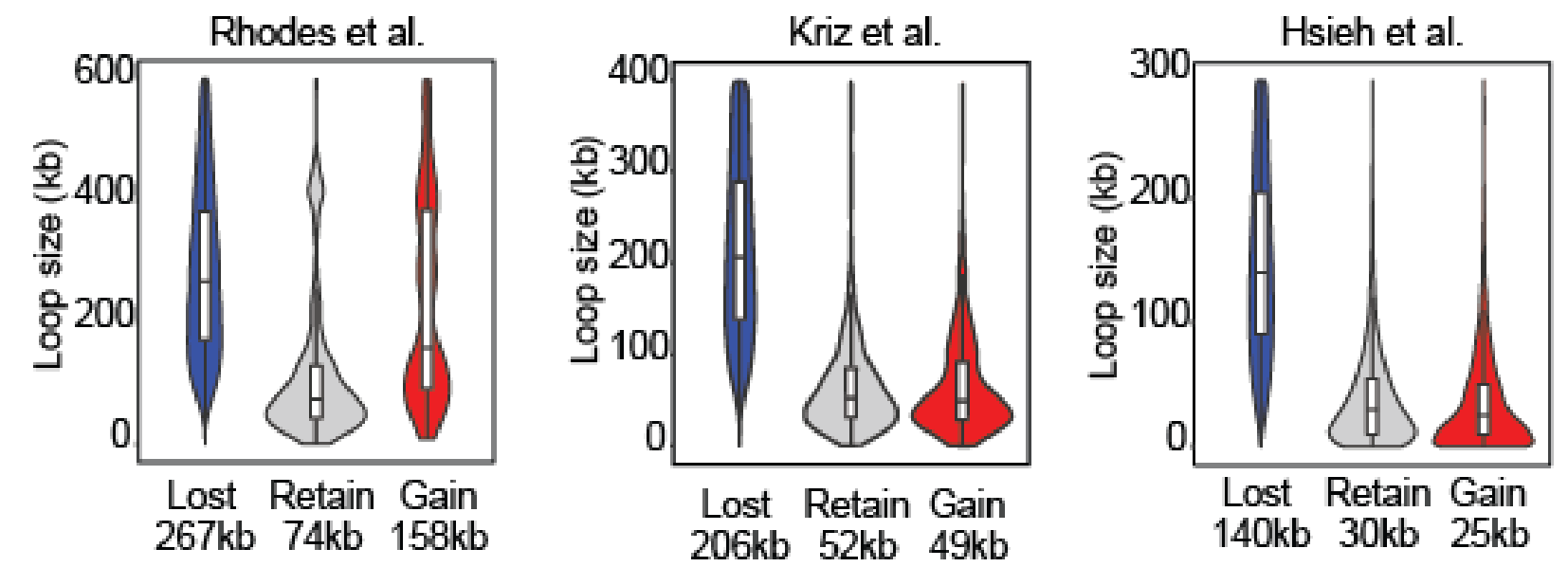

Reviewer Fig. 3. Loop size analysis in three Rad21-degron datasets.

Note that the “gained” loops in Rhodes *et al.* are longer than the “retained” loops in the same study. Data sparsity cannot explain this difference because the “retained” loops from Rhodes *et al.* are also called from low-depth Hi-C data (both control and ΔRad21 Hi-C data from Rhodes *et al.* are low-depth). In contrast, the “gained” loops in Kriz *et al.* and Hsieh *et al.* are smaller than “retained” loops from the same studies (**Reviewer. Fig. 3**).

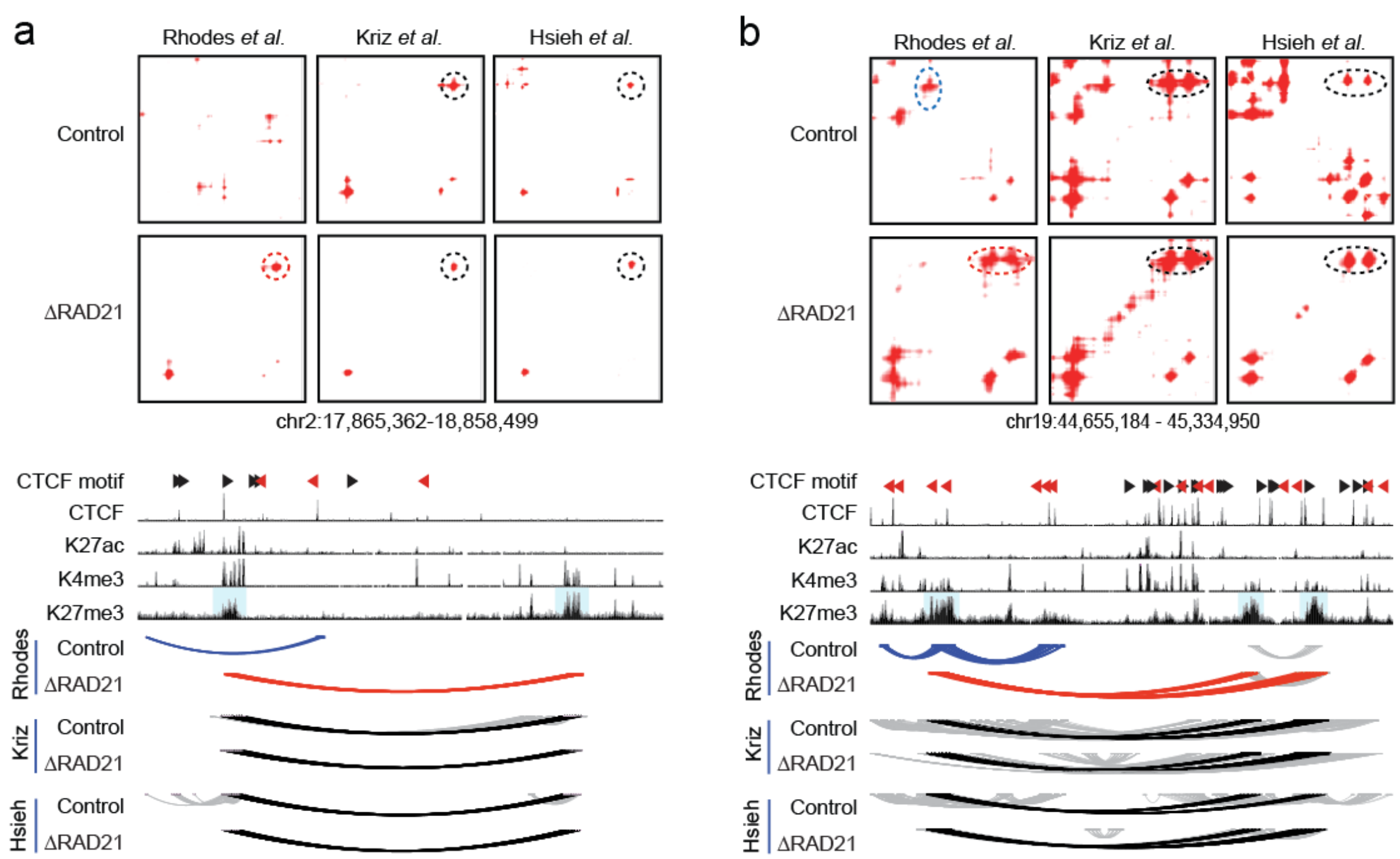

Supp. Fig. 8. “Gained” long-range H3K27me3 loops in Rad21-depleted cells from Rhodes et al. already exist before Rad21-depletion in two other studies.

We further examined the “gained” long H3K27me3 loops in Rad21-depleted cells from Rhodes *et al.* and found that in almost all cases, the “gained” long-range H3K27me3 loops are already present in the control cells from Kriz *et al*. and Hsieh *et al.* before Rad21-depletion (two examples are shown in a new **Supp. Fig. 8** above). Therefore, at least in the control mESCs from Kriz et al. and Hsieh et al., cohesin does not prevent the formation of these long-range H3K27me3 loops.

In lines 192-196, the term CTCF loop is brought up then switched to just insulation while this neither are really shown, more than just CTCF sites. It would be good to have an example region for each type of loop. TADs are also inconsistently discussed but not shown. Moreover, since TAD-calling can be noisy, more insulation score analysis at FIN vs non-FIN sites could strengthen the work.

We added a new **Supplementary Fig. 10** showing examples for each category of events discussed in **Fig. 3d**.

In this revision we also performed a much more comprehensive TAD level analysis shown in the new **Fig. 3f-i** and **Supp. Fig. 11.** We now call TAD boundaries in control cells using data from three CTCF-degron studies at 40kb resolution with two methods: (i) “Directionality index” (DI) -based method as described by Dixon et al.; (ii) “insulation score”-based method (Crane et al., PMID: 26030525) using *Cooltools*.

Indeed, as the reviewer commented, TAD boundaries calling can be quite variable depending on the dataset and algorithm. DI-method calls many more TAD boundaries than InsScore method. The number of TAD boundaries also tends to be higher when using more frequent Hi-C cutters (Micro-C > in situ Hi-C > Hi-C) (**Supp. Fig. 11a**). Unsurprisingly, the overlap between TAD boundaries and FINs increases if more TAD boundaries are called but regardless of the method of choice, it should be noted that TAD boundary always out-numbers FIN and only a fraction of FINs overlapped with TAD boundaries (**Supp. Fig. 11b**).

In this revision we also added a new **Fig.3f** with a FIN example (highlighted in pink) that is not at TAD boundary. Here the FIN is at the right anchor of a CTCF loop in control cells (blue arches). Upon CTCF depletion, the blue arches disappear and new loops (red arches) form connecting two clusters of

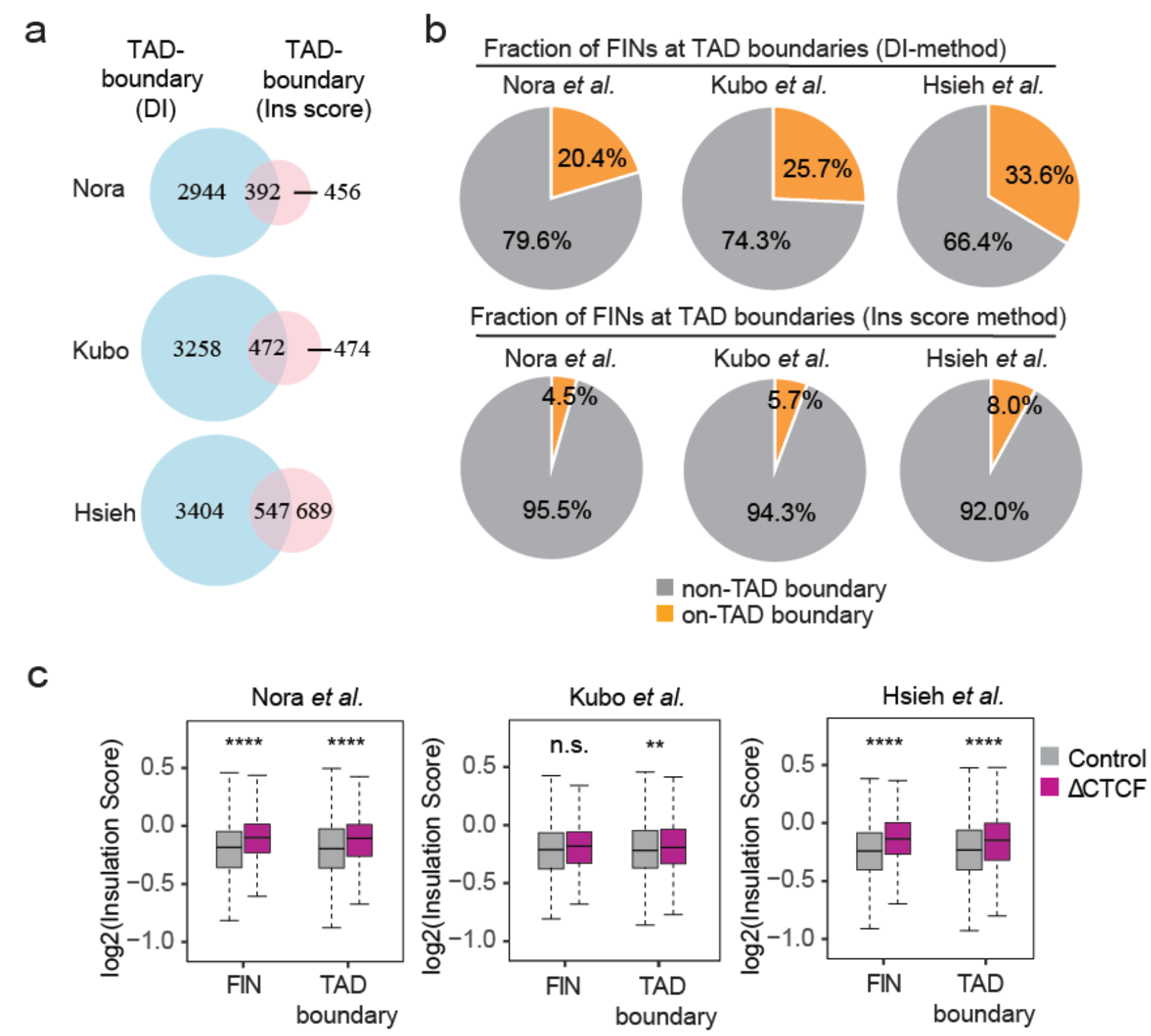

Supp. Fig. 11. (a) Compare the overlap between TAD boundaries called from DI and Insulation score. **(b)** Pie chart showing the percentage of FINs that overlap TAD boundaries called from different algorithms. **(c)** Compare insulation scores at FINs and TAD boundaries in different studies.

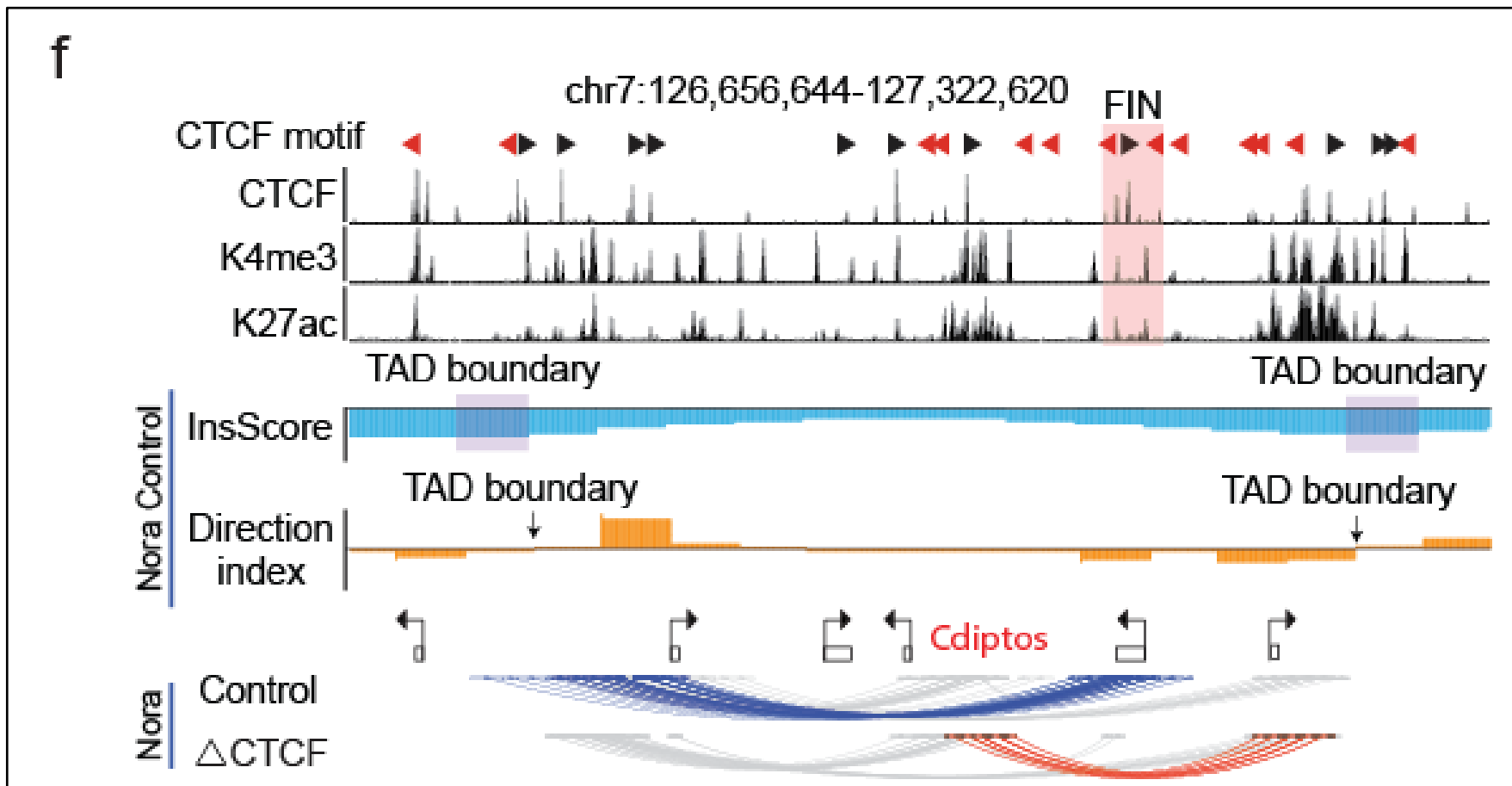

Fig. 3f. An example of non-TAD boundary FIN.

H3K27ac peaks flanking the FIN. In this figure we also included the tracks of “insulation score” and “directionality index”. Importantly, the two TAD boundaries in this example reach local minimum in the InsScore track but the FIN is not a local minimum (**Fig. 3f**). Similarly, using DI-based method, TAD boundaries are defined as locations where DI changes its sign, but in this example, the FIN is located inside a TAD and its DI does not change sign.

Change of InsScore also does not predict FINs. Although we observed an overall trend of increased InsScore at FINs upon CTCF depletion (except that the increase of “insulation score” in Kubo et al. is less significant) TAD boundaries also show the same trend and cannot be distinguished from FINs (**Supp. Fig. 11c**). Taking together, low-resolution TAD features lack the precision to reveal the dynamic changes of loop profiles as we used to define FINs.

This paper generally lacks in depth characterization of FIN’s. If there is ATAC-seq, for example, footprinting analysis would be interesting even in just wildtype mESC, to ask if there are any specific motifs or promoter types enriched at these regions which are being functionally insulated in mESCs? Moreover, Are these FINs dynamic across cell types, or stable but functionally relevant only in specific developmental contexts? Some of this is touched on in the discussion but could at least be looked at by analyzing a none-mESC Hi-C or Micro-C dataset.

We thank the reviewer for the great suggestions.

Following the reviewer’s suggestion, we added a new **Fig. 3l** on the right showing motifs that are enriched or depleted at loop-gaining ATAC-peaks in CTCF-depleted cells. Interestingly, the loop-gaining ATAC-peaks are enriched with many G-rich motifs but depleted with A/T-rich motifs (**Fig. 3l**). This result is reminiscent of previous reports that G-quadruplexes (G4) structure may regulate chromatin looping (Li et al., PMID:33199912; Wulfridge et al., PMID:37552993). Notably, many of the putative transcription factors binding to these G-rich motifs have been shown to regulate loop formation in various cell types, such as KLFs (Schoenfelder et al., PMID:20010836; Wei et al., PMID:23747203; Di Giammartino et al., PMID:31548608), SP1 (Bryan et al., PMID:40490791), MAZ and several zinc-finger proteins (Ortabozkoyun et al., PMID: 35145304, 39173638; Xiao et al., PMID: 33558242). Therefore, our results suggest that G-rich *cis*-regulatory elements are more likely to act as new loop anchors upon CTCF-depletion. We have added these discussions into the revised main text.

Regarding cell type specific FINs, we speculated in the discussion that FINs are likely to be cell type- or cell state-specific. However, mapping cell type-specific FINs requires CTCF-degron Hi-C data in a different mouse cell type, but to our knowledge, there’s no public CTCF-depletion Hi-C data in non-ES mouse cells yet. It will be certainly interesting to further explore the context specific roles of FINs in health and diseases in the future.

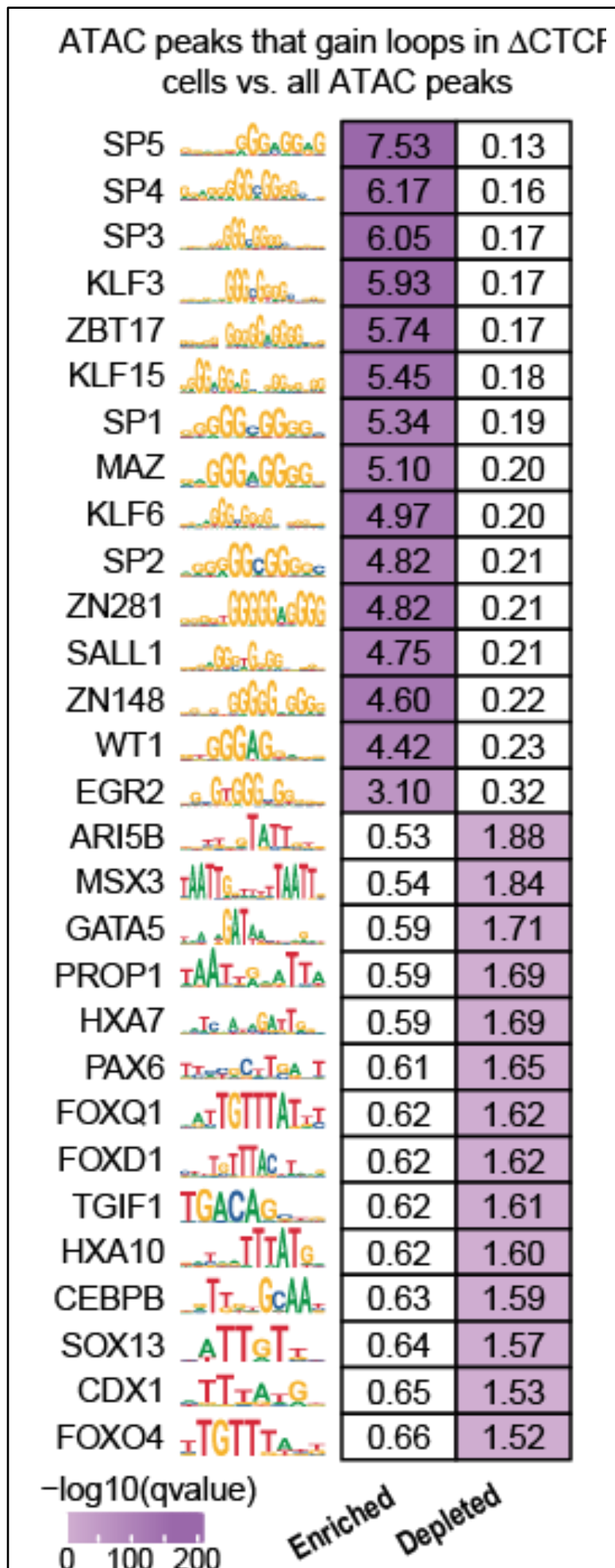

Fig. 3l. Motifs analysis at gained E-P loops in CTCF-depleted cells.

For Figure 3g: How does a FIN at a non-TAD boundary looks like, is it just because a TAD-call is not there or is it because this region shows no insulation (which is then a bit perplexing). While the schematics are helpful, real data should be shown. I would think that would be much more important for a main figure as a sanity check to the classifications than panel d for example.

We have already discussed on the last page of this document that we included a new **Fig. 3f** showing a non-boundary FIN example. This FIN is near the *Cdiptos* locus which is also shown in **Fig. 4g** and used for validation in **Fig. 5b-d**. **Fig. 3f** has included tracks of “insulation score” or “directionality index” with which we show that the FIN cannot be called by these measurements. Regardless of using “insulation score” or

“directionality index” based methods, TAD boundaries outnumber FINs, and only a minority of FINs overlapped with TAD boundaries. We note that FINs are defined based on dynamic change of high-resolution loop profiles and cannot be simply represented as TADs or TAD boundaries because after all, TADs or TAD boundaries are low-resolution 3D genome features.

Many of the figures need much better background and Supplemental to back up the claims being made. Few examples:

1. A bit more about the enhance models, since these were specific to each experiment.

We have added more descriptions about how we choose LoopEnhance models for different Hi-C datasets in Method section.

2. Classification of chromatin loops based on the epigenetic data, it would be helpful to see more examples of loop pixels within each group, and to see what “other” is, is it noisy regions of the genome for example or just regions with no CHIP coverage?

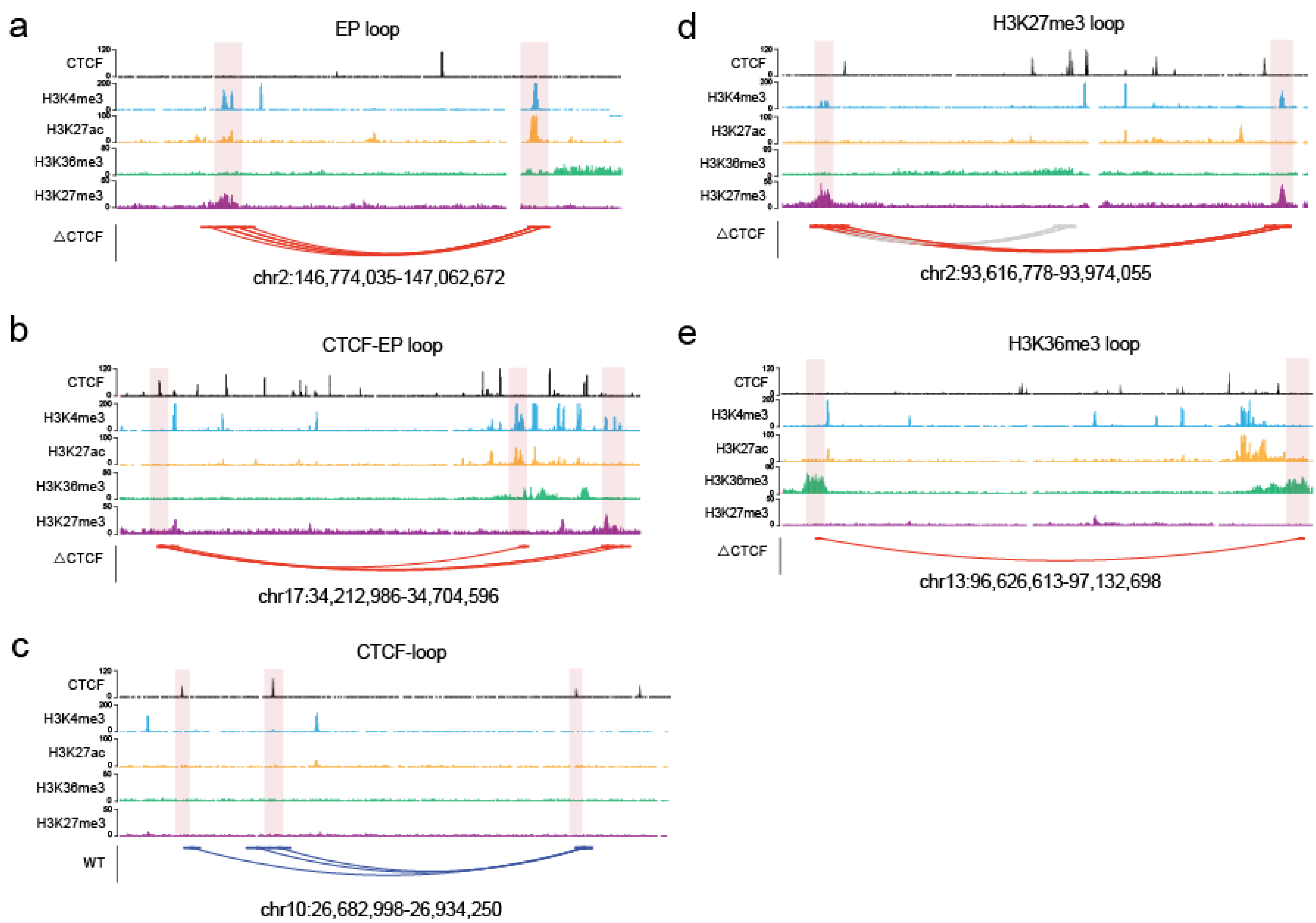

Supp. Fig. 6. Examples of chromatin loop classification.

We have included a new **Supplement Fig. 6** showing different types of loops. “Other” loops are pixels that neither end has ChIP-seq signal. We found many “other” pixels are “bystanders” that are next to a strong loop pixel (*e.g.*, a CTCF loop) but the two anchors do not have any ChIP-seq peaks.

3. A Supplement for Figure 4 could have easily incorporated the Hi-C maps between controls and degradation experiments without DeepLoop models, are they still visible?

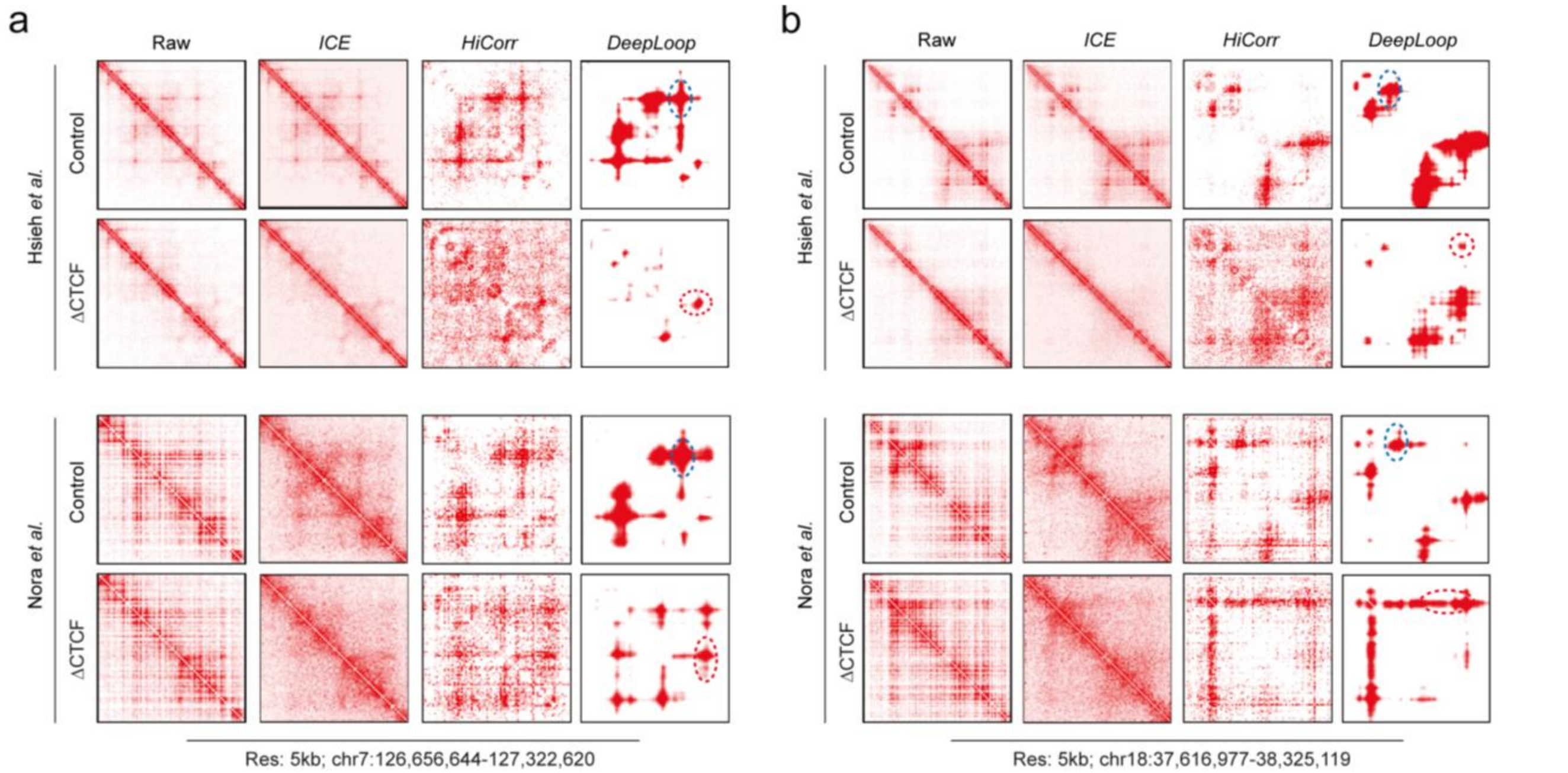

Supp. Fig. 12. Example contact heatmaps at putative insulator target genes Cdiptos and Pcdh-γ.

As suggested, we added a new **Supp. Fig. 12** showing Raw, ICE, *HiCorr*, and *DeepLoop* heatmaps for examples in **Fig. 4**. In all these examples, loop profiles are much cleaner in *DeepLoop*.

4. How was the RNA-seq normalized and how does that affect DEGs. Are ERCC spike-ins better than using empirical genes in RUVseq for example?

We performed differential expression analysis using *DESeq2*, which applies a robust normalization method based on scaling factors estimated from the median ratio of gene counts across samples. This approach assumes that most genes are not differentially expressed and is one of the most widely used methods in bulk RNA-seq studies.

RNA-seq data from Nora *et al.* and Kubo *et al.* do not have ERCC spike-ins. In **Reviewer Fig. 4**, we compared differentially expressed genes (DEGs) called with *DESeq2* and *RUVseq* packages**.** Applying RUVseq without ERCC spike-ins is tricky because we need to empirically pick some genes as negative controls. Here we used four housekeeping genes (*Actb*, *Gapdh*, *Pgk1*, *Atp5f1*) to estimate unwanted

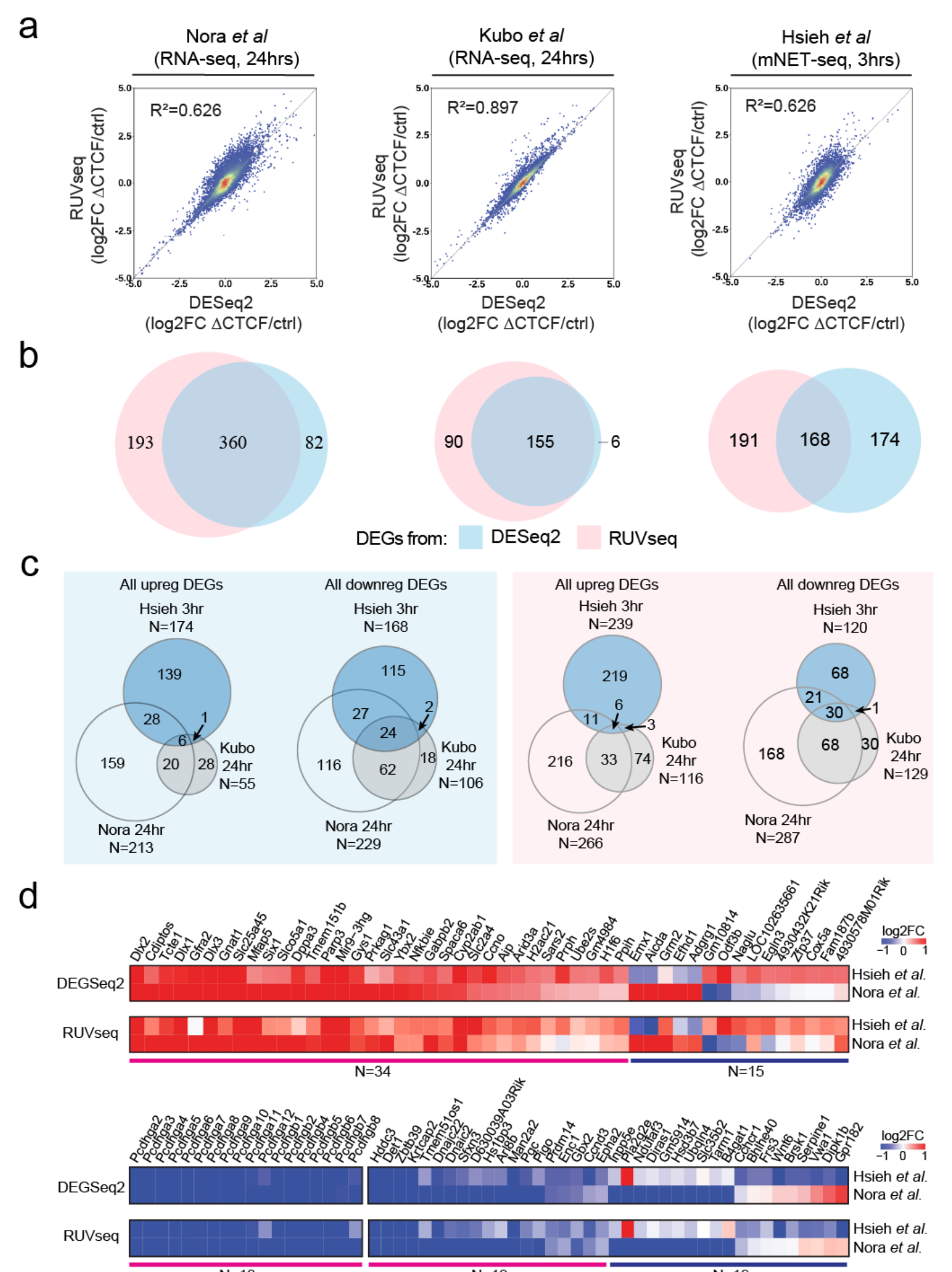

Reviewer Fig. 4. Compare DESeq2 and RUVseq in the differentially expressed gene analysis.

variations for RUVseq and compare the results to those from DESeq2.

We compared the fold-change values (ΔCTCF/control) of all genes from DESeq2 and RUVseq for all studies and the fold-change values are highly consistent with each other (**Reviewer Fig. 4a**). DEGs called from the two pipelines overlapped well. The overlap of Hsieh et al. data is lower because for NET-seq data we use looser cutoff for fold changes (we used 2-fold and $p<0.05$ for RNA-seq and 1.5-fold and $p<0.05$ for NET-seq to call DEGs) (**Reviewer Fig. 4b**). Regardless of DEseq2 or RUVseq, the reproducibility between the three independent studies is limited (**Reviewer Fig. 4c**). Finally, the putative FIN target genes (which are DEGs at "gained" loop anchors upon CTCF depletion) show consistent transcription changes regardless of using DESeq2 or RUVseq (**Reviewer Fig. 4d**). We therefore believe that using different RNA-seq analysis pipelines do not affect our conclusions.

Figure 4f isn't clear. Also, switching between Nora/Kudo and Hsieh datasets across subpanels (f–j) is confusing.

As we showed in **Fig. 4e**, the reproducibility of DEGs is limited between independent CTCF-degron studies due to experimental variations and indirect effects caused following CTCF-depletion. Our assumption is that experimental variations and indirect effects are less likely to be reproducible.

We hypothesize that DEGs near FINs are more likely to be the direct targets of CTCF-mediated enhancer-promoter insulation. If this hypothesis is true, DEGs near FINs should be more reproducible between independent CTCF-degron studies. Indeed, the upregulated DEGs near FINs (i.e., at "gained" loop anchors upon CTCF depletion) are more reproducible, but downregulated DEGs near FINs are less reproducible than controls (**Fig. 4f**, red bars). We have revised the main text with this explanation in line 326-332.

We also revise the text further explaining why we focus on the comparison of RNA-seq data from Nora (RNA-seq, 24hr) and Hsieh (NET-seq, 3hr) as following:

"…Both Nora et al. and Kubo et al. performed RNA-seq and the earliest timepoint was 24hr CTCF-depletion, which may still include late-response DEGs caused by indirect mechanisms (e.g., loss of pluripotency). Among the two RNA-seq studies, Kubo et al. detected fewer DEGs than Nora et al., but 70% of Kubo et al.'s DEGs are also observed in Nora et al. (47% of the upregulated DEGs and 81% of the downregulated DEGs, Fig. 4e). This observation is consistent with our discussion above that CTCF depletion in Kubo et al. was less complete. Hsieh et al. performed both NET-seq and RNA-seq after only 3hr CTCF-depletion, which should include fewer late-response genes, but the short treatment time may also reduce the sensitivity of DEG detection. We choose to use NET-seq data because the Hsieh et al. reported that NET-seq has higher sensitivity in identifying DEGs as it maps newly synthesized RNA, while RNA-seq measures the amount of steady state RNA in the cells…" (line 313-324)

The functional validation experiments are nice. However, matrix views of the targeted regions should be shown in addition to the *DeepLoop* model outputs. I think it would be also interesting to do this on a down regulated gene.

As the reviewer requested, we have added raw, ICE, HiCorr, and DeepLoop heatmaps for these two examples into **Fig. 5b,e**.

We also performed a new validation experiment showing that deleting a putative insulator indeed leads to down-regulation of γa and γb genes. The results are now included in the new **Fig. 4k-l** shown below.

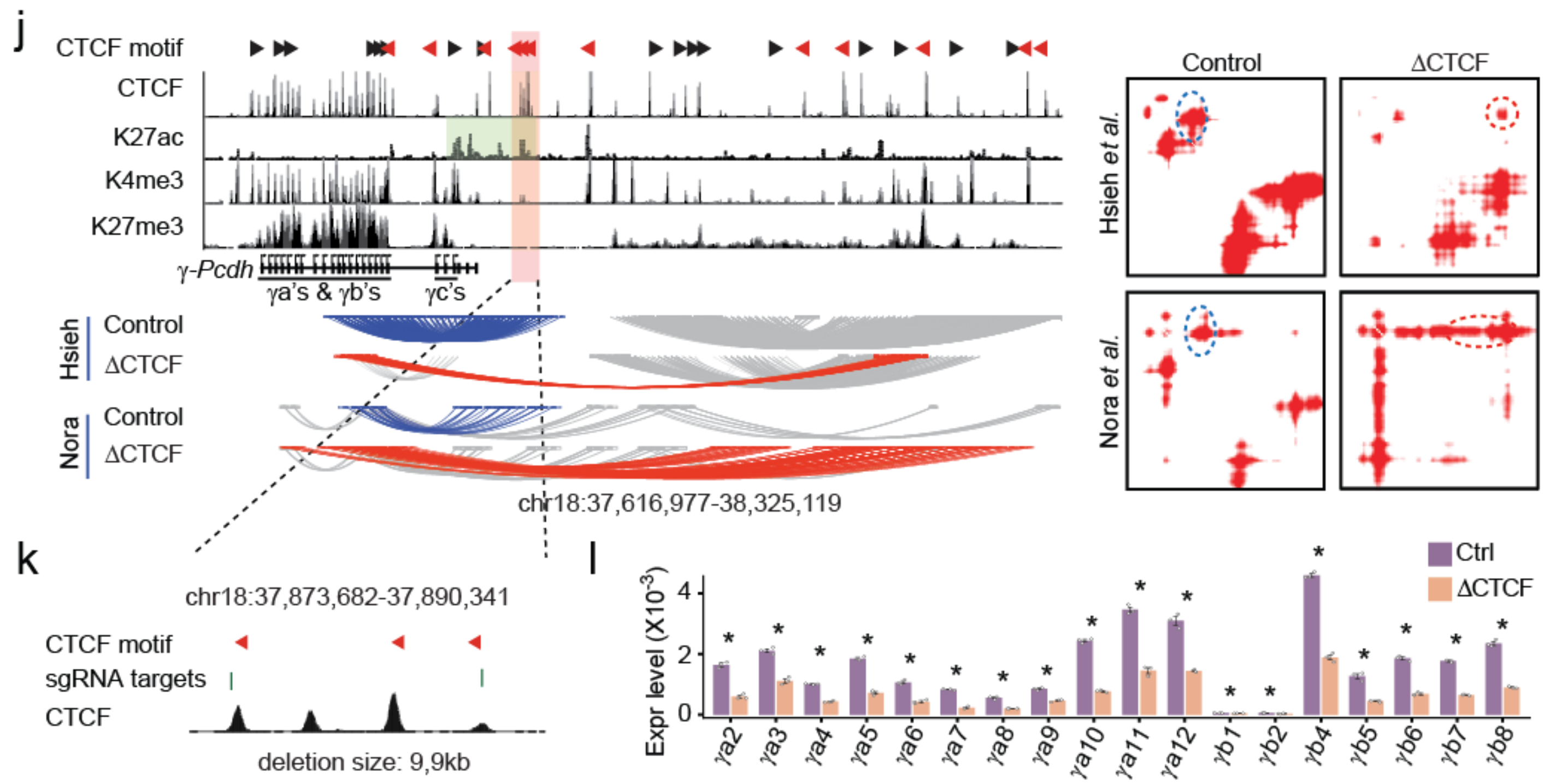

Fig. 4j,k,l. **(j)** Left: browser tracks near *Pcdh-γ* locus; right: Hi-C heatmaps from two CTCF-degron studies. **(k)** Design of sgRNAs for insulator deletion. (**l**) RT-qPCR shows the down-regulation of *Pcdh-γ* genes in mESCs after insulator deletion.

Minor comments:
The title needs improvement.

We have changed the title to:
"Cross-platform Hi-C meta-analysis identifies functional insulators that actively block enhancer-promoter interactions."

Supplemental Figures 3 and 4 should be made into a single supplementary figure.

We have merged these two supplementary figures into a new **Supplementary Fig. 7** as the reviewer suggested.

Line 180 ("more analysis below") can be deleted.

This sentence has been deleted.

Figure 4c could be moved to the supplemental section; the plot is confusing and adds limited value.

We have revised **Fig. 4c** to include only one pie chart showing that 16.8% of enhancers blocked by FIN are super enhancers.

For Figure 4e, adding NET-seq data next to Hsieh's data and RNA-seq results for other experiments would make comparisons easier.

We have revised **Fig. 4e** as advised with labeling of “NET-seq” for Hsieh’s transcriptome data and “RNA-seq” for Nora’s and Kubo’s transcriptome data.

In Line 232, it’s overstated that “most” DEGs overlap between Kudo et al. and Nora et al.—especially considering upregulated genes.

We have revised the sentence to make it clear that 47% of the upregulated DEGs and 81% of the downregulated DEGs from Kubo’s data are also among Nora’s DEGs (line 317-318).

Reviewer #1 (Remarks to the Author)

All my previous points have been satisfactorily addressed, and I recommend the manuscript for publication.

Reviewer #2 (Remarks to the Author):

Cross-platform Hi-C meta-analysis identifies functional insulators that actively block enhancer-promoter interactions Cui, Xu, Lang et al.

General Thoughts:

The authors have added substantial new analysis and some experiments to the work. They have also improved the overall discussion of their results, which initially contained too much over-interpretation with limited support, as also noted by Reviewer #1. While the manuscript has improved, figure presentation for easier interpretability can still be improved.

We sincerely thank the reviewer for the time and constructive feedback. We are pleased to see that the additional experiments, expanded analyses, and the refined discussion have addressed the previous concerns regarding interpretation. Following the reviewer's latest suggestions, we have further revised the manuscript and figure presentation to improve interpretability.

Minor comments:
1. Sentence 44-46:
"TADs disappear upon loss of NIPBL, while loss of WAPL causes bigger loops because cohesin can pass through CTCF blocks due to prolonged loop extrusion" could be rewritten to be a bit more readable.

We thank the reviewer for the suggestion. We have revised the text to improve flow and readability as follows (Line 46-49):

"…While deleting MAU2 or NIPBL results in the disappearance of TADs and a massive reduction of chromatin loops, the loss of WAPL produces the opposite effect, generating larger loops by enabling cohesin to bypass CTCF barriers through prolonged loop extrusion…"

2. Section 116-121
Current text:
"Only the matrices from HiCorr and DeepLoop clustered CTCF- and Rad21-degron datasets together and Wapl-degron datasets are separated out (Fig. 1c, Supplementary Fig. 3). This is consistent with the knowledge that depletion of CTCF or Rad21, but not Wapl, causes massive loss of TADs and CTCF loops. Taken together, these results support the robustness of DeepLoop in cross-platform analysis regardless of read depth."
I think it would be worth noting that both HiCorr and DeepLoop signals appear to saturate toward looping (as one would expect of DeepLoop for example). In contrast, ICE/ICE+distance correction may retain additional or broader structural features, which could explain the lack of clustering/correlation. Clarifying this would strengthen the interpretation.

We agree that the divergent performance of these methods partly reflects their different priorities: while ICE-based normalization preserves more architectural features, the HiCorr/DeepLoop pipeline is specifically optimized to prioritize discrete loop signals. We have added the following clarification to the manuscript (Line 131-139):

"…We attribute this improved robustness to the fact that the HiCorr/DeepLoop pipeline prioritizes loop signals through rigorous bias correction and normalization. This process effectively eliminates signals arising from both platform-specific technical biases (e.g., restriction site density and distribution) and non-loop chromatin interactions, including non-specific local chromatin compaction and other broader structural features. While the non-loop architectural signals retained by ICE-based normalization may carry biological meaning, they are

often highly sensitive to protocol variations in kilobase-resolution analyses. At this scale, such features can act as confounding noise, compromising the consistent and accurate quantification of loop strength across disparate datasets…"

3. Figure interpretability:
I think a simple heatmap (as in deeptools) of different tracks (H3K27ac, H3K27me3, RNA-seq, etc.) at loop anchors of FINs (perhaps even separated by class type) could be useful to visually summarize the features associated with dynamic loops.
I appreciate the new analysis in Supplemental Figure 1. I do think a simple heatmap showing insulation at gained, lost, and maintained anchors would be interesting. This could help clarify whether these are regions that are already prepped but blocked, or if it's a mix of multiple dynamics including de novo gain of regulation. I would suggest using anchors rather than merged regions, as otherwise there may be too few regions and mixed sizes - but not sure.

We thank the reviewer for this excellent suggestion. We have now added a new panel to **Figure 2b** (shown as **Reviewer Fig. 1** on the right) that displays the ChIP-seq signals for various histone marks centered on the anchors of "lost," "retained," and "gained" loops. Following the reviewer's recommendation, we performed this analysis using discrete loop anchors rather than merged regions to maintain spatial precision. We found that RNA-seq data was unfortunately too sparse and too diverse to provide a clear visual summary in this format.

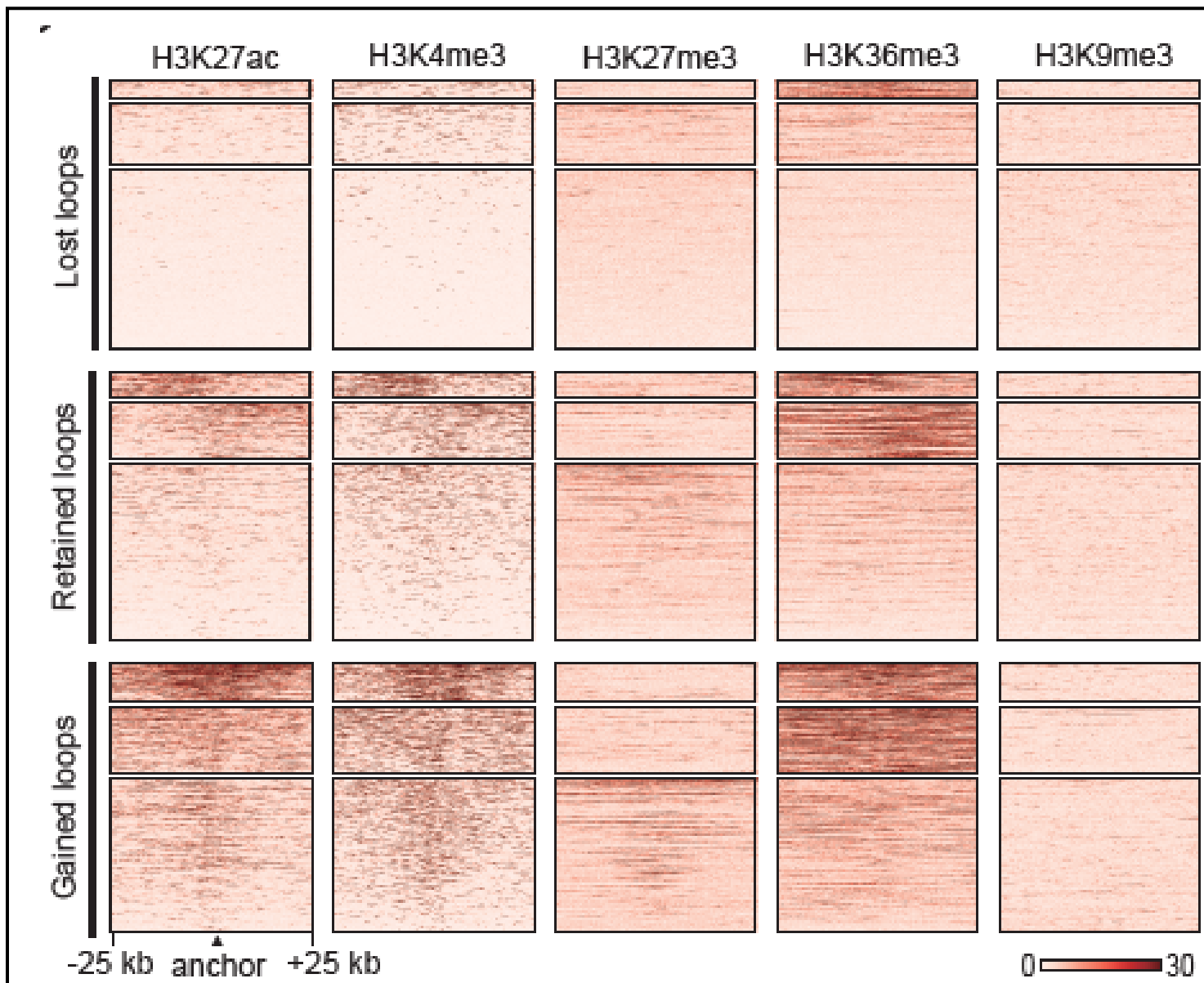

Reviewer Fig. 1. Heatmaps of ChIP-seq signal for histone marks at the anchors of lost, retained, and gained loop pixels in CTCF-depleted cells. The centers of loop anchors are aligned and the heatmaps plotted the regions ± 25kb of the anchor centers. Adopted from **Fig. 2b** in our manuscript.

As shown in the new Figure 2b, the anchors of "retained" and "gained" loops exhibit significantly higher enrichment of H3K27ac, H3K4me3, H3K27me3, and H3K36me3 compared to "lost" loop anchors. This enrichment indicates that, in the absence of CTCF, chromatin loops preferentially anchor at these epigenetically marked or "prepped" regions. This result is highly consistent with our loop classification in **Figure 2c**, which highlights a higher proportion of enhancer-associated loops within the retained and gained populations.

Regarding the question of "prepped but blocked" versus *de novo* regulation, the presence of these marks in the baseline (wild-type) state suggests that these regions are functionally "prepped" for interaction prior to CTCF loss. While we cannot directly measure histone mark changes following acute depletion (as these data were not generated in the original studies), the rapid timescale of CTCF degradation suggests that the observed loop rewiring primarily utilizes this pre-existing epigenetic landscape rather than relying on *de novo* chromatin remodeling.

4. Figure Organization:
Figure 1: I would move Nora et al. to the bottom to be closer to the tracks in panel e.

We thank the reviewer for this helpful suggestion.

To ensure the Nora et al. heatmaps are positioned close to the genome tracks in Figure 1e, we reorganized the layout as follows:

- Figure 1d has been revised to exclusively display the Nora et al. heatmaps, positioning them directly above the tracks in panel e for easier comparison.
- The heatmaps for Rhodes et al. (Rad21-AID) and Liu et al. (Wapl-AID) have been moved to a new panel, Figure 1f, located below panel e.

We believe this restructured layout significantly improves the interpretability of the multi-omic data integration in this figure.

Figure 2: Could incorporate the panels currently in Supplemental Figure 6. This would clarify and simplify interpretation for the reader. I would consider including EP loops, H3K27me3 loops, and CTCF loops, with matrices of treatment/depletion overlaid. I think the manuscript generally lacked showing the underlying data. While this has improved, there is still room to expand this further.

As the reviewer suggested, we have now incorporated all panels in Supplemental Figure 6 into Figure 2d, with added heatmaps. These examples are included to show how we classified loops based on their epigenetic marks.

In addition, please note that the figure panels in Fig. 1e, 3f, 4h, 4j, 5b, 5e, and Supplementary Fig. 9a, 9b, 9d are all examples of FINs loci.

5. The Pcdh locus is interesting - could this be due to mis-regulation of Polycomb domain?

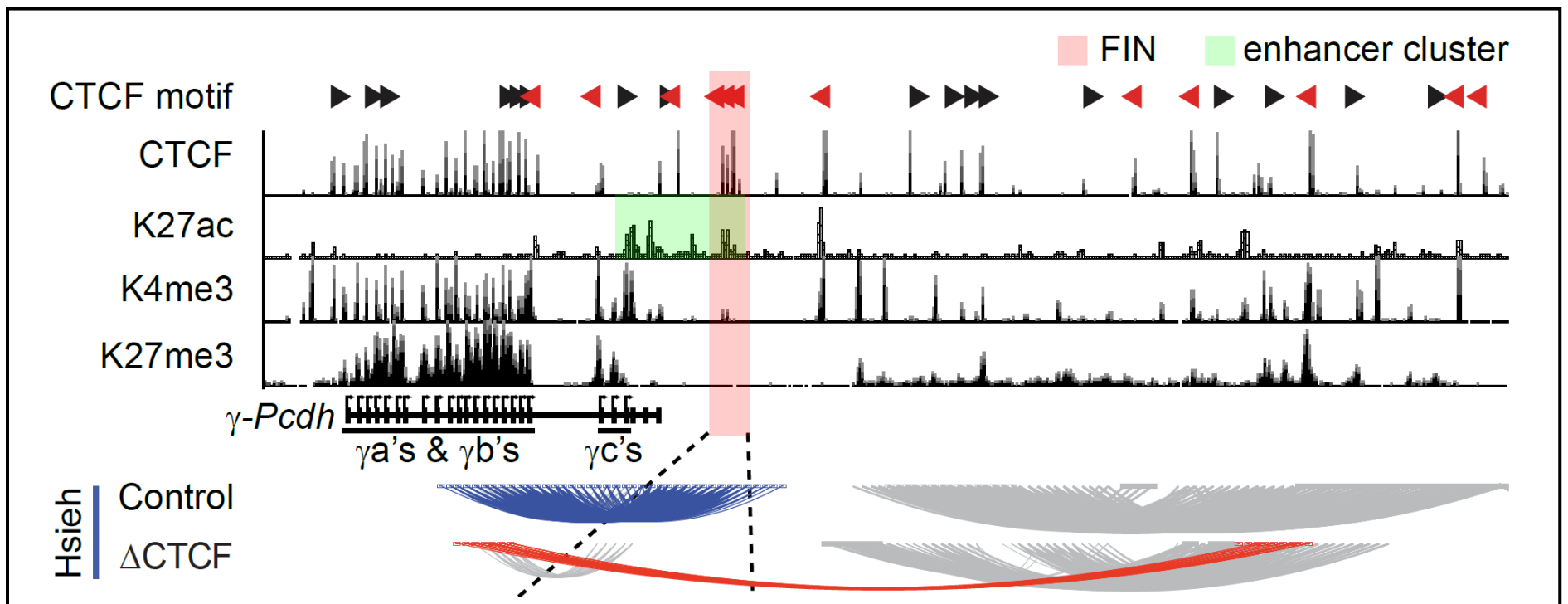

Reviewer Fig. 2. Many H3K27me3 peaks at Pcdh-γ locus form a domain-like structure. Note that this locus also have high density of H3K4me3 and CTCF peaks. Figure adopted from **Fig. 4j** in our manuscript.

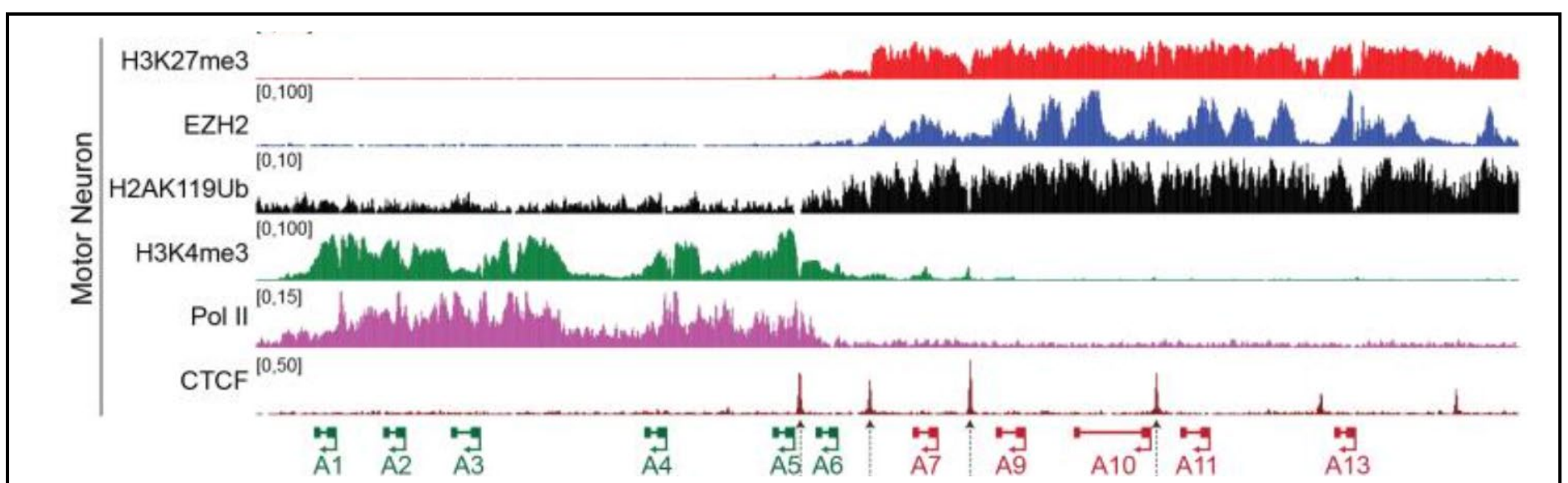

Reviewer Fig. 3. A classic example of "polycomb domain" at HoxA locus. In motor neuron, the polycomb domain marked by H3K27me3 lacks H3K4me3 mark, and the genes (from HoxA7 to HoxA13) covered by this polycomb domain are all repressed. Figure adopted from Narendra et al., Science 2015, PMID:25722416.

We appreciate the reviewer's insight regarding the H3K27me3 enrichment at the *Pcdh* locus.

While the cluster of H3K27me3 peaks at the *Pcdh-γ* locus resembles a Polycomb-repressed domain, this locus is also marked by prominent H3K4me3 and CTCF peaks (**Reviewer Fig. 2**). Furthermore, the *Pcdh-γ* genes exhibit detectable transcription in wild-type mESCs (**Fig. 4l**). In contrast, canonical polycomb domains (e.g., the HoxA cluster, **Reviewer Fig. 3**) lack active H3K4me3 marks and remain transcriptionally silent. Therefore, we interpret the H3K27me3 peaks at *Pcdh-γ* genes as a cluster of "bivalent" or "poised" promoters (Bernstein et al., Cell 2006, PMID: 16630819) rather than a constitutive Polycomb-repressed domain.

Our data support a model of localized loop mis-regulation rather than a domain-wide epigenetic shift. Under baseline conditions, the *Pcdh-γ* genes interact with a nearby enhancer cluster (highlighted in green, **Reviewer Fig. 2**) that is spatially confined by a FIN (highlighted in pink, **Reviewer Fig. 2**). Following CTCF depletion, the loss of this FIN-mediated insulation disrupts these enhancer-promoter interactions, leading to the observed downregulation of

Pcdh-γ genes. This mechanism is directly supported by our experimental validation, in which the targeted deletion of the FIN resulted in the downregulation of *Pcdh-γ* genes (**Fig. 4k,l**).

6. Given the targets in the motif enrichment, I'm curious to whether any of these factors are differentially regulated upon CTCF/Rad depletion – in the depleted anchors for example, there is a considerable amount of homeobox/homeodomain containing factors.

This is an interesting question. In our manuscript (**Fig. 3l**), we show that loop-gaining ATAC-seq peaks are enriched with G-rich motifs and depleted of A/T-rich motifs (such as those for homeodomain factors). Our interpretation is that G-rich enhancers and promoters are intrinsically more likely to act as loop anchors upon CTCF depletion due to their superior capability to stall cohesin, assuming relatively stable expression of the associated transcription factors (TFs).

To address the review's question, we examined the expression changes of these putative TFs across three independent CTCF-depletion datasets (**Reviewer Fig. 4**). We found that none of the TFs associated with "enriched" motifs show more than a 2-fold change in any of the three datasets. While a few "depleted" TFs show changes exceeding 2-fold, these are often inconsistent across studies—for instance, *Gata5* is downregulated in the Nora et al. dataset but upregulated in Hsieh et al. (**Reviewer Fig. 4**). Additionally, although two TFs (*Foxd1* and *Cdx1*) are upregulated in two of the three studies, the magnitude of these changes is modest at slightly more than 2-fold. These observations suggest that TF expression fluctuations are unlikely to drive the loop rewiring observed in CTCF-depleted cells.

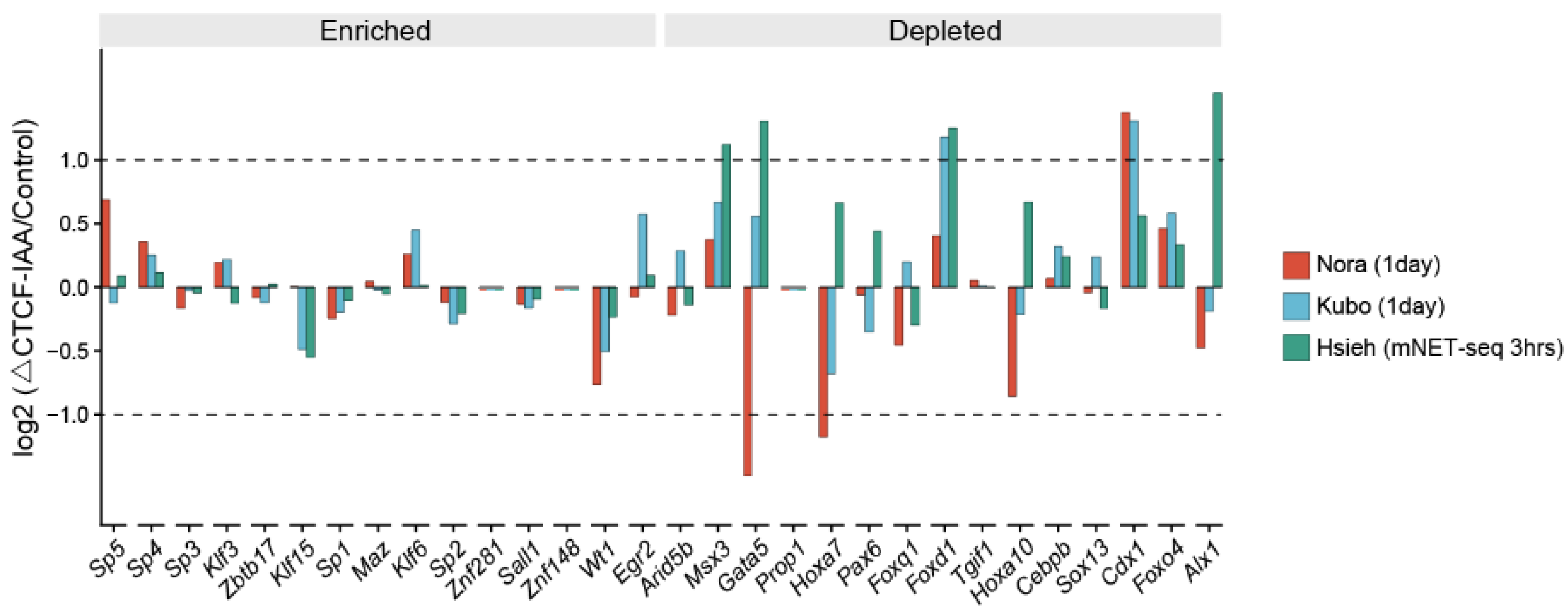

Reviewer Fig. 4. The expression changes of TFs across three CTCF-degron studies. Enriched/Depleted groups are the TFs listed in **Fig. 3l** that are enriched/depleted at loop-gaining ATAC-peaks upon CTCF-depletion.
